# Cobalt-Catalyzed Three-Component Reductive Coupling of Aldehydes, Allenes, and Aryl Halides

**DOI:** 10.1021/acs.orglett.6c00927

**Published:** 2026-03-27

**Authors:** Joshua D. Sieber, Magdalene Togoh, Sara Azad, TyAnn McHenry, Murell Sanders

**Affiliations:** Department of Chemistry, Virginia Commonwealth University, 1001 West Main Street, Richmond, Virginia 23284-3028, United States

## Abstract

A Co-catalyzed reductive three-component coupling of aldehydes, allenes, and aryl halides is described. The reaction is both regio- and stereoselective and produces stereodefined trisubstituted alkenylsilanes that can be converted into trisubstituted alkenes. Broad functional group tolerance was demonstrated through 40 examples, with an oxidative addition mechanism proceeding through halide abstraction.

## Graphical abstract

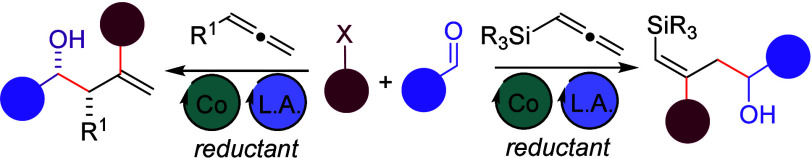

Over the years, allylation reactions have been extensively developed and shown to be powerful tools for the synthesis of complex organic compounds.^1^ Of particular significance in these processes is the fact that the intermediate substituted allylic organometallic reagents used (e.g., **2** (Figure 1A)) can be rendered either electrophilic^2^ or nucleophilic^3^ through the choice of the metal employed in the reaction. In the nucleophilic mode, allyl addition to aldehyde or imine electrophiles has been extensively developed to access homoallylic alcohols or amines (**3**), respectively,^1a,1c,3^ whereas the electrophilic mode is as synthetically powerful, enabling coupling with nucleophiles in allylic alkylation processes (**4**).^1b,2^ Both of these reaction classes, as well as techniques to access the necessary allylic organometallic intermediate (**2**), have been widely studied by numerous groups over the past 60 years.^1–3^ More recently, there has been growing interest in accessing organometallic **2** in a catalytic fashion, often through hydrometalation of an unsaturated hydrocarbon coupling partner (e.g., allene **1**).^3c–3e,4^ Such techniques are highly valuable, as they can increase the atom economy and improve functional group tolerance in common allylation processes (both electrophilic and nucleophilic). However, a majority of these reactions utilize either hydrometalation^3c–3e,4^ or borylmetalation^5^ of the unsaturated hydrocarbon to arrive ultimately at products having only H or B substitution, respectively. This limits the diversity that can be achieved since the same reductant (H or B) must be incorporated into the final product. In contrast, if the allylic organometallic intermediate could be accessed through carbometalation of the allene by a M–C transition metal intermediate,^6,7^ a third carbon-containing substituent could be added in the process to enable increased product diversity through a three-component coupling (TCC) (Figure 1B). Catalytic access to the requisite M–C intermediate could be feasible from either oxidative addition of a metal catalyst to an alkyl or aryl halide coupling partner^6^ or transmetalation with a nucleophilic donor (e.g., organoboronates).^7^ Processes for the TCC of aryl halides, allenes, and nucleophiles have been developed using Pd catalysis.^6a^ Here oxidative addition of the electrophilic aryl halide by Pd followed by insertion of the neutral allene conveniently affords a Pd­(π-allyl) intermediate prone to allylic alkylation with nucleophiles (e.g., amines and malonates), affording **8**. Furthermore, due to the recent interest in energy transfer using light, the same redox-neutral processes can now be achieved using alkyl halides in the presence of LED irradiation.^8^ However, metal-catalyzed TCC reactions generating a nucleophilic allyl organometallic reagent *in situ* through carbometalation for subsequent coupling to an electrophilic coupling partners to afford **9** are much less developed.^6c–6e,7^ While redox-neutral TCC of arylboronoates, allenes, and aldehydes using Pd catalysis has been disclosed;^7^ our interest was in the development of TCC reactions enabling the coupling of two electrophilic fragments with a neutral unsaturated hydrocarbon (e.g., allene **1**) in an overall reductive process.^9^ Accordingly, we considered that a stoichiometric reductant will be necessary to achieve this proposed reductive three-component coupling (RTCC) reaction. Herein, we disclose our work on the development of a Co-catalyzed reductive three-component coupling of allenes, aldehydes, and aryl halides (RTCC-3A), affording stereodefined branched and linear homopropargylic alcohols (Figure 1C).

**Figure 1:**
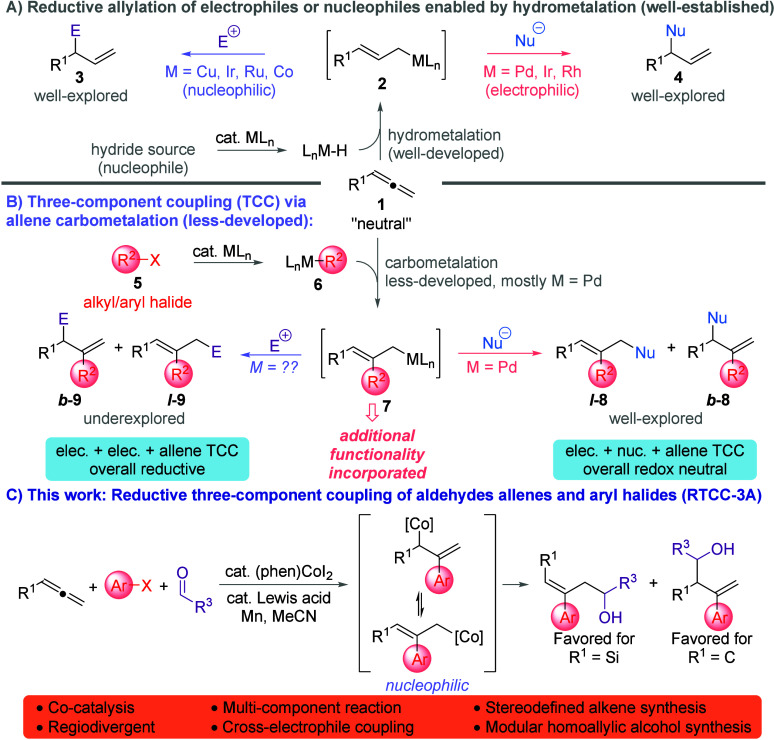
(A) Metal-catalyzed reductive allylation. (B) Three-component coupling through carbometalation. (C) Reductive three-component coupling.

To initially pursue this idea, we chose to investigate Co as the metal catalyst of choice based on recent works of Li^10^ and Meng^11^ that demonstrate that Co­(allyl) intermediates can be electrophilic or nucleophilic in nature based on the oxidation state at Co. When using a stoichiometric reductant, Co­(I)–allyl complexes that are nucleophilic toward aldehyde electrophiles form. Therefore, we envisioned the RTCC-3A process could proceed using a Co­(I) catalyst through oxidative addition of the aryl halide, followed by allene insertion and reduction to generate a nucleophilic substituted Co­(I)–allyl complex that would add to the aldehyde coupling partner, providing branched or linear TCC products (Figure 1C).^12^ A previous report by Komeyama^6e^ demonstrated a RTCC-3A process cocatalytic in both Co and Cr using carbon-substituted allenes to afford branched products. Here, the intermediate allyl–Co complexes were not nucleophilic enough to add to the aldehyde electrophile, which was circumvented by transmetalation to the Cr cocatalyst, enabling aldehyde allylation. In this work, we hypothesized that aldehyde allylation may be achieved through activation of the aldehyde by addition of a Lewis acid cocatalyst. Gratifyingly, this rationale proved to be successful, and optimal conditions^13^ identified (phen)­CoI_2_ as the catalyst using Mn as the stoichiometric reductant in conjunction with a Lewis acid cocatalyst (Scheme 1). No reaction was observed in absence of the Lewis acid cocatalyst, and the regiochemical outcome was controlled by the allene employed in the reaction. Allenyl silanes provided linear products (**10**), whereas carbon-substituted allenes afforded branched product **11**.^12,13^

**Scheme 1:**
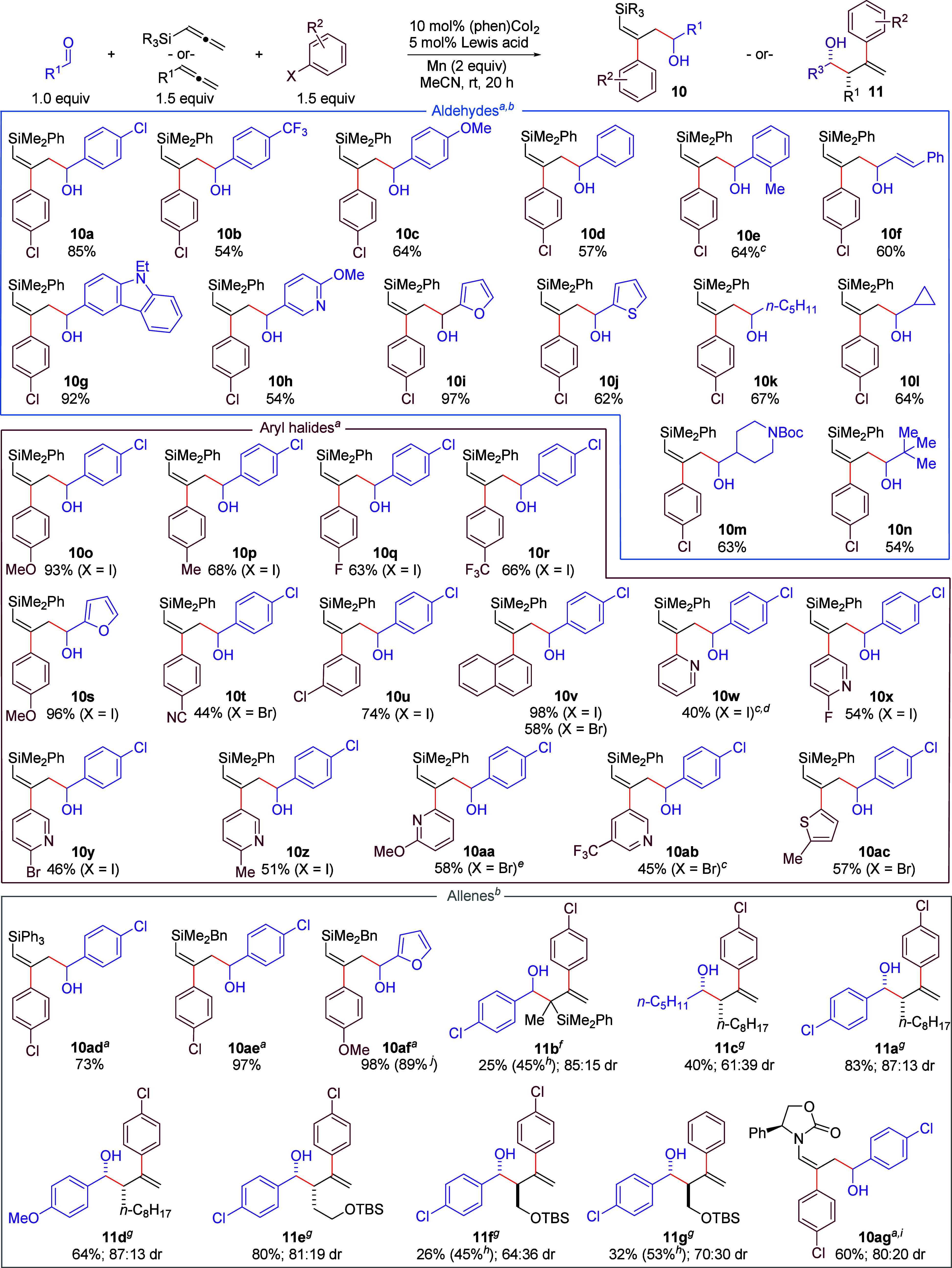
Substrate Scope of the RTCC-3A Reaction^13^

a Mg­(OTf)_2_ as the Lewis acid; >95:5 *E*:*Z* obtained unless otherwise noted.
b Aryl iodide used (X = I).
c At 80 °C.
d 93:7 *E*:*Z* obtained.
e 93:7 **10aa**:**11aa**, 90:10 *E*:*Z* obtained.
f Eu­(OTf)_3_ as the Lewis acid and X = I.
g Sn­(OTf)_2_ as the Lewis acid and X = I.
h Yield determined by ^1^H NMR spectroscopy.
i From rt to 60 °C.
j Performed on a 1.0 mmol scale using 5 mol % Co.

Investigation of the scope of the Co-catalyzed RTCC-3A reaction primarily focused on the use of allenyl silanes (**10a–10af**). For these cases, a variety of aldehydes participated well in the coupling (**10a**–**10n**). Aromatic electron-deficient (**10a** and **10b**), electron-rich (**10c**), and *ortho*-substituted (**10e**) aldehydes were all tolerated well as were heterocyclic (**10g**–**10j**) and aliphatic aldehydes (**10k**–**10n**). A variety of aryl halides were also well tolerated in the reaction (**10o–10ac**), giving variable yields. In general, heterocyclic aryl halides (e.g., **10w–10ac**) afforded lower yields then simple benzene-substituted aryl halides (**10o**–**10v**). In particular, halopyridines having *ortho* substitution at the N atom were more efficient, implying that catalyst inhibition by N binding of the heterocycle may be responsible for the reduced reaction efficiency (**10w** vs **10x**–**10aa**). Aryl bromides could be used rather than aryl iodides, albeit with reduced efficiency (**10t**, **10v**, and **10aa**–**10ac**). Notably, the reaction was chemoselective for the C–I bond in aryl halides bearing both an iodo group and another halogen group (**10u**, **10x**, and **10y**). Such a feature allows for the possibility of downstream processing of the remaining C–X bond using alternative chemistries. A pendent nitrile group on the aryl halide was also tolerated (**10t**). In the context of the allene coupling partner, other allenyl silanes could be used with nearly identical efficiencies (**10ad**–**10af**), and the use of a 1,1-disubstituted allenyl silane afforded a reversal in regioselectivity to provide branched product **11b** in modest yield and diastereoselectivity. Performing the reaction on a 1.0 mmol scale using 5 mol % Co provided similar results (**10af**). Using a carbon-based allene, a similar yield and a similar diastereoselectivity were obtained with electron-deficient or electron-rich aromatic aldehydes (**11a** and **11d**), but aliphatic aldehydes provided a reduced yield and a reduced diastereoselectivity (**11c**). Two allenes bearing a pendant TBS ether group were also tested and an impact on the spatial distance of this group from the allene on reaction efficiency and stereoselectivity was observed (**11e** vs **11f** and **11g**). A switch in stereoselectivity for the *anti* diastereomer for **11f** and **11g** was obtained and has been rationalized previously based on dipole effects of the heteroatom substituent.^6e^ Finally, the use of a chiral allenamide^14^ afforded linear product **10ag** in good diastereoselectivity at the newly formed alcohol stereocenter.

To gain insight into the oxidative addition mechanism for Co in this system, the competition experiment outlined in Scheme 2A was performed. Previous mechanistic studies on the oxidative addition of aryl and alkyl halides to Co complexes by Budzelaar^15^ and Chirik^16^ support a halide abstraction followed by a radical capture mechanism; however, single-electron transfer (SET) from the transition metal to the arene followed by *ipso* halide loss is also a possible pathway.^17^ To distinguish between these two possibilities, the competition experiment between 1-fluoro-4-iodobenzene and 4-iodobenzotrifluoride was performed based on a recent report by Hartwig.^17^ Because 1-fluoro-4-iodobenzene and 4-iodobenzotrifluoride have similar C–I bond strengths but 4-iodobenzotrifluoride is easier to reduce (more positive *E*_red_), high selectivity for 4-iodobenzotrifluoride over 1-fluoro-4-iodobenzene indicates an oxidative addition mechanism proceeding through an SET pathway.^17^ Only 65:35 selectivity for **10r**:**10q** was observed, indicating that an SET pathway is not likely. Furthermore, 1-chloro-3,5-bis­(trifluoromethyl)­benzene was also tested in the reaction and afforded no product. If an SET oxidative addition mechanism was operable, this aryl halide would be expected to participate since it has a reduction potential between those of the reactive 1-halonaphthalene coupling partners.^17^ Together, these results are more consistent with the relative C–X bond strengths and support a halide abstraction process for oxidative addition.

**Scheme 2:**
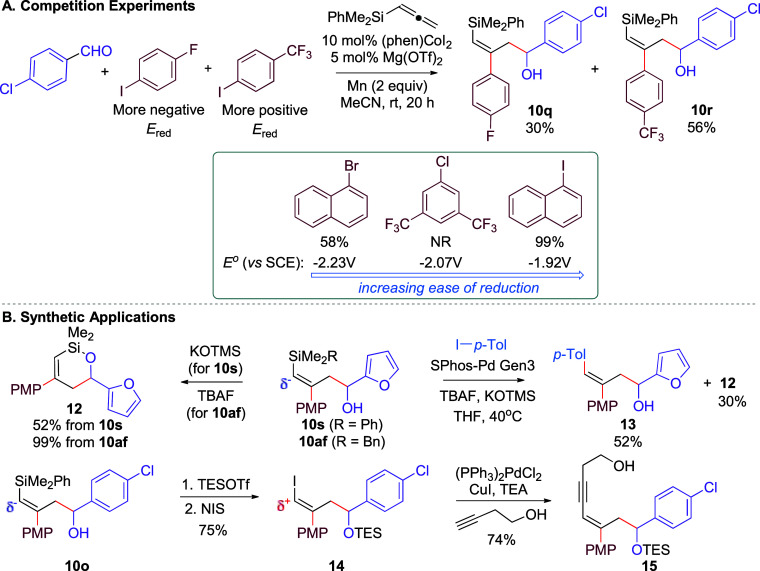
Competition Experiment and Applications

The RTCC-3A reaction utilizing allenyl silanes offers a convenient technique to access complex stereodefined trisubstituted alkenes (Scheme 2B). Notably, alkenylsilanes are versatile intermediates for synthesis, since the C–Si bond can be employed as a nucleophilic or electrophilic fragment. For instance, the polarity of the C–Si bond is nucleophilic at carbon, enabling cross-coupling with aryl halides via Hiyama–Denmark coupling.^18^ First, cleavage of a Si–C bond to access cyclic siloxane **12** needed for Hiyama–Denmark cross-coupling could be achieved.^18^ Subsequently, a one-pot Hiyama–Denmark method using **10af** was demonstrated to afford stereodefined trisubstituted styrene **13** in moderate yield along with recovery of siloxane **12**. Alternatively, the C–Si bond could be transformed into a C–I bond using NIS to access electrophilic vinyl iodide **14**. Subsequent Sonogashira coupling with but-3-yn-1-ol furnished access to stereodefined enyne **15** in good yield.

In conclusion, we have disclosed a Co-catalyzed reductive three-component coupling reaction of aldehydes, allenes, and aryl halides that operates under mild conditions, affording useful reaction products in a stereoselective and regioselective fashion. A wide variety of aldehydes, allenes, and aryl halides could be employed in the reaction, and the synthetic utility of the vinylsilane products produced were demonstrated. Oxidative addition by the Co catalyst in these processes likely occurs through the expected halide abstraction mechanism.

## Supplementary Material



## Data Availability

The data underlying this study are available in the published article and its Supporting Information.

## Notes

Experimental procedures, optimization data, proposed catalytic cycle, analytical data, and NMR spectra (PDF)
