# Supplementary material for: Cobalt-Catalyzed Three-Component Reductive Coupling of Aldehydes, Allenes, and Aryl Halides
Source: Org Lett. 2026 Mar 27;28(14):4585–90. doi: 10.1021/acs.orglett.6c00927 (PMC13077677; doi:10.1021/acs.orglett.6c00927)
Supplement: Supplementary file 1 [file ol6c00927_si_001.pdf]

Supporting Information

Cobalt-Catalyzed Three-Component Reductive Coupling of Aldehydes, Allenes and Aryl Halides.

Joshua D. Sieber,*[,a] Magdalene Togoh,[a] Sara Azad,[a] TyAnn McHenry,[a] Murell Sanders[a]

[a]*Department of Chemistry, Virginia Commonwealth University, 1001 West Main St., Richmond, VA 23284-3028, USA*

*Correspondence to: jdsieber@vcu.edu

Table of Contents:

General……………………………………………………………………………………S2

General Procedures……………………………………………………………………………S2

Optimization tables…………………………………………………………………………… S3-S5

Proposed catalytic cycle and explanation of regiochemical control………………..………………… S6-S7

Product characterization data...................................………………………………………………….S8-S22

1.0 mmol scale reactions………………………………………………………………………… S22

Competition experiment………………………………………………………………………… S23

Synthetic applications…..…………………………………………………………………...S23-S26

^{1}H and ^{13}C NMR data………………………………………………………………….S27-S71

References.. S72

General. ^{1}H NMR spectra were recorded on Bruker 600 MHz spectrometers. Chemical shifts are reported in ppm from tetramethylsilane with the solvent resonance as an internal standard ($CDCl_3$: 7.26 ppm). Data are reported as follows: chemical shift, integration, multiplicity (s = singlet, d = doublet, t = triplet, q = quartet, p = pentet, h = hextet, hept = heptet, br = broad, m = multiplet), and coupling constants (Hz). ^{13}C NMR was recorded on a Bruker 600 MHz (151 MHz) instrument with complete proton decoupling. Chemical shifts are reported in ppm from tetramethylsilane with the solvent as the internal standard ($CDCl_3$: 77.0 ppm). Liquid chromatography was performed using forced flow (flash chromatography) on silica gel purchased from Silicycle. Thin layer chromatography (TLC) was performed on glass-backed 250 µm silica gel F254 plates purchased from Silicycle. Visualization was achieved using UV light, a 10% solution of phosphomolybdic acid in EtOH, or potassium permanganate in water followed by heating. HRMS was collected using a Jeol AccuTOF-DART^{TM} mass spectrometer using DART source ionization. All reactions were conducted in oven or flame dried glassware under an inert atmosphere of nitrogen or argon with magnetic stirring unless otherwise noted. Acetonitrile was anhydrous grade purchased from Sigma Aldrich. Other solvents were obtained from VWR as HPLC grade and transferred to septa sealed bottles, degassed by Ar sparge, and analyzed by Karl-Fischer titration to ensure water content was $\leq$ 600 ppm. (phen)CoI_2 was prepared according to the literature.[1] Cobalt salts were purchased from Strem Chemical company. Aldehydes were purchased from Sigma Aldrich, TCI America, Alfa Aesar, Combi-Blocks, or Oakwood Chemicals. Liquid aldehydes were distilled before use. Mn powder was 325 mesh from Alfa Aesar. Silyl-allenes were prepared from the silylchloride and propargyl bromide in the presence of In powder (325 mesh) according to the published protocols.[2] Carbon-based allenes were prepared using the Crabbe reaction as previously reported.[3] The Evans-auxiliary derived allenamide was prepared according to the literature.[4] All other materials were purchased from VWR, Sigma Aldrich, Combi-Blocks, Alfa-Aesar, or Strem Chemical Company and used as received.

General procedure.

To a flame-dried 2-dram screw-cap vial with magnetic stir-bar in an Ar-filled glove-box was charged 6.7 mg (0.013 mmol, 10 mol%) of (phen)CoI_2, 2.2 mg (0.0068 mmol, 5 mol%) $Mg(OTf)_2$ and 14.9 mg (0.270 mmol, 2 equiv) of Mn. Acetonitrile (0.43 mL) was then added, and the mixture was allowed to stir for 5 min. To the mixture was sequentially added aryl halide (0.203 mmol, 1.5 equiv), allene (0.203 mmol, 1.5 equiv), and aldehyde (0.135 mmol, 1.0 equiv). The vial was sealed with a screw-cap, removed from the glove-box, and allowed to stir rapidly at ambient temperature for 20 h. Methyl *tert*-butyl ether (MTBE, 5 mL) was then added, and the mixture was filtered through a small pad of silica gel rinsing with additional MTBE (~5 mL). The combined organics were washed with 2.5% Aq. NH_4OH (2x5mL), dried with Na_2SO_4 and concentrated *in vacuo*. The crude residue was then purified by flash chromatography on silica gel using hexanes/EtOAc mixtures. All linear products (**10**) were obtained in >95:5 *E:Z* diastereopurity unless otherwise specified.

Table SI-1: Ligand survey.

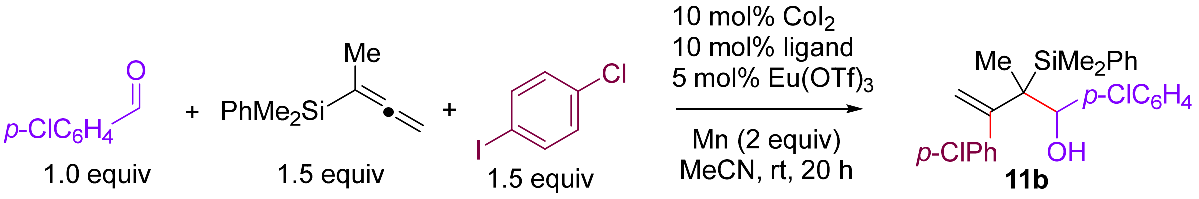

Entry	Ligand	% aldehyde[a]	% allene[a]	% yield 11b[a]	dr[a]
1	1,10-Phen	15	22	44	85:15
2	4,7-Dimethyl-1,10-Phen (CAS# 3248-05-3)	6	12	16	84:16
3	Tetramethyl-1,10-Phen (CAS# 1660-93-1)	28	46	23	85:14
4	4,7-Dihydroxy-1,10-phen (CAS# 3922-40-5)	0	0	7.5	50:50
5	2,9-Dichloro-1,10-phen (CAS# 29176-55-4)	5.9	0.5	8.5	80:20
6	Neocuproine (CAS# 484-11-7)	21	34	15	87:13
7	2,2′-Bipyridyl (CAS# 366-18-7)	13	26	21	80:20
8	6,6′-Dimethyl-2,2′-dipyridyl (CAS# 4411-80-7)	34	45	0	0
9	4,4′-Di-tert-butyl-2,2′-dipyridyl (CAS# 72914-19-3)	6.9	19	16	83:17
10	4-4′-Dimethoxy-2-2′-bipyridine (CAS# 17217-57-1)	16	22	7.9	86:14
11	[2,2'-Bipyridine]-4,4'-dicarbonitrile (CAS# 67491-43-4)	19	23	7.3	83:16
12	Dppbz	9.6	72	0	0
13	PPh_3	51	62	8	87:13
14	Dppb	57	76	0	0
15	Dppf	4.7	49	7.9	89:10
16	Bis-(trimethylbenzylidene)hydrazine	3.9	33	17	88:12

[a]Determined on the crude reaction mixture by quantitiative ^{1}HNMR spectroscopy relative to 1,3,5-trimethoxybenzene as analytical standard.

Table SI-2: Lewis acid survey.

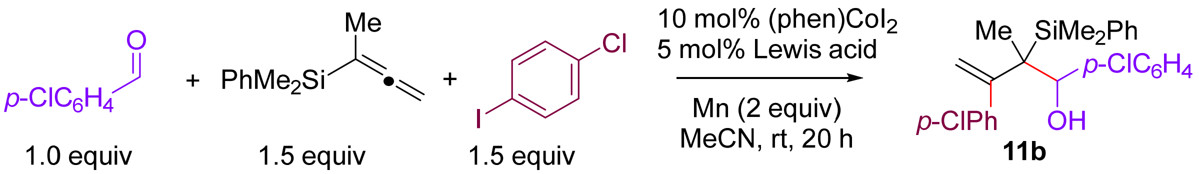

Entry	Lewis Acid	% aldehyde[a]	% allene[a]	%yield 11b[a]	dr[a]
1	$Sc(OTf)_3$	15	20	22	88:12
2	$Sm(OTf)_3$	5.8	21	17	80:20
3	$In(OTf)_3$	12	30	18	80:20
4	$La(OTf)_3$	34	65	0	-
5	$Gd(OTf)_3$	53	83	0	-
6	$BF_3•OEt_2$	16	37	20	86:14
7	$Sn(OTf)_2$	43	63	4	84:16
8	$Mg(OTf)_2$	61	n.d.	23	85:15
9	$Eu(OTf)_3$	15	22	44	85:15
10[b]	$Eu(OTf)_3$	26	33	5	79:21
11[c]	$Eu(OTf)_3$	29	45	3	87:13

[a]Determined on the crude reaction mixture by quantitiative ^{1}HNMR spectroscopy relative to 1,3,5-trimethoxybenzene as analytical standard. [b]Uses Zn instead of Mn. [c]Uses In instead of Mn.

Table SI-3. Lewis acid survey using a terminal silyl allene.

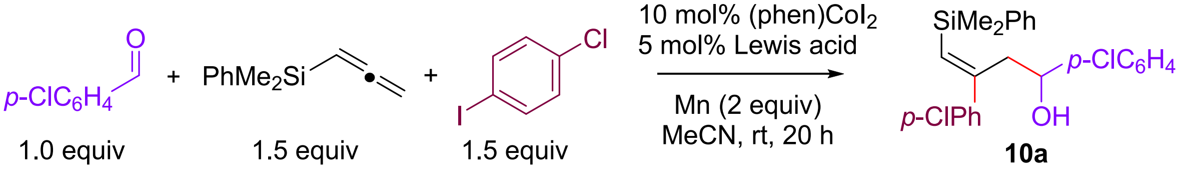

10a

Entry	Lewis Acid	% yield 10a[a,b]
1	None	0
2	$Sc(OTf)_3$	74
3	$In(OTf)_3$	65
4	$Sn(OTf)_2$	62
5	$Eu(OTf)_3$	90
6	$Mg(OTf)_2$	87

[a]Yield determined by ^{1}H NMR spectroscopy on the unpurified reaction mixture using 1,3,5-trimethoxybenzene as standard. [b]Obtained in >95:5 *E*:*Z*

Table SI-3. Lewis acid survey using a carbon-substituted allene.

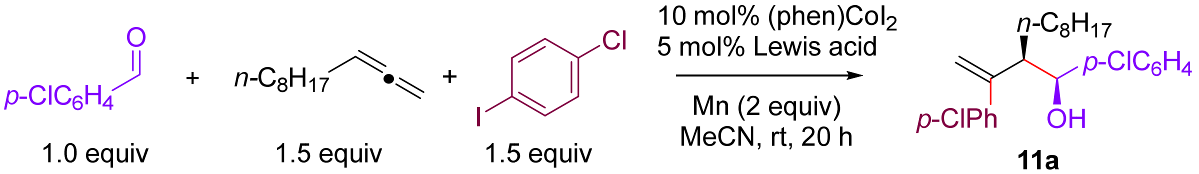

11a

Entry	Lewis Acid	% aldehyde[a]	% allene[a]	%yield 11a[a]	dr[a]
1	None	n.d.	n.d.	0	
2	$Sc(OTf)_3$	15	0	68	87:13
3	$Sm(OTf)_3$	3.4	0	64	85:15
4	$La(OTf)_3$	2.7	0	55	86:14
5	$Eu(OTf)_3$	0	0	79	86:14
6	$Gd(OTf)_3$	59	91	0	-
7	$In(OTf)_3$	3.9	0	72	84:16
8	$Mg(OTf)_2$	0	0	86	87:13
9	LiOTF	3.6	0	69	84:16
10	$Cu(OTf)_2$	2.1	0	67	85:15
11	$Sn(OTf)_2$	0	0	87	86:14
12	ZnI_2	64	86	0	0
13	$B(C_6F_5)_3$	59	83	0	0
14	$MgCl_2$	1.5	0	65	86:14
15	TMSOTf	1.8	0	24	85:15
16	$TiCl_4$	5.2	0	58	86:14
17	BF_3-OEt_2	4.5	0	60	85:15

[a]Determined on the crude reaction mixture by quantitiative ^{1}HNMR spectroscopy relative to 1,3,5-trimethoxybenzene as analytical standard.

Table SI-4. Solvent investigation.

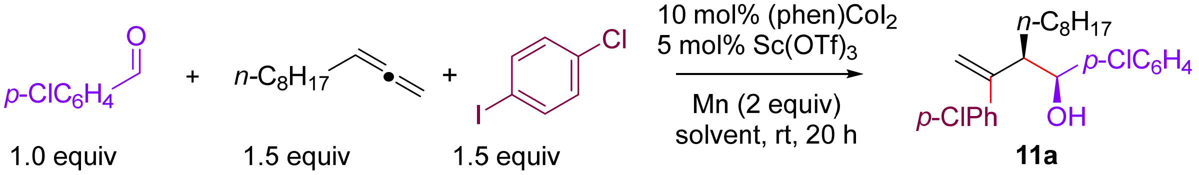

Entry	Solvent	% aldehyde[a]	% allene[a]	%yield 11a[a]	dr[a]
1	MeCN	15	0	68	87:13
2	DMF	32	60	0	N/A
3	NMP	17	58	0	N/A
4	DMAc	6	75	0	N/A
5	DMSO	56	84	0	N/A
6	THF	47	70	0	N/A

[a]Determined on the crude reaction mixture by quantitiative ^{1}HNMR spectroscopy relative to 1,3,5-trimethoxybenzene as analytical standard.

Table SI-5. Preliminary chiral ligand investigation.

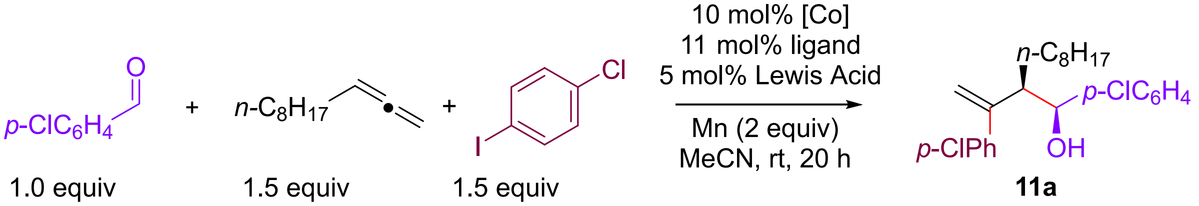

Entry	[Co]	Ligand	Lewis Acid	%yield 11a[a]	*syn:anti*[b]	er *syn/anti*[c]
1	CoI_2	**L1**	$Sc(OTf)_3$	45	86:14	rac / rac
2	CoI_2	**L2**	$Sc(OTf)_3$	< 10%	88:12	n.d.
3	CoI_2	**L3**	$Sc(OTf)_3$	69	86:14	rac / rac
4	(phen)CoI_2	--	$Sc(OTf)_3$ + **L2**	82	89:11	53:47 / 53:47
5	(phen)CoI_2	--	$Sc(OTf)_3$ + **L4**	87	86:14	rac / rac

[a]Isolated yield of combined diastereomers. [b]Determined on the crude reaction mixture by ^{1}HNMR spectroscopy. [c]Determined by HPLC on chiral stationary phase; AD-3 x 250 mm, 97:3 hexanes:isopropanol, 1.0 mL/min isocratic, t_R: 9.0/9.6 min (minor diast., *anti*) and 14.8/22.2 min (major diast., *syn*).

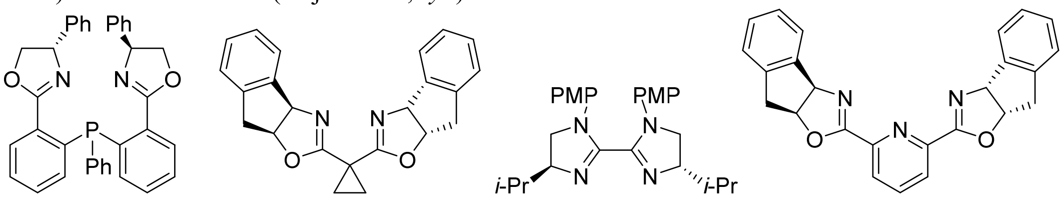

L1 **L2** **L3** **L4**

Proposed catalytic cycle and regiochemical model:

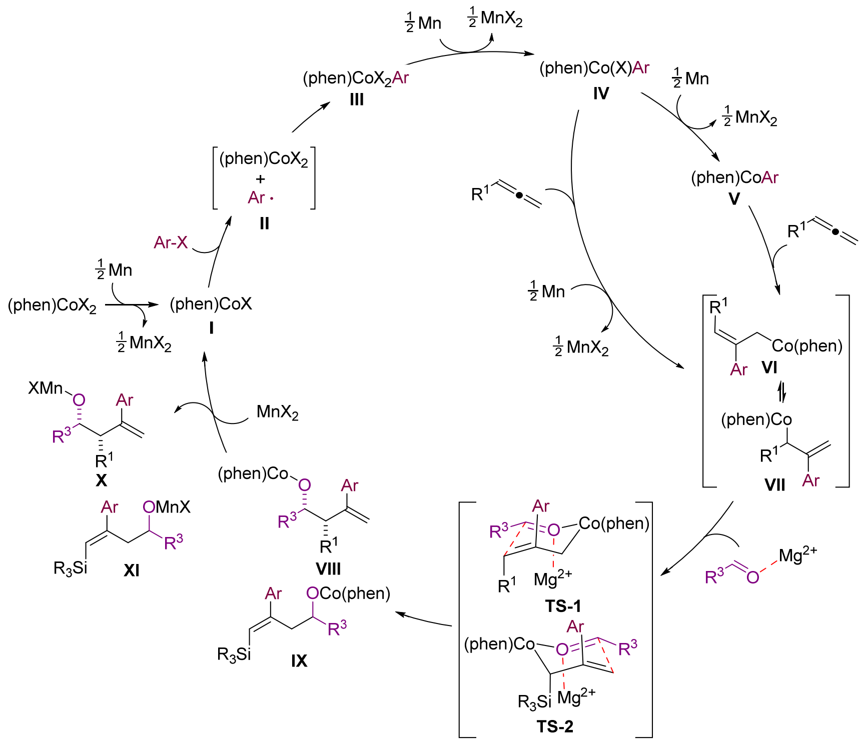

Figure S-1: Proposed catalytic cycle.

Reduction of the Co(II) pre-catalyst by Mn affords Co(I) complex **I** that undergoes oxidative addition to ultimately afford Ar-Co(III) complex **III**.[5] Oxidative addition likely goes through halide abstraction (**II**) followed by radical capture based on previous mechanistic studies by Budzelaar[6] and Chirik.[7] The intermediate Co(III)-complex **III** is subsequently reduced by Mn to Co(II) complex **IV** that ultimately must undergo carbometalation of the allene to afford nucleophilic Co(I) complexes **VI** and/or **VII**.[5b] This may occur through first reduction of **IV** to Co(I)-Aryl **V** followed by allene insertion, or inversion of the reaction steps: allene insertion followed by reduction. Nucleophilic allylic Co(I) complexes **VI** and **VII** may then undergo allylation of the Lewis-acid activated aldehyde through chair-like transition structures **TS-1** and **TS-2** to arrive at Co-alkoxide products **VIII** and **IX** depending on the nature of the R^1-substituent of the allene, respectively. For C-based allenes, chair transition structure **TS-1** predominates by way of Co-complex **VI** due to the minimization of steric effects furnishing branched intermediate **VIII**. In contrast, due to the α-anion stabilizing effect of the Si-atom,[8] allenyl silanes favor reaction through **VII** (R^1 = SiR_3) via **TS-2** having the SiR_3 group in an axial orientation to avoid an $A^{1,2}$-interaction with the Ar-group[9] and due to the elongated C-Si bond[10] ultimately providing the *E*-linear intermediate **IX**. These chair-like transition structures account for the observed good stereocontrol in these processes. Finally, salt-metathesis of **VIII** or **IX** with MnX_2 regenerates catalyst **I** and affords the Mn-alkoxides **X** or **XI**, respectively that are protonated during workup.

When using a 1,1-disubstituted silylallene (*e.g.* **A**, Figure S-2), turnover to afford the branched product (**11b**) is presumably due to increased steric penalty in **TS-2a** due to the presence of a fully substituted C-atom next to the Co-coordination sphere resulting in destabilization of this transition structure relative to **TS-1a** (Figure S-2).

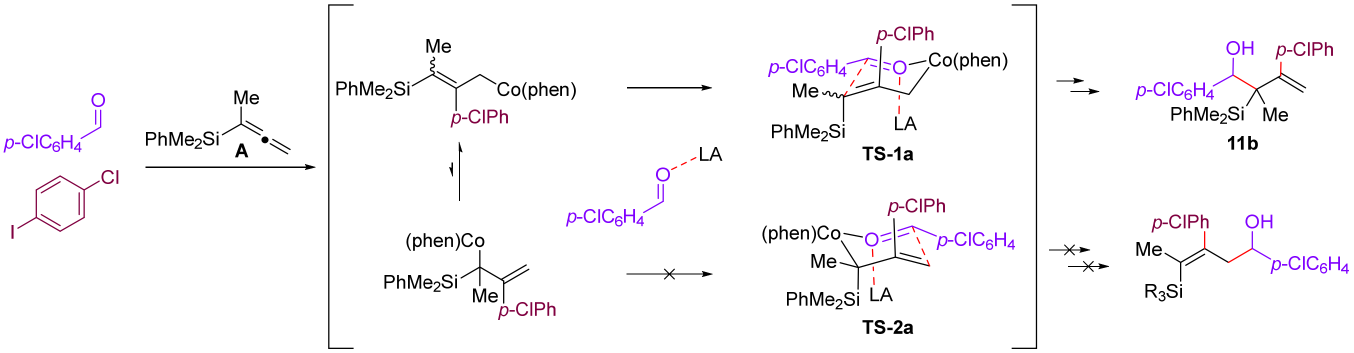

Figure S-2: Branched selectivity with 1,1-disubstituted silylallene.

Analytical data

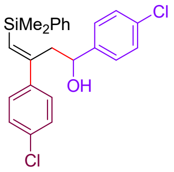

(*E*)-1,3-bis(4-chlorophenyl)-4-(dimethyl(phenyl)silyl)but-3-en-1-ol (10a): According to the general procedure using $Mg(OTf)_2$ as Lewis acid and 1-chloro-4-iodobenzene, purification by silica gel chromatography (eluent: 0 – 10% EtOAc in hexanes) afforded 49.0 mg (85%) of **10a** as a colorless thick oil in >95:5 *E:Z*. The stereochemistry was assigned by analogy to that of **10i**. R_f = 0.25 (15% EtOAc/hexanes). ^{1}HNMR ($CDCl_3$, 600 MHz) δ: 7.54 – 7.58 (m, 2H), 7.36 – 7.51 (m, 3H), 7.34 (d, J = 8.9 Hz, 2H), 7.32 (d, J = 8.9 Hz, 2H), 7.21 (d, J = 8.5 Hz, 2H), 6.98 (d, J = 8.5 Hz, 2H), 6.05 (s, 1H), 4.49 (dd, J = 8.7 Hz, 5.1 Hz, 1H), 2.87 (dd, J = 14 Hz, 8.7 Hz, 1H), 2.86 (dd, J = 14 Hz, 5.1 Hz, 1H), 1.57 (br s, 1H), 0.45 (s, 3H), 0.44 (s, 3H); $^{13}C\{^1H\}$NMR (151 MHz, $CDCl_3$): δ 153.3, 142.3, 141.6, 139.0, 133.8, 133.6, 133.1, 131.8, 129.2, 128.6, 128.4, 128.1, 127.7, 126.9, 71.6, 44.6, –0.8, –1.0 ppm. HRMS (DART) *m/z* calcd for $C_{24}H_{25}Cl_2OSi$ [M+H]$^+$: 427.1052; Found [M + H]$^+$: 427.1081.

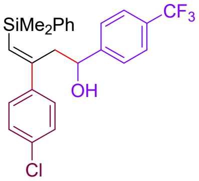

(*E*)-3-(4-chlorophenyl)-4-(dimethyl(phenyl)silyl)-1-(4-(trifluoromethyl)phenyl)but-3-en-1-ol (10b): According to the general procedure using $Mg(OTf)_2$ as Lewis acid and 1-chloro-4-iodobenzene, purification by silica gel chromatography (eluent: 0 – 10% EtOAc in hexanes) afforded 33.5 mg (54%) of **10b** as a colorless thick oil in >95:5 *E:Z*. The stereochemistry was assigned by analogy to that of **10i**. R_f = 0.44 (15% EtOAc/hexanes). ^{1}H NMR ($CDCl_3$, 600 MHz) δ: 7.56–7.58 (m, 2H), 7.49 (d, J = 8.1 Hz, 2H), 7.37 – 7.40 (m, 3H), 7.34 (d, J = 8.8 Hz, 2H), 7.31 (d, J = 8.8 Hz, 2H), 7.15 (d, J = 8.1 Hz, 2H), 6.07 (s, 1H), 4.55 (t, J = 6.8 Hz,1H), 2.88 (d, J = 6.8 Hz, 2H), 1.63 (br s, 1H), 0.45 (s, 3H), 0.44 (s, 3H); $^{13}C\{^1H\}$NMR (151 MHz, $CDCl_3$) δ 153.1, 147.7, 141.5, 139.0, 133.9, 133.7, 132.1, 129.6 (q, $^2J_{CF}$ = 31 Hz), 129.3, 128.6, 128.2, 127.7, 125.8, 125.2 (q, $^3J_{CF}$ = 3.4 Hz), 124.1 (q, $^1J_{CF}$ = 272 Hz), 71.7, 44.5, –0.8, –1.1 ppm $^{19}F\{^1H\}$NMR (565 MHz, $CDCl_3$) δ: –62.5 ppm. HRMS (DART) *m/z* calcd for $C_{25}H_{25}ClF_3OSi$ [M+H]$^+$: 461.1315; Found [M + H]$^+$: 461.1325.

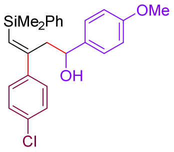

(*E*)-3-(4-chlorophenyl)-4-(dimethyl(phenyl)silyl)-1-(4-methoxyphenyl)but-3-en-1-ol (10c): According to the general procedure using $Mg(OTf)_2$ as Lewis acid and 1-chloro-4-iodobenzene, purification by silica gel chromatography (eluent: 0 – 10% EtOAc in hexanes) afforded 37 mg (64%) of **10c** as a colorless thick oil in >95:5 *E:Z*. The stereochemistry was assigned by analogy to that of **10i**. R_f = 0.66 (15% EtOAc/hexanes). ^{1}H NMR ($CDCl_3$, 600 MHz) δ: 7.55–7.57 (m, 2H), 7.35–7.39 (m, 3H), 7.33 (d, J = 8.6 Hz, 2H), 7.30 (d, J = 8.6 Hz, 2H), 6.99 (d, J = 8.6 Hz, 2H), 6.78 (d, J = 8.6 Hz, 2H), 6.01 (s, 1H), 4.47 (dd, J = 9.0 Hz, 4.5 Hz, 1H), 3.78 (s, 3H), 2.93 (dd, J = 14 Hz, 9.0 Hz, 1H), 2.86 (dd, J = 14 Hz, 4.5 Hz, 1H), 1.55 (s, 1H), 0.46 (s, 3H), 0.45 (s, 3H). $^{13}C\{^1H\}$ NMR (151 MHz, $CDCl_3$): δ 159.0, 153.8, 141.9, 139.2, 136.0, 133.9, 133.4, 131.2, 129.1, 128.4, 128.0, 127.8, 126.8, 113.7, 72.0, 55.2, 44.5, –0.7, –0.9 ppm. HRMS (DART) *m/z* calcd for $C_{25}H_{28}ClO_2Si$ [M+H]$^+$: 423.1547; Found [M + H]$^+$: 423.1541.

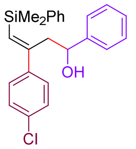

(*E*)-3-(4-chlorophenyl)-4-(dimethyl(phenyl)silyl)-1-phenylbut-3-en-1-ol (10d): According to the general procedure using Mg(OTf)2 as Lewis acid and 1-chloro-4-iodobenzene, purification by silica gel chromatography (eluent: 0 – 10% EtOAc in hexanes) afforded 30 mg (57%) of **10d** as a colorless thick oil in >95:5 *E:Z*. The stereochemistry was assigned by analogy to that of **10i**. R_f = 0.44 (15% EtOAc/hexanes). ^{1}H NMR ($CDCl_3$, 600 MHz) δ: 7.56–7.58 (m, 2H), 7.37–7.38 (m, 3H), 7.35 (d, *J* = 8.6 Hz, 2H), 7.31 (d, *J* = 8.6 Hz, 2H), 7.20 – 7.29 (m, 3H), 7.09 (d, *J* = 7.0 Hz, 2H), 6.04 (s, 1H), 4.52 (dd, *J* = 8.8 Hz, 4.7 Hz, 1H), 2.93 (dd, *J* = 14 Hz, 8.8 Hz, 1H), 2.90 (dd, *J* = 14 Hz, 4.7 Hz, 1H), 1.58 (s, 1H), 0.46 (s, 3H), 0.45 (s, 3H). ^{13}C{^{1}H}NMR (151 MHz, $CDCl_3$): δ 153.7, 143.9, 141.8, 139.2, 133.8, 133.5, 131.4, 129.2, 128.5, 128.3, 128.0, 127.8, 127.5, 125.6, 72.3, 44.6, –0.7, –0.9 ppm. HRMS (DART) *m/z* calcd for $C_{24}H_{26}ClOSi$ [M+H]$^+$:393.1441 ; Found [M + H]$^+$: 393.1446.

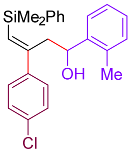

(*E*)-3-(4-chlorophenyl)-4-(dimethyl(phenyl)silyl)-1-(*o*-tolyl)but-3-en-1-ol (10e): According to the general procedure using Mg(OTf)2 as Lewis acid and 1-chloro-4-iodobenzene at 80 °C maintained using an oil bath, purification by silica gel chromatography (eluent: 0 – 10% EtOAc in hexanes) afforded 35 mg (64%) of **10e** as a colorless thick oil in >95:5 *E:Z*. The stereochemistry was assigned by analogy to that of **10i**. R_f = 0.55 (15% EtOAc/hexanes). ^{1}H NMR ($CDCl_3$, 600 MHz) δ: 7.59–7.60 (m, 2H), 7.37–7.38 (m, 3H), 7.35 (d, *J* = 8.6 Hz, 2H), 7.30 (d, *J* = 8.6 Hz, 2H), 7.09 –7.19 (m, 3H), 7.04 (d, *J* = 7.3 Hz, 1H), 6.07 (s, 1H), 4.69 (dd, *J* = 9.8 Hz, 3.5 Hz, 1H), 2.87 (dd, *J* = 14 Hz, 3.5 Hz, 1H), 2.82 (dd, *J* = 14 Hz, 9.8 Hz, 1H), 2.13 (s, 3H), 1.52 (s, 1H), 0.50 (s, 3H), 0.49 (s, 3H). ^{13}C{^{1}H}NMR (151 MHz, $CDCl_3$): δ 154.0, 142.0, 141.7, 139.2, 134.0, 133.9, 133.5, 131.7, 130.2, 129.2, 128.5, 128.1, 127.7, 127.2, 126.3, 125.2, 68.2, 43.4, 19.0, –0.7, –0.9 ppm. HRMS (DART) *m/z* calcd for $C_{25}H_{26}ClSi$ [M–OH]$^+$: 389.1492; Found [M–OH]$^+$: 389.1522.

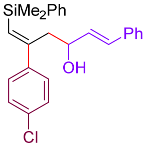

(1*E*,5*E*)-5-(4-chlorophenyl)-6-(dimethyl(phenyl)silyl)-1-phenylhexa-1,5-dien-3-ol (10f): According to the general procedure using Mg(OTf)2 as Lewis acid and 1-chloro-4-iodobenzene, purification by silica gel chromatography (eluent: 0 – 15% EtOAc in hexanes) afforded 33.8 mg (60%) of **10f** as a colorless thick oil in >95:5 *E:Z*. The stereochemistry was assigned by analogy to that of **10i**. R_f = 0.35 (20% EtOAc/hexanes). ^{1}H NMR ($CDCl_3$, 600 MHz) δ: 7.56–7.85 (m, 2H), 7.35 – 7.40 (m, 3H), 7.31 (d, *J* = 8.6 Hz, 2H), 7.25 – 7.28 (m, 4H), 7.19 – 7.22 (m, 3H), 6.26 (d, *J* = 16 Hz, 1H), 6.07 (s, 1H), 5.92 (dd, *J* = 16 Hz, 6.5 Hz, 1H), 4.09 – 4.16 (m, 1H), 2.86 (dd, *J* = 14 Hz, 8.0 Hz, 1H), 2.83 (dd, *J* = 14 Hz, 5.9 Hz, 1H), 1.31 (d, *J* = 3.5 Hz, 1H), 0.49 (s, 3H), 0.48 (s, 3H). ^{13}C{^{1}H}NMR (151 MHz, $CDCl_3$): δ 153.6, 142.0, 139.2, 136.5, 133.9, 133.4, 131.2, 131.0, 130.1, 129.2, 128.48, 128.46, 128.0, 127.8, 127.6, 126.4, 71.2, 42.5, –0.8, –0.9 ppm. HRMS (DART) *m/z* calcd for $C_{26}H_{28}ClOSi$ [M+H]$^+$: 419.1598; Found [M+H]$^+$: 419.1628.

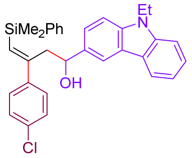

(*E*)-3-(4-chlorophenyl)-4-(dimethyl(phenyl)silyl)-1-(9-ethyl-9H-carbazol-3-yl)but-3-en-1-ol (10g): According to the general procedure using $Mg(OTf)_2$ as Lewis acid and 1-chloro-4-iodobenzene, purification by silica gel chromatography (eluent: 0 – 20% EtOAc in hexanes) afforded 63.1 mg (92%) of **10g** as a white foam oil in >95:5 *E:Z*. The stereochemistry was assigned by analogy to that of **10i**. R_f = 0.27 (20% EtOAc/hexanes). ^{1}H NMR ($CDCl_3$, 600 MHz) δ: 8.05 (d, *J* = 7.8 Hz 1H), 7.78 (s, 1H), 7.58–7.61 (m, 2H), 7.47 (t, *J* = 7.4 Hz, 1H) , 7.34 – 7.40 (m, 6H), 7.27 – 7.32 (m, 5H), 6.02 (s, 1H), 4.72 (dd, *J* = 9.1 Hz, 4.7 Hz, 1H), 4.34 (q, *J* = 7.3 Hz, 2H), 3.08 (dd, *J* = 14 Hz, 9.1 Hz, 1H), 3.00 (dd, *J* = 14 Hz, 4.7 Hz, 1H), 1.67 (br s, 1H), 1.42 (t, *J* = 7.3 Hz, 3H), 0.47 (s, 3H), 0.46 (s, 3H). ^{13}C NMR (151 MHz, $CDCl_3$): δ 154.2, 142.1, 140.2, 139.5, 139.3, 134.4, 133.9, 133.3, 131.0, 129.1, 128.4, 128.0, 127.9, 125.6, 123.5, 122.8, 122.7, 120.4, 118.7, 117.6, 108.5, 108.2, 73.0. 45.0, 37.5, 13.8, –0.7, –0.9 ppm. HRMS (DART) *m/z* calcd for $C_{32}H_{31}ClNSi$ $[M–OH]^+$: 492.1914; Found $[M–OH]^+$: 492.1916.

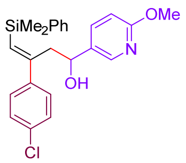

(*E*)-3-(4-chlorophenyl)-4-(dimethyl(phenyl)silyl)-1-(6-methoxypyridin-3-yl)but-3-en-1-ol (10h): According to the general procedure using $Mg(OTf)_2$ as Lewis acid and 1-chloro-4-iodobenzene, purification by silica gel chromatography (eluent: 0 – 20% EtOAc in hexanes) afforded 31 mg (54%) of **10h** as a colorless thick oil in >95:5 *E:Z*. The stereochemistry was assigned by analogy to that of **10i**. R_f = 0.22 (15% EtOAc/hexanes). ^{1}H NMR ($CDCl_3$, 600 MHz) δ: 7.89 (s, 1H), 7.54 – 7.59 (m, 2H), 7.36 – 7.42 (m, 3H), 7.30 (app. s, 4H), 6.71 (d, *J* = 8.8 Hz, 1H), 6.07 (s, 1H), 4.46 – 4.51 (m, 1H), 4.00 (s, 3H), 2.93 (dd, *J* = 14 Hz, 9.0 Hz, 1H), 2.6 (dd, *J* = 14 Hz, 4.7 Hz, 1H), 1.62 (s, 1H), 0.46 (s, 3H), 0.45 (s, 3H); ^{13}C{^{1}H}NMR (151 MHz, $CDCl_3$): δ 163.7, 153.2, 144.3, 141.6, 139.0, 136.4, 133.8, 133.6, 131.8, 129.6, 129.3, 128.6, 128.1, 127.7, 110.7, 70.0, 53.4, 44.2, –0.8, –1.0 ppm. HRMS (DART) *m/z* calcd for $C_{24}H_{27}ClNO_2Si$ $[M+H]^+$: 424.1500; Found $[M+H]^+$: 492.1509.

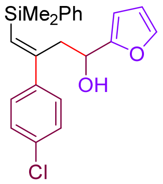

(*E*)-3-(4-chlorophenyl)-4-(dimethyl(phenyl)silyl)-1-(furan-2-yl)but-3-en-1-ol (10i): According to the general procedure using 11.2 µL (0.135 mmol) of 2-fufural, $Mg(OTf)_2$ as Lewis acid, and 1-chloro-4-iodobenzene, purification by silica gel chromatography (eluent: 0 – 15% EtOAc in hexanes) afforded 50.0 mg (97%) of **10i** as a colorless thick oil in >95:5 *E:Z*. The stereochemistry was assigned by NOE analysis. R_f = 0.24 (15% EtOAc/hexanes). ^{1}HNMR ($CDCl_3$, 600 MHz) δ: 7.57 – 7.62 (m, 2H), 7.35 – 7.39 (m, 3H), 7.35 (d, *J* = 8.6 Hz, 2H), 7.27 – 7.30 (m, 3H), 6.23 (dd, *J* = 3.2 Hz, 1.9 Hz, 1H), 6.04 (s, 1H), 5.99 (d, *J* = 3.2 Hz, 1H), 4.50 – 4.55 (m, 1H), 3.01 – 3.10 (m, 2H), 1.49 (d, *J* = 5.2 Hz, 1H), 0.490 (s, 3H), 0.487 (s, 3H); ^{13}C{^{1}H}NMR (151 MHz, $CDCl_3$): δ 155.6, 153.1, 141.9, 141.6, 139.1, 133.8, 133.4, 131.3, 129.2, 128.4, 128.0, 127.7, 110.0, 105.9, 66.2, 40.8, –0.8, –1.0 ppm. HRMS (DART) *m/z* calcd for $C_{22}H_{23}ClO_2Si$ $[M]^+$: 382.1156; Found $[M]^+$: 382.1155.

NOESY (D6-acetone, 600 MHz):

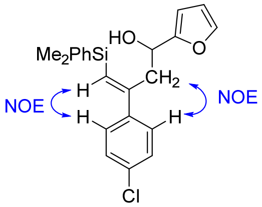

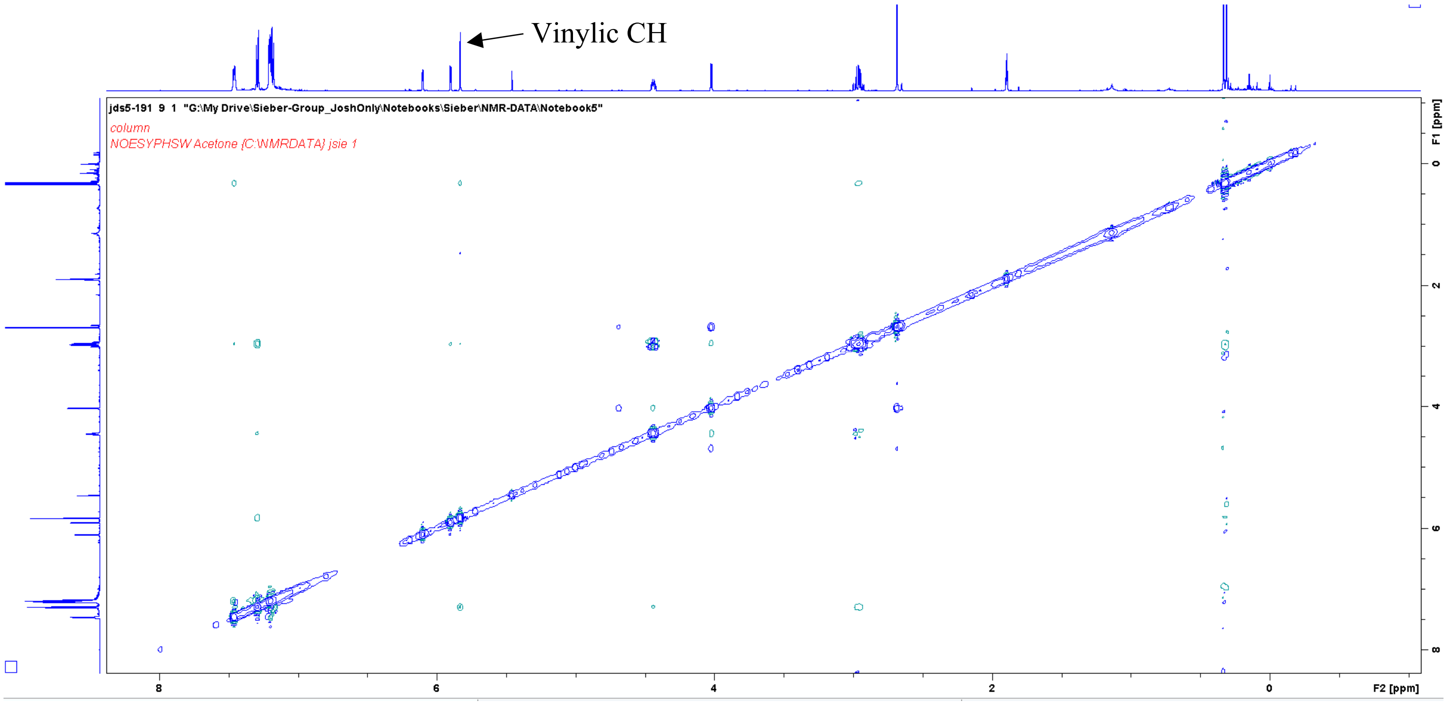
Vinylic CH
jds5-191 9 1 "G:\My Drive\Sieber-Group_JoshOnly\Notebooks\Sieber\NMR-DATA\Notebook5"
column
NOESYPHSW Acetone {C:\NMRDATA} jsie 1
F1 [ppm]
F2 [ppm]
8
6
4
2
0

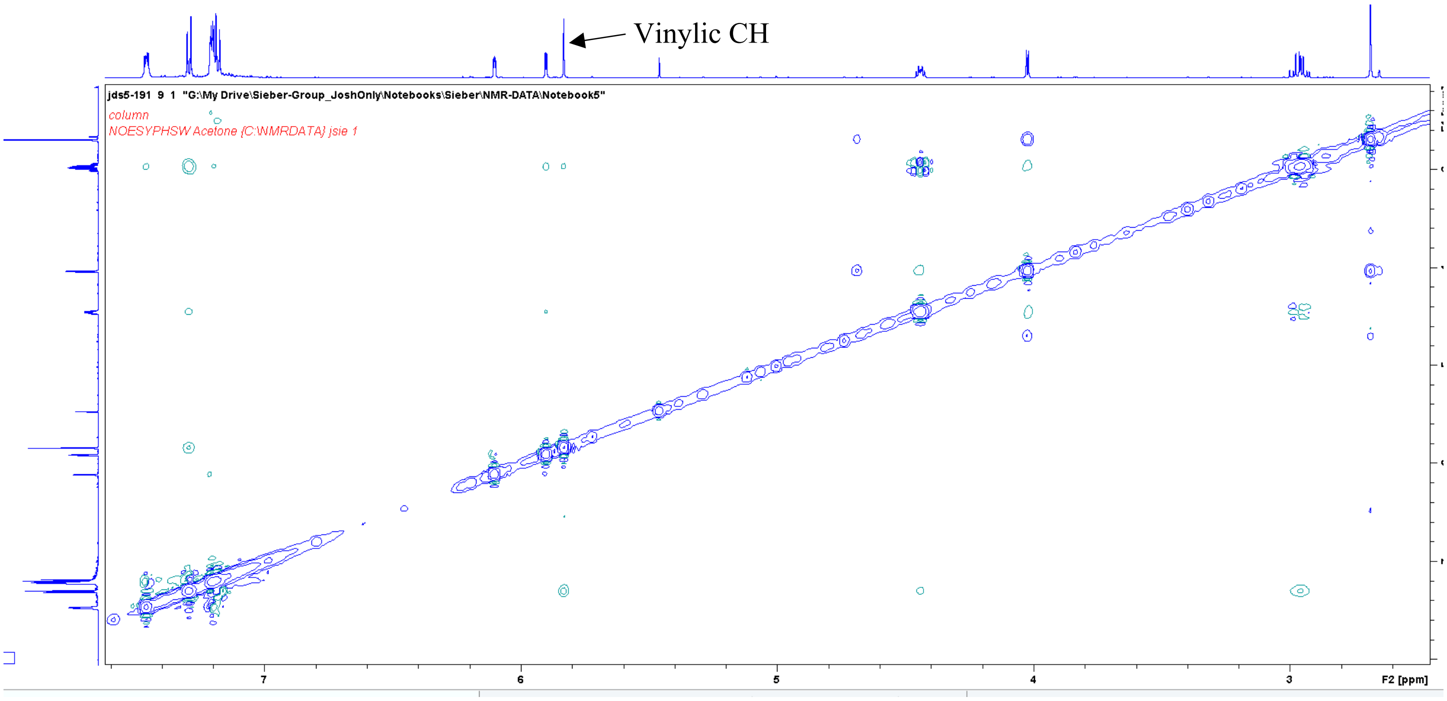
Vinylic CH
jds5-191 9 1 "G:\My Drive\Sieber-Group_JoshOnly\Notebooks\Sieber\NMR-DATA\Notebook5"
column
NOESYPHSW Acetone {C:\NMRDATA} jsie 1
7
6
5
4
3
F2 [ppm]

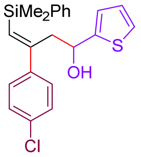

(*E*)-3-(4-chlorophenyl)-4-(dimethyl(phenyl)silyl)-1-(thiophen-2-yl)but-3-en-1-ol (10j): According to the general procedure using $Mg(OTf)_2$ as Lewis acid and 1-chloro-4-iodobenzene, purification by silica gel chromatography (eluent: 0 – 10% EtOAc in hexanes) afforded 34 mg (62%) of **10j** as a colorless thick oil in >95:5 *E:Z*. The stereochemistry was assigned by analogy to that of **10i**. R_f = 0.44 (15% EtOAc/hexanes). ^{1}H NMR ($CDCl_3$, 600 MHz) δ: 7.57 –7.59 (m, 2H), 7.36-7.37 (m, 3H), 7.34 (d, *J* = 8.7 Hz, 2H), 7.30 (d, *J* = 8.7 Hz, 2H), 7.19 (dd, *J* = 5.0 Hz, 1.0 Hz, 1H), 6.89 (dd, *J* = 5.0 Hz, 3.5 Hz, 1H), 6.74 (d, *J* = 3.5 Hz, 1H), 6.07 (s, 1H), 4.77 (dd, *J* = 9.2 Hz, 4.6 Hz, 1H), 3.06 (dd, *J* = 14 Hz, 9.2 Hz, 1H), 3.02 (dd, *J* = 14 Hz, 4.6 Hz, 1H), 1.67 (br s, 1H), 0.484 (s, 3H), 0.480 (s, 3H); ^{13}C{^{1}H}NMR (151 MHz, $CDCl_3$): δ 153.1, 147.6, 141.6, 139.1, 133.8, 133.5, 131.7, 129.2, 128.5, 128.0, 127.7, 126.5, 124.4, 123.4, 68.4, 44.5, –0.8, –1.0 ppm. HRMS (DART) *m/z* calcd for $C_{22}H_{22}ClSSi$ $[M-OH]^+$: 381.0900; Found $[M-OH]^+$: 381.0930.

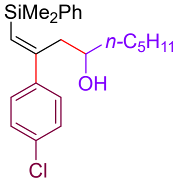

(*E*)-2-(4-chlorophenyl)-1-(dimethyl(phenyl)silyl)non-1-en-4-ol (10k): According to the general procedure using 16.6 µL (0.135 mmol) of *n*-hexanal, $Mg(OTf)_2$ as Lewis acid, and 1-chloro-4-iodobenzene, purification by silica gel chromatography (eluent: 0 – 15% EtOAc in hexanes) afforded 36.1 mg (67%) of **10k** as a colorless oil in >95:5 *E:Z*. The stereochemistry was assigned by analogy to that of **10i**. R_f = 0.31 (15% EtOAc/hexanes). ^{1}HNMR ($CDCl_3$, 600 MHz) δ: 7.56 – 7.61 (m, 2H), 7.35 – 7.40 (m, 3H), 7.31 (d, *J* = 8.7 Hz, 2H), 7.28 (d, *J* = 8.7 Hz, 2H), 6.00 (s, 1H), 3.38 – 3.46 (m, 1H), 2.66 (dd, *J* = 14 Hz, 4.3 Hz, 1H), 2.60 (dd, *J* = 14 Hz, 8.9 Hz, 1H), 1.10 – 1.34 (m, 8H), 1.06 (br s, 1H), 0.85 (t, *J* = 7.2 Hz, 3H), 0.48 (s, 3H), 0.47 (s, 3H); ^{13}C{^{1}H}NMR (151 MHz, $CDCl_3$): δ 154.5, 142.0, 139.3, 133.8, 133.4, 130.5, 129.1, 128.4, 128.0, 127.7, 70.0, 42.5, 37.1, 31.7, 25.2, 22.5, 14.0, –0.8 ppm. HRMS (DART) *m/z* calcd for $C_{23}H_{31}ClOSi$ $[M]^+$: 386.1833; Found $[M]^+$: 386.1843.

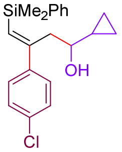

(*E*)-3-(4-chlorophenyl)-1-cyclopropyl-4-(dimethyl(phenyl)silyl)but-3-en-1-ol (10l): According to the general procedure using $Mg(OTf)_2$ as Lewis acid and 1-chloro-4-iodobenzene, purification by silica gel chromatography (eluent: 0 – 10% EtOAc in hexanes) afforded 31 mg (64%) of **10l** as a colorless thick oil in >95:5 *E:Z*. The stereochemistry was assigned by analogy to that of **10i**. R_f = 0.33 (15% EtOAc/hexanes). ^{1}H NMR ($CDCl_3$, 600 MHz) δ: 7.58 – 7.62 (m, 2H), 7.35 – 7.39 (m, 3H), 7.31 (d, *J* =8.7 Hz, 2H), 7.27 (d, *J* = 8.7 Hz, 2H), 6.00 (s, 1H), 2.85 (dd, *J* = 14 Hz, 5.0 Hz, 1H), 2.74 – 2.84 (m, 2H), 1.21 (d, *J* = 3.1 Hz, 1H), 0.61 – 0.68 (m, 1H), 0.50 (s, 3H), 0.49 (s, 3H), 0.29 – 0.39 (m, 2H), 0.061 – 0.12 (m, 1H), –0.058 – 0.00 (m, 1H); ^{13}C{^{1}H}NMR (151 MHz, $CDCl_3$): δ 154.1, 142.1, 139.3, 133.8, 133.3, 130.4, 129.1, 128.4, 127.9, 127.6, 74.4, 42.3, 17.5, 2.6, 2.5, –0.7, –0.8 ppm. HRMS (DART) *m/z* calcd for $C_{21}H_{26}ClOSi$ $[M+H]^+$: 357.1441; Found $[M+H]^+$: 357.1438.

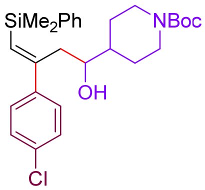

***tert*-butyl (*E*)-4-(3-(4-chlorophenyl)-4-(dimethyl(phenyl)silyl)-1-hydroxybut-3-en-1-yl)piperidine-1-carboxylate (10m)**: According to the general procedure using $Mg(OTf)_2$ as Lewis acid and 1-chloro-4-iodobenzene. The aldehyde (28.3 mg, 0.133 mmol) was added as a 123 mg/mL stock solution in acetonitrile (0.23 mL). Purification by silica gel chromatography (eluent: 0 – 30% EtOAc in hexanes) afforded 41.8 mg (63%) of **10m** as an amorphous white solid in >95:5 *E:Z*. The stereochemistry was assigned by analogy to that of **10i**. R_f = 0.19 (20% EtOAc/hexanes). ^{1}HNMR ($CDCl_3$, 600 MHz) δ: 7.56 – 7.60 (m, 2H), 7.36 – 7.40 (m, 3H), 7.28 (app. s, 4H), 6.05 (s, 1H), 3.98 – 4.19 (m, 2H), 3.18 – 3.26 (m, 1H), 2.69 (dd, *J* = 14 Hz, 2.0 Hz, 1H), 2.49 – 2.61 (m, 3H), 1.54 – 1.59 (m, 1H), 1.45 – 1.48 (m, 1H), 1.44 (s, 9H), 1.25 – 1.31 (m, 1H), 1.00 – 1.17 (m, 3H), 0.48 (s, 3H), 0.47 (s, 3H); $^{13}C\{^1H\}$NMR (151 MHz, $CDCl_3$): δ 154.7, 154.3, 141.6, 139.2, 133.8, 133.5, 131.2, 129.2, 128.5, 128.0, 127.8, 127.6, 79.3, 72.6, 42.1, 39.1, 28.4, 39.1, 28.4, 27.9, –0.8, –0.8 ppm. HRMS (DART) *m/z* calcd for $C_{28}H_{39}ClNO_3Si$ $[M+H]^+$: 500.2388; Found $[M + H]^+$: 500.2370.

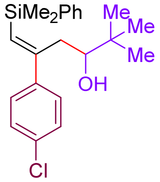

(*E*)-5-(4-chlorophenyl)-6-(dimethyl(phenyl)silyl)-2,2-dimethylhex-5-en-3-ol (10n): According to the general procedure using $Mg(OTf)_2$ as Lewis acid and 1-chloro-4-iodobenzene, purification by preparatory TLC (silica gel, 7% EtOAc in hexanes) afforded 26.9 mg (54%) of **10n** as a colorless thick oil in >95:5 *E:Z*. The stereochemistry was assigned by analogy to that of **10i**. R_f = 0.26 (7% EtOAc/hexanes). ^{1}H NMR ($CDCl_3$, 600 MHz) δ: 7.55 – 7.62 (m, 2H), 7.36 – 7.40 (m, 3H), 7.28 – 7.32 (m, 4H), 6.03 (s, 1H), 3.01 – 3.08 (m, 1H), 2.75 (dd, *J* = 14 Hz, 1.3 Hz, 1H), 2.47 (dd, *J* = 14 Hz, 11 Hz, 1H), 1.02 (d, *J* = 4.4 Hz, 1H), 0.78 (s, 9H), 0.49 (s, 3H), 0.48 (s, 3H); $^{13}C\{^1H\}$NMR (151 MHz, $CDCl_3$): δ 155.2, 141.8, 139.3, 133.8, 133.4, 131.0, 129.2, 128.4, 128.0, 127.7, 76.5, 36.7, 34.8, 25.6, –0.7, –0.8 ppm. HRMS (DART) *m/z* calcd for $C_{22}H_{30}ClOSi$ $[M + H]^+$: 373.1754; Found $[M + H]^+$: 373.1757.

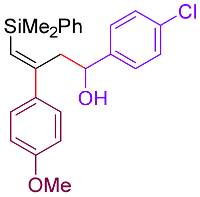

(*E*)-1-(4-chlorophenyl)-4-(dimethyl(phenyl)silyl)-3-(4-methoxyphenyl)but-3-en-1-ol (10o): According to the general procedure using $Mg(OTf)_2$ as Lewis acid and 1-iodo-4-methoxybenzene, purification by silica gel chromatography (eluent: 0 – 20% EtOAc in hexanes) afforded 53.3 mg (93%) of **10o** as a colorless thick oil in >95:5 *E:Z*. The stereochemistry was assigned by analogy to that of **10i**. R_f = 0.18 (20% EtOAc/hexanes). ^{1}HNMR ($CDCl_3$, 600 MHz) δ: 7.55 – 7.99 (m, 2H), 7.35 – 7.40 (m, 5H), 7.21 (d, *J* = 8.4 Hz, 2H), 7.02 (d, *J* = 8.4 Hz, 2H), 6.89 (d, *J* = 8.8 Hz, 2H), 6.02 (s, 1H), 4.52 – 4.57 (m, 1H), 3.83 (s, 3H), 2.88 (dd, *J* = 14 Hz, 4.7 Hz, 1H), 2.85 (dd, *J* = 14 Hz, 8.9 Hz, 1H), 1.66 (d, *J* = 3.1 Hz, 1H), 0.45 (s, 3H), 0.44 (s, 3H); $^{13}C\{^1H\}$NMR (151 MHz, $CDCl_3$): δ 159.4, 153.8, 142.5, 139.4, 135.3, 133.9, 132.9, 129.2, 129.1, 128.3, 128.0, 127.6, 127.0, 113.8, 71.6, 55.3, 44.8, –0.6, –0.8 ppm. HRMS (DART) *m/z* calcd for $C_{25}H_{28}ClO_2Si$ $[M+H]^+$: 423.1547; Found $[M + H]^+$: 423.1576.

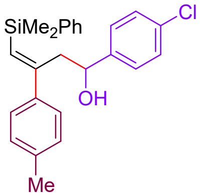

(*E*)-1-(4 chlorophenyl)-4-(dimethyl(phenyl)silyl)-3-(p-tolyl)but-3-en-1-ol (10p): According to the general procedure using $Mg(OTf)_2$ as Lewis acid and 4-iodotoluene as the aryl halide. Purification by silica gel chromatography (eluent: 0 – 10% EtOAc in hexanes) afforded 37.5 mg (68%) of **10p** as a colorless thick oil in >95:5 *E:Z*. The stereochemistry was assigned by analogy to that of **10i**. R_f = 0.19 (10% EtOAc/hexanes). ^{1}HNMR ($CDCl_3$, 600 MHz) δ 7.55 – 7.60 (m, 2H), 7.36 – 7.42 (m, 3H), 7.34 (d, *J* = 8.1 Hz, 2H), 7.22 (d, *J* = 8.4 Hz, 2H), 7.18 (d, *J* = 8.1 Hz, 2H), 7.02 (d, *J* = 8.4 Hz, 2H), 6.07 (s, 1H), 4.55 (dd, *J* = 8.8 Hz, 4.7 Hz, 1H), 2.90 (dd, *J* = 14, 4.7 Hz, 1H), 2.88 (dd,

J = 14, 8.8 Hz, 1H), 2.38 (s, 3H), 1.71 (br s, 1H), 0.46 (s, 3H), 0.45 (s, 3H); ^{13}C NMR (151 MHz, $CDCl_3$): 154.4, 142.5, 140.1, 139.4, 137.8, 133.9, 132.9, 130.2, 129.2, 129.1, 128.3, 128.0, 126.9, 126.3, 71.6, 44.8, 21.1, –0.7, –0.8. HRMS (DART) *m/z* calcd for $C_{25}H_{28}ClOSi$ [M+H]$^+$: 407.1598; Found [M + H]$^+$: 407.1576.

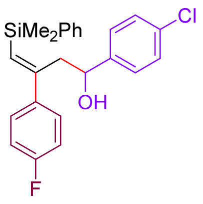

(*E*)-1-(4-chlorophenyl)-4-(dimethyl(phenyl)silyl)-3-(4-fluorophenyl)but-3-en-1-ol (10q): According to the general procedure using $Mg(OTf)_2$ as Lewis acid and 1-fluoro-4-iodobenzene, purification by silica gel chromatography (eluent: 0 – 15% EtOAc in hexanes) afforded 35.1 mg (63%) of **10q** as a colorless thick oil in >95:5 *E:Z*. The stereochemistry was assigned by analogy to that of **10i**. R_f = 0.25 (15% EtOAc/hexanes). ^{1}HNMR ($CDCl_3$, 600 MHz) δ: 7.55 – 7.59 (m, 2H), 7.36 – 7.41 (m, 5H), 7.21 (d, J = 8.5 Hz, 2H), 7.04 (t, J = 8.7 Hz, 2H), 6.99 (d, J = 8.5 Hz, 2H), 6.02 (s, 1H), 4.47 – 4.52 (m, 1H), 2.83 – 2.91 (m, 2H), 1.59 (br s, 1H), 0.46 (s, 3H), 0.44 (s, 3H); ^{13}C{^{1}H}NMR (151 MHz, $CDCl_3$): δ 162.4 (d, $^1J_{CF}$ = 248 Hz), 153.4, 142.3, 139.18, 139.15, 133.9, 133.0, 131.2, 129.2, 128.4, 128.1, 128.0 (d, $^3J_{CF}$ = 8.0 Hz), 126.9, 115.3 (d, $^2J_{CF}$ = 21 Hz), 71.6, 44.8, –0.8, –1.0 ppm. ^{19}F{^{1}H}NMR (564 MHz, $CDCl_3$): δ –114.4 ppm. HRMS (DART) *m/z* calcd for $C_{24}H_{25}ClFOSi$ [M+H]$^+$: 411.1347; Found [M + H]$^+$: 411.1322.

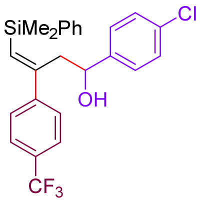

(*E*)-1-(4-chlorophenyl)-4-(dimethyl(phenyl)silyl)-3-(4-(trifluoromethyl)phenyl)but-3-en-1-ol (10r): According to the general procedure using $Mg(OTf)_2$ as Lewis acid and 1-iodo-4-(trifluoromethyl)benzene as the aryl halide. Purification by silica gel chromatography (eluent: 0 – 10% EtOAc in hexanes) afforded 41 mg (66%) of **10r** as a colorless thick oil in >95:5 *E:Z*. The stereochemistry was assigned by analogy to that of **10i**. R_f = 0.42 (10% EtOAc/hexanes). ^{1}H NMR (600 MHz, $CDCl_3$) δ 7.59 (d, J = 8.1 Hz, 2H), 7.55 – 7.58 (m, 2H), 7.48 (d, J = 8.1 Hz, 2H), 7.37 – 7.42 (m, 3H), 7.20 (d, J = 8.4 Hz, 2H), 6.96 (d, J = 8.4 Hz, 2H), 6.10 (s, 1H), 4.47 (dd, J = 8.6 Hz, 5.0 Hz, 1H), 2.92 (dd, J = 14 Hz, 8.6 Hz, 1H), 2.89 (dd, J = 14 Hz, 5.0 Hz, 1H), 1.56 (br s, 1H), 0.47 (s, 3H), 0.46 (s, 3H); ^{13}C NMR (151 MHz, $CDCl_3$): δ 153.3, 146.9, 142.2, 138.9, 133.9, 133.4, 133.2, 129.6 (q, $^2J_{CF}$ = 33 Hz), 129.3, 128.4, 128.1, 126.9, 126.8, 125.3 (q, $^3J_{CF}$ = 3.8 Hz), 124.1 (q, $^1J_{CF}$ = 272 Hz), 71.6, 44.5, –0.9, –1.1 ppm. ^{19}F{^{1}H}NMR (565 MHz, $CDCl_3$) δ: –62.5 ppm. HRMS (DART) *m/z* calcd for $C_{25}H_{25}ClF_3OSi$ [M+H]$^+$: 461.1315; Found [M + H]$^+$: 461.1319.

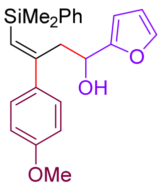

(*E*)-4-(dimethyl(phenyl)silyl)-1-(furan-2-yl)-3-(4-methoxyphenyl)but-3-en-1-ol (10s): According to the general procedure using 12.9 µL (0.156 mmol) of 2-fufural, $Mg(OTf)_2$ as Lewis acid and 1-iodo-4-methoxybenzene, purification by silica gel chromatography (eluent: 0 – 20% EtOAc in hexanes) afforded 56.5 mg (96%) of **10s** as a colorless thick oil in >95:5 *E:Z*. The stereochemistry was assigned by analogy to that of **10i**. R_f = 0.24 (20% EtOAc/hexanes). ^{1}HNMR ($CDCl_3$, 600 MHz) δ: 7.57 – 7.62 (m, 2H), 7.33 – 7.38 (m, 5H), 7.30 (dd, J = 1.7 Hz, 0.7 Hz, 1H), 6.85 (d, J = 8.8 Hz, 2H), 6.25 (dd, J = 3.2 Hz, 1.7 Hz, 1H), 6.03 (d, J = 3.2 Hz, 1H), 6.02 (s, 1H), 4.55 – 4.62 (m, 1H), 3.81 (s, 3H), 3.01 – 3.10 (m, 2H), 1.56 (d, J = 4.9 Hz, 1H), 0.484 (s, 3H), 0.482 (s, 3H); ^{13}C{^{1}H}NMR (151 MHz, $CDCl_3$): δ 159.3, 155.9, 153.5, 141.8, 139.5, 135.3, 133.8, 129.0, 128.9, 127.9, 127.5, 113.7, 110.0, 105.8, 66.2, 55.3, 40.9, –0.7, –0.8 ppm. HRMS (DART) *m/z* calcd for $C_{23}H_{26}O_3Si$ [M]$^+$: 378.1651; Found [M]$^+$: 378.1642.

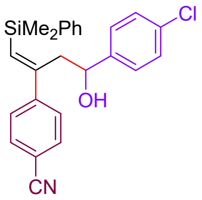

(*E*)-4-(4-(4-chlorophenyl)-1-(dimethyl(phenyl)silyl)-4-hydroxybut-1-en-2-yl)benzonitrile (10t): According to the general procedure using Mg(OTf)$_2$ as Lewis acid and 4-bromobenzonitrile as the aryl halide. Purification by silica gel chromatography (eluent: 0 – 10% EtOAc in hexanes) afforded 25 mg (44%) of **10t** as a colorless thick oil in >95:5 *E:Z*. The stereochemistry was assigned by analogy to that of **10i**. R$_f$ = 0.14 (10% EtOAc/hexanes). ^{1}H NMR (600 MHz, CDCl$_3$) δ 7.62 (d, *J* = 8.4 Hz, 2H), 7.54 – 7.58 (m, 2H), 7.47 (m, 2H), 7.37 – 7.44 (m, 3H), 7.21 (d, *J* = 8.5 Hz, 2H), 6.94 (d, *J* = 8.5 Hz, 2H), 6.12 (s, 1H), 4.44 (dd, *J* = 8.5 Hz, 4.8 Hz, 1H), 2.89 (dd, *J* = 14 Hz, 8.5 Hz, 1H), 2.87 (dd, *J* = 14 Hz, 4.8 Hz, 1H), 1.52 (br s, 1H), 0.47 (s, 3H), 0.46 (s, 3H); ^{13}C{^{1}H}NMR (151 MHz, CDCl$_3$): δ 152.9, 148.0, 142.0, 138.6, 134.4, 133.8, 133.3, 129.4, 128.5, 128.2, 127.1, 126.9, 118.7, 111.2, 71.8, 44.1, –1.0, –1.2 ppm. HRMS (DART) *m/z* calcd for $C_{25}H_{24}ClNSiO$ [M]$^+$: 417.1316; Found [M]$^+$: 417.1345.

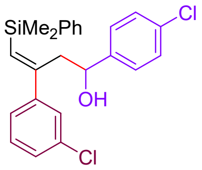

(*E*)-3-(3-chlorophenyl)-1-(4-chlorophenyl)-4-(dimethyl(phenyl)silyl)but-3-en-1-ol (10u): According to the general procedure using Mg(OTf)$_2$ as Lewis acid and 1-chloro-3-Iodobenzene as the aryl halide. Purification by silica gel chromatography (eluent: 0 – 10% EtOAc in hexanes) afforded 43 mg (74%) of **10u** as a colorless thick oil in >95:5 *E:Z*. The stereochemistry was assigned by analogy to that of **10i**. R$_f$ = 0.28 (10% EtOAc/hexanes). ^{1}H NMR (600 MHz, CDCl$_3$) δ 7.54 – 7.60 (m, 2H), 7.35 – 7.42 (m, 4H), 7.26 – 7.29 (m, 3H), 7.21 (d, *J* = 8.4 Hz, 2H), 6.97 (d, *J* = 8.4 Hz, 2H), 6.07 (s, 1H), 4.49 (dd, *J* = 9.2 Hz, 5.0 Hz, 1H), 2.87 (dd, *J* = 14 Hz, 9.2 Hz, 1H), 2.85 (dd, *J* = 14 Hz, 5.0 Hz, 1H), 1.56 (br s, 1H), 0.46 (s, 3H), 0.45 (s, 3H); ^{13}C{^{1}H}NMR (151 MHz, CDCl$_3$) δ 153.2, 145.1, 142.2, 138.9, 134.4, 133.8, 133.1, 132.4, 129.6, 129.3, 128.4, 128.1, 127.7, 126.9, 126.7, 124.5, 71.6, 44.5, –0.8, –1.1 ppm. HRMS (DART) *m/z* calcd for $C_{24}H_{25}Cl_2OSi$ [M+H]$^+$: 427.1052; Found [M+H]$^+$: 427.1024.

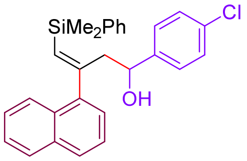

(*E*)-1-(4-chlorophenyl)-4-(dimethyl(phenyl)silyl)-3-(naphthalen-1-yl)but-3-en-1-ol (10v): According to the general procedure using Mg(OTf)$_2$ as Lewis acid and 1-iodonaphthalene as the aryl halide. Purification by silica gel chromatography (eluent: 0 – 10% EtOAc in hexanes) afforded 58.8 mg (98%) of **10v** as a colorless thick oil in >95:5 *E:Z*. Performing the reaction with 1-bromonapthalene provided 34.7 mg (58%) of **10v**. The stereochemistry was assigned by analogy to that of **10i**. R$_f$ = 0.41 (10% EtOAc/hexanes). ^{1}H NMR (600 MHz, CDCl$_3$) δ 8.03 (s, 1H), 7.86 – 7.92 (m, 1H), 7.81(d, *J* = 8.2 Hz, 1H), 7.69 – 7.74 (m, 2H), 7.43 – 7.53 (m, 6H), 7.35 (d, *J* = 7.0 Hz, 1H), 7.15 (d, *J* = 8.4 Hz, 2H), 6.88 (d, *J* = 8.4 Hz, 2H), 6.01 (s, 1H), 4.36 – 4.42 (m, 1H), 2.99 (dd, *J* = 14 Hz, 10 Hz, 1H), 2.89 (dd, *J* = 14 Hz, 3.6 Hz, 1H), 1.71 (s, 1H), 0.57 (s, 3H), 0.56 (s, 3H); ^{13}C{^{1}H}NMR (151 MHz, CDCl$_3$) δ: 154.8, 142.5, 142.4, 139.4, 133.93, 133.89, 133.84, 132.8, 130.7, 129.3, 128.4, 128.3,128.1, 127.5, 127.0, 126.1, 125.8, 125.5, 125.2, 124.5, 71.5, 47.5, –0.7, –0.8. HRMS (DART) *m/z* calcd for $C_{28}H_{28}ClOSi$ [M+H]$^+$: 443.1598; Found [M + H]$^+$: 443.1603.

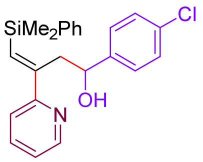

(*E*)-1-(4-chlorophenyl)-4-(dimethyl(phenyl)silyl)-3-(pyridin-2-yl)but-3-en-1-ol (10w): According to the general procedure using $Mg(OTf)_2$ as Lewis acid and 2-iodopyridine as the aryl halide. The reaction was heated at 80°C. Purification by silica gel chromatography (eluent: 0 – 10% EtOAc in hexanes) afforded 21.4 mg (40%) of **10w** as a colorless thick oil in in 93:7 *E:Z* ratio. The stereochemistry was assigned by analogy to that of **10i**. R_f =0.14 (10% EtOAc/hexanes. ^{1}H NMR (600 MHz, $CDCl_3$) δ 8.52 – 8.56 (m, 1H), 7.74 (td, *J* = 7.9 Hz, 1.7 Hz, 1H), 7.68 (d, *J* = 8.0 Hz, 1H), 7.56 – 7.61 (m, 2H), 7.36 – 7.42 (m, 3H), 7.22 – 7.26 (m, 1H), 7.22 (d, *J* = 8.4 Hz, 2H), 7.11 (d, *J* = 8.4 Hz, 2H), 6.49 (s, 1H), 4.68 (dd, *J* = 10, 2.6 Hz, 1H), 2.90 (dd, *J* = 14 Hz, 2.6 Hz, 1H), 2.84 (dd, *J* = 14 Hz, 10 Hz, 1H), 1.55 (s, 1H), 0.49 (s, 6H); ^{13}C{^{1}H}NMR (151 MHz, $CDCl_3$) δ 160.9, 154.6, 147.1, 144.5, 138.4, 137.4, 133.9, 133.6, 132.4, 129.4, 128.2, 128.1, 127.3, 122.3, 121.7, 74.3, 43.9, –0.7, –1.0 ppm HRMS (DART) *m/z* calcd for $C_{23}H_{25}ClNSiO$ $[M + H]^+$: 394.1394; Found $[M + H]^+$: 394.1401.

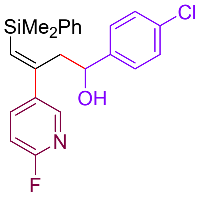

(*E*)-1-(4-chlorophenyl)-4-(dimethyl(phenyl)silyl)-3-(6-fluoropyridin-3-yl)but-3-en-1-ol (10x): According to the general procedure using $Mg(OTf)_2$ as Lewis acid and 2-fluoro-5-iodopyridine as the aryl halide. Purification by silica gel chromatography (eluent: 0 – 10% EtOAc in hexanes) afforded 30 mg (54%) of **10x** as a colorless thick oil in >95:5 *E:Z*. The stereochemistry was assigned by analogy to that of **10i**. R_f = 0.25 (10% EtOAc/hexanes). ^{1}H NMR (600 MHz, $CDCl_3$) δ 8.23 (s, 1H), 7.77 (t, *J* = 7.1 Hz, 1H), 7.54 – 7.60 (m, 2H), 7.35 – 7.44 (m, 3H), 7.20 (d, *J* = 8.2 Hz, 2H), 6.95 (d, *J* = 8.2 Hz, 2H), 6.89 (s, 1H), 6.05 (s, 1H), 4.46 (dd, *J* = 9.0, 4.6 Hz, 1H), 2.90 (dd, *J* = 14 Hz, 9.0 Hz, 1H), 2.83 (dd, *J* = 14 Hz, 4.6 Hz, 1H), 1.58 (br s, 1H), 0.47 (s, 3H), 0.46 (s, 3H); ^{13}C{^{1}H}NMR (151 MHz, $CDCl_3$) δ 162.9 (d, $^1J_{CF}$ = 240 Hz), 150.1, 145.2 (d, $^3J_{CF}$ = 14 Hz), 142.1, 139.1 (d, $^3J_{CF}$ = 7.8 Hz), 138.7, 137.0, 133.8, 133.4, 133.2, 129.3, 128.5, 128.1, 126.9, 108.9 (d, $^2J_{CF}$ = 37 Hz), 71.7, 44.2, –1.0, –1.1 ppm. ^{19}F{^{1}H} NMR (565 MHz, $CDCl_3$) δ –69.1HRMS (DART) *m/z* calcd for $C_{23}H_{24}ClFNSiO$ $[M + H]^+$: 412.1300; Found $[M + H]^+$: 412.1311.

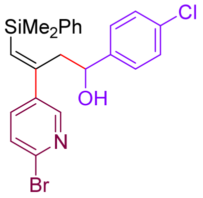

(*E*)-3-(6-bromopyridin-3-yl)-1-(4-chlorophenyl)-4-(dimethyl(phenyl)silyl)but-3-en-1-ol (10y): According to the general procedure using $Mg(OTf)_2$ as Lewis acid and 2-bromo-5-iodopyridine as the aryl halide. Purification by silica gel chromatography (eluent: 0 – 10% EtOAc in hexanes) afforded 29.4 mg (46%) of **10y** as a colorless thick oil in >95:5 *E:Z*. The stereochemistry was assigned by analogy to that of **10i**. R_f =0.34 (10% EtOAc/hexanes). ^{1}H NMR (600 MHz, $CDCl_3$) δ 8.37 (d, *J* = 2.4 Hz, 1H), 7.54 – 7.59 (m, 2H), 7.52 (dd, *J* = 8.3 Hz, 2.4 Hz, 1H), 7.42 (d, *J* = 8.3 Hz, 1H), 7.37 – 7.43 (m, 3H), 7.21 (d, *J* = 8.4 Hz, 2H), 6.95 (d, *J* = 8.4 Hz, 2H), 6.08 (s, 1H), 4.46 – 4.36 (m, 1H), 2.89 (dd, *J* = 14, 9.1 Hz, 1H), 2.81 (dd, *J* = 14, 4.7 Hz, 1H), 1.54 (s, 1H), 0.47 (s, 3H), 0.46 (s, 3H); ^{13}C{^{1}H}NMR (151 MHz, $CDCl_3$) δ: 150.1, 147.9, 142.0, 140.8, 138.6, 138.2, 136.4, 134.1, 133.8, 133.3, 129.4, 128.5, 128.2, 127.5, 126.9, 71.8, 44.0, –1.0, –1.2 ppm.HRMS (DART) *m/z* calcd for $C_{23}H_{24}BrClNSiO$ $[M + H]^+$: 472.0499; Found $[M + H]^+$: 472.0522.

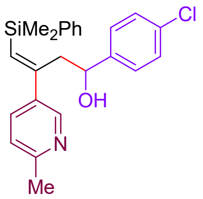

(*E*)-1-(4-chlorophenyl)-4-(dimethyl(phenyl)silyl)-3-(6-methylpyridin-3-yl)but-3-en-1-ol (10z): According to the general procedure using $Mg(OTf)_2$ as Lewis acid and 5-iodo-2-methylpyridine as the aryl halide. Purification by silica gel chromatography (eluent: 0 – 10% EtOAc in hexanes) afforded 28 mg (51%) of **10z** as a colorless thick oil in >95:5 *E:Z*. The stereochemistry was assigned by analogy to that of **10i**. R_f = 0.15 (10% EtOAc/hexanes). 1H NMR (600 MHz, CDCl3) δ 8.53 (d, J = 2.2 Hz, 1H), 7.55 – 7.61 (m, 3H), 7.37 – 7.42 (m, 3H), 7.21 (d, J = 8.4 Hz, 2H), 7.12 (d, J = 8.0 Hz, 1H), 6.98 (d, J = 8.4 Hz, 2H), 6.07 (s, 1H), 4.46 – 4.52 (m, 1H), 2.88 (dd, J = 14 Hz, 8.8 Hz, 1H), 2.86 (dd, J = 14 Hz, 5.0 Hz, 1H), 2.56 (s, 3H), 1.64 (d, J = 3.5 Hz, 1H), 0.47 (s, 3H), 0.46 (s, 3H); $^{13}C\{^1H\}$NMR (151 MHz, $CDCl_3$) δ: 157.5, 151.3, 146.8, 142.4, 139.0, 135.8, 134.2, 133.8, 133.0, 132.2, 129.2, 128.4, 128.1, 126.9, 122.8, 71.5, 44.4, 23.9, –0.8, –1.0 ppm. HRMS (DART) *m/z* calcd for $C_{24}H_{27}ClNSiO$ $[M+H]^+$: 408.1550; Found $[M + H]^+$: 408.1570.

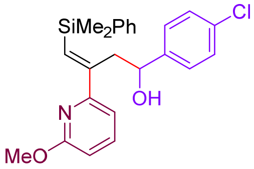

(*E*)-1-(4-chlorophenyl)-4-(dimethyl(phenyl)silyl)-3-(6-methoxypyridin-2-yl)but-3-en-1-ol (10aa): According to the general procedure using $Mg(OTf)_2$ as Lewis acid and 2-bromo-6-methoxypyridine as the aryl halide. Purification by silica gel chromatography (eluent: 0 – 10% EtOAc in hexanes) afforded 33.4 mg (58%) of **10aa** as a colorless thick oil as a 90:10 *E:Z* ratio contaminated with the branched isomer (93:7 linear/branched). The stereochemistry was assigned by analogy to that of **10i**. R_f =0.41 (10% EtOAc/hexanes. ^1H NMR (600 MHz, $CDCl_3$) δ 7.56 – 7.63 (m, 3H), 7.35 – 7.41 (m, 3H), 7.21 – 7.25 (m, 3H), 7.10 (d, J = 8.4 Hz, 2H), 6.71 (d, J = 8.2 Hz, 1H), 6.51 (s, 1H), 6.02 (br s, 1H), 4.71 (dd, J = 10 Hz, 2.9 Hz, 1H), 4.00 (s, 3H), 2.94 (dd, J = 14 Hz, 2.9 Hz, 1H), 2.81 (dd, J = 14 Hz, 10 Hz, 1H), 0.50 (s, 3H), 0.49 (s, 3H); $^{13}C\{^1H\}$NMR (151 MHz, $CDCl_3$) δ: 162.9, 158.8, 153.9, 143.9, 139.6, 138.6, 133.9, 132.8, 132.6, 129.3, 128.3, 128.1, 127.1, 114.4, 109.8, 74.0, 53.9, 43.4, –0.7, –0.9 ppm. HRMS (DART) *m/z* calcd for $C_{24}H_{27}ClNSiO_2$ $[M + H]^+$: 424.1500; Found $[M + H]^+$: 424.1501.

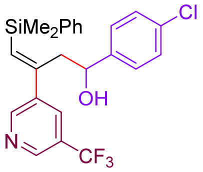

(*E*)-1-(4-chlorophenyl)-4-(dimethyl(phenyl)silyl)-3-(5-(trifluoromethyl)pyridin-3-yl)but-3-en-1-ol (10ab): According to the general procedure using $Mg(OTf)_2$ as Lewis acid and 3-Bromo-5-(trifluoromethyl)pyridine as the aryl halide. The reaction was heated at 80°C. Purification by silica gel chromatography (eluent: 0 – 10% EtOAc in hexanes) afforded 28 mg (45%) of **10ab** as a colorless thick oil in >95:5 *E:Z*. The stereochemistry was assigned by analogy to that of **10i**. R_f = 0.24 (10% EtOAc/hexanes). ^1H NMR (600 MHz, $CDCl_3$) δ 8.78 (s, 1H), 8.76 (s, 1H), 7.81 (s,1H), 7.60 – 7.62 (m, 2H), 7.42 – 7.45 (m, 3H), 7.19 (d, J = 8.4 Hz, 2H), 6.93 (d, J = 8.4 Hz, 2H), 6.14 (s, 1H), 4.43 (dd, J = 8.9 Hz, 5.0 Hz, 1H), 2.94 (dd, J = 14 Hz, 8.9 Hz, 1H), 2.87 (dd, J = 14 Hz, 5.0 Hz, 1H), 1.55 (br s, 1H), 0.50 (s, 3H), 0.49 (s, 3H); $^{13}C\{^1H\}$NMR (151 MHz, $CDCl_3$): δ 150.8, 150.0, 145.1 (q, $^3J_{CF}$ = 3.6 Hz), 141.9, 139.1, 138.4, 135.5, 133.9, 133.5, 130.6 (q, $^3J_{CF}$ = 3.8 Hz), 129.5, 128.6, 128.2, 126.9, 126.2 (q, $^2J_{CF}$ = 32 Hz), 123.3 (q, $^1J_{CF}$ = 272 Hz), 72.1, 43.9, –1.0, –1.2 ppm. $^{19}F\{^1H\}$NMR (565 MHz, $CDCl_3$) δ: –62.4 ppm. HRMS (DART) *m/z* calcd for $C_{24}H_{24}ClF_3NOSi$ $[M + H]^+$: 462.1268; Found $[M + H]^+$: 462.1286.

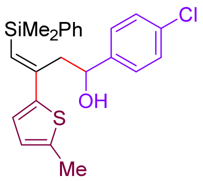

(*E*)-1-(4-chlorophenyl)-4-(dimethyl(phenyl)silyl)-3-(5-methylthiophen-2-yl)but-3-en-1-ol (10ac): According to the general procedure using $Mg(OTf)_2$ as Lewis acid and 2-Bromo-5-methylthiophene as the aryl halide. Purification by silica gel chromatography (eluent: 0 – 10% EtOAc in hexanes) afforded 32 mg (57%) of **10ac** as a colorless thick oil in >95:5 *E:Z*. The stereochemistry was assigned by analogy to that of **10i**. R_f = 0.40 (10% EtOAc/hexanes). ^{1}H NMR (600 MHz, $CDCl_3$) δ 7.54 – 7.60 (m, 2H), 7.34 – 7.41(m, 3H), 7.26 (d, *J* = 8.4 Hz, 2H), 7.13 (d, *J* = 8.4 Hz, 2H), 6.89 (d, *J* = 3.5 Hz, 1H), 6.66 (dd, *J* = 3.5 Hz, 0.6 Hz, 1H), 6.16 (s, 1H), 4.78 – 4.84 (m, 1H), 2.72 – 2.81 (m, 2H), 2.47 (s, 3H), 1.85 (t, *J* = 2.7 Hz, 1H), 0.47 (s, 3H), 0.45 (s, 3H); ^{13}C{^{1}H}NMR (151 MHz, $CDCl_3$) δ: 146.5, 144.8, 142.4, 140.4, 139.1, 133.9, 133.0, 129.2, 128.4, 128.0, 127.5, 126.9, 125.9, 124.1, 75.0, 44.7, 15.5, –0.6, –0.7 ppm. HRMS (DART) *m/z* calcd for $C_{23}H_{25}ClOSSi$ $[M]^+$: 412.1084; Found $[M]^+$: 412.1085.

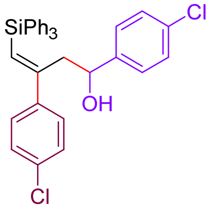

(*E*)-1,3-bis(4-chlorophenyl)-4-(triphenylsilyl)but-3-en-1-ol (10ad): According to the general procedure using $Mg(OTf)_2$ as Lewis acid and 1-chloro-4-iodobenzene, purification by silica gel chromatography (eluent: 0 – 10% EtOAc in hexanes) afforded 54 mg (73%) of **10ad** as an amorphous white solid in >95:5 *E:Z*. The stereochemistry was assigned by analogy to that of **10i**. R_f = 0.33 (15% EtOAc/hexanes). ^{1}H NMR ($CDCl_3$, 600 MHz) δ: 7.58 – 7.63 (m, 6H), 7.37 – 7.46 (m, 11H), 7.35 (d, *J* = 7.7 Hz, 2H), 7.08 (d, *J* = 8.4 Hz, 2H), 6.59 (d, *J* = 8.4 Hz, 2H), 6.44 (s, 1H), 4.36 (dd, *J* = 9.3 Hz, 4.7 Hz, 1H), 2.85 (dd, *J* = 15 Hz, 9.3 Hz, 1H), 2.83 (dd, *J* = 15 Hz, 4.7 Hz, 1H), 1.56 (s, 1H); ^{13}C{^{1}H}NMR (151 MHz, $CDCl_3$) δ 156.8, 142.1, 141.9, 135.9, 135.0, 133.8, 132.9, 129.7, 128.6, 128.2, 128.1, 127.9, 127.5, 126.9, 71.8, 44.5 ppm HRMS (DART) *m/z* calcd for $C_{34}H_{29}Cl_2OSi$ $[M+H]^+$: 551.1365; Found $[M+H]^+$: 551.1394.

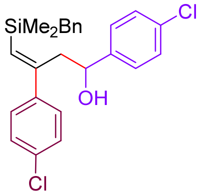

(*E*)-4-(benzyldimethylsilyl)-1,3-bis(4-chlorophenyl)but-3-en-1-ol (10ae): According to the general procedure using 30.0 mg (0.213 mmol) of 4-chlorobenzaldehyde, $Mg(OTf)_2$ as Lewis acid and 1-chloro-4-iodobenzene, purification by silica gel chromatography (eluent: 0 – 10% EtOAc in hexanes) afforded 91.2 mg (97%) of **10ae** as a slight pink oil in >95:5 *E:Z*. The stereochemistry was assigned by analogy to that of **10i**. R_f = 0.25 (15% EtOAc/hexanes). ^{1}HNMR ($CDCl_3$, 600 MHz) δ: 7.32 (d, = 8.6 Hz, 2H), 7.27 – 7.31 (m, 4H), 7.21 (t, *J* = 7.6 Hz, 2H), 7.18 (d, *J* = 8.3 Hz, 2H), 7.07 (t, *J* = 7.3 Hz, 1H), 7.00 (d, *J* = 7.2 Hz, 2H), 5.84 (s, 1H), 4.53 (t, *J* = 6.7 Hz, 1H), 2.79 – 2.88 (m, 2H), 2.20 (s, 2H), 1.73 (d, *J* = 1.4 Hz, 1H), 0.19 (s, 3H), 0.16 (s, 3H); ^{13}C{^{1}H}NMR (151 MHz, $CDCl_3$): δ 152.6, 142.3, 141.7, 139.7, 133.5, 133.2, 131.9, 128.6, 128.5, 128.21, 128.19, 127.6, 127.0, 124.2, 71.5, 44.7, 26.7, –1.4, –1.5 ppm. HRMS (DART) *m/z* calcd for $C_{25}H_{26}Cl_2OSi$ $[M]^+$: 440.1130; Found $[M]^+$: 440.1138.

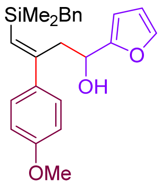

(*E*)-4-(benzyldimethylsilyl)-1-(furan-2-yl)-3-(4-methoxyphenyl)but-3-en-1-ol (10af): According to the general procedure using 12.9 µL (0.156 mmol) of 2-furfural, $Mg(OTf)_2$ as Lewis acid and 1-iodo-4-methoxybenzene, purification by silica gel chromatography (eluent: 0 – 15% EtOAc in hexanes) afforded 60.3 mg (98%) of **10af** as a colorless thick oil in >95:5 *E:Z*. The stereochemistry was assigned by analogy to that of **10i**. R_f = 0.27 (20% EtOAc/hexanes). ^{1}HNMR ($CDCl_3$, 600 MHz) δ: 7.35 (dd, *J* = 1.7 Hz, 0.7 Hz, 1H), 7.30 (d, *J* = 8.8 Hz, 2H), 7.21 (t, *J* = 7.7 Hz, 2H), 7.08 (t, *J* = 7.4 Hz, 1H), 7.03 (d, *J* = 7.0 Hz, 2H), 6.86 (d, *J* = 8.8 Hz, 2H), 6.29 (dd, *J* = 3.2 Hz, 1.8 Hz, 1H), 6.13 (d, *J* = 3.2 Hz, 1H), 5.80 (s, 1H), 4.60 – 4.65 (m, 1H), 3.82 (s, 3H), 2.95 – 3.06 (m, 2H), 2.23 (s, 2H), 1.75 (d, *J* = 3.9 Hz, 1H), 0.201 (s, 3H), 0.196 (s, 3H); ^{13}C{^{1}H}NMR (151 MHz, $CDCl_3$): δ 159.3, 155.8, 153.0, 141.9, 140.0, 135.4, 129.1, 128.3, 128.1, 127.5, 124.0, 113.7, 110.0, 106.0, 66.1, 55.3, 40.0, 26.9, –1.39, –1.42 ppm. HRMS (DART) *m/z* calcd for $C_{24}H_{28}O_3Si$ $[M]^+$: 392.1808; Found $[M]^+$: 392.1825.

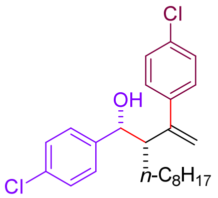

Compound 11a: According to the general procedure using 15.0 mg (0.107 mmol) of 4-chlorobenzaldehyde, $Sn(OTf)_2$ as Lewis acid and 1-chloro-4-iodobenzene, purification by silica gel chromatography (eluent: 0 – 15% EtOAc in hexanes) afforded 35.8 mg (83%) of **11a** as a colorless thick oil in 87:13 dr. The major diastereomer was assigned by comparison of the ^{1}HNMR spectra to the literature.[11] R_f = 0.31 (15% EtOAc/hexanes). ^{1}HNMR ($CDCl_3$, 600 MHz) δ: 7.21 – 7.25 (m, 4H), 7.17 (d, *J* = 8.4 Hz, 2H), 7.11 (d, *J* = 8.6 Hz, 2H), 5.37 (s, 1H), 5.12 (s, 1H), 4.58 (dd, *J* = 5.3 Hz, 2.0 Hz, 1H), 2.81 – 2.86 (m, 1H), 1.95 (d, *J* = 2.0 Hz, 1H), 1.56 – 1.70 (m, 2H), 1.34 – 1.43 (m, 1H), 1.12 – 1.32 (m, 11H), 0.86 (t, *J* = 7.0 Hz, 3H); ^{13}C{^{1}H}NMR (151 MHz, $CDCl_3$): δ 148.6, 141.7, 141.1, 133.3, 132.9, 128.4, 128.1, 127.81, 127.78, 114.9, 75.5, 52.0, 31.8, 29.8, 29.4, 29.2, 28.3, 27.3, 22.6, 14.1 ppm. HRMS (DART) *m/z* calcd for $C_{24}H_{31}Cl_2O$ $[M+H]^+$: 405.1752; Found $[M + H]^+$: 405.1728.

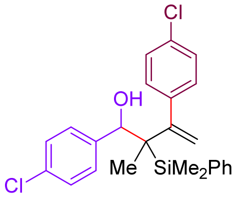

1,3-bis(4-chlorophenyl)-2-(dimethyl(phenyl)silyl)-2-methylbut-3-en-1-ol (11b): According to the general procedure using $Eu(OTf)_3$ as Lewis acid and 1-chloro-4-iodobenzene. After workup, the %yield was initially determined on the crude mixture by quantitative ^{1}HNMR spectroscopy using either 1,3,5-trimethoxybenzene or 1,4-dioxane as analytical standard (45% yield by NMR). Unreacted aldehyde cannot be separated by chromatography. Therefore, the crude material was dissolved in 0.4 mL of MeOH and 7.0 mg (0.19 mmol) of $NaBH_4$ was added. After agitating for ~1 min, 5% aq. NH_4Cl was added and the mixture extracted with MTBE. After drying the organics with Na_2SO_4 and concentration *in vacuo*, the residue was purified by flash chromatography on silica gel (hexanes/EtOAc 0 – 10%) followed by preparatory TLC (10% EtOAc/hexanes) to obtain 14.8 mg (25%) of a single diastereomer of **11b** as a white amorphous solid. R_f = 0.29 (10% EtOAc/hexanes). ^{1}HNMR ($CDCl_3$, 600 MHz) δ: 7.57 – 7.62 (m, 2H), 7.32 – 7.41 (m, 3H), 7.27 – 7.31 (m, 4H), 6.97 (d, *J* = 8.6 Hz, 2H), 6.15 (d, *J* = 8.6 Hz, 2H), 5.79 (d, *J* = 1.7 Hz, 1H), 5.15 (d, *J* = 1.7 Hz, 1H), 4.76 (d, *J* = 2.6 Hz, 1H), 2.11 (d, *J* = 2.6 Hz, 1H), 0.79 (s, 3H), 0.46 (s, 3H), 0.42 (s, 3H); ^{13}C{^{1}H}NMR (151 MHz, $CDCl_3$): δ 151.4, 144.5, 142.5, 139.6, 137.1, 135.5, 133.9, 131.7, 131.3, 131.1, 129.8, 129.5, 129.2, 121.0, 81.3, 41.5, 21.4, –0.3, –0.8 ppm. HRMS (DART) *m/z* calcd for $C_{25}H_{27}Cl_2OSi$ $[M + H]^+$: 441.1208; Found $[M + H]^+$: 441.1198.

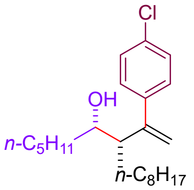

Compound 11c: According to the general procedure using 13.5 µL (0.110 mmol) of *n*-hexanal, $Sn(OTf)_2$ as Lewis acid and 1-chloro-4-iodobenzene, purification by silica gel chromatography (eluent: 0 – 15% EtOAc in hexanes) afforded 15.9 mg (40%) of **11c** as a colorless thick oil in 64:36 dr. The major diastereomer was assigned by analogy to compound **11a**. R_f = 0.34 (15% EtOAc/hexanes). ^{1}HNMR ($CDCl_3$, 600 MHz) δ: 7.26 – 7.32 (m, 4H, major + minor diastereomers), 5.40 (s, 1H, minor diast.), 5.38 (s, 1H, major diast.), 5.17 (s, 1H, minor diast.), 5.09 (s, 1H, major diast.), 3.56 – 3.62 (m, 1H, minor diast.), 3.44 – 3.50 (m, 1H, major diast.), 2.55 – 2.61 (m, 1H, minor diast.), 2.51 – 2.55 (m, 1H, major diast.), 1.71 – 1.78 (m, 1H, major diast.), 1.63 (d, *J* = 5.9 Hz, 1H, minor diast.), 1.49 (d, *J* = 4.3 Hz, 1H, major diast.), 1.18 – 1.46 (m, 21H, major + minor diast.) 0.85 – 0.91 (m, 3H, major + minor diast.); $^{13}C\{^1H\}$NMR (151 MHz, $CDCl_3$): δ (major + minor diast.): 149.55, 149.48, 142.3, 141.9, 133.2, 133.1, 128.5, 128.4, 128.1, 127.9, 115.0, 114.0, 74.1, 73.9, 50.9, 50.2, 34.8, 34.7, 31.86, 31.85, 31.77, 31.5, 29.9, 29.8, 29.7, 29.5, 29.4, 29.3, 29.2, 28.7, 27.5, 27.4, 25.9, 25.6, 22.68, 22.65, 22.60, 22.57, 14.10, 14.09, 14.01, 14.00 ppm. HRMS (DART) *m/z* calcd for $C_{23}H_{37}ClO$ $[M]^+$: 364.2533; Found $[M]^+$: 364.2503.

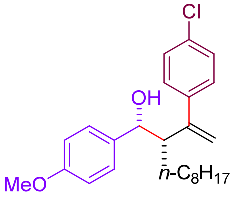

Compound 11d: According to the general procedure using 13.4 µL (0.110 mmol) of 4-anisaldehyde, $Sn(OTf)_2$ as Lewis acid and 1-chloro-4-iodobenzene, purification by silica gel chromatography (eluent: 0 – 20% EtOAc in hexanes) afforded 28.3 mg (64%) of **11d** as a colorless thick oil in 87:13 dr. The major diastereomer was assigned by analogy to compound **11a**. R_f = 0.22 (15% EtOAc/hexanes). ^{1}HNMR ($CDCl_3$, 600 MHz): δ 7.21 (d, *J* = 8.5 Hz, 2H), 7.14 (d, *J* = 8.6 Hz, 2H), 7.07 (d, *J* = 8.5 Hz, 2H), 6.78 (d, *J* = 8.6 Hz, 2H), 5.32 (s, 1H), 5.10 (s, 1H), 4.56 (d, *J* = 6.0 Hz, 1H), 3.77 (s, 3H), 2.82 – 2.88 (m, 1H), 1.86 (s, 1H), 1.75 – 1.83 (m, 1H), 1.55 – 1.64 (m, 1H), 1.36 – 1.44 (m, 1H), 1.16 – 1.33 (m, 11H), 0.87 (t, *J* = 7.0 Hz, 3H); $^{13}C\{^1H\}$NMR (151 MHz, $CDCl_3$): δ 158.8, 149.0, 142.1, 134.9, 132.9, 128.2, 127.9, 127.6, 114.6, 113.4, 76.3, 55.2, 52.2, 31.8, 29.8, 29.4, 29.2, 29.1, 27.4, 22.6, 14.1 ppm. HRMS (DART) *m/z* calcd for $C_{17}H_{16}ClO_2$ $[M]^+$: 287.0839; Found $[M]^+$: 287.08632.

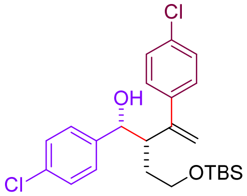

***syn*-2-(2-((*tert*-butyldimethysilyl)oxy)ethyl)-1,3-bis(4-chlorophenyl)but-3-en-1-ol (11e)**: According to the general procedure using 15.0 mg (0.107 mmol) of 4-chlorobenzaldehyde, $Sn(OTf)_2$ as Lewis acid and 1-chloro-4-iodobenzene, purification by silica gel chromatography (eluent: 0 – 10% EtOAc in hexanes) afforded 38.3 mg (80%) of **11e** as a colorless thick oil in 81:19 dr. The major diastereomer was assigned by analogy to compound **11a**. R_f = 0.22 (15% EtOAc/hexanes). ^{1}HNMR ($CDCl_3$, 600 MHz) δ (major diast.): 7.18 – 7.23 (m, 6H), 7.12 (d, *J* = 8.5 Hz, 2H), 5.35 (s, 1H), 5.17 (s, 1H), 4.69 (d, *J* = 6.4 Hz, 1H), 3.72 (dd, *J* = 10 Hz, 5.1 Hz, 1H), 3.58 – 3.63 (m, 1H), 3.20 (d, *J* = 2.5 Hz, 1H), 3.10 (q, *J* = 6.4 Hz, 1H), 1.84 – 1.92 (m, 2H), 0.89 (s, 9H), 0.033 (s, 3H), 0.025 (s, 3H); $^{13}C\{^1H\}$NMR (151 MHz, $CDCl_3$): δ (major diast.) 148.9, 141.4, 141.3, 133.3, 132.8, 128.3, 128.1, 128.0, 127.8, 114.9, 75.5, 60.9, 49.2, 32.4, 25.9, 18.2, –5.5 ppm. HRMS (DART) *m/z* calcd for $C_{24}H_{32}Cl_2O_2Si$ $[M]^+$: 450.1549; Found $[M]^+$: 450.1554.

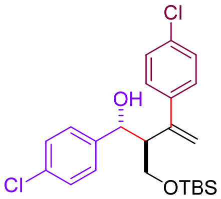

***anti*-2-(((*tert*-butyldimethysilyl)oxy)methyl)-1,3-bis(4-chlorophenyl)but-3-en-1-ol (11f)**: According to the general procedure using 15.0 mg (0.107 mmol) of 4-chlorobenzaldehyde, $Sn(OTf)_2$ as Lewis acid and 1-chloro-4-iodobenzene, purification by silica gel chromatography (eluent: 0 – 10% EtOAc in hexanes; R_f = 0.19 in 10% EtOAc/hexanes) afforded 21.6 mg of **11f** as a colorless thick oil in 64:36 dr contaminated with unreacted aldehyde. Purity quantification of this mixture using quantitative ^{1}HNMR spectroscopy determined a 45% yield. To obtain analytically pure material, the mixture was dissolved in 0.3 mL of MeOH and 3.5 mg (0.093 mmol) of $NaBH_4$ was added. After agitating for ~1 min, 5% aq. NH_4Cl was added and the mixture extracted with MTBE. After drying the organics with Na_2SO_4 and concentration *in vacuo*, the residue was purified by flash chromatography on silica gel (hexanes/EtOAc 0 – 10%) to obtain 12.2 mg (26%) of pure **11f** as a mixture of diastereomers. The major diastereomer was assigned by analogy to compound **11g**. ^{1}HNMR ($CDCl_3$, 600 MHz) δ (major diast): 6.92 – 7.25 (m, 8H), 5.30 (s, 1H), 5.22 (s, 1H), 4.93 (dd, *J* = 7.7 Hz, 2.6 Hz, 1H), 4.69 (d, *J* = 2.6 Hz, 1H), 3.95 (dd, *J* = 10 Hz, 7.7 Hz, 1H), 3.93 (dd, *J* = 10 Hz, 4.2 Hz, 1H), 2.94 (td, *J* = 7.7 Hz, 4.2 Hz, 1H), 0.93 (s, 9H), 0.093 (s, 3H), 0.087 (s, 3H); minor diast.: 7.18 – 7.29 (m, 8H), 5.37 (s, 1H), 5.03 (s, 1H), 4.96 (t, *J* = 5.3 Hz, 1H), 3.66 (dd, *J* = 10 Hz, 3.6 Hz, 1H), 3.65 (dd, *J* = 10 Hz, 5.2 Hz, 1H), 3.42 (d, *J* = 5.3 Hz, 1H), 3.12 (q, *J* = 5.3 Hz, 1H), 0.91 (s, 9H), 0.039 (s, 3H), 0.035 (s, 3H); ^{13}C{^{1}H}NMR (151 MHz, $CDCl_3$): δ (major diast.): 146.1, 141.4, 140.9, 133.3, 133.0, 128.34, 128.30, 128.1, 127.5, 115.9, 78.2, 66.5, 52.2, 25.7, 18.1, –5.60, –5.69; (minor diast): 145.4, 140.8, 140.2, 133.4, 133.0, 128.5, 128.04, 128.00, 127.9, 115.8, 74.1, 63.7, 52.3, 25.8, 18.1, –5.5, –5.6 ppm. HRMS (DART) *m/z* calcd for $C_{23}H_{30}Cl_2O_2Si$ $[M]^+$: 436.1392; Found $[M]^+$: 436.1372.

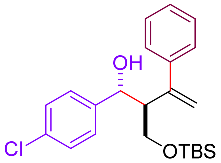

***anti*-2-(((*tert*-butyldimethysilyl)oxy)methyl)-1-(4-chlorophenyl)-3-phenylbut-3-en-1-ol (11g)**: According to the general procedure using 15.0 mg (0.107 mmol) of 4-chlorobenzaldehyde, $Sn(OTf)_2$ as Lewis acid and iodobenzene, purification by silica gel chromatography (eluent: 0 – 10% EtOAc in hexanes; R_f = 0.26 in 10% EtOAc/hexanes) afforded 24.9 mg of **11g** as a colorless thick oil in 70:30 dr contaminated with unreacted aldehyde. Purity quantification of this mixture using quantitative ^{1}HNMR spectroscopy determined a 53% yield. To obtain analytically pure material, the mixture was dissolved in 0.3 mL of MeOH and 3.5 mg (0.093 mmol) of $NaBH_4$ was added. After agitating for ~1 min, 5% aq. NH_4Cl was added and the mixture extracted with MTBE. After drying the organics with Na_2SO_4 and concentration *in vacuo*, the residue was purified by flash chromatography on silica gel (hexanes/EtOAc 0 – 10%) to obtain 13.8 mg (32%) of pure **11g** as a mixture of diastereomers. The major diastereomer was assigned by deprotection of the TBS group using TBAF in THF followed by comparison of the ^{1}HNMR spectrum of the obtained free alcohol to that reported in the literature.[11] ^{1}HNMR ($CDCl_3$, 600 MHz) δ: (major diast): 7.00 – 7.35 (m, 9H), 5.31 (s, 1H), 5.20 (s, 1H), 4.94 – 4.97 (m, 1H), 4.80 (d, *J* = 2.5 Hz, 1H), 3.93 – 3.99 (m, 2H), 3.01 (td, *J* = 7.5 Hz, 4.3 Hz, 1H), 0.94 (s, 9H), 0.095 (s, 3H), 0.087 (s, 3H); minor diast.: 7.00 – 7.35 (m, 8H), 5.38 (s, 1H), 4.95 – 4.98 (m, 2H), 3.68 (dd, *J* = 10 Hz, 4.6 Hz, 1H), 3.66 (dd, *J* = 10 Hz, 6.9 Hz, 1H), 3.60 (d, *J* = 5.3 Hz, 1H), 3.20 – 3.24 (m, 1H), 0.93 (s, 9H), 0.052 (s, 3H), 0.047 (s, 3H); ^{13}C{^{1}H}NMR (151 MHz, $CDCl_3$): δ (major diast.): 147.1, 142.3, 141.5, 132.8, 128.4, 128.1, 128.0, 127.3, 126.2, 115.4, 78.3, 66.7, 52.2, 25.7, 18.0, –5.6, –5.7; (minor diast): 146.3, 142.2, 140.2, 132.9, 127.9, 127.8, 127.7, 127.6, 126.5, 115.3, 74.2, 63.7, 52.1, 25.8, 18.1, –5.55, –5.64 ppm. HRMS (DART) *m/z* calcd for $C_{23}H_{32}ClO_2Si$ $[M + H]^+$: 403.1860; Found $[M + H]^+$: 403.1875.

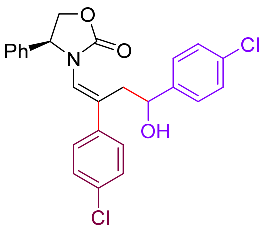

(4*S*)-3-((*E*)-2,4-bis(4-chlorophenyl)-4-hydroxybut-1-en-1-yl)-4-phenyloxazolidin-2-one (10ag): According to the general procedure using 15.0 mg (0.107 mmol) of 4-chlorobenzaldehyde, $Mg(OTf)_2$ as Lewis acid and 1-chloro-4-iodobenzene, the reaction was conducted at rt for 24 h followed by heating at 60 °C for 9 h before the described workup and purification by silica gel chromatography (eluent: 15 – 50% EtOAc in hexanes) afforded 28.9 mg (60%) of **10ag** as a white amorphous solid in 80:20 dr at the carbinol stereocenter. The *E*-alkene configuration was assigned by analogy to **10i**. *E/Z* ratio was >95:5. R_f = 0.27 (50% EtOAc/hexanes). ^{1}HNMR ($CDCl_3$, 600 MHz) δ: major diast.: 7.20 – 7.46 (m, 13H), 5.64 (d, *J* = 1.1 Hz, 1H), 4.83 (dd, *J* = 8.8 Hz, 4.5 Hz, 1H), 4.75 (t, *J* = 8.8 Hz, 1H), 4.53 – 4.59 (m, 2H), 4.32 (dd, *J* = 8.8 Hz, 4.5 Hz, 1H), 2.82 – 2.94 (m, 2H); minor diast.: 7.20 – 7.46 (m, 13H), 5.68 (d, *J* = 1.6 Hz, 1H, minor diast.), 4.99 – 5.05 (m, 2H), 4.73 – 4.78 (m, 1H), 4.52 – 4.56 (m, 1H), 4.19 (t, *J* = 8.9 Hz, 1H), 2.96 (dd, *J* = 15 Hz, 11 Hz, 1H), 2.79 (ddd, *J* = 15 Hz, 4.3 Hz, 1.5 Hz, 1H); $^{13}C\{^1H\}$NMR (151 MHz, $CDCl_3$): δ major diast.: 156.7, 143.9, 137.1, 137.0, 136.4, 133.9, 132.6, 129.6, 129.4, 128.7, 128.4, 128.3, 126.9, 126.7, 121.5, 70.11, 70.05, 62.2, 40.4 ppm. HRMS (DART) *m/z* calcd for $C_{25}H_{22}Cl_2NO_3$ $[M + H]^+$: 454.0977; Found $[M + H]^+$: 454.0971.

<u>1.0 mmol scale run:</u>

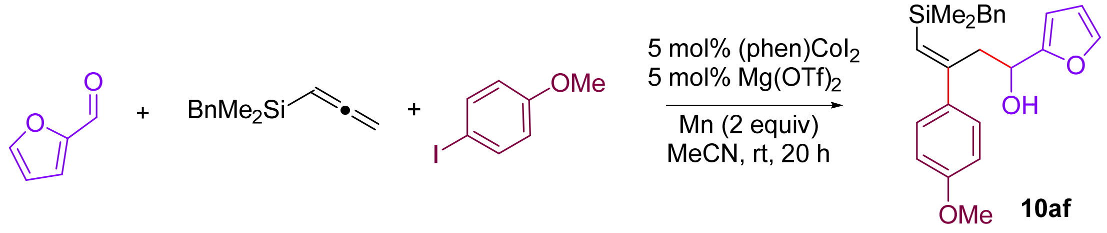

10af

To a flame-dried 20 mL screw-cap vial with magnetic stir-bar in an Ar-filled glove-box was charged 24.7 mg (0.0501 mmol, 5 mol%) of (phen)CoI_2, 16.2 mg (0.0501 mmol, 5 mol%) of $Mg(OTf)_2$, 110 mg (2.0 mmol, 2 equiv) of Mn, and 3.5 mL of acetonitrile. The mixture was allowed to stir for 5 min, and then, 1-iodo-4-methoxybenzene (353 mg, 1.50 mmol), $BnMe_2Si$-CH=C=CH_2 (255 mg, 1.35 mmol), and 83.0 µL of 2-furfural (96.3 mg, 1.00 mmol) were added sequentially. The vial was sealed with a screw-cap, removed from the glove-box, and allowed to stir rapidly at ambient temperature for 20 h. Methyl *tert*-butyl ether (MTBE, 20 mL) was then added, and the mixture was filtered through a small pad of silica gel by vacuum filtration, followed by rinsing of the filter-cake with additional MTBE (2x5 mL). To the combined organics were then added 15 mL of water followed by 1 mL of 29 wt% aqueous NH_4OH. After shaking in a separatory funnel, the layers were separated, and the organic layer was washed again as before (15 mL H_2O + 1 mL aq. 29 wt% NH_4OH). The organic layer was then dried with anhydrous Na_2SO_4 and concentrated *in vacuo*. The crude residue was then purified by flash chromatography on silica gel (0 – 20% EtOAc in hexanes) to afford 351 mg (89%) of **10af** as a slight yellow oil.

Competition experiment:

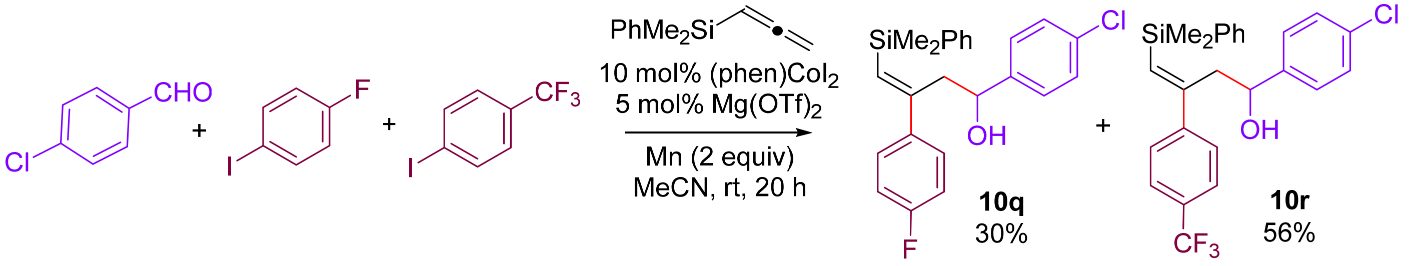

To a flame-dried 2-dram screw-cap vial with magnetic stir-bar in an Ar-filled glove-box was charged 5.3 mg (0.011 mmol) of (phen)CoI_2, 1.7 mg (0.0053 mmol) of $Mg(OTf)_2$, 11.7 mg (0.213 mmol) of Mn, and 0.33 mL of acetonitrile. The mixture was allowed to stir for 5 min, and then, 1-fluoro-4-iodobenzene (18.5 µL, 0.160 mmol), 4-iodobenzotrifluoride (23.5 µL, 0.160 mmol), $PhMe_2Si$-CH=C=CH_2 (27.9 mg, 0.160 mmol), and 4-chlorobenzaldehyde (15.0 mg, 0.107 mmol) were added sequentially. The vial was sealed with a screw-cap, removed from the glove-box, and allowed to stir rapidly at ambient temperature for 20 h. Methyl *tert*-butyl ether (MTBE, 5 mL) was then added, and the mixture was filtered through a small pad of silica gel rinsing with additional MTBE (~5 mL). The combined organics were washed with 2.5% Aq. NH_4OH (2x5mL), dried with Na_2SO_4 and concentrated *in vacuo*. The crude residue was then assayed using quantitative ^{1}HNMR spectroscopy using 1,3,5-trimethoxybenzene as standard to determine the yield of **10q** and **10r** produced.

Synthetic Applications:

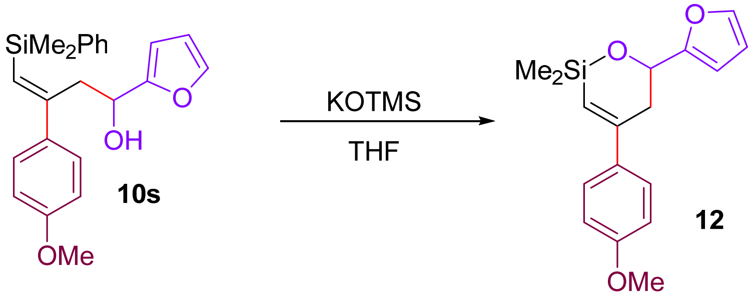

To a solution of 54.1 mg (0.143 mmol) of **10s** in 0.55 mL of THF under N_2 was added 72 µL (0.14 mmol) of a 2.0 M solution of KOTMS in THF. The resultant dark mixture was stirred at rt and monitored by TLC analysis (20% EtOAc in hexanes). After 24 h, an additional 72 µL (0.14 mmol) of a 2.0 M solution of KOTMS in THF was added and stirring was continued for an additional 24 h. To the dark mixture was added 10% Aq. NH_4Cl (2 mL) followed by extraction with MTBE (3x2mL). The combined organics were then washed with saturated brine (1x3mL), dried with anhydrous Na_2SO_4, filtered, and volatile material removed *in vacuo*. The crude residue was purified by flash chromatography on silica gel (0 – 10% EtOAc in hexanes) to provide 22.3 mg (52%) of silyl-ether **12** as a slight yellow oil. R_f = 0.29 (10% EtOAc/hexanes). ^{1}HNMR ($CDCl_3$, 600 MHz): δ 7.41 (d, J = 9.0 Hz, 2H), 7.40 (dd, J = 1.7 Hz, 0.8 Hz, 1H), 6.88 (d, J = 9.0 Hz, 2H), 6.34 (dd, J = 3.2 Hz, 1.8 Hz, 1H), 6.30 (d, J = 3.2 Hz, 1H), 6.03 (d, J = 2.2 Hz, 1H), 3.82 (s, 3H), 3.03 (ddd, J = 17 Hz, 10 Hz, 2.2 Hz, 1H), 2.85 (dd, J = 17 Hz, 2.8 Hz, 1H), 0.31 (s, 3H), 0.24 (s, 3H); $^{13}C\{^1H\}$NMR (151 MHz, $CDCl_3$): δ 159.5, 156.1, 154.5, 141.9, 135.4, 126.5, 120.8, 113.6, 110.1, 105.8, 67.5, 55.3, 36.6, –0.2, –0.4 ppm. HRMS (DART) *m/z* calcd for $C_{17}H_{21}O_3Si$ $[M + H]^+$: 301.1260; Found $[M + H]^+$: 301.1243.

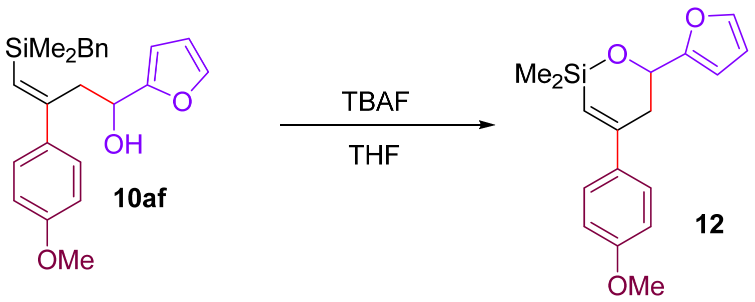

To a solution of 66.9 mg (0.170 mmol) of **10af** in 0.85 mL of THF under N_2 at rt was added 43 µL (0.043 mmol) of 1.0 M TBAF in THF. The reaction was monitored by TLC analysis (20% EtOAc in hexanes). After 10 min, the reaction was judged complete. To the mixture was added 7.5% Aq. $NaHCO_3$ (2 mL) followed by extraction with MTBE (3x2mL). The combined organics were dried with anhydrous Na_2SO_4, filtered, and concentrated *in vacuo* to provide 50.5 mg (99%) of silyl-ether **12** as a slight yellow oil that was pure by NMR spectroscopy.

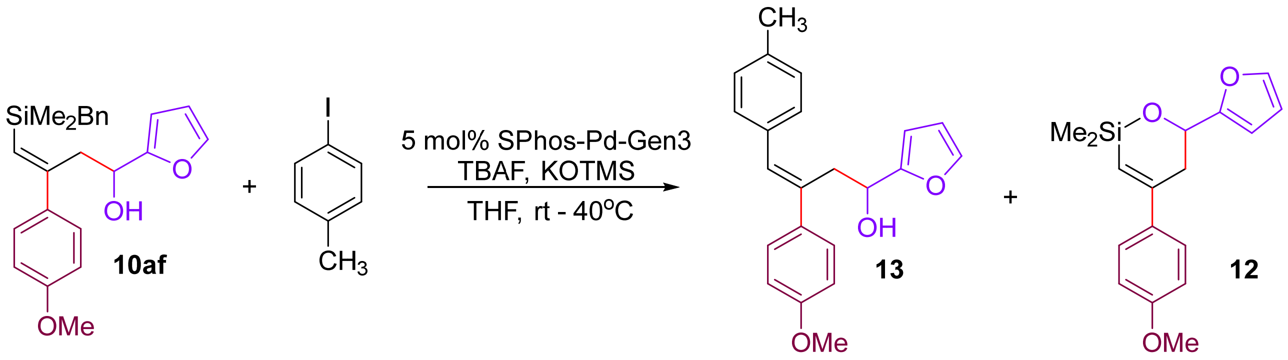

To a 2-dram screw-cap vial with stir-bar in an Ar filled glove-box was sequentially charged 50.0 mg (0.127 mmol) of **10af**, 31.9 mg (0.146 mmol) of *p*-iodotoluene, 5.0 mg (0.0064 mmol) of SPhos-Pd Gen. 3 catalyst, 0.15 mL of THF, 0.26 mL (0.26 mmol) of 1.0 M TBAF in THF, and 13 µL (0.026 mmol) of 2.0 M KOTMS in THF. The vial was sealed with a screw-cap, removed from the glove-box, and the cap further sealed to the vial using electrical tape. The mixture was then allowed to stir at rt for 1 h followed by immersing in an oil bath at 40 °C and allowing to stir at this temperature for 18 h. The reaction was then cooled to rt and diluted with 2 mL of 7.5% Aq. $NaHCO_3$ and 2 mL of MTBE. After efficiently mixing the layers, the mixture was allowed to stand for the layers to separate, and the aqueous layer was removed and extracted with MTBE (1x2mL). The organic layers were then combined and 200 mg of activated charcoal was added along with anhydrous Na_2SO_4 for drying purposes. The mixture was then filtered through celite, rinsed with additional MTBE, and concentrated *in vacuo*. Purification of the crude residue by flash chromatography on silica gel (gradient, 0 – 20% EtOAc in hexanes) afforded 11.3 mg (30%) of cyclic silyl-ether **12** which eluted first, followed by 22.2 mg (52%) of cross-coupling product **13** as a red-brown oil. R_f = 0.29 (25% EtOAc/hexanes). ^{1}HNMR ($CDCl_3$, 600 MHz): δ 7.40 (d, J = 8.8 Hz, 2H), 7.33 (dd, J = 1.8 Hz, 0.8 Hz, 1H), 7.19 (d, J = 8.0 Hz, 2H), 7.17 (d, J = 8.0 Hz, 2H), 6.79 (s, 1H), 6.28 (dd, J = 3.2 Hz, 1.9 Hz, 1H), 6.14 (d, J = 3.2 Hz, 1H), 4.76 – 4.81 (m, 1H), 3.84 (s, 3H), 3.40 (dd, J = 14 Hz, 9.1 Hz, 1H), 3.17 (dd, J = 14 Hz, 5.0 Hz, 1H), 3.36 (s, 3H), 1.89 (d, J = 4.3 Hz, 1H); $^{13}C\{^1H\}$NMR (151 MHz, $CDCl_3$): δ 159.1, 155.9, 141.9, 137.0, 136.5, 134.7, 134.6, 130.7, 129.0, 128.9, 127.8, 113.9, 110.1, 106.0, 66.2, 55.3, 36.2, 21.2 ppm. HRMS (DART) *m/z* calcd for $C_{22}H_{22}O_3$ $[M]^+$: 334.1569; Found $[M]^+$: 334.1551.

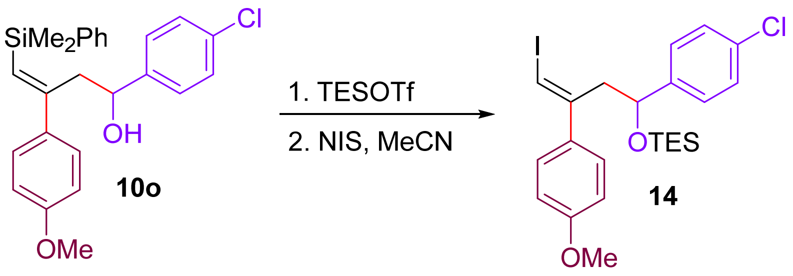

To a solution of 108 mg (0.255 mmol) of **10o** in 2.2 mL of CH_2Cl_2 at 1 – 78°C was added 59 µL (0.51 mmol) of 2,6-lutidine followed by dropwise addition of 69 µL (0.31 mmol) of TESOTf. The reaction was monitored by TLC analysis (15% EtOAc in hexanes). After 30 min, the reaction was judged complete. To the mixture was added 10% Aq. NH_4Cl (5 mL), additional 5 mL of CH_2Cl_2, and the mixture was allowed to warm to ambient temperature. The mixture was transferred to a separatory funnel to separate the layers, and the aqueous layer was further extracted with CH_2Cl_2 (2x5mL). The combined organics were dried with anhydrous Na_2SO_4, filtered, and volatile material removed *in vacuo*. The crude residue was purified by flash chromatography on silica gel (0 – 10% EtOAc in hexanes) to provide 118 mg (86%) of the protected TES-ether of **10o** (**SI-1**) as a slight yellow oil. R_f = 0.31 (5% EtOAc/hexanes). ^{1}HNMR ($CDCl_3$, 600 MHz) δ: 7.46 – 7.51 (m, 2H), 7.36 (d, *J* = 8.8 Hz, 2H), 7.30 – 7.36 (m, 3H), 7.18 (d, *J* = 8.4 Hz, 2H), 7.02 (d, *J* = 8.4 Hz, 2H), 6.88 (d, *J* = 8.8 Hz, 2H), 5.86 (s, 1H), 4.58 (dd, *J* = 7.8 Hz, 5.5 Hz, 1H), 3.84 (s, 3H), 2.98 (dd, *J* = 14 Hz, 7.8 Hz, 1H), 2.74 (dd, *J* = 14 Hz, 5.5 Hz, 1H), 0.74 (t, *J* = 8.0 Hz, 9H), 0.36 (s, 3H), 0.34 (s, 3H), 0.26 – 0.32 (m, 6H); $^{13}C\{^1H\}$NMR (151 MHz, $CDCl_3$): δ 159.2, 153.9, 144.0, 139.8, 136.0, 133.8, 132.5, 128.7, 128.0, 127.8, 127.7, 127.6, 127.2, 113.5, 72.8, 55.3, 45.9, 6.7, 4.6, –0.6, –0.7 ppm. HRMS (DART) *m/z* calcd for $C_{31}H_{42}ClO_2Si_2$ $[M + H]^+$: 537.2412; Found $[M + H]^+$: 537.2442.

To a solution of 36.8 mg (0.0685 mmol) of the TES-ether of **10o** prepared above in 0.35 mL of MeCN was added 23.1 mg (0.103 mmol) of *N*-iodosuccinimide, and the mixture was allowed to stir at rt and monitored by HPLC. After 1h, 0.25 mL of 10% Aq. $Na_2S_2O_3$ and 2 mL of 7.5% Aq. $NaHCO_3$ were added and the mixture was extracted with MTBE (3x2mL). The combined organics were then washed with water (2x2mL), dried with anhydrous Na_2SO_4, filtered, and concentrated *in vacuo*. The crude residue was purified by flash chromatography on silica gel (0 – 5% EtOAc in hexanes) to provide 31.5 mg (87%) of vinyl iodide **14** as a slight yellow oil. R_f = 0.32 (5% EtOAc/hexanes). ^{1}HNMR ($CDCl_3$, 600 MHz) δ: 7.19 – 7.26 (m, 6H), 6.83 (d, *J* = 8.8 Hz, 2H), 6.42 (s, 1H), 4.84 (dd, *J* = 7.5 Hz, 6.2 Hz, 1H), 3.82 (s, 3H), 3.07 (dd, *J* = 14 Hz, 7.5 Hz, 1H), 2.94 (dd, *J* = 14 Hz, 6.2 Hz, 1H), 0.79 (t, *J* = 7.9 Hz, 9H), 0.37 – 0.45 (m, 6H); $^{13}C\{^1H\}$NMR (151 MHz, $CDCl_3$): δ 159.3, 147.6, 143.2, 133.6, 132.8, 128.1, 127.8, 127.4, 113.7, 79.5, 72.6, 55.3, 48.0, 6.7, 4.7 ppm. HRMS (DART) *m/z* calcd for $C_{23}H_{31}ClIO_2Si$ $[M + H]^+$: 529.0827; Found $[M + H]^+$: 529.0857.

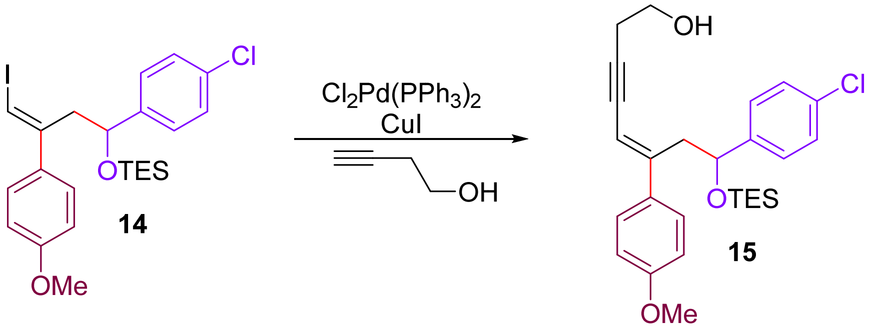

To 29.0 mg (0.0548 mmol) of vinyl iodide **14** in a 1-dram vial with stir-bar in an Ar-filled glove-box was sequentially charged 1.9 mg (0.0027 mmol) of $(PPh_3)_2PdCl_2$, 2.1 mg (0.011 mmol) of CuI, 12 µL (0.16 mmol) of but-3-yn-1-ol, 0.28 mL of toluene, and 61 µL (0.44 mmol) of triethylamine. The vial was then sealed with a screw-cap, removed from the glove-box, and allowed to stir at rt for 24 h. To the mixture was then added 10% Aq. NH_4Cl (1 mL) and the mixture was extracted with MTBE (3x1mL). The combined organics were then dried with anhydrous Na_2SO_4, filtered, and concentrated *in vacuo*. The crude residue was purified by flash chromatography on silica gel (0 – 20% EtOAc in hexanes) to provide 19.0 mg (74%) of enyne **15** as a yellow oil. R_f = 0.19 (20% EtOAc/hexanes). ^{1}HNMR ($CDCl_3$, 600 MHz) δ: 7.33 (d, J = 8.9 Hz, 2H), 7.24 (d, J = 8.5 Hz, 2H), 7.21 (d, J = 8.5 Hz, 2H), 6.87 (d, J = 8.9 Hz, 2H), 5.73 (t, J = 2.2 Hz, 1H), 4.78 (dd, J = 7.6 Hz, 5.8 Hz, 1H), 3.83 (s, 3H), 3.76 (t, J = 6.1 Hz 2H), 3.15 (dd, J = 14 Hz, 7.6 Hz, 1H), 3.00 (dd, J = 14 Hz, 5.8 Hz, 1H), 2.59 – 2.69 (m, 2H), 2.03 (br s, 1H), 0.79 (t, J = 8.0 Hz, 9H), 0.37 – 0.44 (m, 6H); ^{13}C{^{1}H}NMR (151 MHz, $CDCl_3$): δ 159.5, 148.4, 143.6, 132.7, 132.6, 128.0, 127.3, 127.2, 113.8, 107.4, 91.4, 81.3, 73.4, 61.2, 55.3, 43.7, 24.2, 6.6, 4.6 ppm. HRMS (DART) *m/z* calcd for $C_{27}H_{36}ClO_3Si$ $[M + H]^+$: 471.2122; Found $[M + H]^+$: 471.2134.

NMR Data:

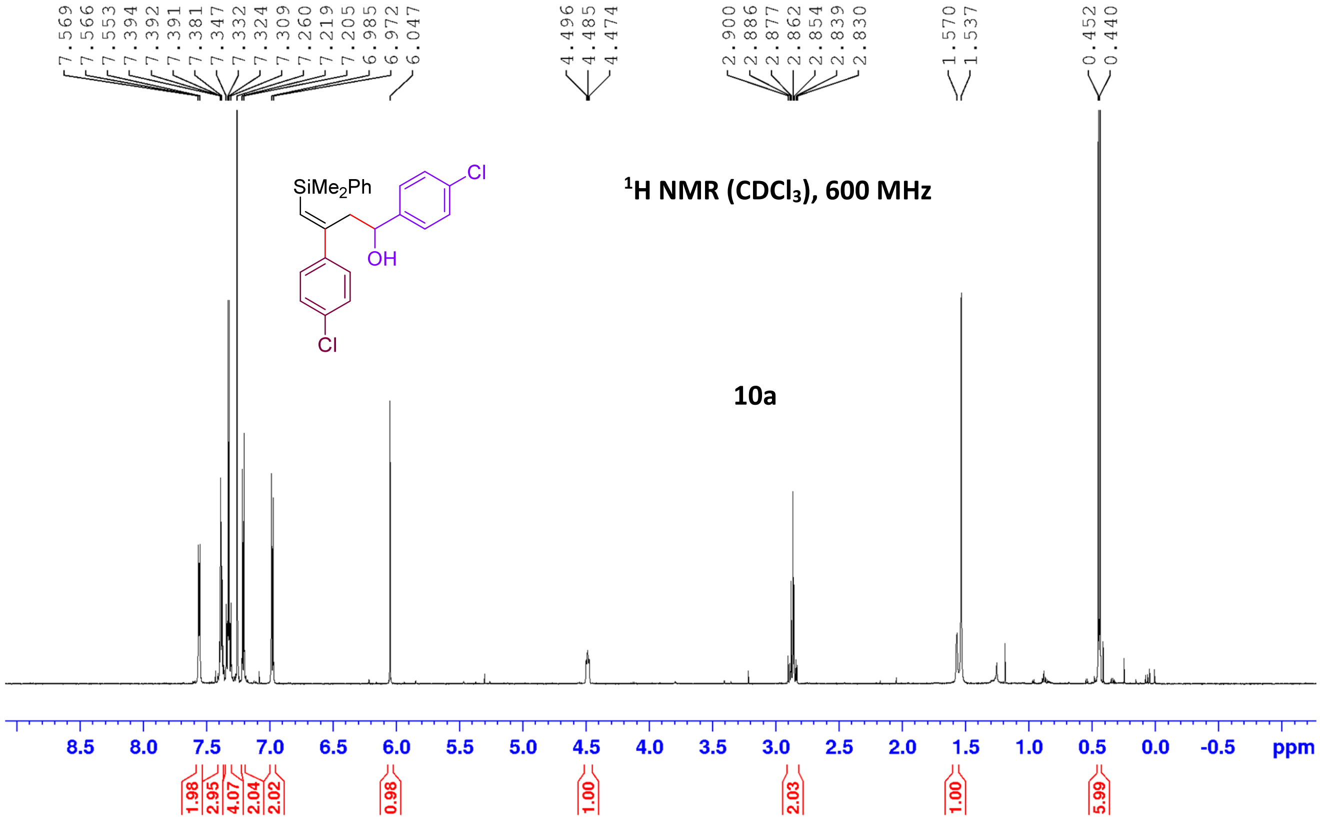

7.569 7.566 7.553 7.394 7.392 7.391 7.381 7.347 7.332 7.324 7.309 7.260 7.219 7.205 6.985 6.972 6.047
4.496 4.485 4.474
2.900 2.886 2.877 2.862 2.854 2.839 2.830
1.570 1.537
0.452 0.440
SiMe2Ph
Cl
OH
Cl
1H NMR (CDCl3), 600 MHz
10a
8.5 8.0 7.5 7.0 6.5 6.0 5.5 5.0 4.5 4.0 3.5 3.0 2.5 2.0 1.5 1.0 0.5 0.0 -0.5 ppm
1.98 2.95 4.07 2.04 2.02 0.98 1.00 2.03 1.00 5.99

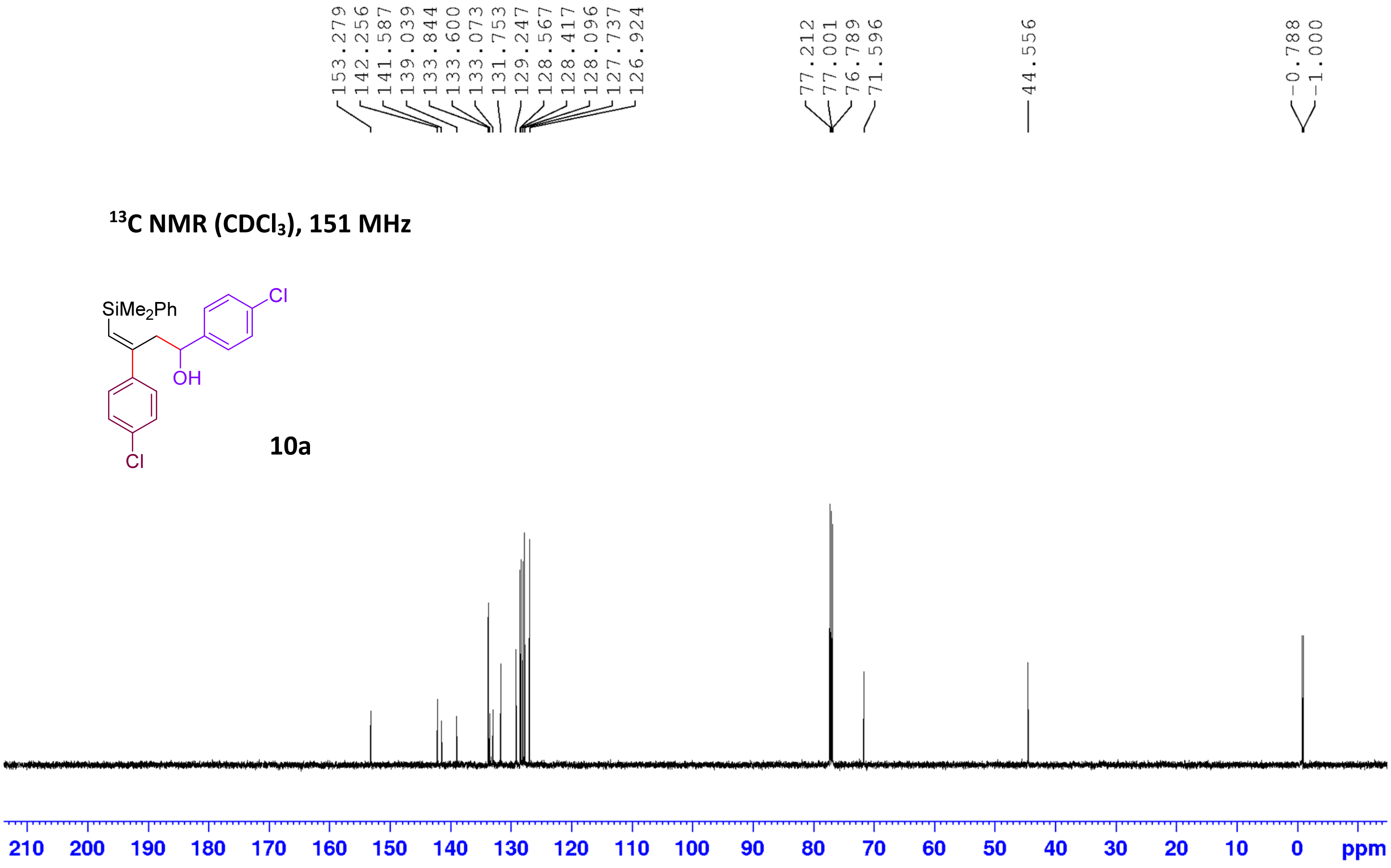

153.279 142.256 141.587 139.039 133.844 133.600 133.073 131.753 129.247 128.567 128.417 128.096 127.737 126.924
77.212 77.001 76.789 71.596
44.556
-0.788 -1.000
13C NMR (CDCl3), 151 MHz
SiMe2Ph
Cl
OH
Cl
10a
210 200 190 180 170 160 150 140 130 120 110 100 90 80 70 60 50 40 30 20 10 0 ppm

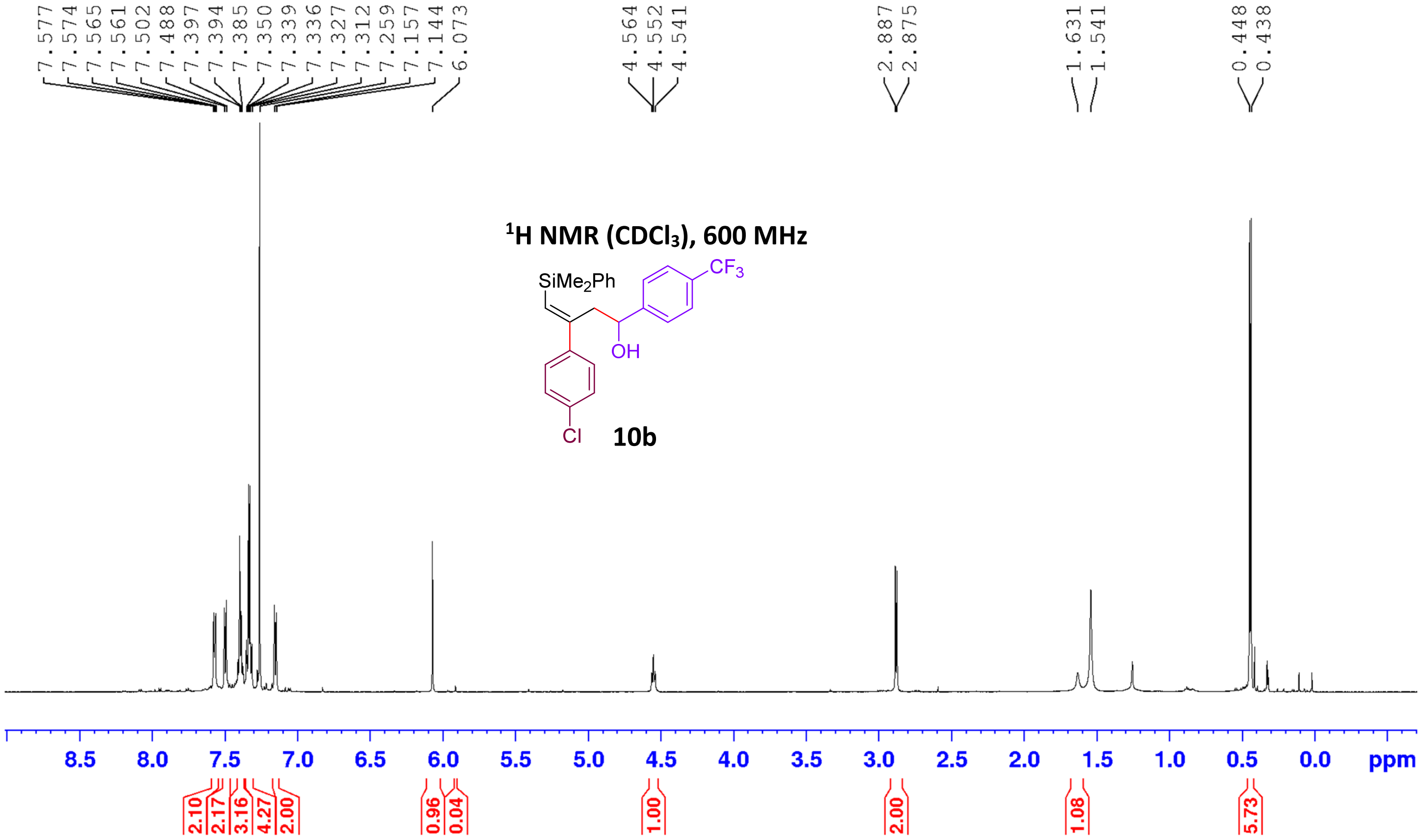

7.577
7.574
7.565
7.561
7.502
7.488
7.397
7.394
7.385
7.350
7.339
7.336
7.327
7.312
7.259
7.157
7.144
6.073
4.564
4.552
4.541
2.887
2.875
1.631
1.541
0.448
0.438
1H NMR (CDCl3), 600 MHz
SiMe2Ph
CF3
OH
Cl
10b
8.5
8.0
7.5
7.0
6.5
6.0
5.5
5.0
4.5
4.0
3.5
3.0
2.5
2.0
1.5
1.0
0.5
0.0
ppm
2.10
2.17
3.16
4.27
2.00
0.96
0.04
1.00
2.00
1.08
5.73

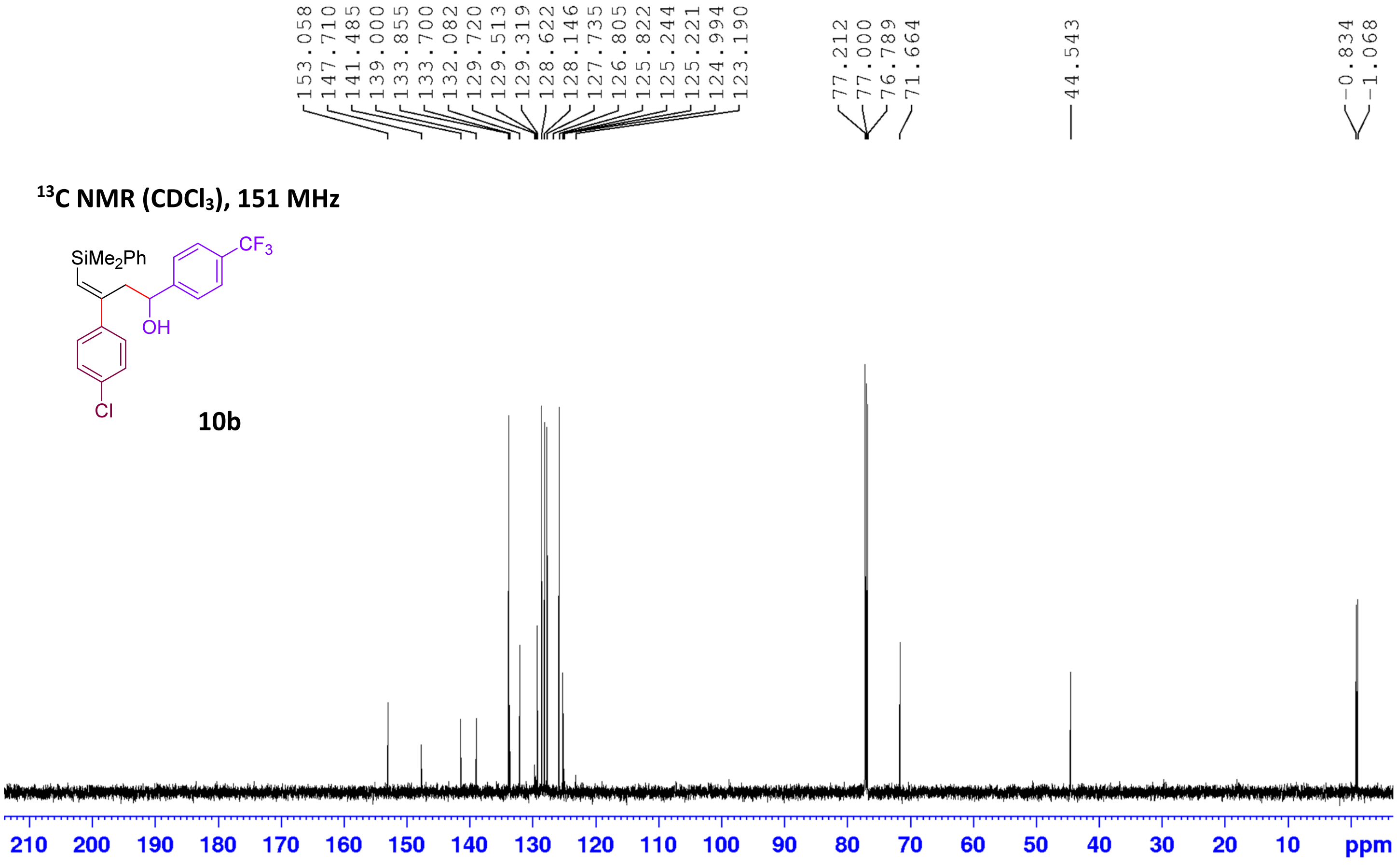

153.058
147.710
141.485
139.000
133.855
133.700
132.082
129.720
129.513
129.319
128.622
128.146
127.735
126.805
125.822
125.244
125.221
124.994
123.190
77.212
77.000
76.789
71.664
44.543
-0.834
-1.068
13C NMR (CDCl3), 151 MHz
SiMe2Ph
CF3
OH
Cl
10b
210
200
190
180
170
160
150
140
130
120
110
100
90
80
70
60
50
40
30
20
10
ppm

^{1}H NMR ($CDCl_3$), 600 MHz

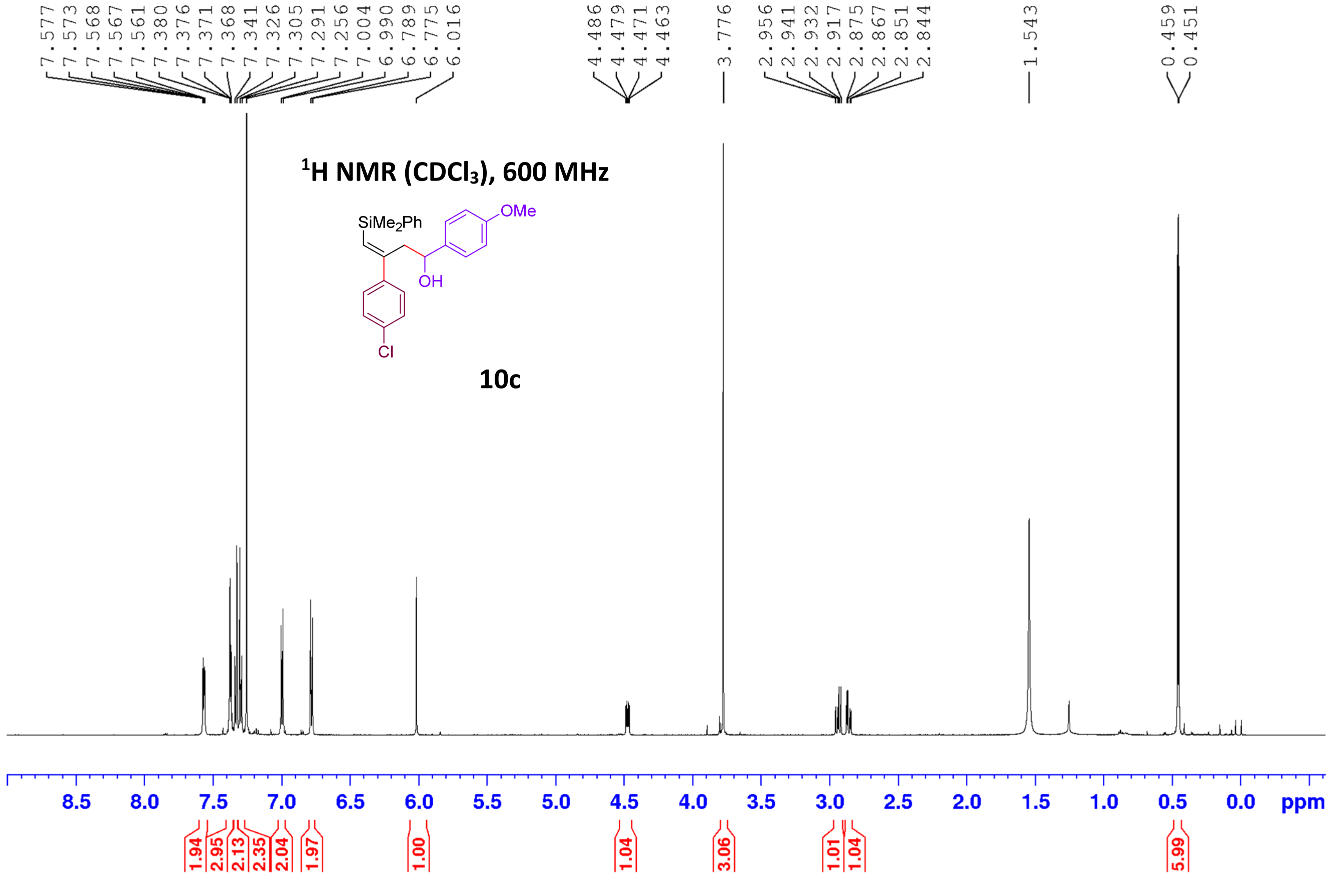

10c

^{13}C NMR ($CDCl_3$), 151 MHz

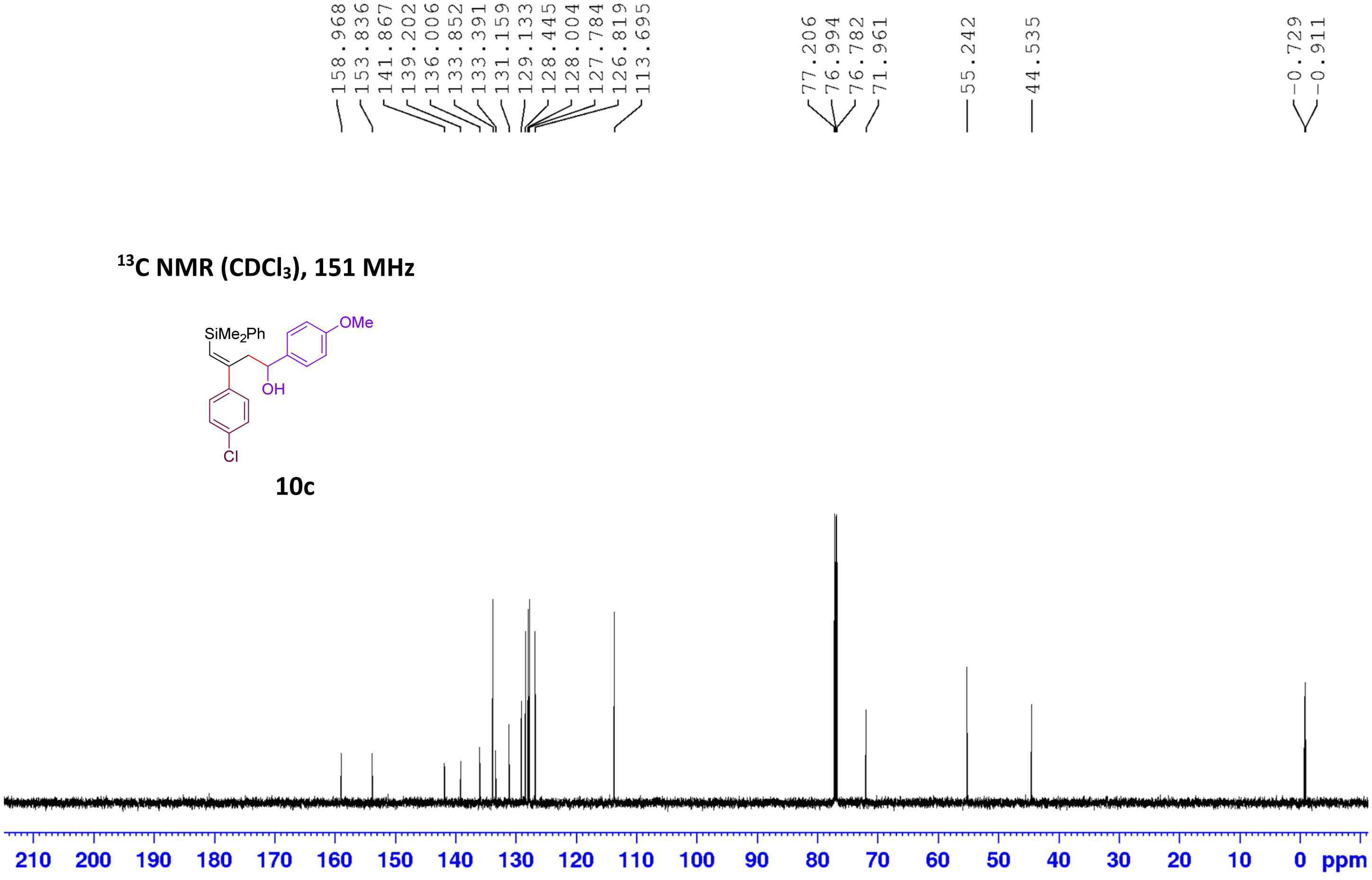

10c

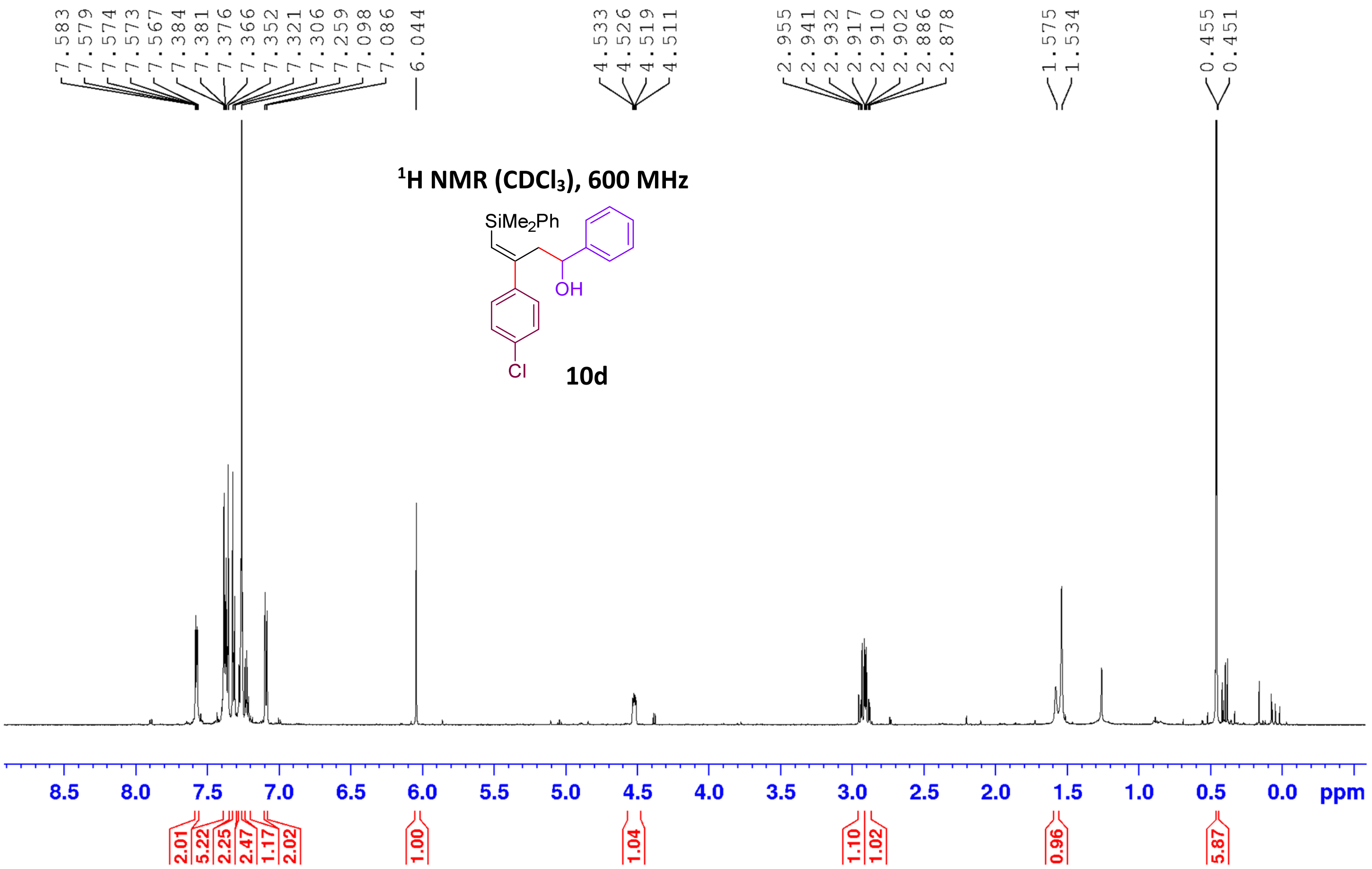

7.583
7.579
7.574
7.573
7.567
7.384
7.381
7.376
7.366
7.352
7.321
7.306
7.259
7.098
7.086
6.044
4.533
4.526
4.519
4.511
2.955
2.941
2.932
2.917
2.910
2.902
2.886
2.878
1.575
1.534
0.455
0.451
¹H NMR (CDCl₃), 600 MHz
SiMe₂Ph
OH
Cl
10d
8.5
8.0
7.5
7.0
6.5
6.0
5.5
5.0
4.5
4.0
3.5
3.0
2.5
2.0
1.5
1.0
0.5
0.0
ppm
2.01
5.22
2.25
2.47
1.17
2.02
1.00
1.04
1.10
1.02
0.96
5.87

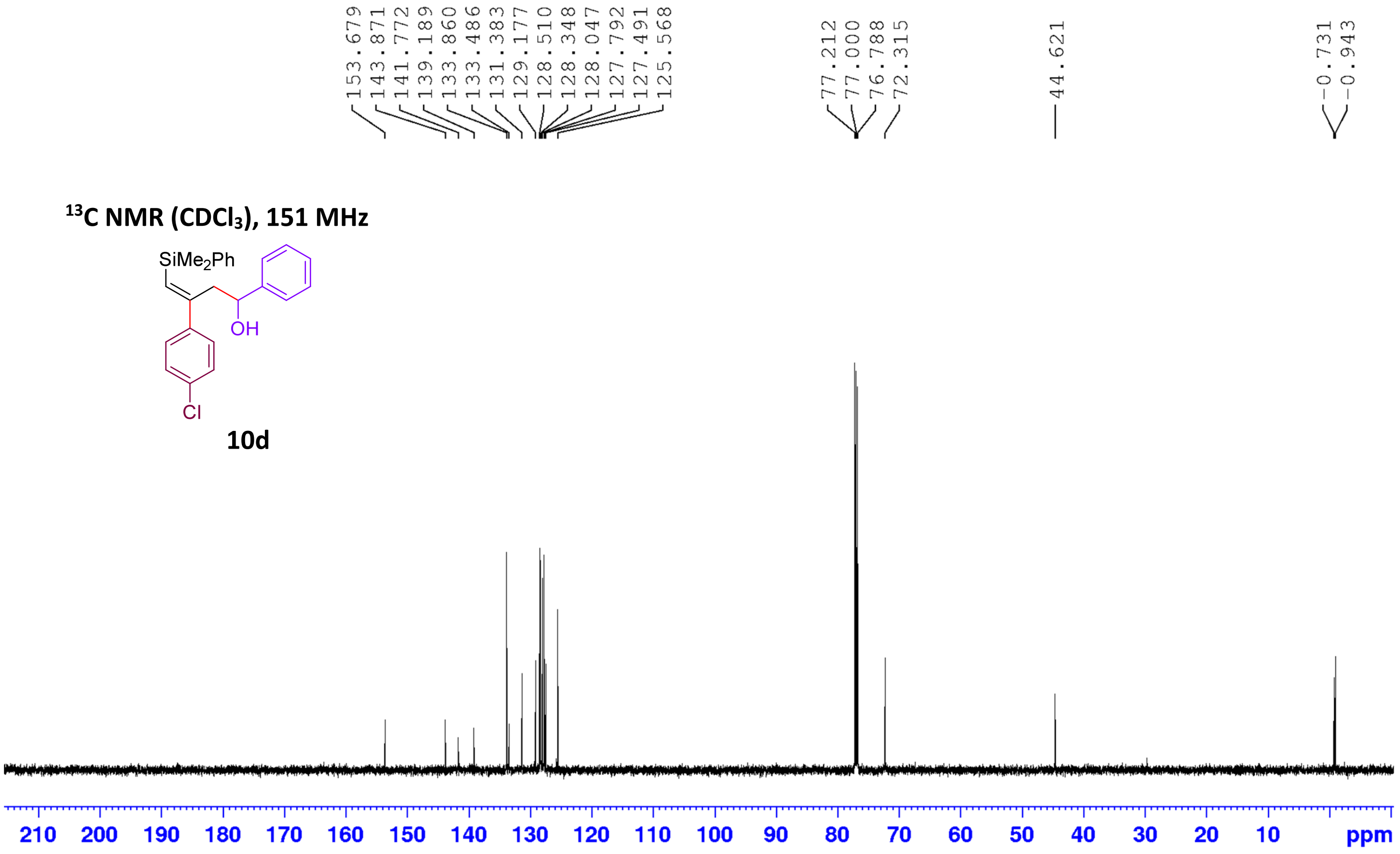

153.679
143.871
141.772
139.189
133.860
133.486
131.383
129.177
128.510
128.348
128.047
127.792
127.491
125.568
77.212
77.000
76.788
72.315
44.621
-0.731
-0.943
¹³C NMR (CDCl₃), 151 MHz
SiMe₂Ph
OH
Cl
10d
210
200
190
180
170
160
150
140
130
120
110
100
90
80
70
60
50
40
30
20
10
ppm

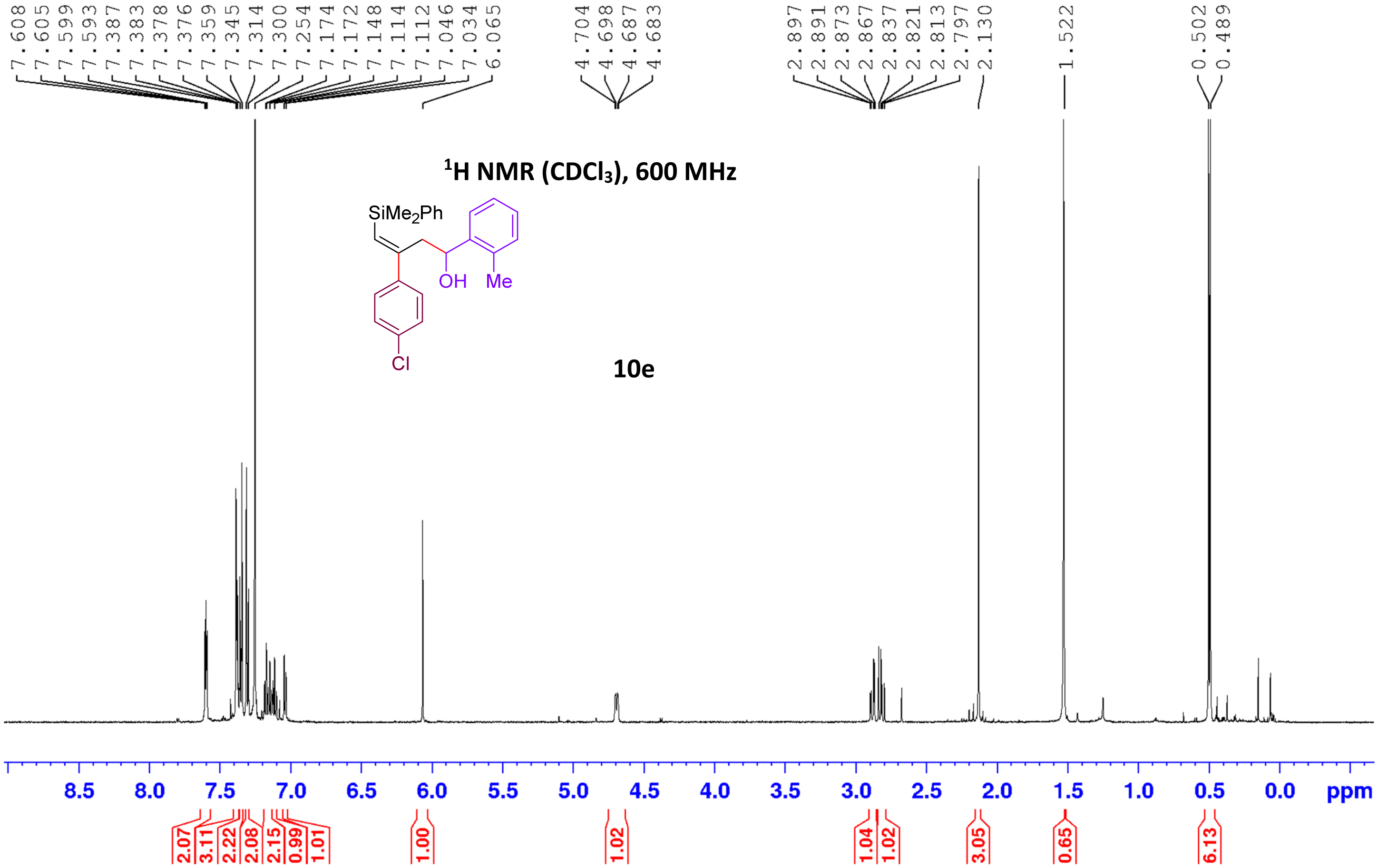
7.608 7.605 7.599 7.593 7.387 7.383 7.378 7.376 7.359 7.345 7.314 7.300 7.254 7.174 7.172 7.148 7.114 7.112 7.046 7.034 6.065
4.704 4.698 4.687 4.683
2.897 2.891 2.873 2.867 2.837 2.821 2.813 2.797 2.130
1.522
0.502 0.489
1H NMR (CDCl3), 600 MHz
SiMe2Ph
OH
Me
Cl
10e
8.5 8.0 7.5 7.0 6.5 6.0 5.5 5.0 4.5 4.0 3.5 3.0 2.5 2.0 1.5 1.0 0.5 0.0 ppm
2.07 3.11 2.22 2.08 2.15 0.99 1.01 1.00 1.02 1.04 1.02 3.05 0.65 6.13

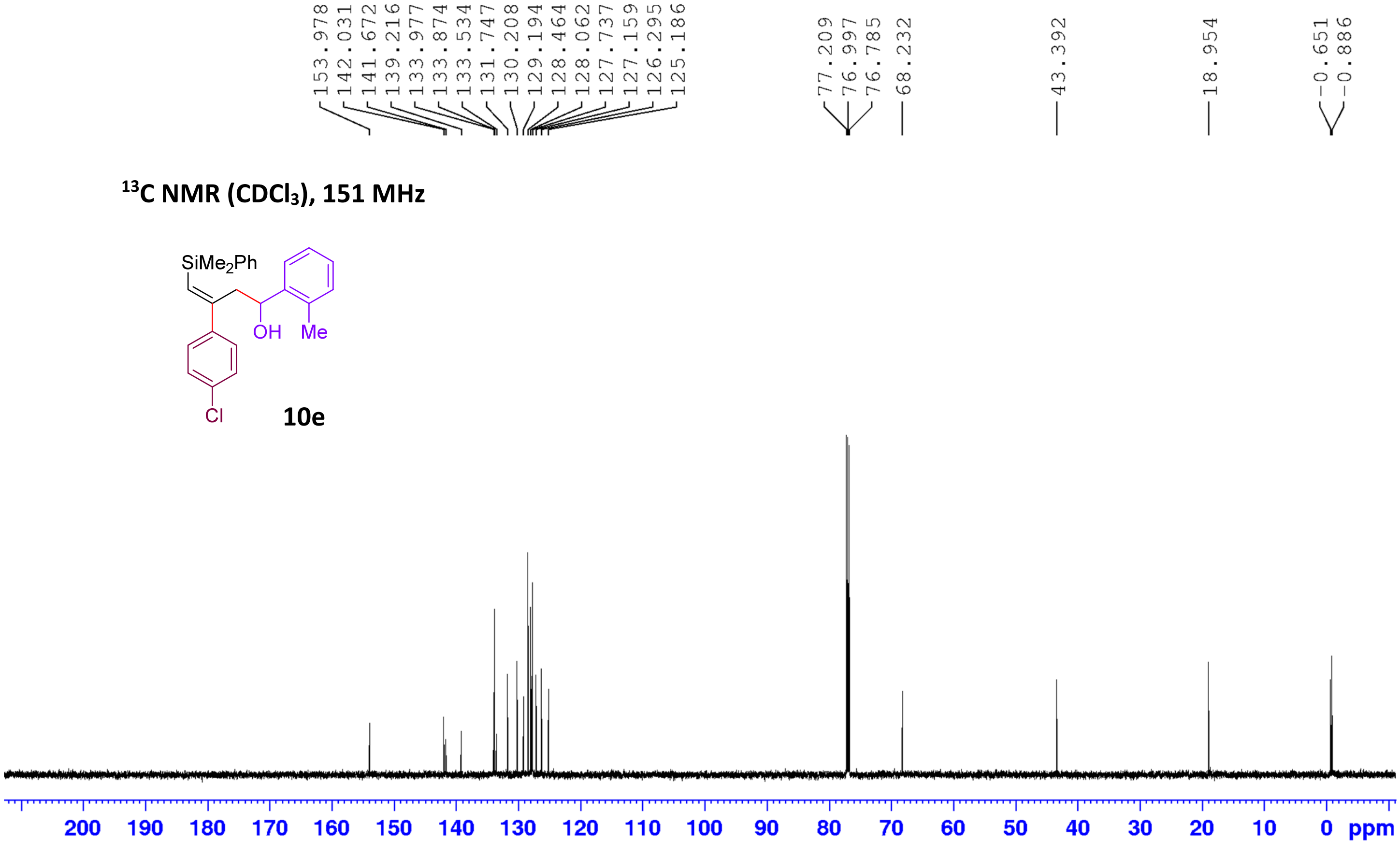
153.978 142.031 141.672 139.216 133.977 133.874 133.534 131.747 130.208 129.194 128.464 128.062 127.737 127.159 126.295 125.186
77.209 76.997 76.785 68.232
43.392
18.954
-0.651 -0.886
13C NMR (CDCl3), 151 MHz
SiMe2Ph
OH
Me
Cl
10e
200 190 180 170 160 150 140 130 120 110 100 90 80 70 60 50 40 30 20 10 0 ppm

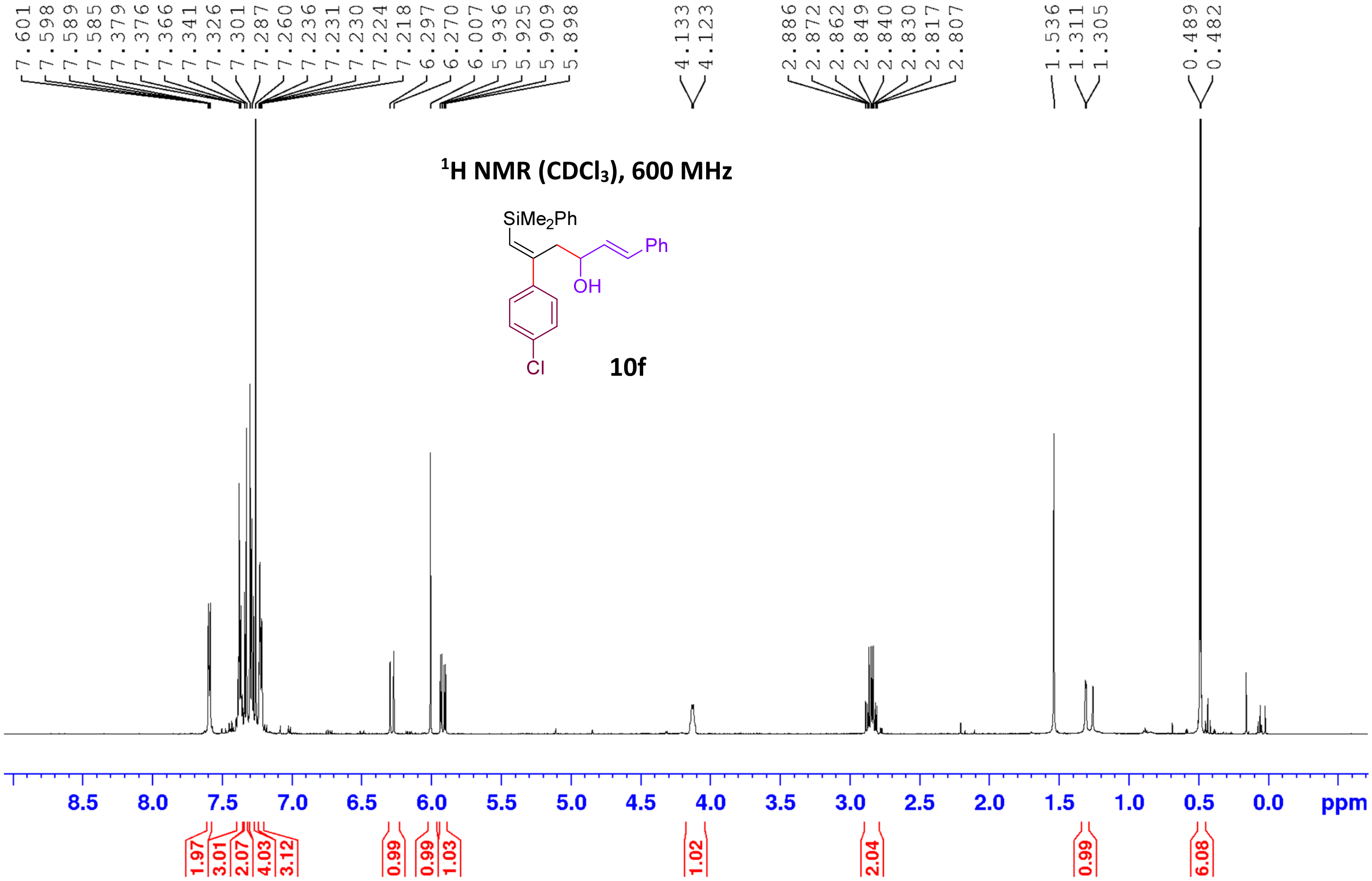
7.601 7.598 7.589 7.585 7.379 7.376 7.366 7.341 7.326 7.301 7.287 7.260 7.236 7.231 7.230 7.224 7.218 6.297 6.270 6.007 5.936 5.925 5.909 5.898
4.133 4.123
2.886 2.872 2.862 2.849 2.840 2.830 2.817 2.807
1.536 1.311 1.305
0.489 0.482
1H NMR (CDCl3), 600 MHz
SiMe2Ph
Ph
OH
Cl
10f
8.5 8.0 7.5 7.0 6.5 6.0 5.5 5.0 4.5 4.0 3.5 3.0 2.5 2.0 1.5 1.0 0.5 0.0 ppm
1.97 3.01 2.07 4.03 3.12 0.99 0.99 1.03 1.02 2.04 0.99 6.08

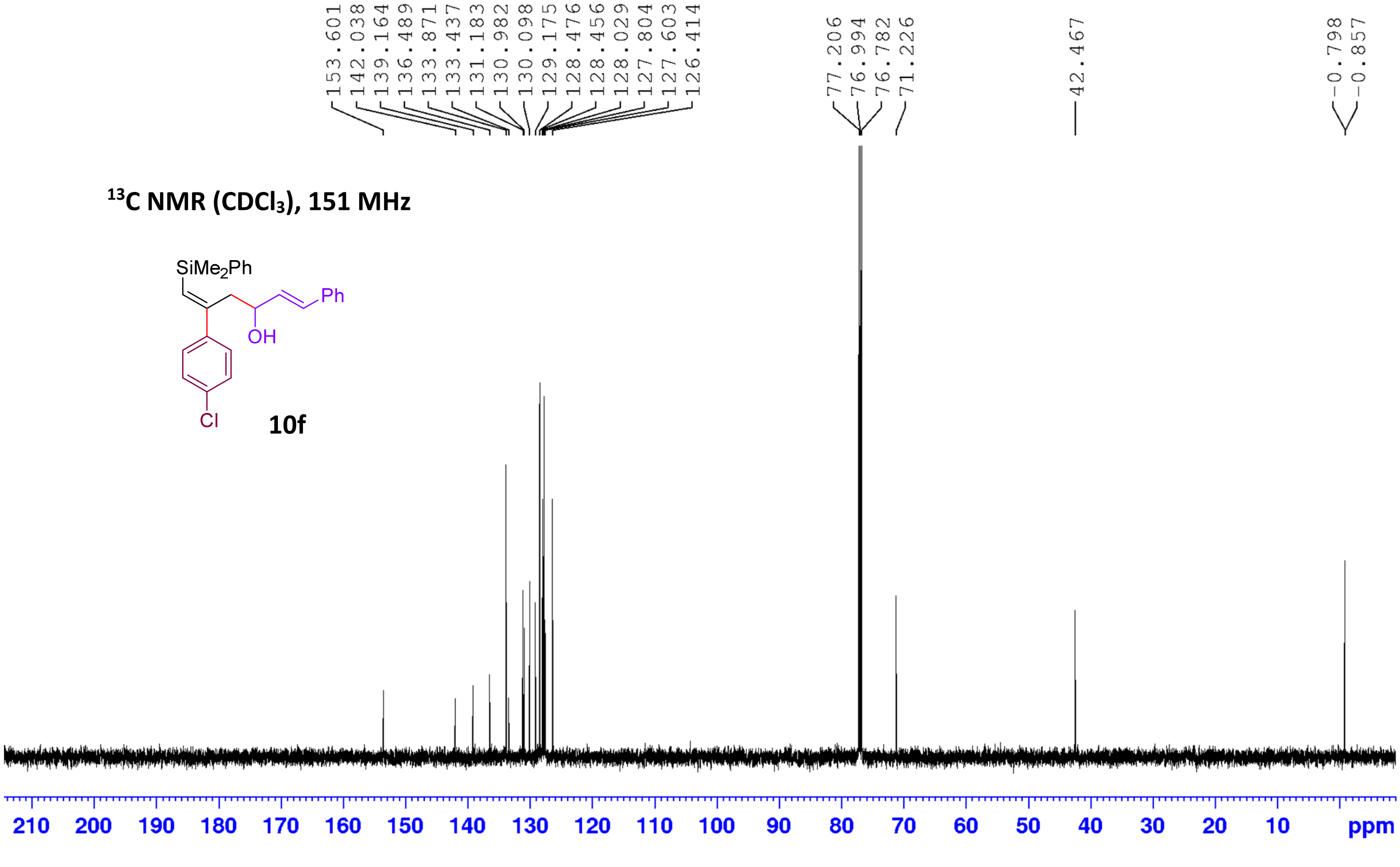
153.601 142.038 139.164 136.489 133.871 133.437 131.183 130.982 130.098 129.175 128.476 128.456 128.029 127.804 127.603 126.414
77.206 76.994 76.782 71.226
42.467
-0.798 -0.857
13C NMR (CDCl3), 151 MHz
SiMe2Ph
Ph
OH
Cl
10f
210 200 190 180 170 160 150 140 130 120 110 100 90 80 70 60 50 40 30 20 10 ppm

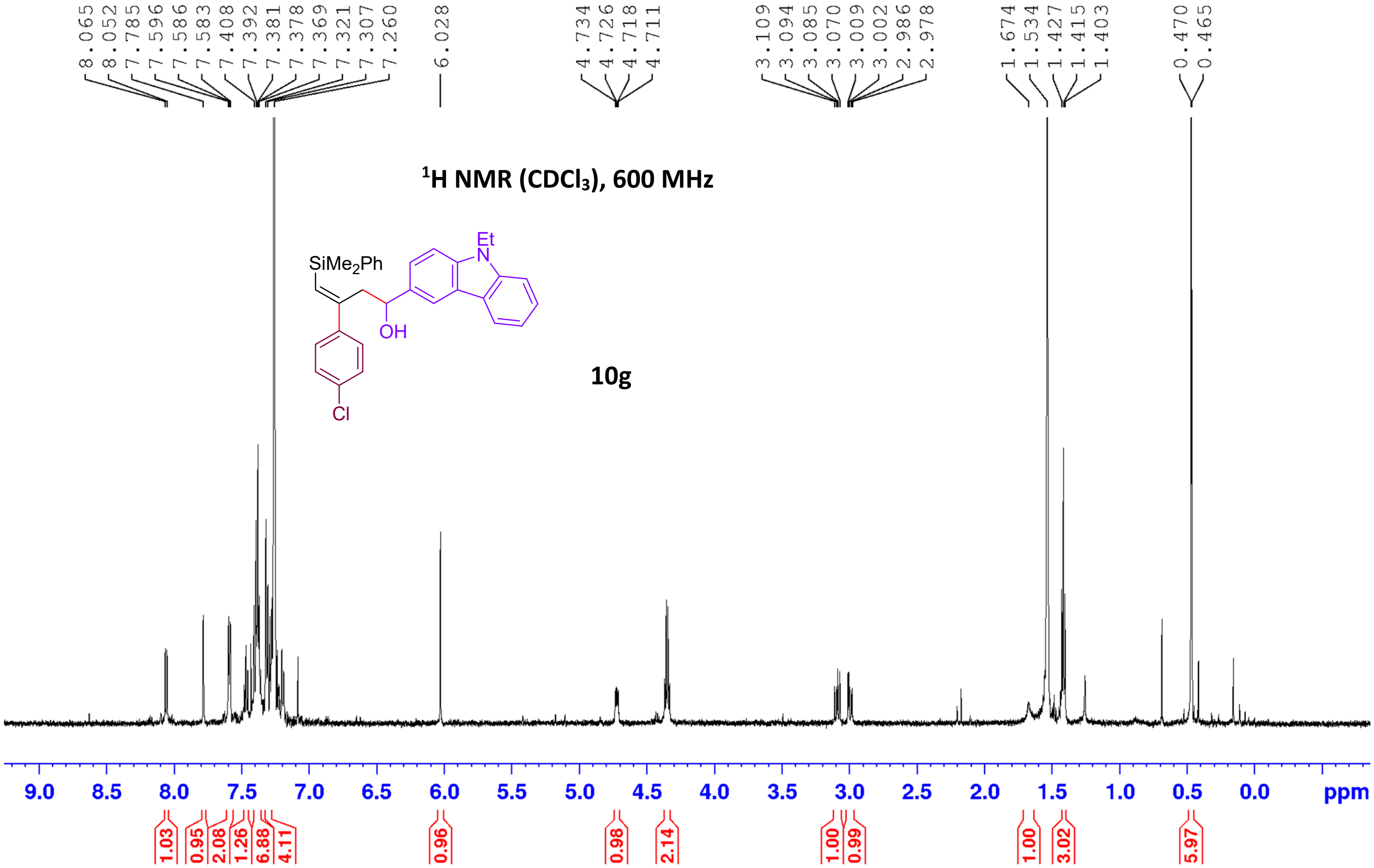

8.065 8.052 7.785 7.596 7.586 7.583 7.408 7.392 7.381 7.378 7.369 7.321 7.307 7.260
6.028
4.734 4.726 4.718 4.711
3.109 3.094 3.085 3.070 3.009 3.002 2.986 2.978
1.674 1.534 1.427 1.415 1.403
0.470 0.465
^{1}H NMR (CDCl3), 600 MHz
SiMe2Ph
Et
N
OH
Cl
10g
9.0 8.5 8.0 7.5 7.0 6.5 6.0 5.5 5.0 4.5 4.0 3.5 3.0 2.5 2.0 1.5 1.0 0.5 0.0 ppm
1.03 0.95 2.08 1.26 6.88 4.11 0.96 0.98 2.14 1.00 0.99 1.00 3.02 5.97

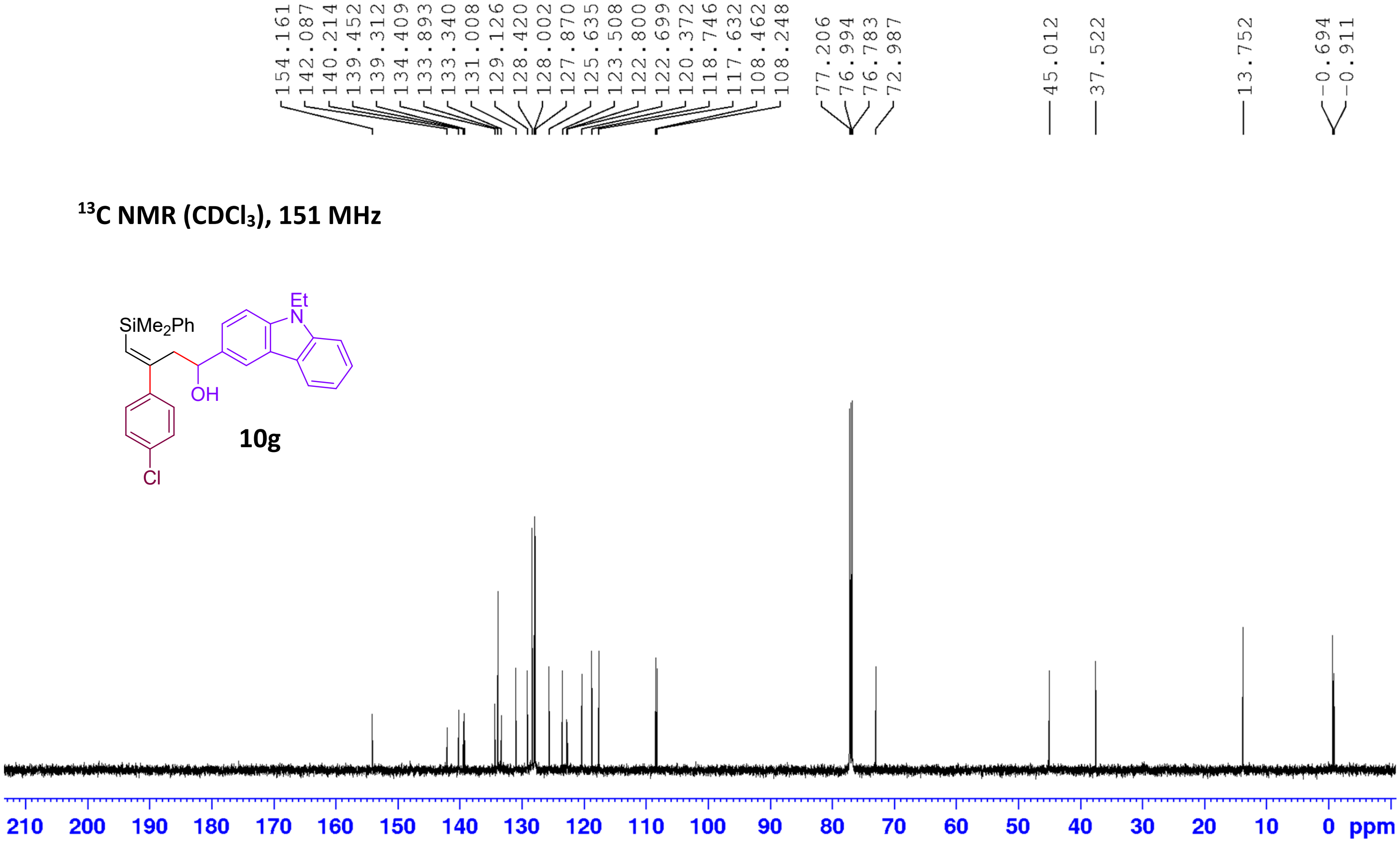

154.161 142.087 140.214 139.452 139.312 134.409 133.893 133.340 131.008 129.126 128.420 128.002 127.870 125.635 123.508 122.800 122.699 120.372 118.746 117.632 108.462 108.248
77.206 76.994 76.783 72.987
45.012 37.522
13.752
-0.694 -0.911
^{13}C NMR (CDCl3), 151 MHz
SiMe2Ph
Et
N
OH
Cl
10g
210 200 190 180 170 160 150 140 130 120 110 100 90 80 70 60 50 40 30 20 10 0 ppm

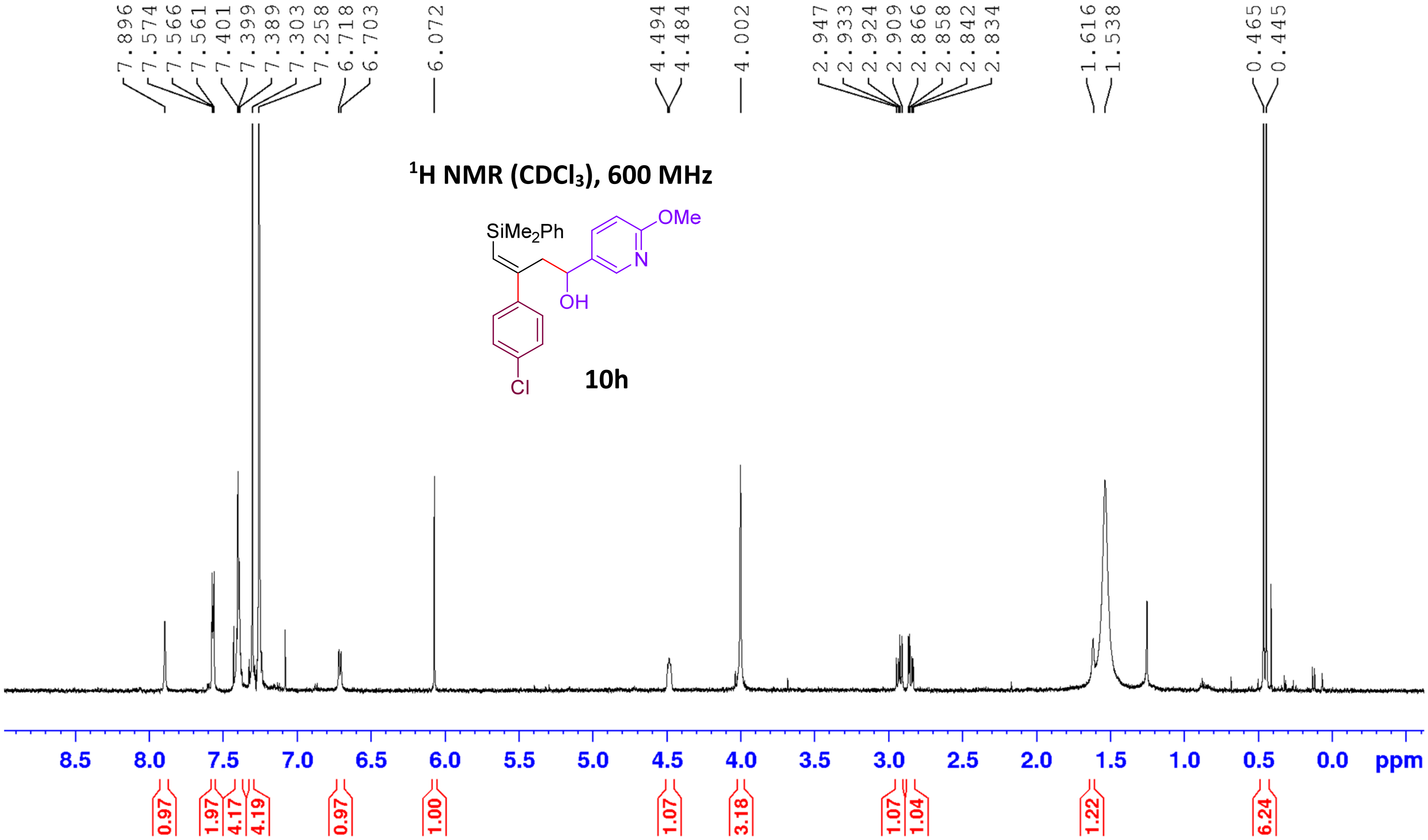

7.896 7.574 7.566 7.561 7.401 7.399 7.389 7.303 7.258 6.718 6.703 6.072 4.494 4.484 4.002 2.947 2.933 2.924 2.909 2.866 2.858 2.842 2.834 1.616 1.538 0.465 0.445
^{1}H NMR (CDCl$_3$), 600 MHz
SiMe$_2$Ph
OMe
N
OH
Cl
10h
8.5 8.0 7.5 7.0 6.5 6.0 5.5 5.0 4.5 4.0 3.5 3.0 2.5 2.0 1.5 1.0 0.5 0.0 ppm
0.97 1.97 4.17 4.19 0.97 1.00 1.07 3.18 1.07 1.04 1.22 6.24

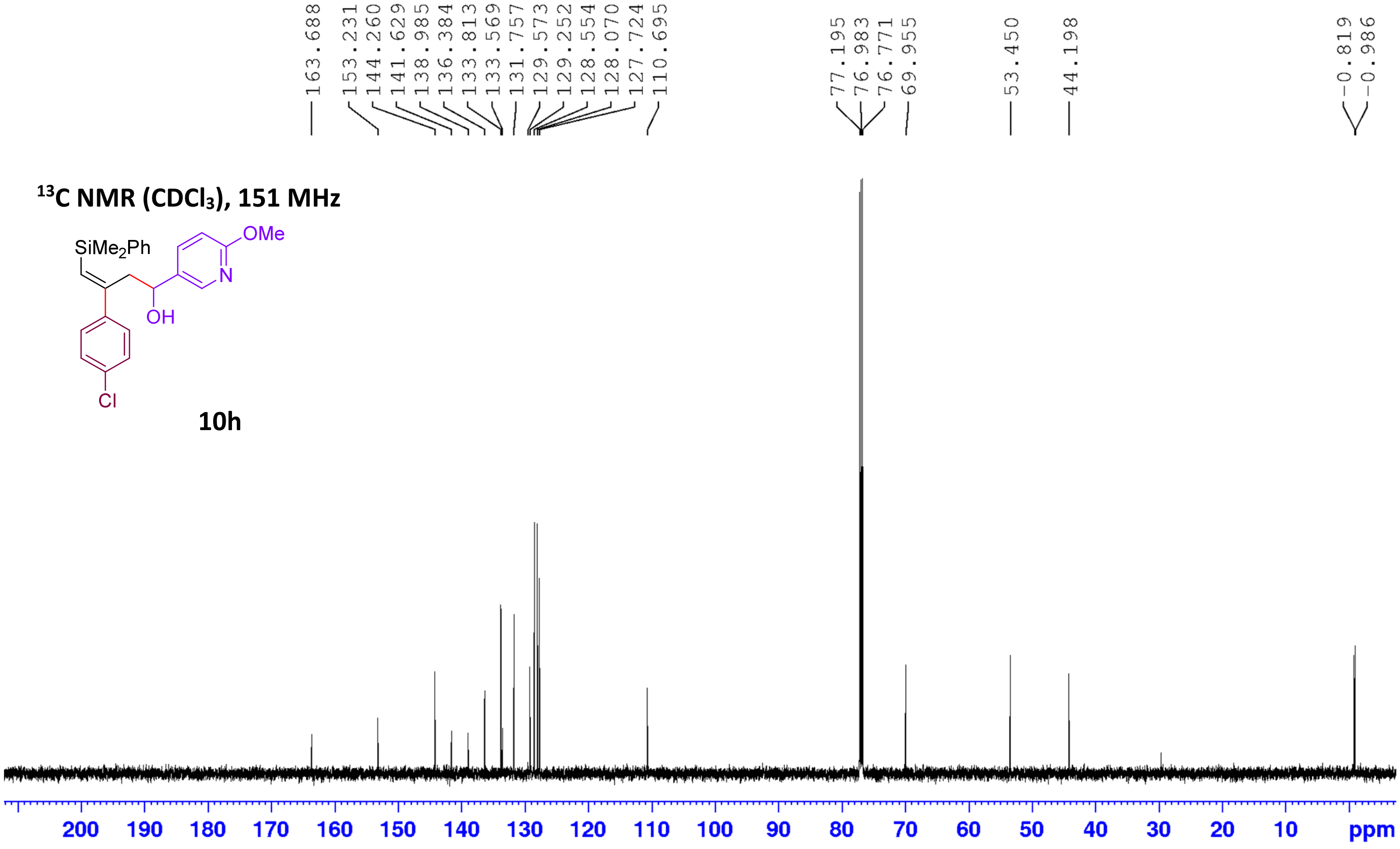

163.688 153.231 144.260 141.629 138.985 136.384 133.813 133.569 131.757 129.573 129.252 128.554 128.070 127.724 110.695 77.195 76.983 76.771 69.955 53.450 44.198 -0.819 -0.986
^{13}C NMR (CDCl$_3$), 151 MHz
SiMe$_2$Ph
OMe
N
OH
Cl
10h
200 190 180 170 160 150 140 130 120 110 100 90 80 70 60 50 40 30 20 10 ppm

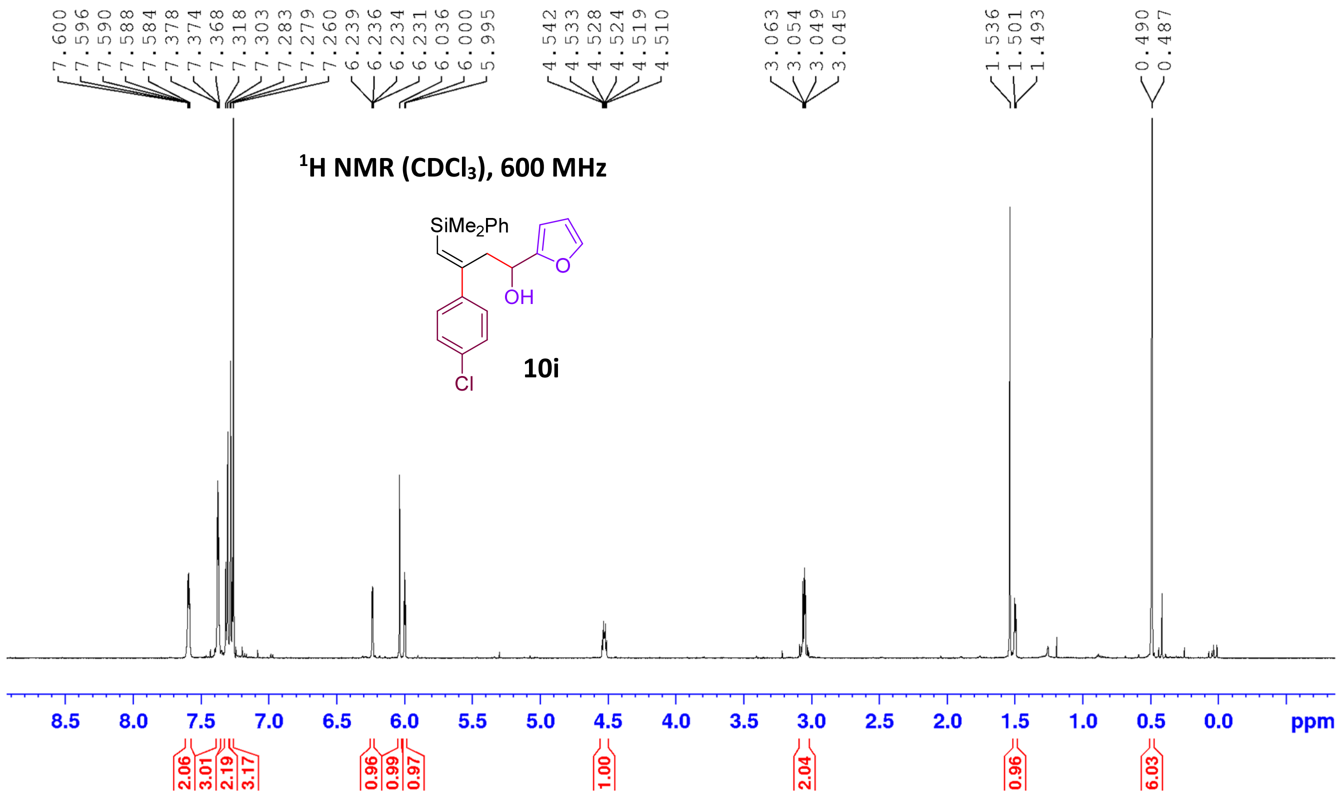
7.600
7.596
7.590
7.588
7.584
7.378
7.374
7.368
7.318
7.303
7.283
7.279
7.260
6.239
6.236
6.234
6.231
6.036
6.000
5.995
4.542
4.533
4.528
4.524
4.519
4.510
3.063
3.054
3.049
3.045
1.536
1.501
1.493
0.490
0.487
¹H NMR (CDCl₃), 600 MHz
SiMe₂Ph
OH
O
Cl
10i
8.5
8.0
7.5
7.0
6.5
6.0
5.5
5.0
4.5
4.0
3.5
3.0
2.5
2.0
1.5
1.0
0.5
0.0
ppm
2.06
3.01
2.19
3.17
0.96
0.99
0.97
1.00
2.04
0.96
6.03

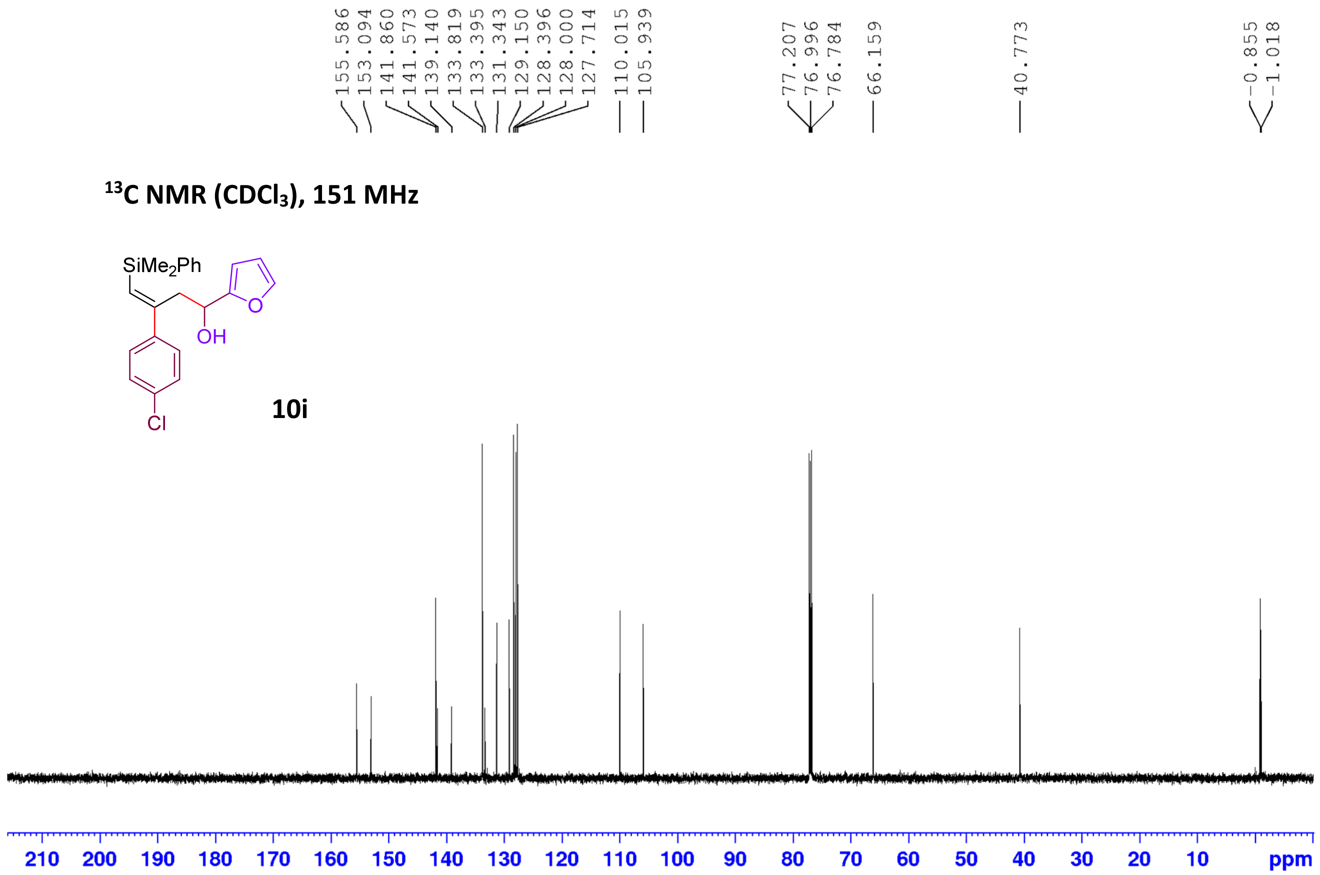
155.586
153.094
141.860
141.573
139.140
133.819
133.395
131.343
129.150
128.396
128.000
127.714
110.015
105.939
77.207
76.996
76.784
66.159
40.773
-0.855
-1.018
¹³C NMR (CDCl₃), 151 MHz
SiMe₂Ph
OH
O
Cl
10i
210
200
190
180
170
160
150
140
130
120
110
100
90
80
70
60
50
40
30
20
10
ppm

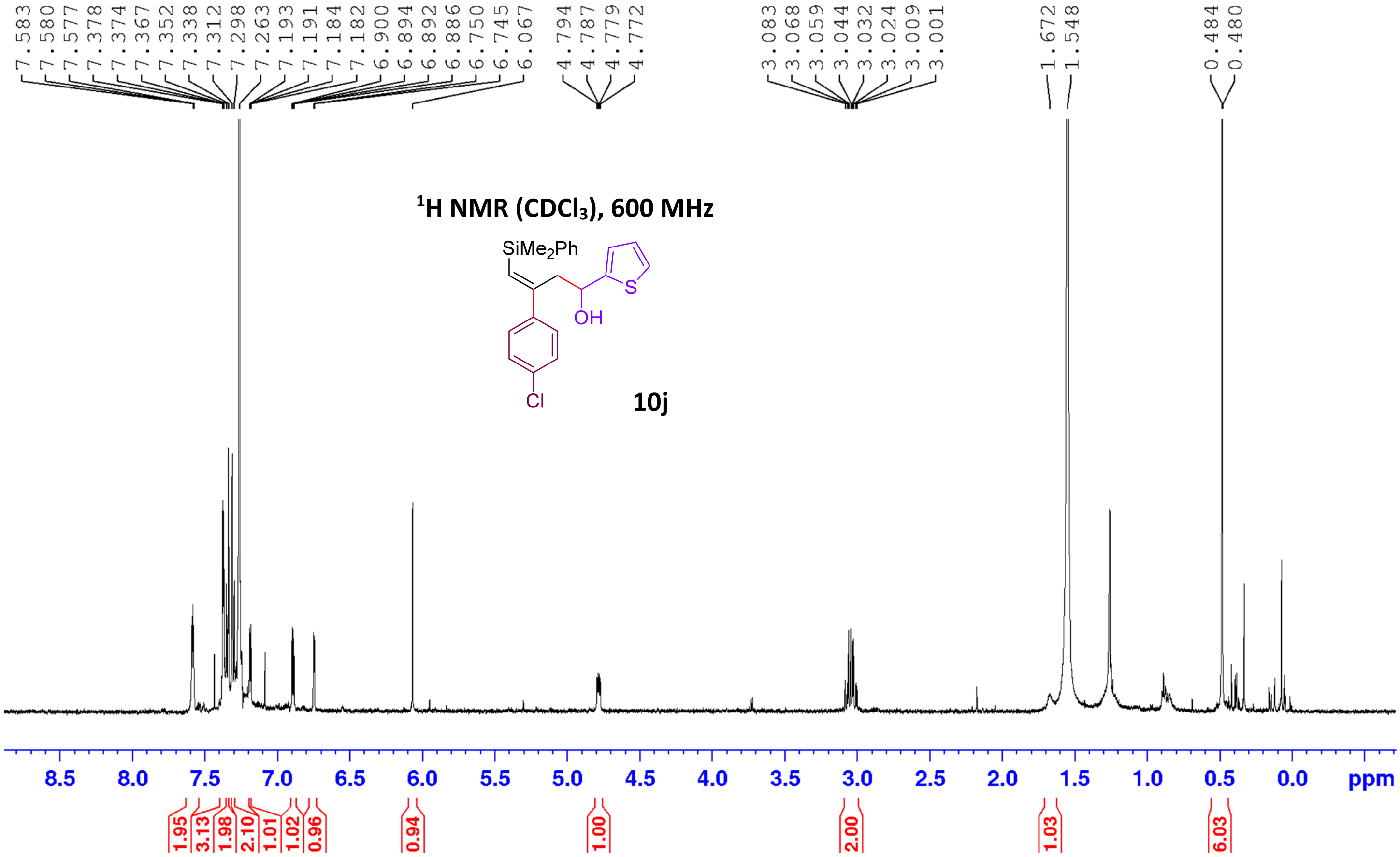

7.583 7.580 7.577 7.378 7.374 7.367 7.352 7.338 7.312 7.298 7.263 7.193 7.191 7.184 7.182 6.900 6.894 6.892 6.886 6.750 6.745 6.067
4.794 4.787 4.779 4.772
3.083 3.068 3.059 3.044 3.032 3.024 3.009 3.001
1.672 1.548
0.484 0.480
1H NMR (CDCl3), 600 MHz
SiMe2Ph
OH
S
Cl
10j
1.95 3.13 1.98 2.10 1.01 1.02 0.96 0.94 1.00 2.00 1.03 6.03
ppm

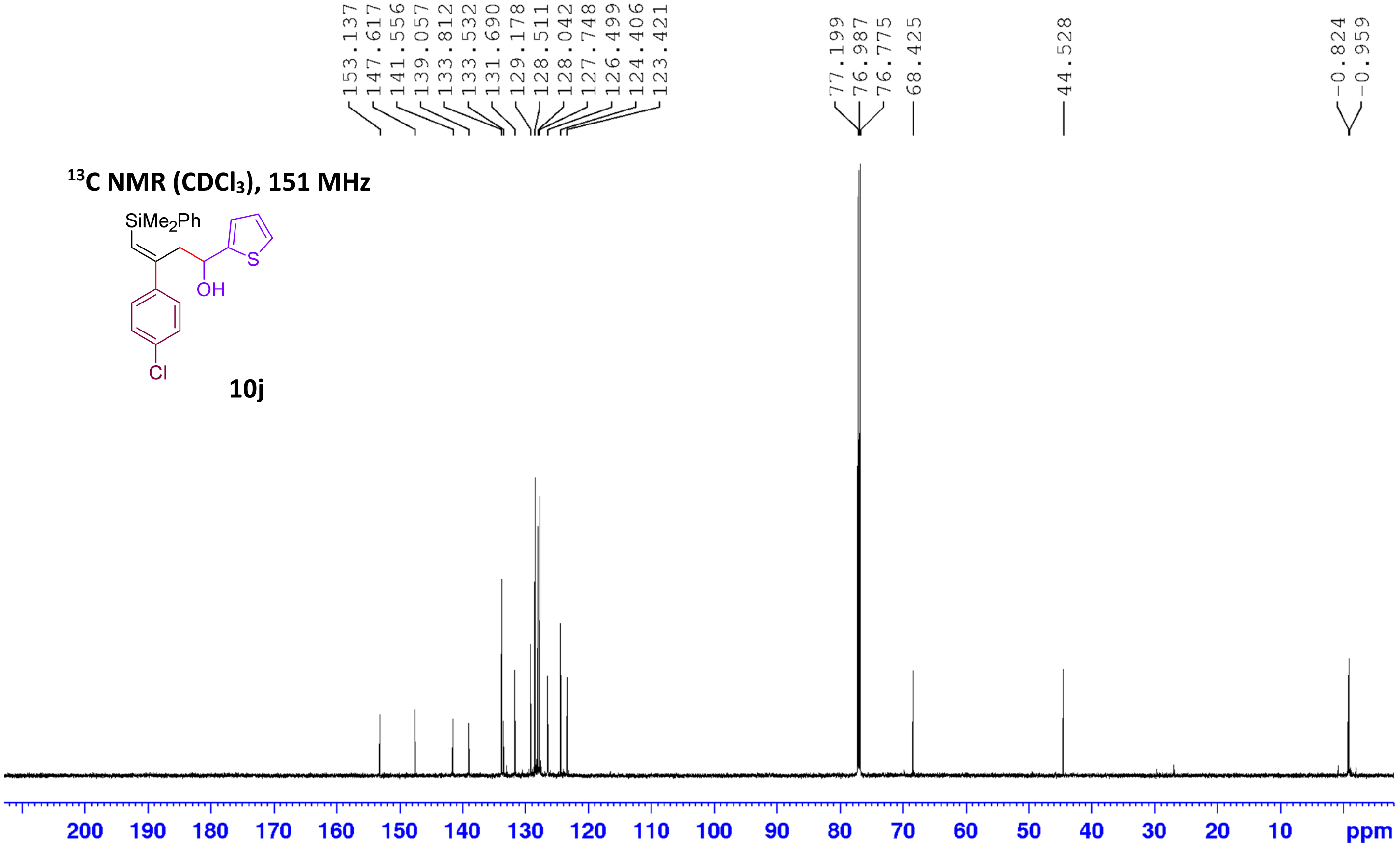

153.137 147.617 141.556 139.057 133.812 133.532 131.690 129.178 128.511 128.042 127.748 126.499 124.406 123.421
77.199 76.987 76.775 68.425
44.528
-0.824 -0.959
13C NMR (CDCl3), 151 MHz
SiMe2Ph
OH
S
Cl
10j
ppm

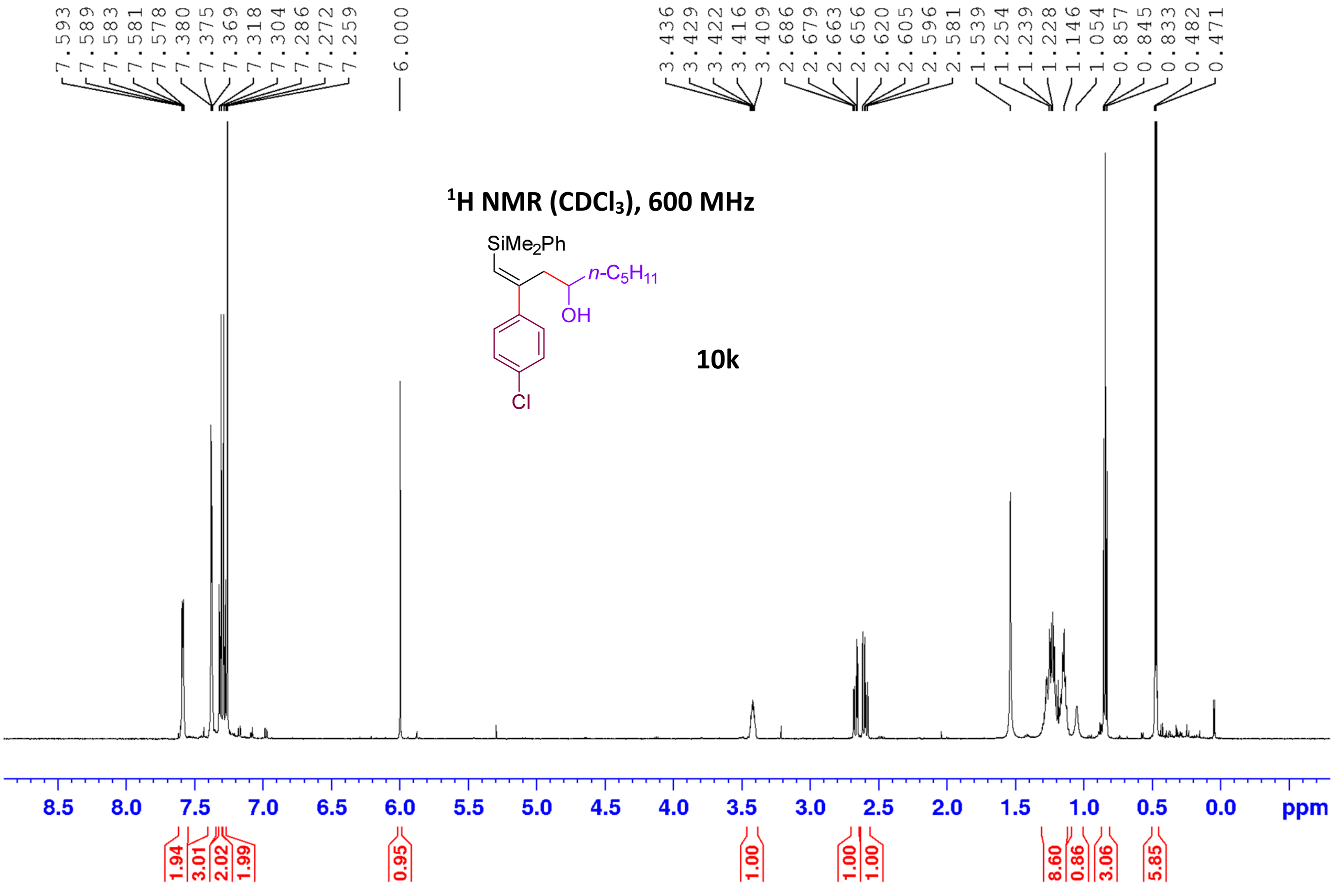
7.593
7.589
7.583
7.581
7.578
7.380
7.375
7.369
7.318
7.304
7.286
7.272
7.259
6.000
3.436
3.429
3.422
3.416
3.409
2.686
2.679
2.663
2.656
2.620
2.605
2.596
2.581
1.539
1.254
1.239
1.228
1.146
1.054
0.857
0.845
0.833
0.482
0.471
1H NMR (CDCl3), 600 MHz
SiMe2Ph
n-C5H11
OH
Cl
10k
8.5
8.0
7.5
7.0
6.5
6.0
5.5
5.0
4.5
4.0
3.5
3.0
2.5
2.0
1.5
1.0
0.5
0.0
ppm
1.94
3.01
2.02
1.99
0.95
1.00
1.00
1.00
8.60
0.86
3.06
5.85

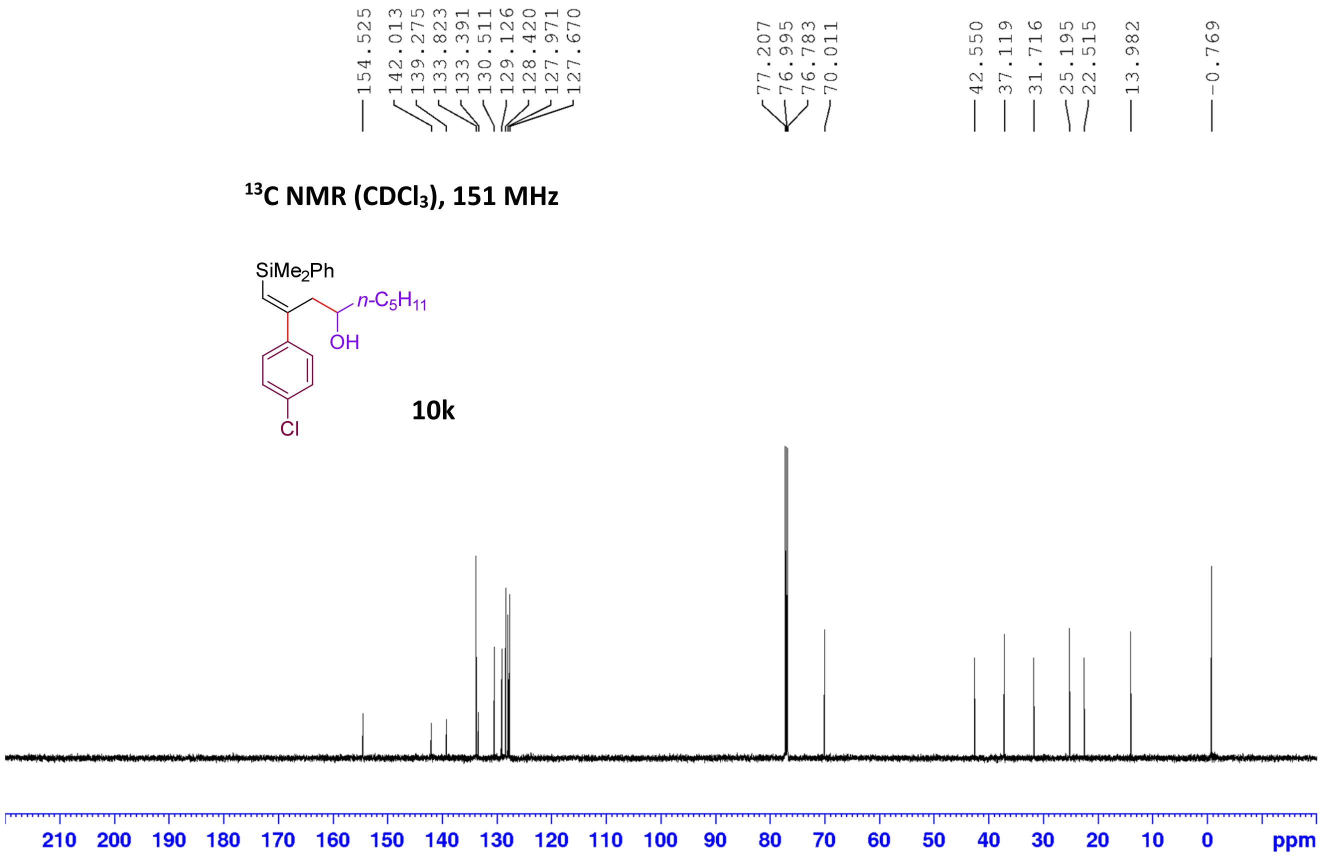
154.525
142.013
139.275
133.823
133.391
130.511
129.126
128.420
127.971
127.670
77.207
76.995
76.783
70.011
42.550
37.119
31.716
25.195
22.515
13.982
-0.769
13C NMR (CDCl3), 151 MHz
SiMe2Ph
n-C5H11
OH
Cl
10k
210
200
190
180
170
160
150
140
130
120
110
100
90
80
70
60
50
40
30
20
10
0
ppm

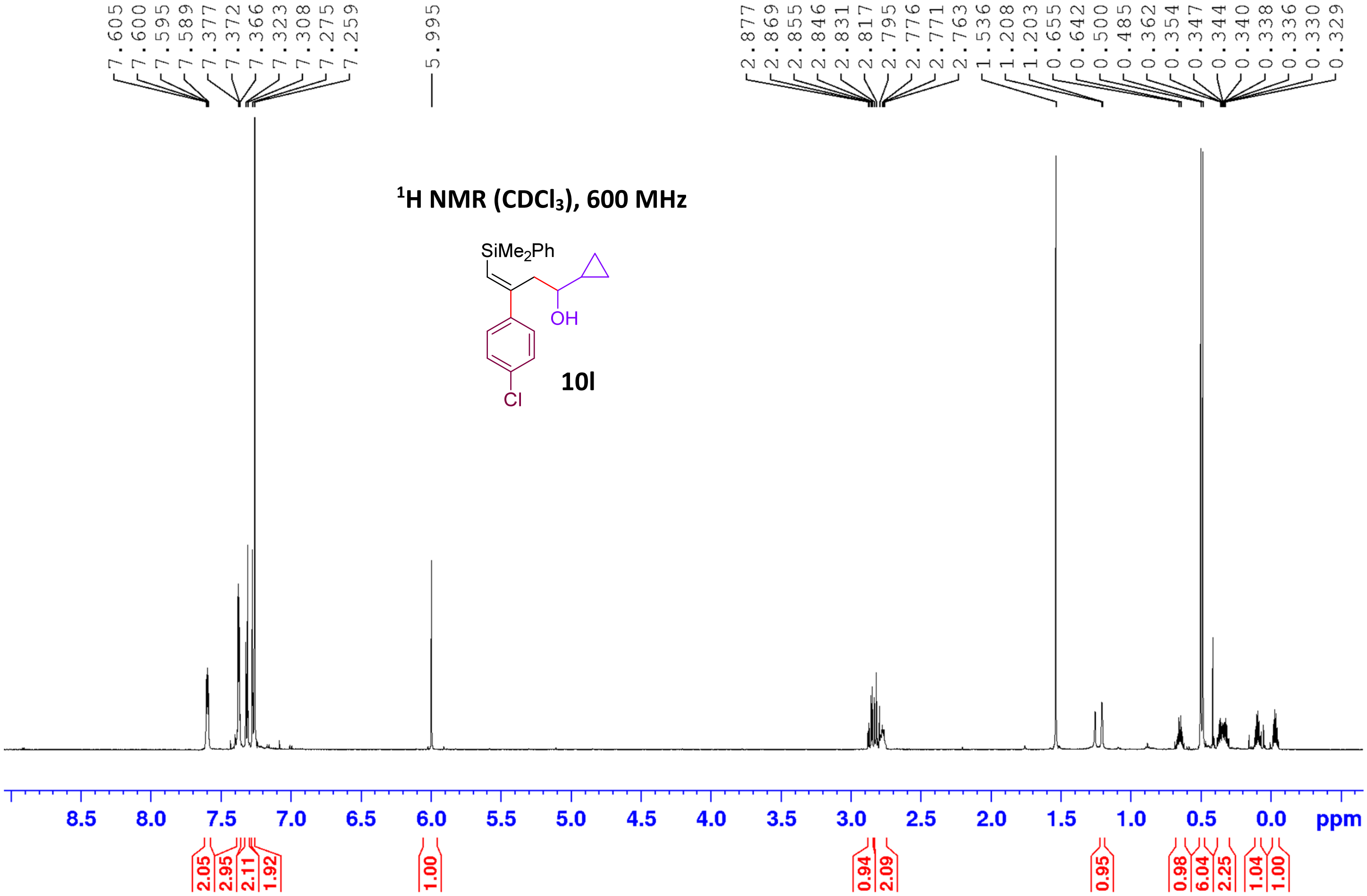
7.605
7.600
7.595
7.589
7.377
7.372
7.366
7.323
7.308
7.275
7.259
5.995
2.877
2.869
2.855
2.846
2.831
2.817
2.795
2.776
2.771
2.763
1.536
1.208
1.203
0.655
0.642
0.500
0.485
0.362
0.354
0.347
0.344
0.340
0.338
0.336
0.330
0.329
¹H NMR (CDCl₃), 600 MHz
SiMe₂Ph
OH
Cl
10l
8.5 8.0 7.5 7.0 6.5 6.0 5.5 5.0 4.5 4.0 3.5 3.0 2.5 2.0 1.5 1.0 0.5 0.0 ppm
2.05
2.95
2.11
1.92
1.00
0.94
2.09
0.95
0.98
6.04
2.25
1.04
1.00

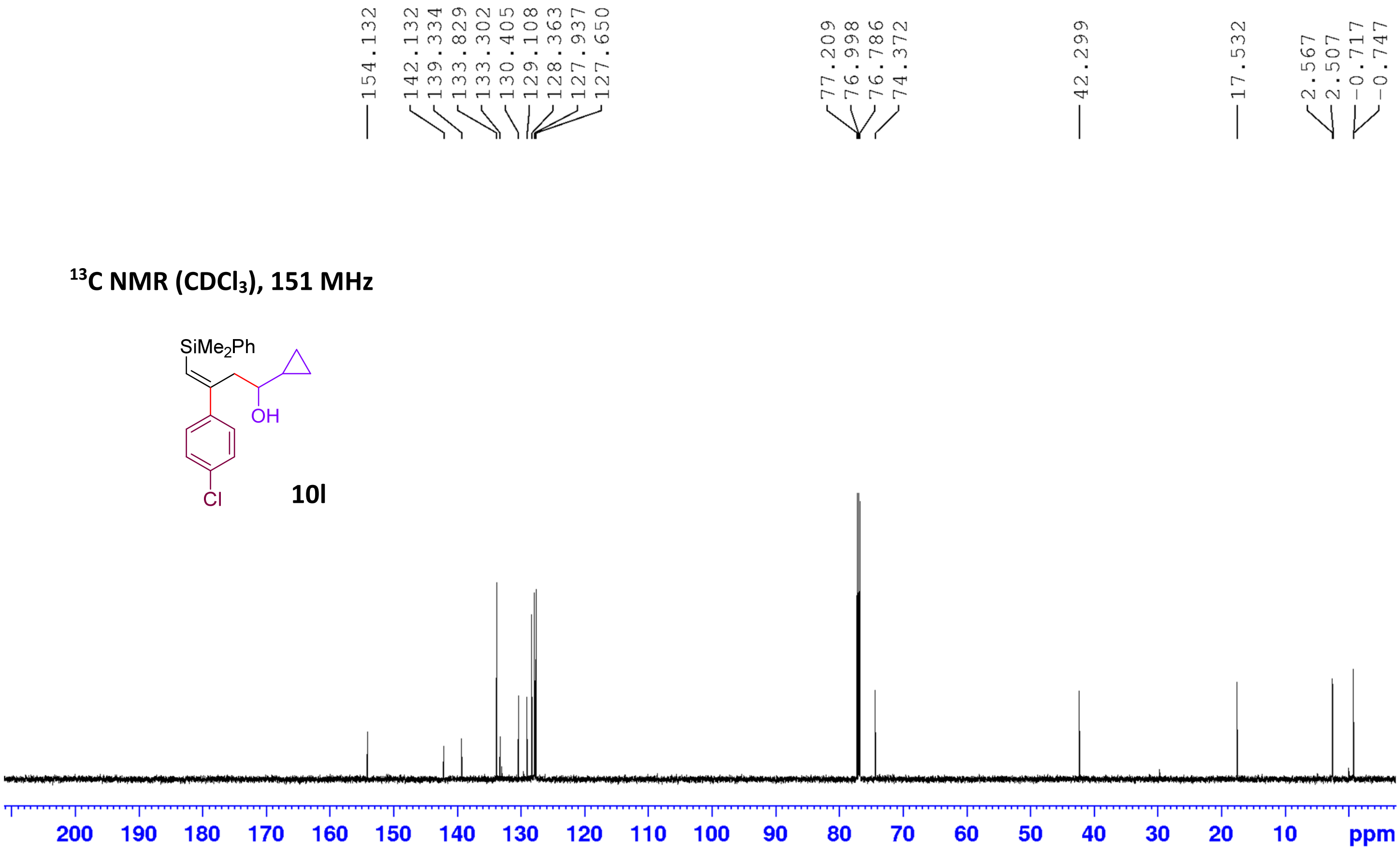
154.132
142.132
139.334
133.829
133.302
130.405
129.108
128.363
127.937
127.650
77.209
76.998
76.786
74.372
42.299
17.532
2.567
2.507
-0.717
-0.747
¹³C NMR (CDCl₃), 151 MHz
SiMe₂Ph
OH
Cl
10l
200 190 180 170 160 150 140 130 120 110 100 90 80 70 60 50 40 30 20 10 ppm

^{1}H NMR ($CDCl_3$), 600 MHz

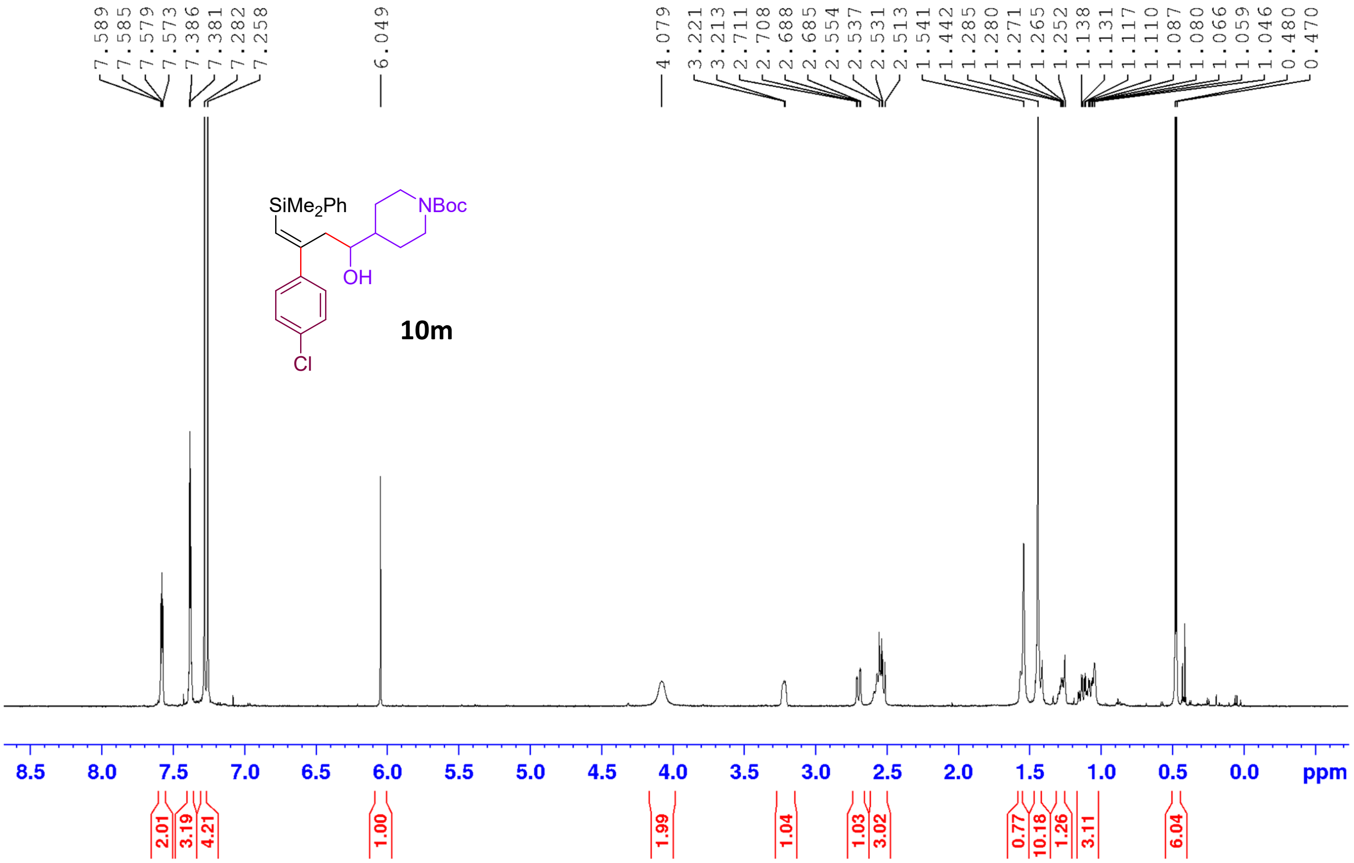

^{13}C NMR ($CDCl_3$), 151 MHz

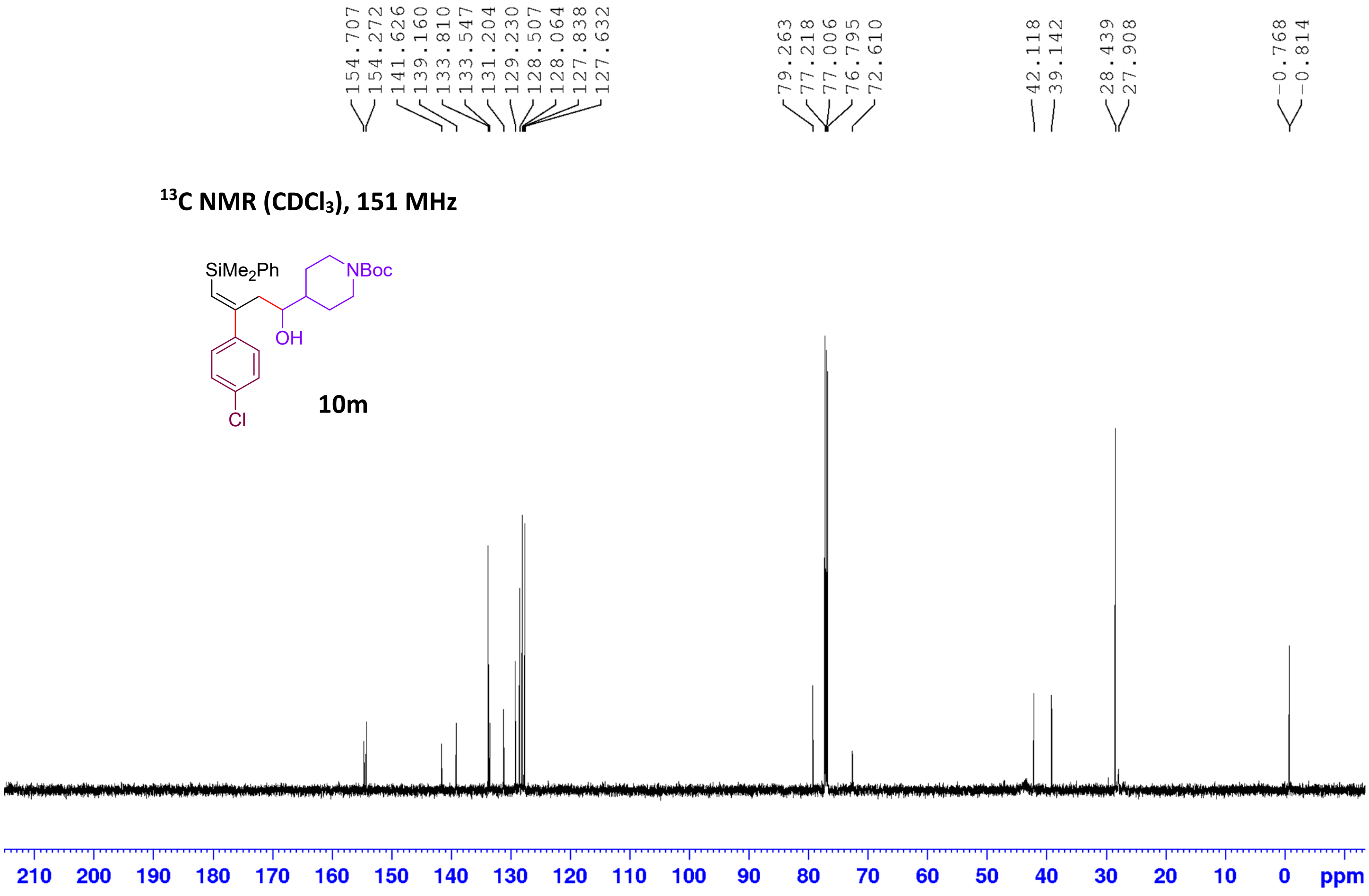

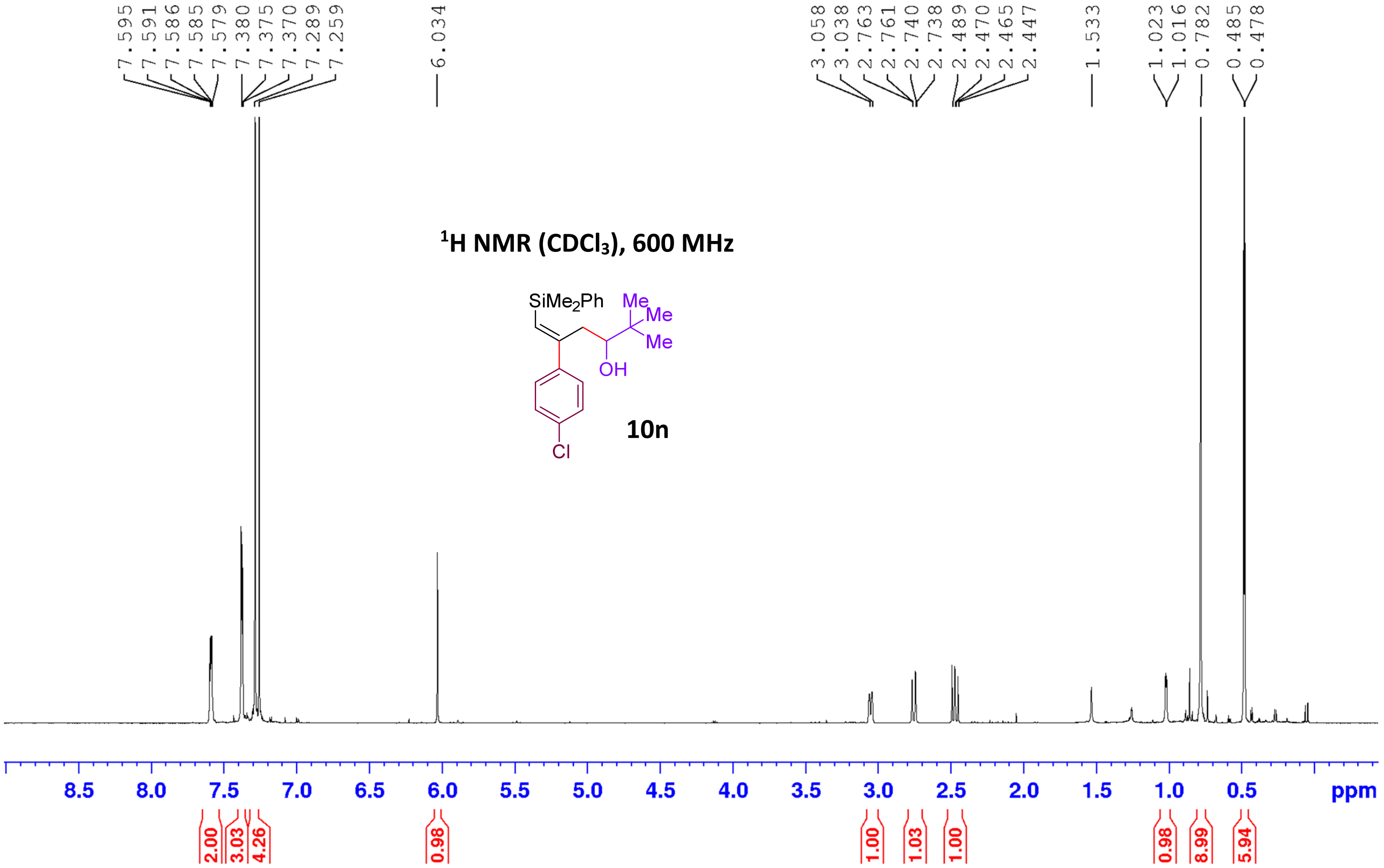

7.595
7.591
7.586
7.585
7.579
7.380
7.375
7.370
7.289
7.259
6.034
3.058
3.038
2.763
2.761
2.740
2.738
2.489
2.470
2.465
2.447
1.533
1.023
1.016
0.782
0.485
0.478
1H NMR (CDCl3), 600 MHz
SiMe2Ph
Me
Me
Me
OH
Cl
10n
8.5
8.0
7.5
7.0
6.5
6.0
5.5
5.0
4.5
4.0
3.5
3.0
2.5
2.0
1.5
1.0
0.5
ppm
2.00
3.03
4.26
0.98
1.00
1.03
1.00
0.98
8.99
5.94

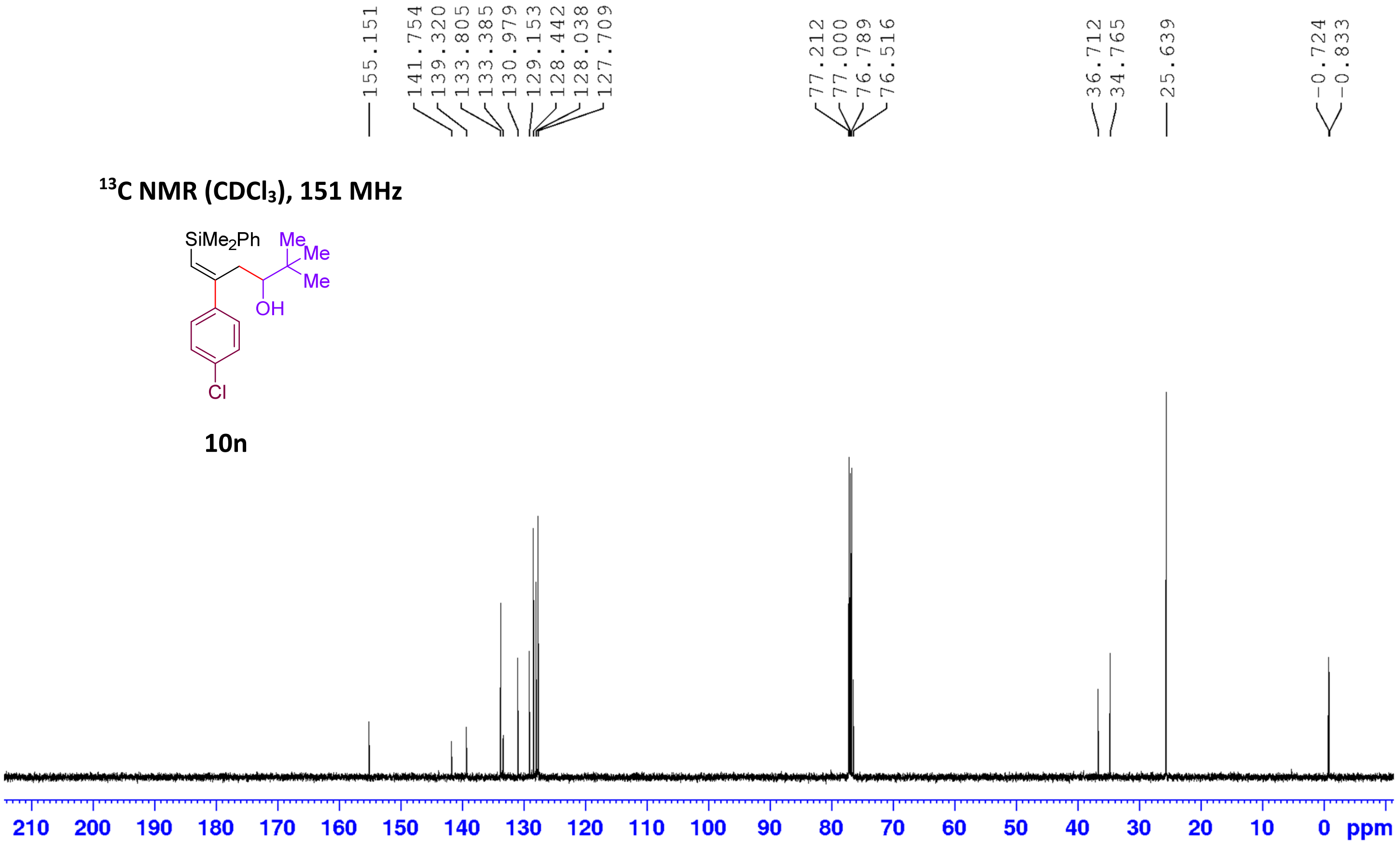

155.151
141.754
139.320
133.805
133.385
130.979
129.153
128.442
128.038
127.709
77.212
77.000
76.789
76.516
36.712
34.765
25.639
-0.724
-0.833
13C NMR (CDCl3), 151 MHz
SiMe2Ph
Me
Me
Me
OH
Cl
10n
210
200
190
180
170
160
150
140
130
120
110
100
90
80
70
60
50
40
30
20
10
0
ppm

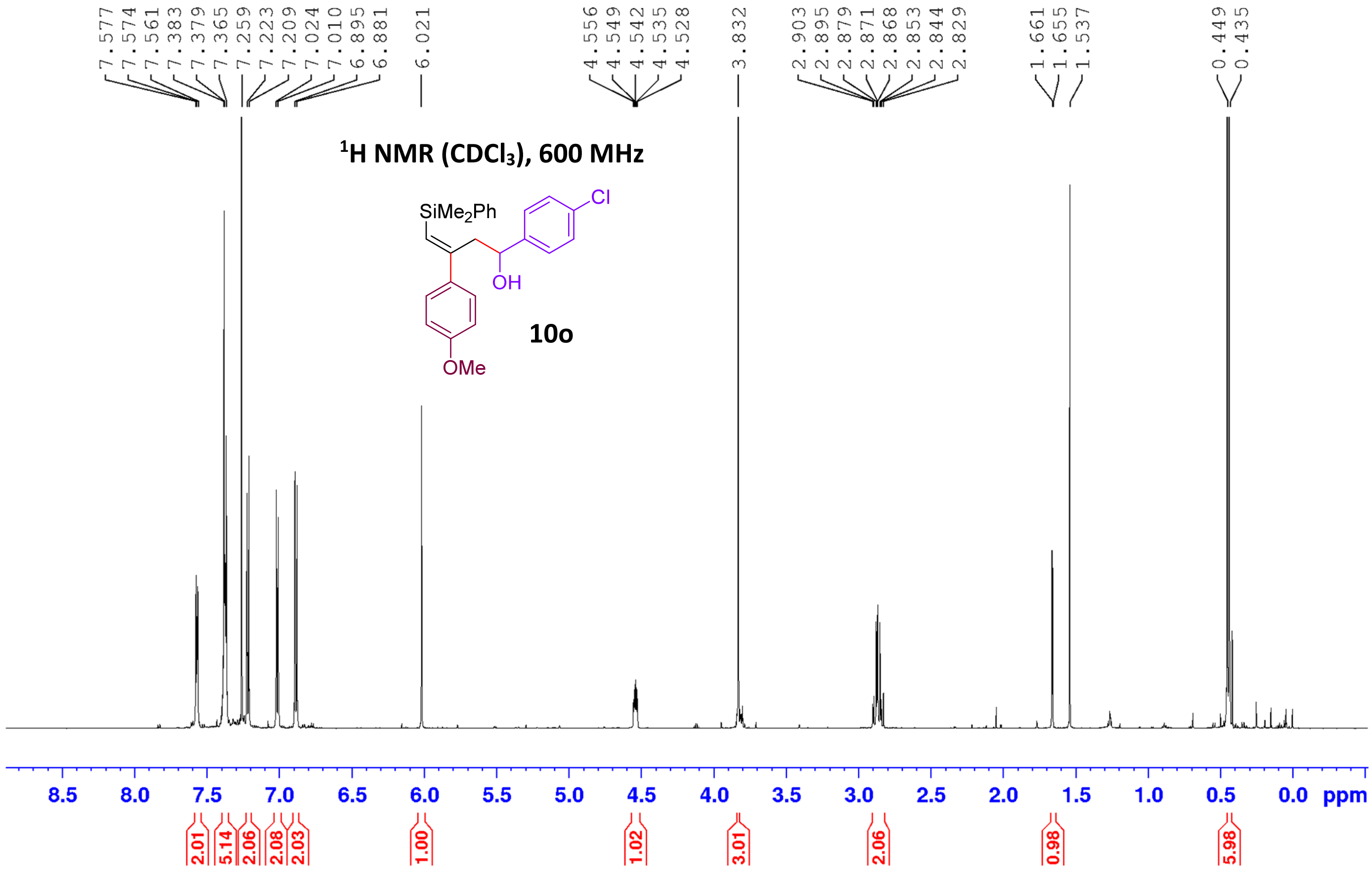
7.577 7.574 7.561 7.383 7.379 7.365 7.259 7.223 7.209 7.024 7.010 6.895 6.881
6.021
4.556 4.549 4.542 4.535 4.528
3.832
2.903 2.895 2.879 2.871 2.868 2.853 2.844 2.829
1.661 1.655 1.537
0.449 0.435
1H NMR (CDCl3), 600 MHz
SiMe2Ph
Cl
OH
OMe
10o
8.5 8.0 7.5 7.0 6.5 6.0 5.5 5.0 4.5 4.0 3.5 3.0 2.5 2.0 1.5 1.0 0.5 0.0 ppm
2.01 5.14 2.06 2.08 2.03 1.00 1.02 3.01 2.06 0.98 5.98

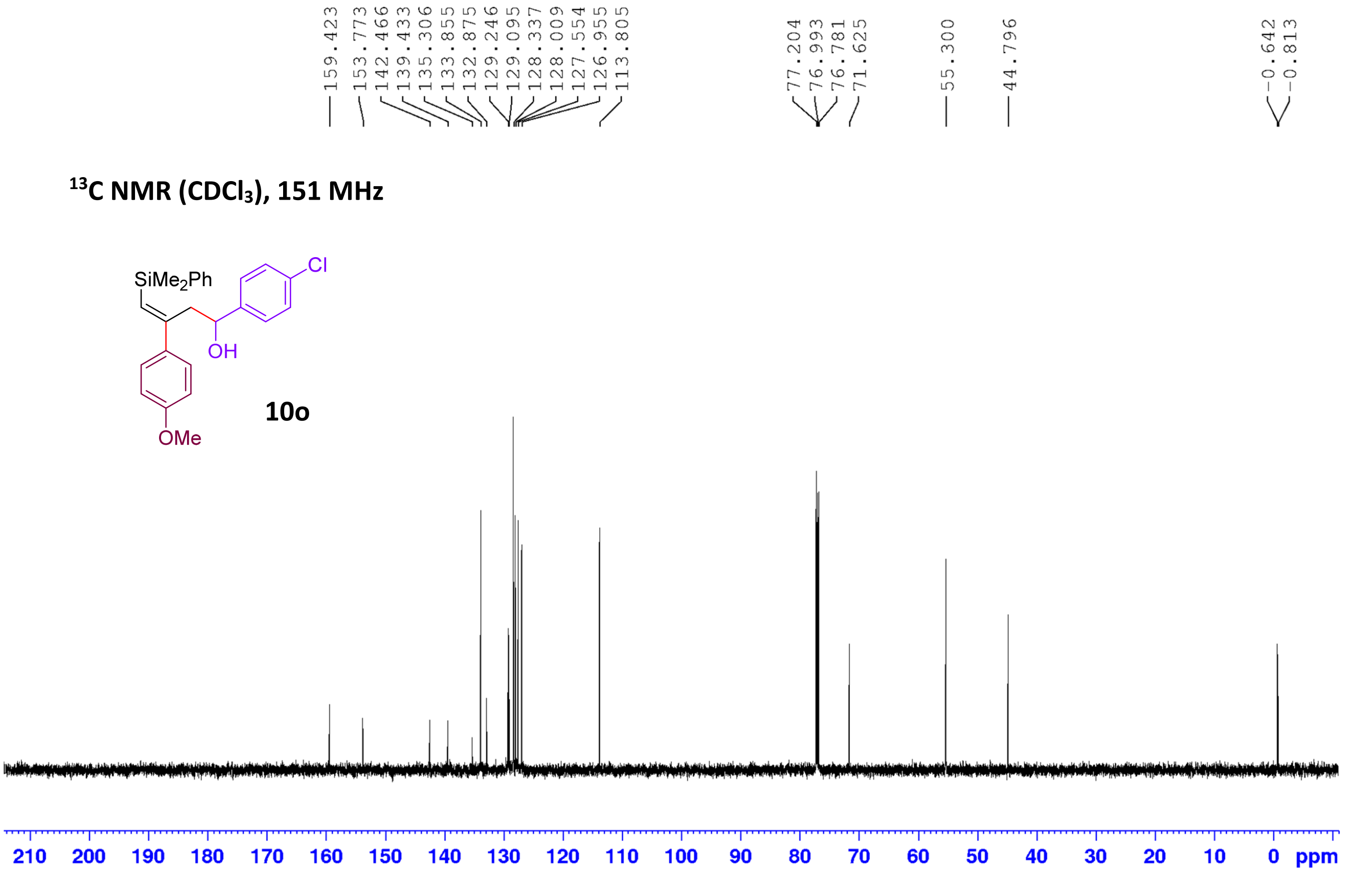
159.423 153.773 142.466 139.433 135.306 133.855 132.875 129.246 129.095 128.337 128.009 127.554 126.955 113.805
77.204 76.993 76.781 71.625
55.300
44.796
-0.642 -0.813
13C NMR (CDCl3), 151 MHz
SiMe2Ph
Cl
OH
OMe
10o
210 200 190 180 170 160 150 140 130 120 110 100 90 80 70 60 50 40 30 20 10 0 ppm

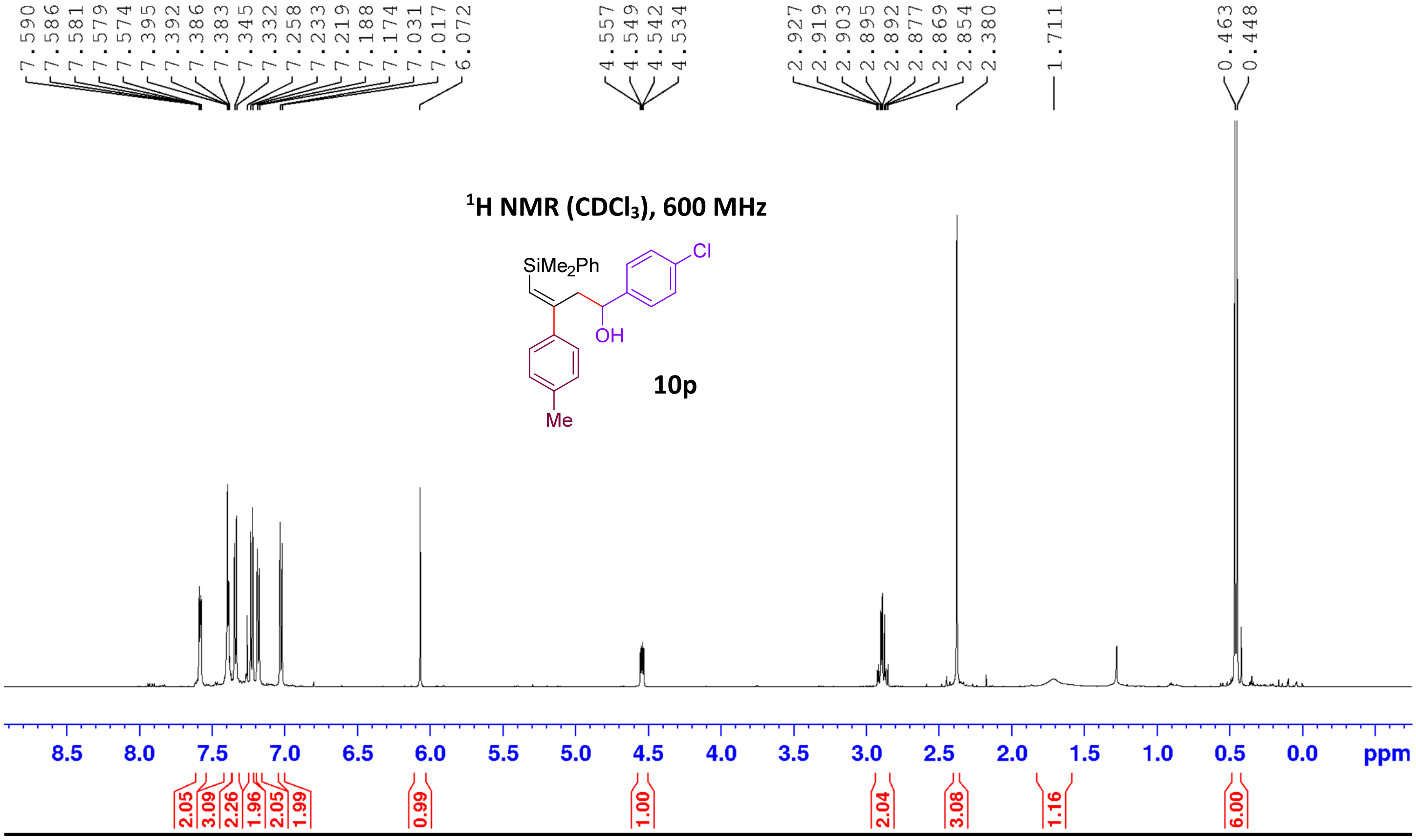
1H NMR (CDCl3), 600 MHz
SiMe2Ph
Cl
OH
Me
10p
7.590 7.586 7.581 7.579 7.574 7.395 7.392 7.386 7.383 7.345 7.332 7.258 7.233 7.219 7.188 7.174 7.031 7.017 6.072
4.557 4.549 4.542 4.534
2.927 2.919 2.903 2.895 2.892 2.877 2.869 2.854 2.380
1.711
0.463 0.448
8.5 8.0 7.5 7.0 6.5 6.0 5.5 5.0 4.5 4.0 3.5 3.0 2.5 2.0 1.5 1.0 0.5 0.0 ppm
2.05 3.09 2.26 1.96 2.05 1.99 0.99 1.00 2.04 3.08 1.16 6.00

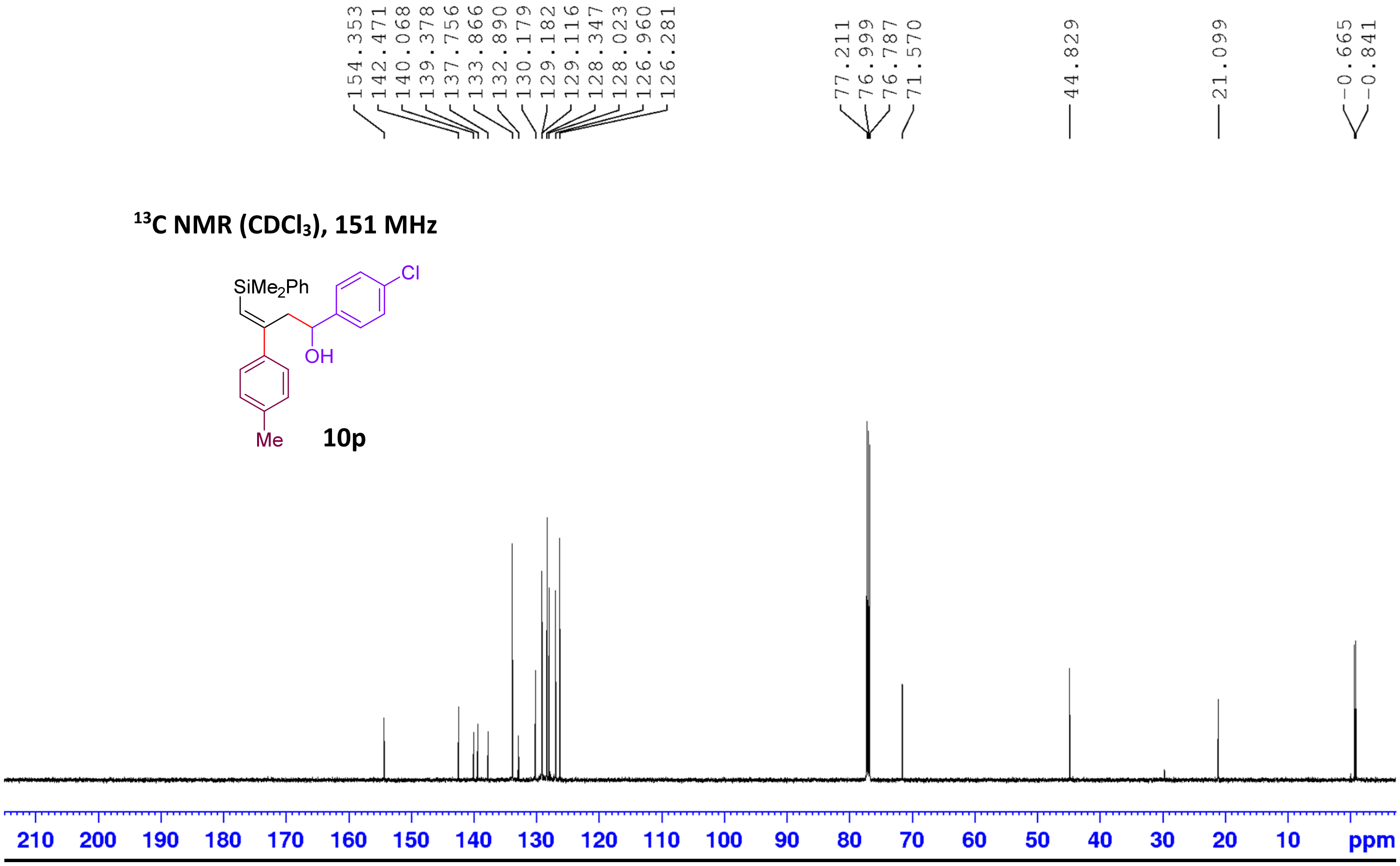
13C NMR (CDCl3), 151 MHz
SiMe2Ph
Cl
OH
Me
10p
154.353 142.471 140.068 139.378 137.756 133.866 132.890 130.179 129.182 129.116 128.347 128.023 126.960 126.281
77.211 76.999 76.787 71.570
44.829
21.099
-0.665 -0.841
210 200 190 180 170 160 150 140 130 120 110 100 90 80 70 60 50 40 30 20 10 ppm

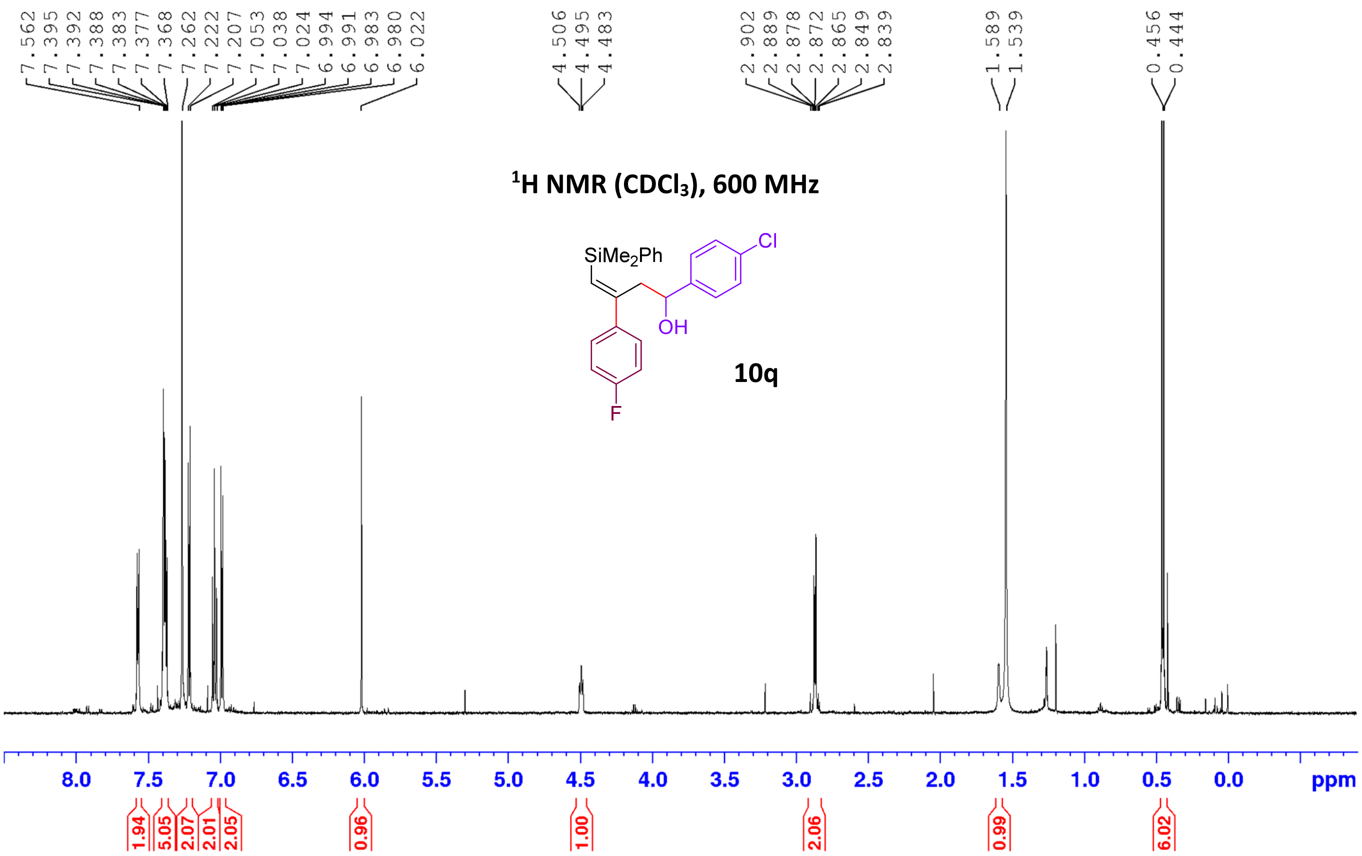

7.562
7.395
7.392
7.388
7.383
7.377
7.368
7.262
7.222
7.207
7.053
7.038
7.024
6.994
6.991
6.983
6.980
6.022
4.506
4.495
4.483
2.902
2.889
2.878
2.872
2.865
2.849
2.839
1.589
1.539
0.456
0.444
^{1}H NMR (CDCl$_3$), 600 MHz
SiMe$_2$Ph
Cl
OH
F
10q
8.0 7.5 7.0 6.5 6.0 5.5 5.0 4.5 4.0 3.5 3.0 2.5 2.0 1.5 1.0 0.5 0.0 ppm
1.94
5.05
2.07
2.01
2.05
0.96
1.00
2.06
0.99
6.02

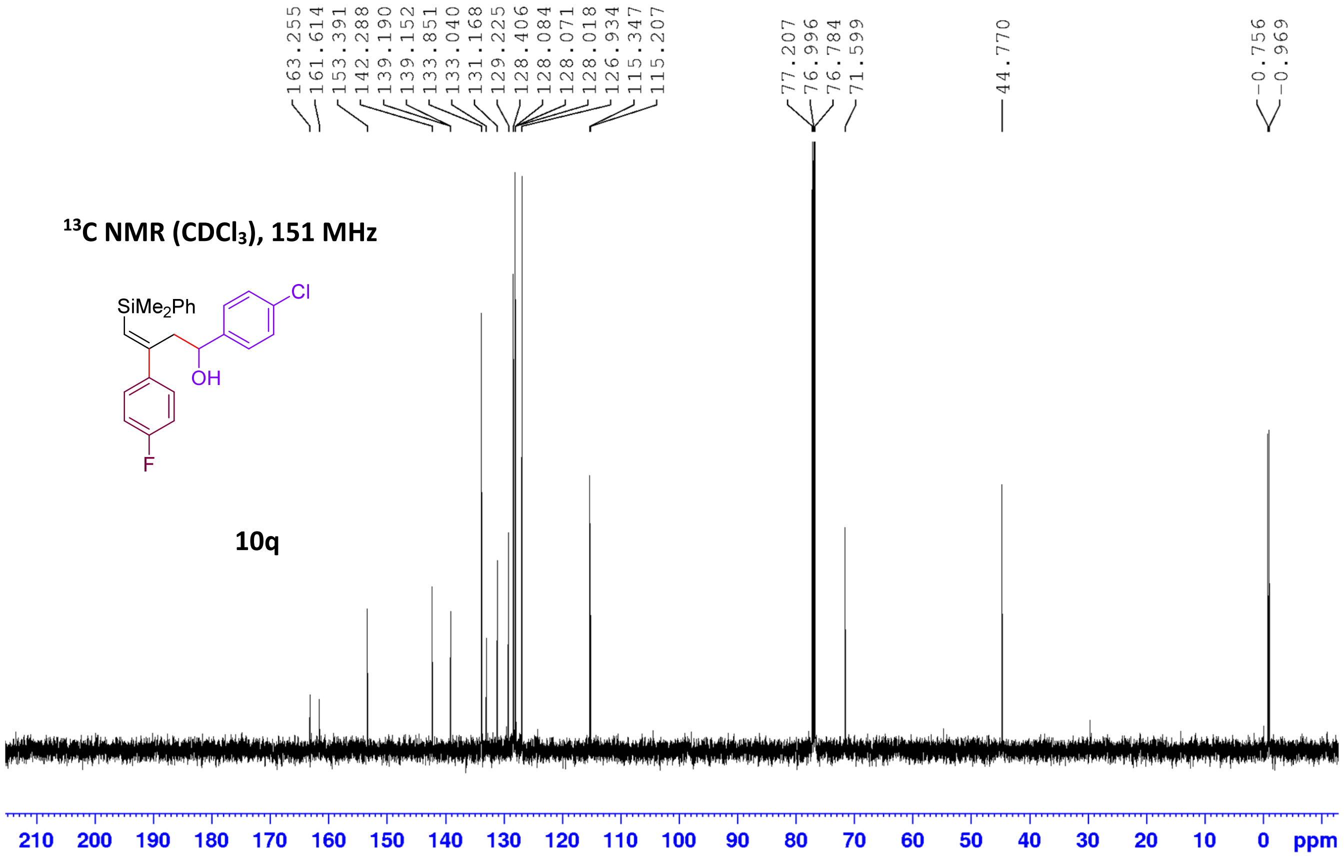

163.255
161.614
153.391
142.288
139.190
139.152
133.851
133.040
131.168
129.225
128.406
128.084
128.071
128.018
126.934
115.347
115.207
77.207
76.996
76.784
71.599
44.770
-0.756
-0.969
^{13}C NMR (CDCl$_3$), 151 MHz
SiMe$_2$Ph
Cl
OH
F
10q
210 200 190 180 170 160 150 140 130 120 110 100 90 80 70 60 50 40 30 20 10 0 ppm

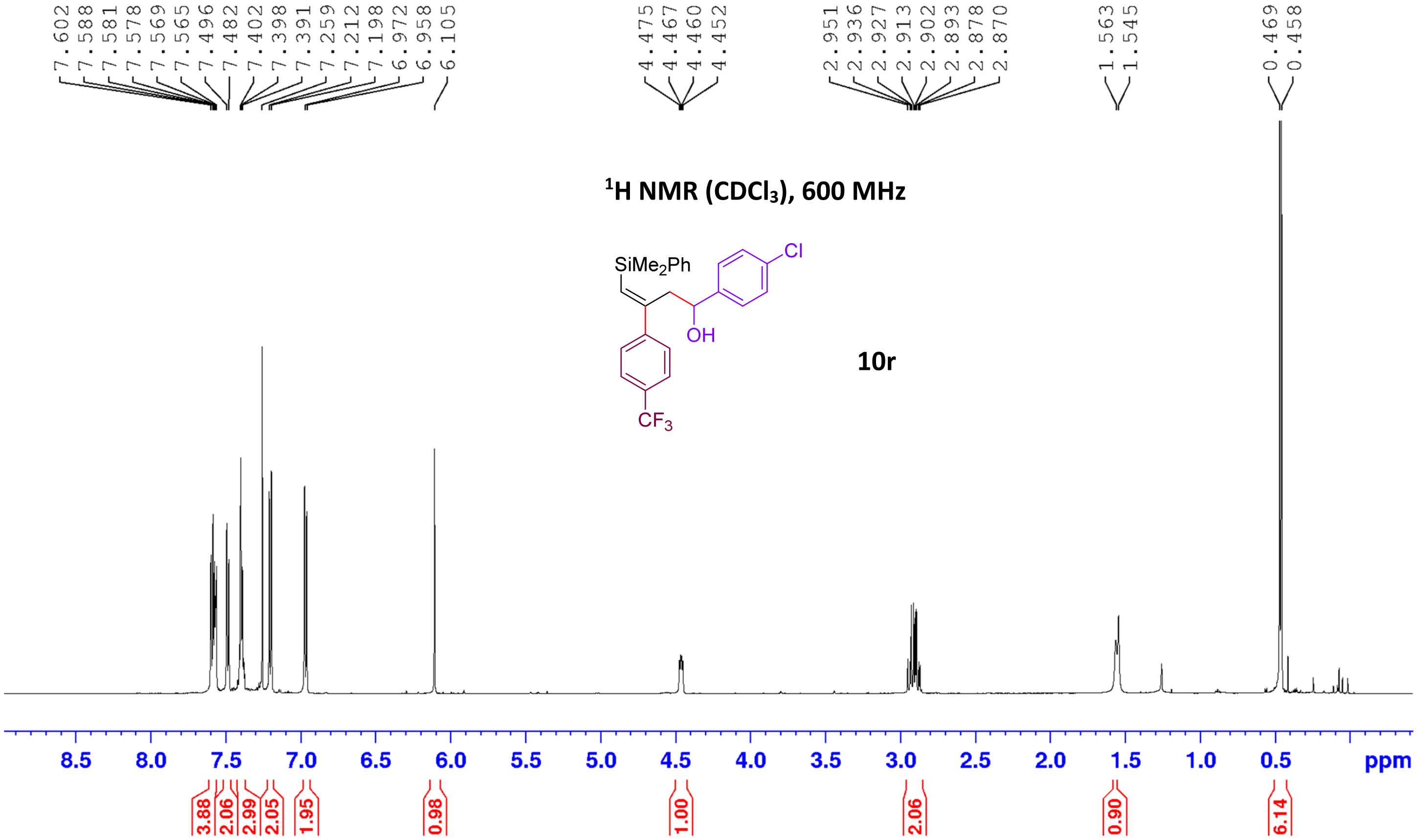
7.602
7.588
7.581
7.578
7.569
7.565
7.496
7.482
7.402
7.398
7.391
7.259
7.212
7.198
6.972
6.958
6.105
4.475
4.467
4.460
4.452
2.951
2.936
2.927
2.913
2.902
2.893
2.878
2.870
1.563
1.545
0.469
0.458
1H NMR (CDCl3), 600 MHz
SiMe2Ph
Cl
OH
CF3
10r
8.5
8.0
7.5
7.0
6.5
6.0
5.5
5.0
4.5
4.0
3.5
3.0
2.5
2.0
1.5
1.0
0.5
ppm
3.88
2.06
2.99
2.05
1.95
0.98
1.00
2.06
0.90
6.14

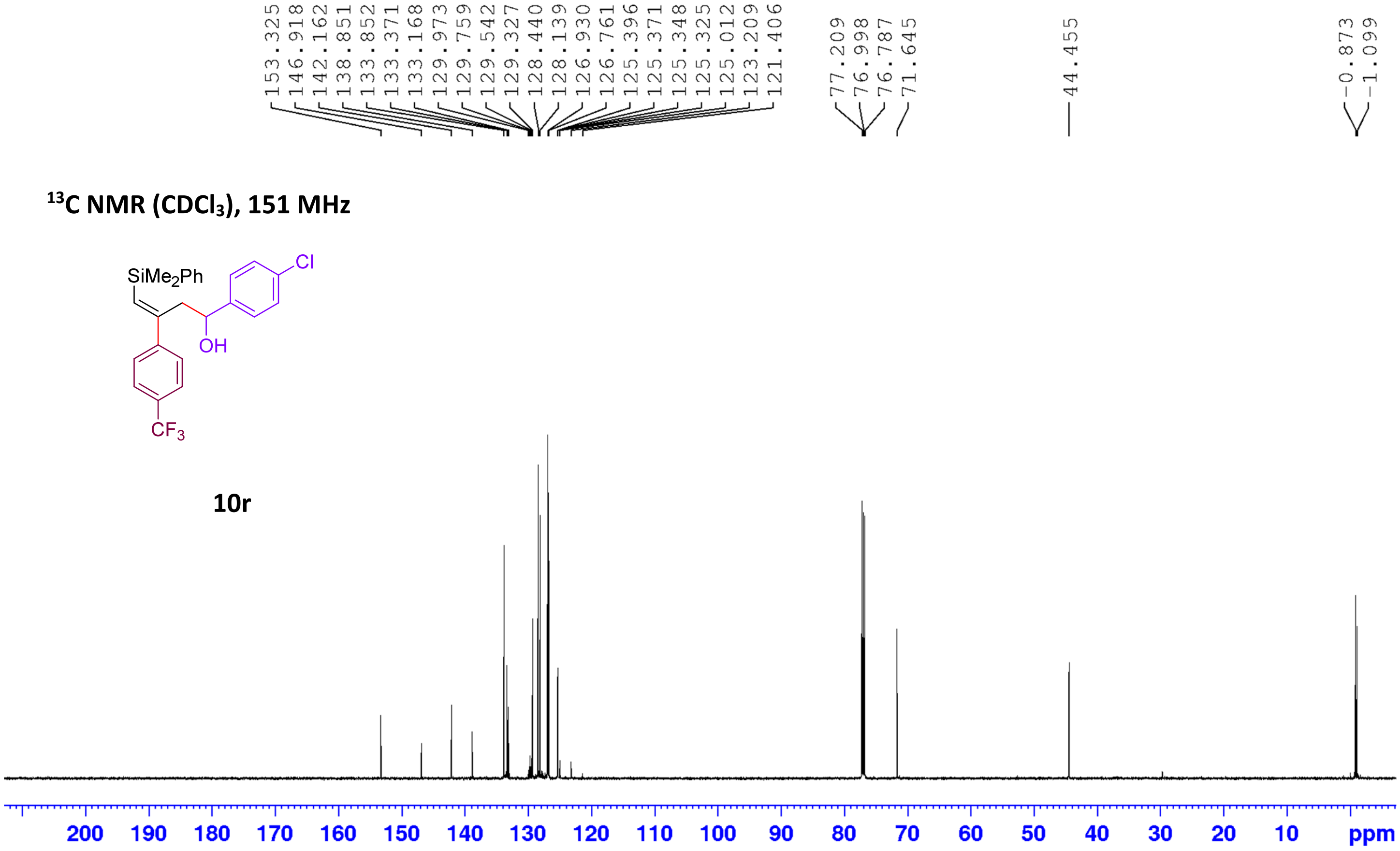
153.325
146.918
142.162
138.851
133.852
133.371
133.168
129.973
129.759
129.542
129.327
128.440
128.139
126.930
126.761
125.396
125.371
125.348
125.325
125.012
123.209
121.406
77.209
76.998
76.787
71.645
44.455
-0.873
-1.099
13C NMR (CDCl3), 151 MHz
SiMe2Ph
Cl
OH
CF3
10r
200
190
180
170
160
150
140
130
120
110
100
90
80
70
60
50
40
30
20
10
ppm

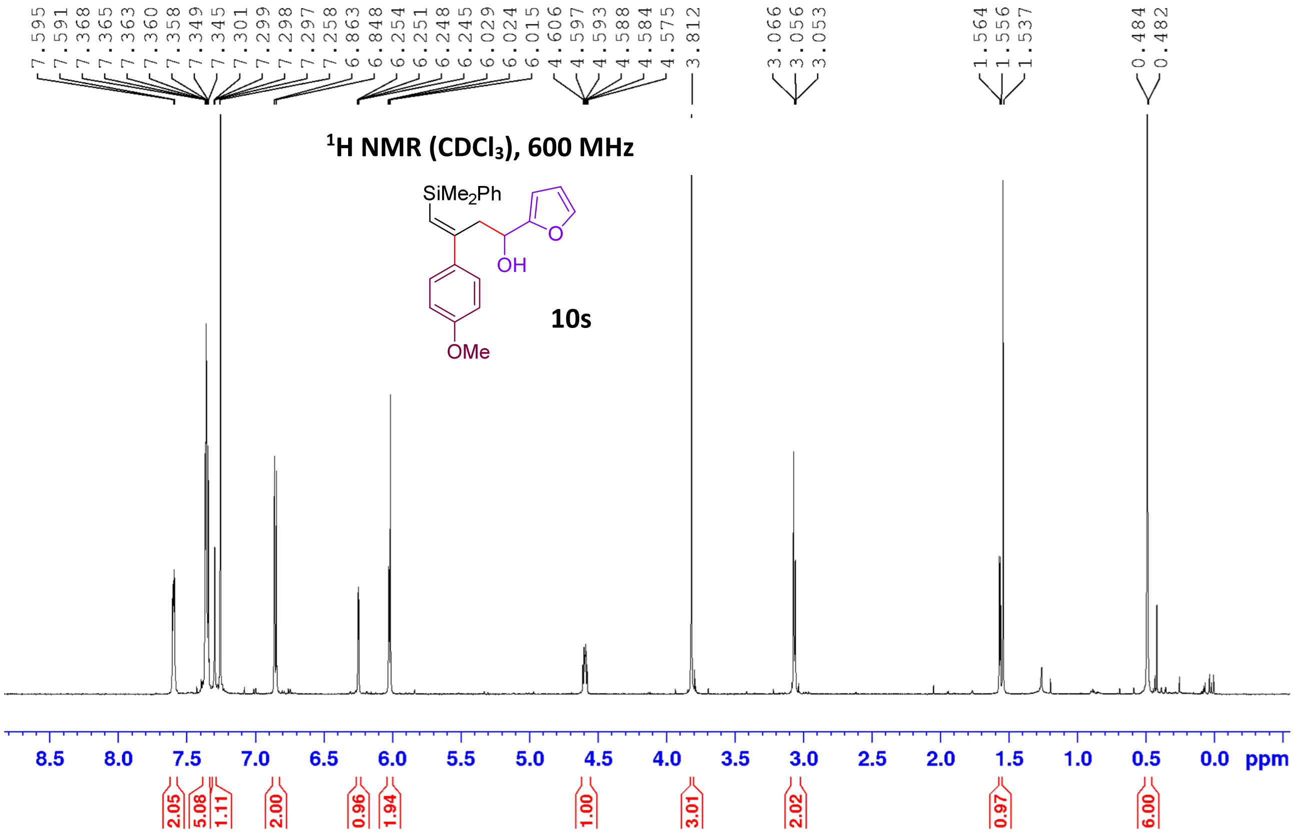
7.595
7.591
7.368
7.365
7.363
7.360
7.358
7.349
7.345
7.301
7.299
7.298
7.297
7.258
6.863
6.848
6.254
6.251
6.248
6.245
6.029
6.024
6.015
4.606
4.597
4.593
4.588
4.584
4.575
3.812
3.066
3.056
3.053
1.564
1.556
1.537
0.484
0.482
¹H NMR (CDCl₃), 600 MHz
SiMe₂Ph
OH
O
OMe
10s
2.05
5.08
1.11
2.00
0.96
1.94
1.00
3.01
2.02
0.97
6.00
ppm

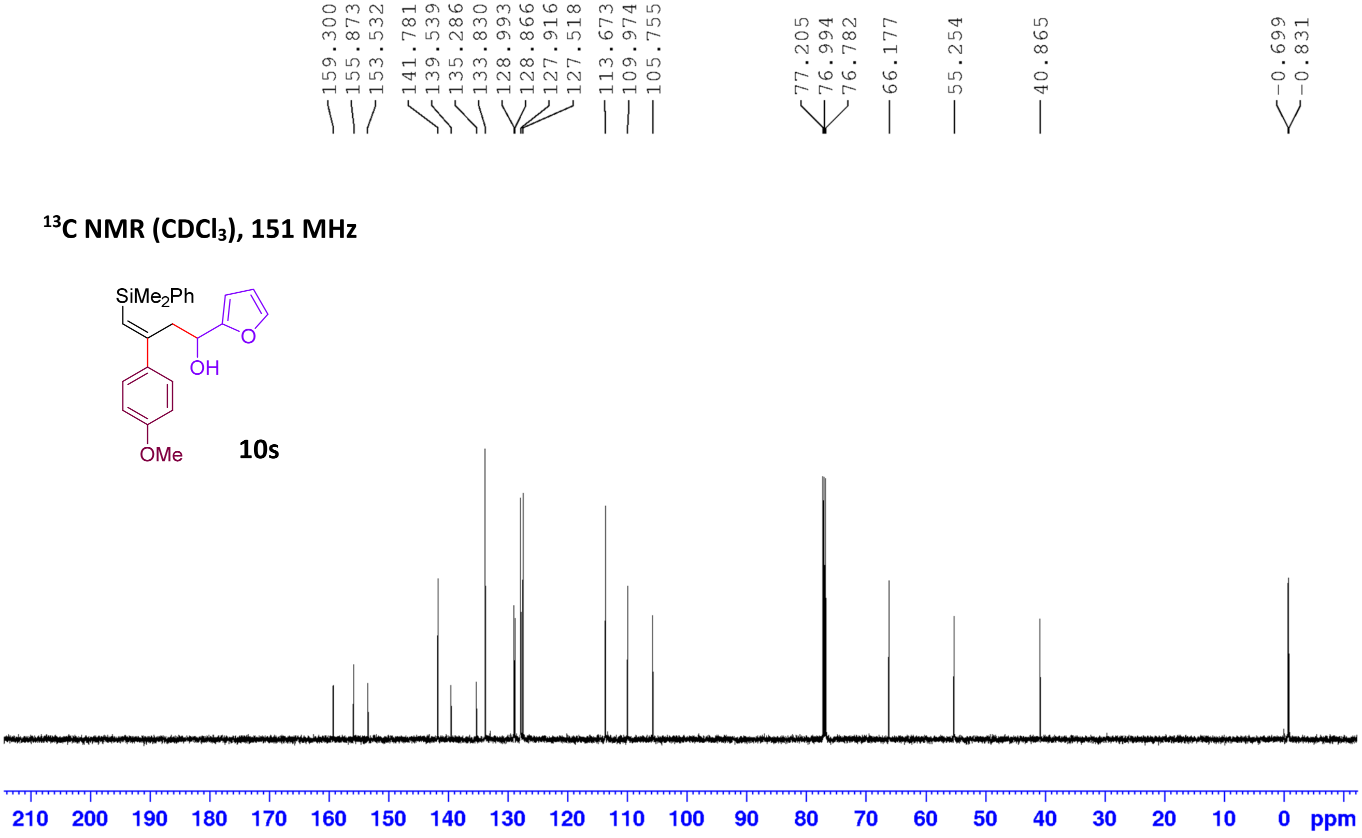
159.300
155.873
153.532
141.781
139.539
135.286
133.830
128.993
128.866
127.916
127.518
113.673
109.974
105.755
77.205
76.994
76.782
66.177
55.254
40.865
-0.699
-0.831
¹³C NMR (CDCl₃), 151 MHz
SiMe₂Ph
OH
O
OMe
10s
ppm

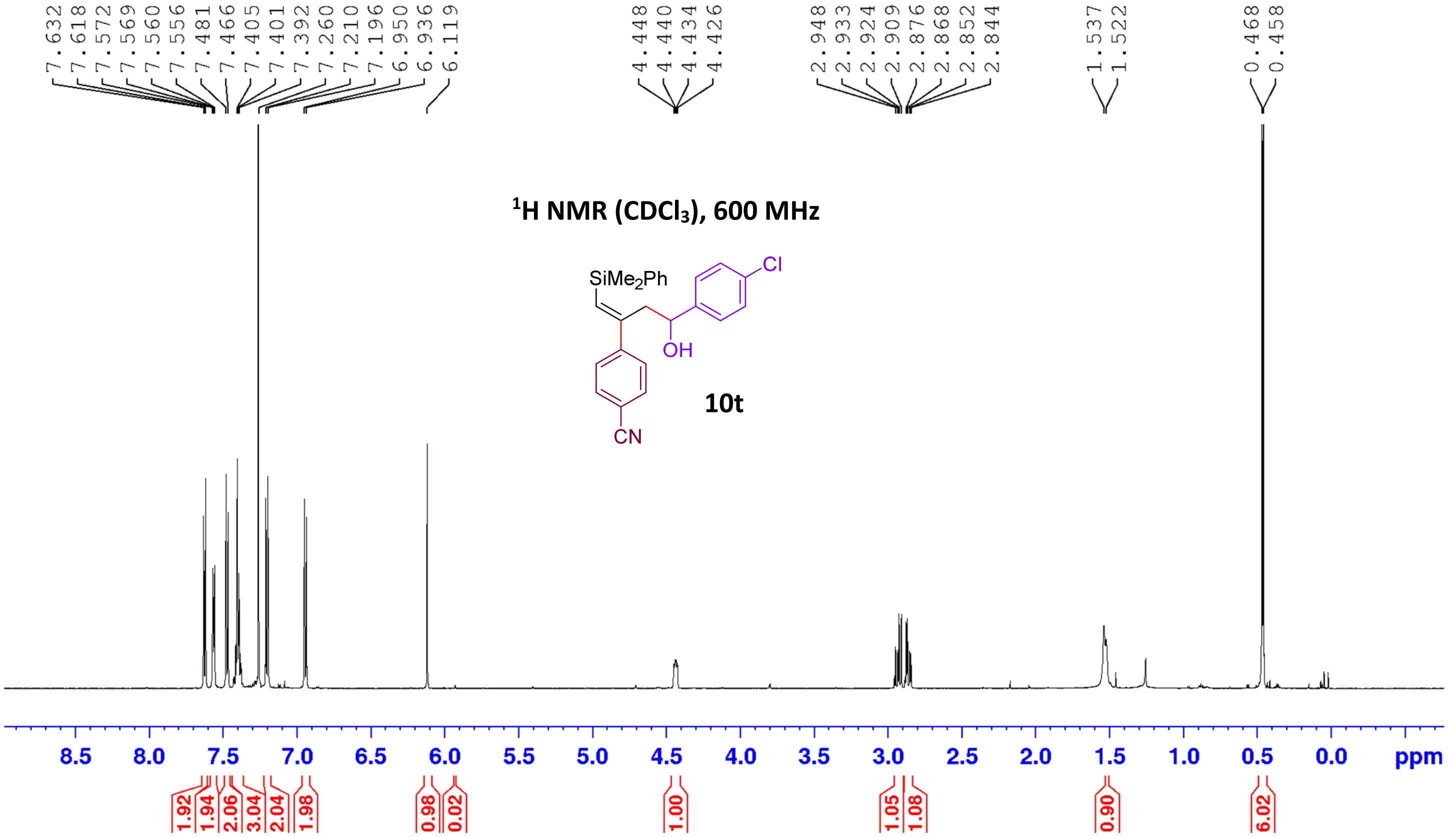

^{1}H NMR (CDCl$_3$), 600 MHz
SiMe$_2$Ph
Cl
OH
CN
10t
7.632 7.618 7.572 7.569 7.560 7.556 7.481 7.466 7.405 7.401 7.392 7.260 7.210 7.196 6.950 6.936 6.119
4.448 4.440 4.434 4.426
2.948 2.933 2.924 2.909 2.876 2.868 2.852 2.844
1.537 1.522
0.468 0.458
1.92 1.94 2.06 3.04 2.04 1.98 0.98 0.02 1.00 1.05 1.08 0.90 6.02
8.5 8.0 7.5 7.0 6.5 6.0 5.5 5.0 4.5 4.0 3.5 3.0 2.5 2.0 1.5 1.0 0.5 0.0 ppm

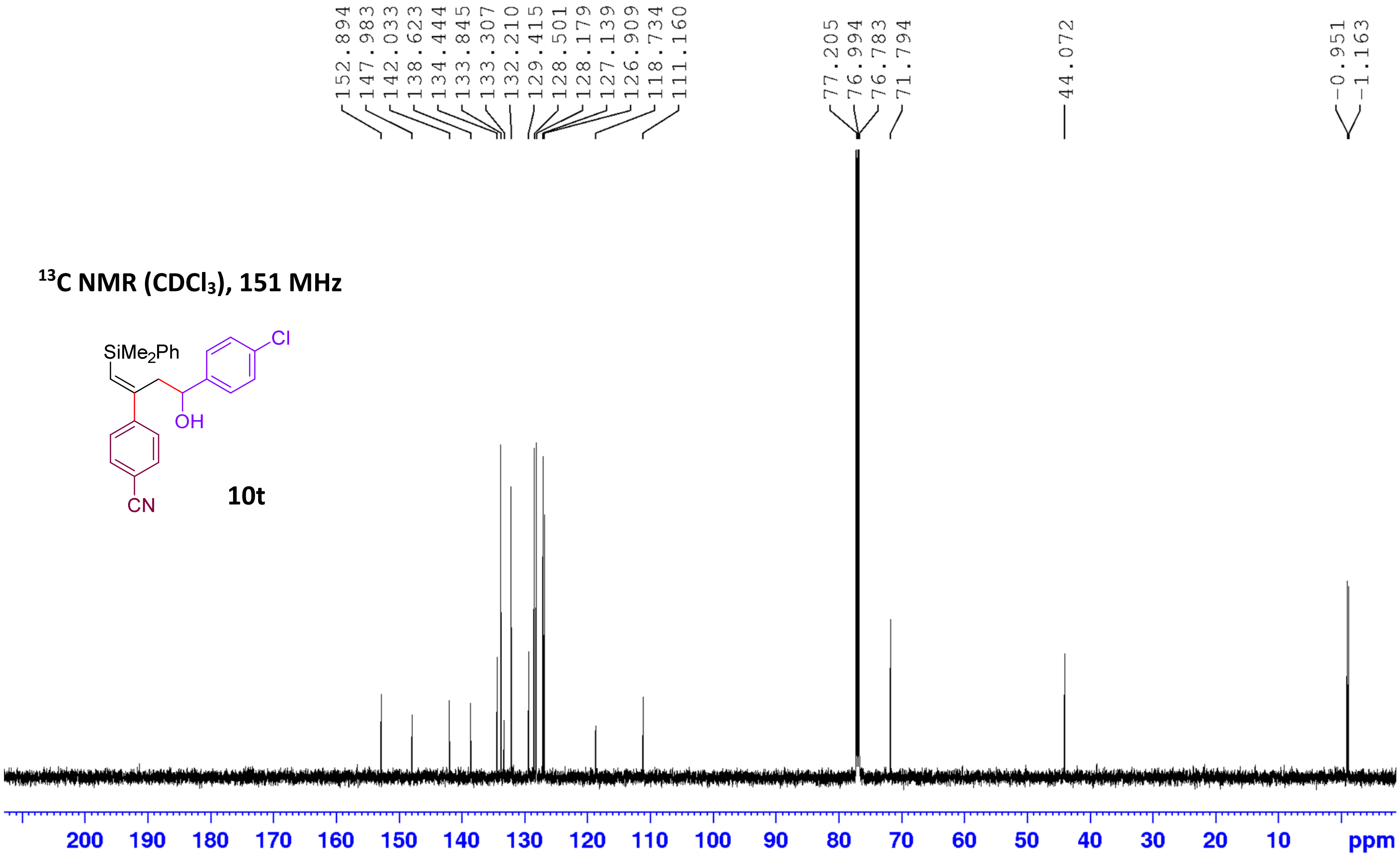

^{13}C NMR (CDCl$_3$), 151 MHz
SiMe$_2$Ph
Cl
OH
CN
10t
152.894 147.983 142.033 138.623 134.444 133.845 133.307 132.210 129.415 128.501 128.179 127.139 126.909 118.734 111.160
77.205 76.994 76.783 71.794
44.072
-0.951 -1.163
200 190 180 170 160 150 140 130 120 110 100 90 80 70 60 50 40 30 20 10 ppm

^{1}H NMR ($CDCl_3$), 600 MHz

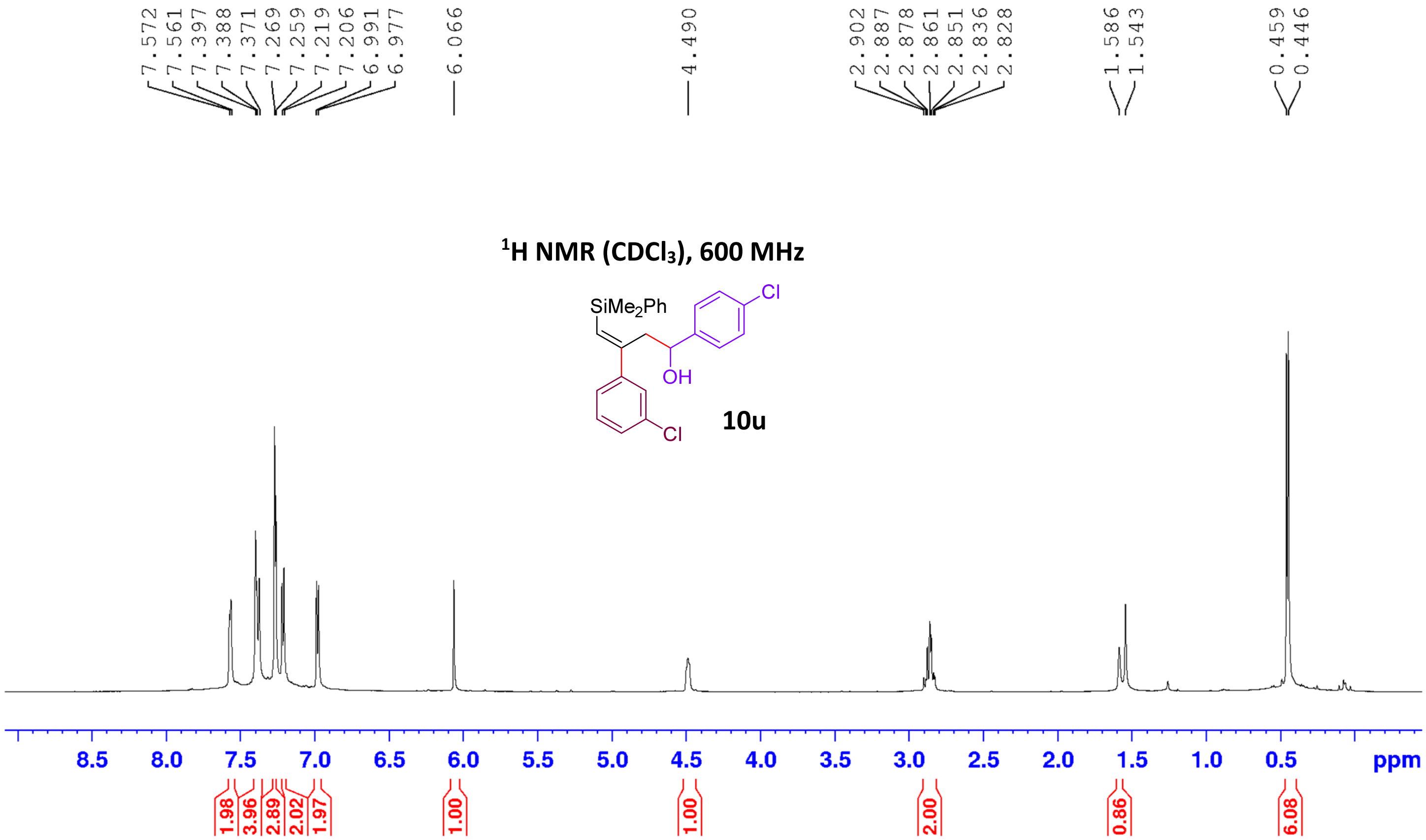

10u

^{13}C NMR ($CDCl_3$), 151 MHz

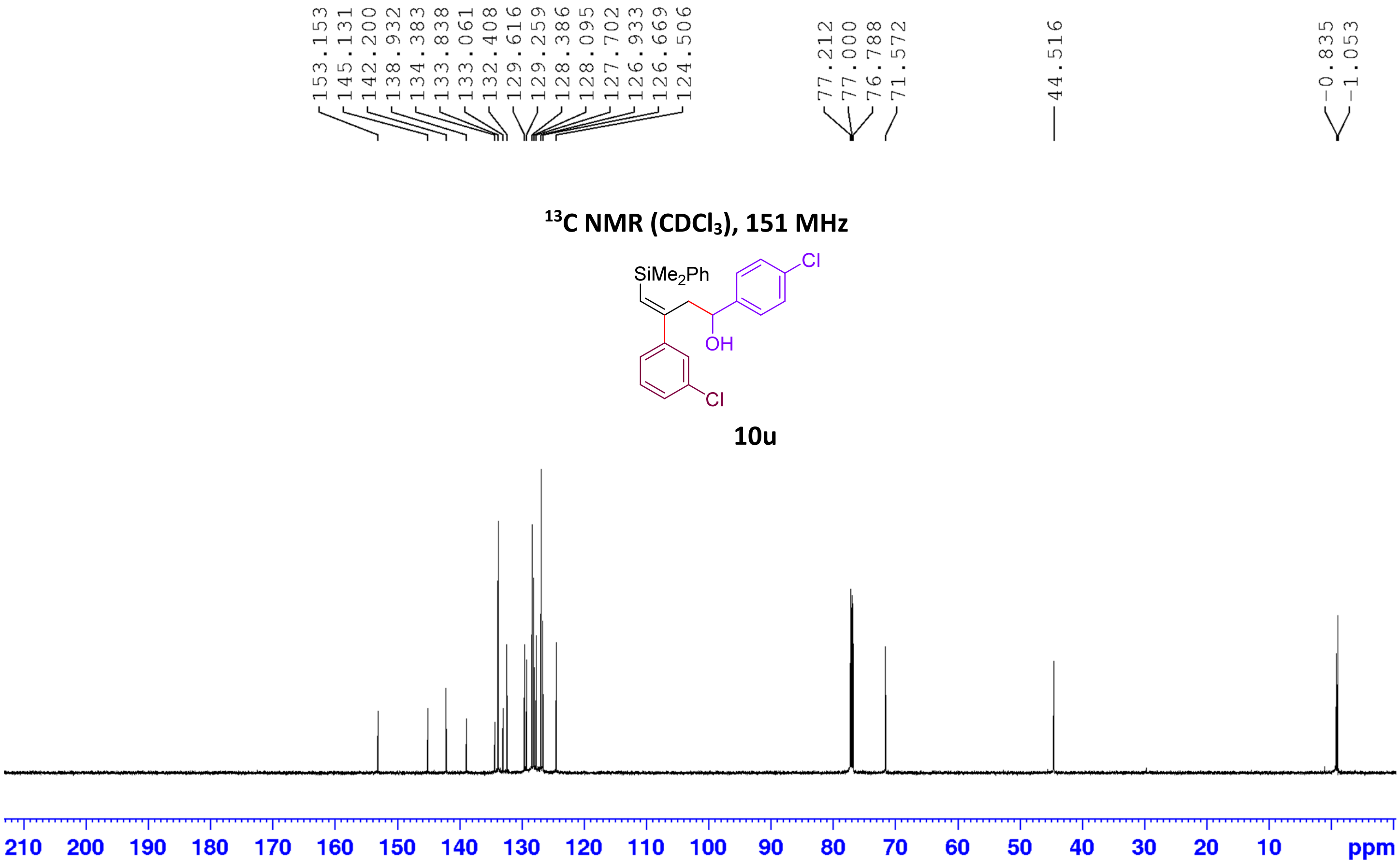

10u

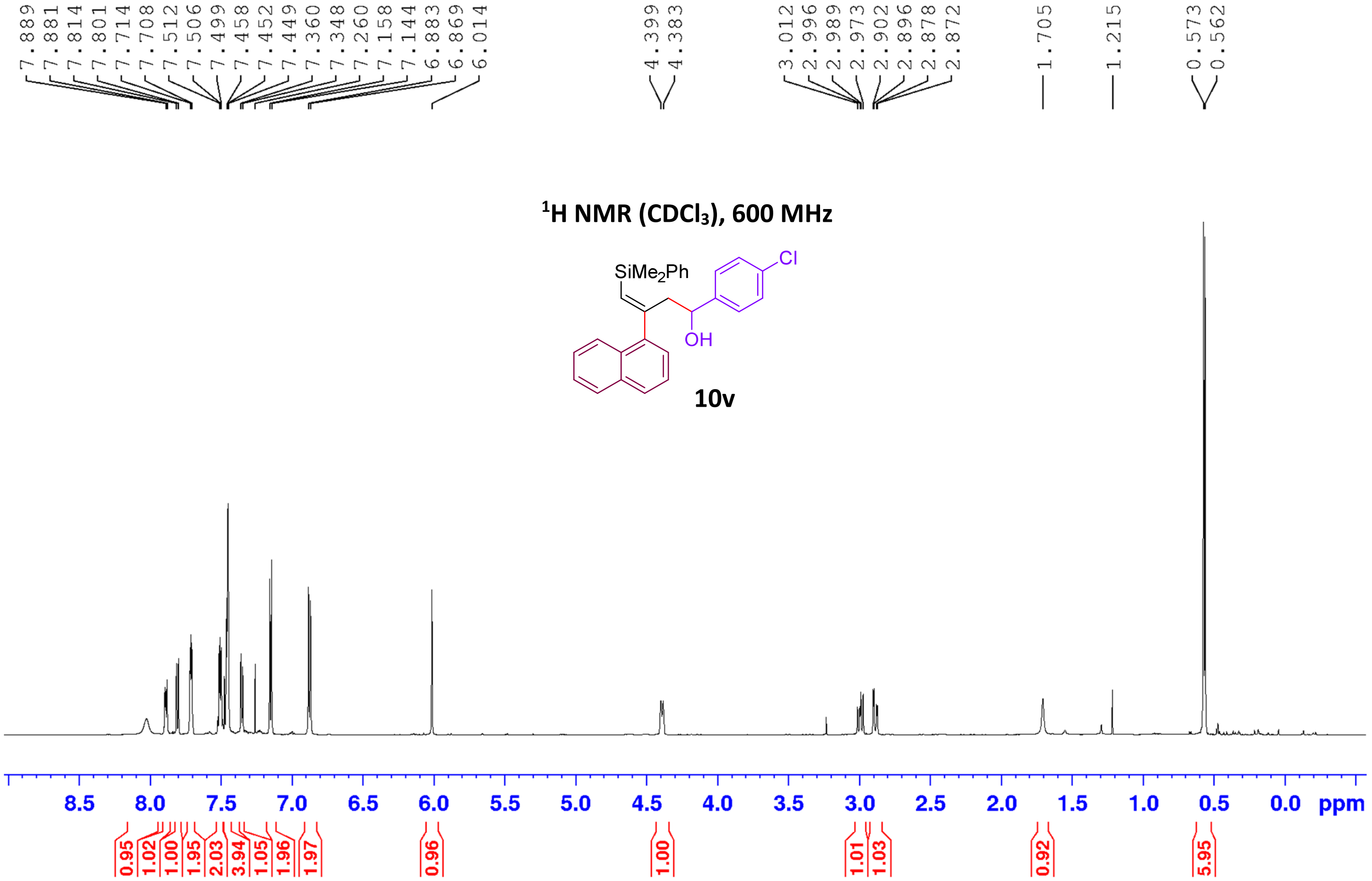
7.889
7.881
7.814
7.801
7.714
7.708
7.512
7.506
7.499
7.458
7.452
7.449
7.360
7.348
7.260
7.158
7.144
6.883
6.869
6.014
4.399
4.383
3.012
2.996
2.989
2.973
2.902
2.896
2.878
2.872
1.705
1.215
0.573
0.562
1H NMR (CDCl3), 600 MHz
SiMe2Ph
Cl
OH
10v
8.5
8.0
7.5
7.0
6.5
6.0
5.5
5.0
4.5
4.0
3.5
3.0
2.5
2.0
1.5
1.0
0.5
0.0
ppm
0.95
1.02
1.00
1.95
2.03
3.94
1.05
1.96
1.97
0.96
1.00
1.01
1.03
0.92
5.95

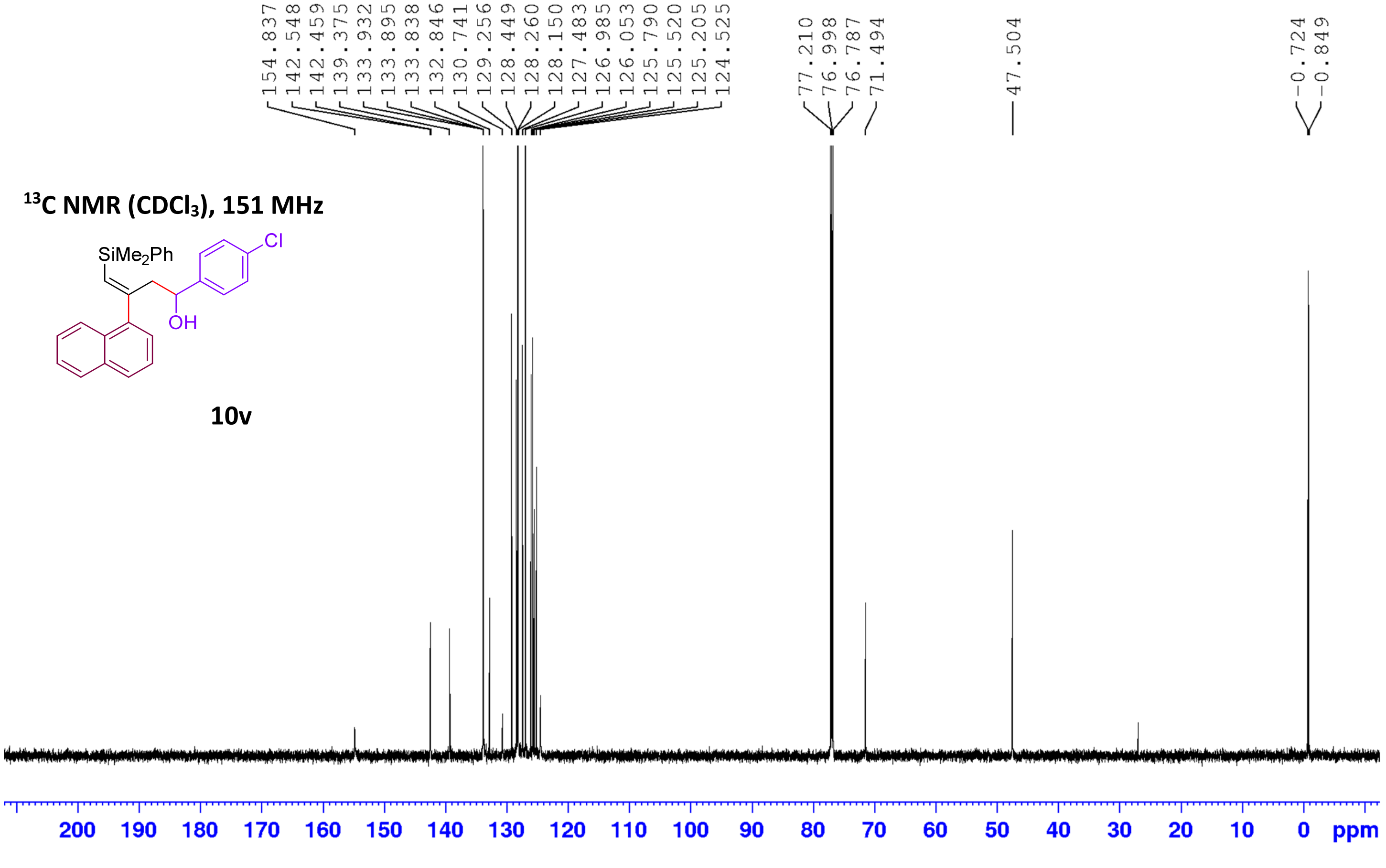
154.837
142.548
142.459
139.375
133.932
133.895
133.838
132.846
130.741
129.256
128.449
128.260
128.150
127.483
126.985
126.053
125.790
125.520
125.205
124.525
77.210
76.998
76.787
71.494
47.504
-0.724
-0.849
13C NMR (CDCl3), 151 MHz
SiMe2Ph
Cl
OH
10v
200
190
180
170
160
150
140
130
120
110
100
90
80
70
60
50
40
30
20
10
0
ppm

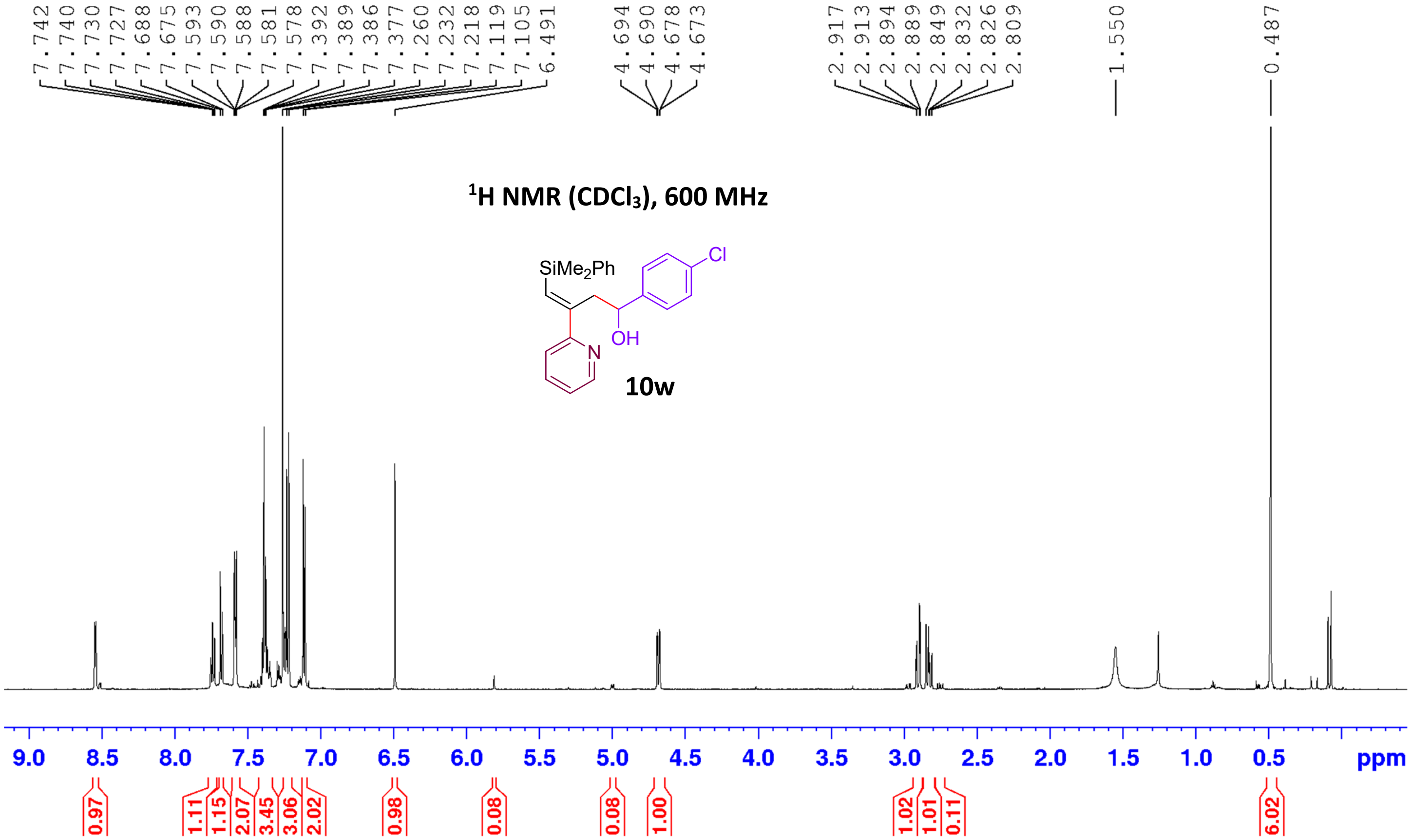
7.742
7.740
7.730
7.727
7.688
7.675
7.593
7.590
7.588
7.581
7.578
7.392
7.389
7.386
7.377
7.260
7.232
7.218
7.119
7.105
6.491
4.694
4.690
4.678
4.673
2.917
2.913
2.894
2.889
2.849
2.832
2.826
2.809
1.550
0.487
1H NMR (CDCl3), 600 MHz
SiMe2Ph
Cl
N
OH
10w
9.0 8.5 8.0 7.5 7.0 6.5 6.0 5.5 5.0 4.5 4.0 3.5 3.0 2.5 2.0 1.5 1.0 0.5 ppm
0.97
1.11
1.15
2.07
3.45
3.06
2.02
0.98
0.08
0.08
1.00
1.02
1.01
0.11
6.02

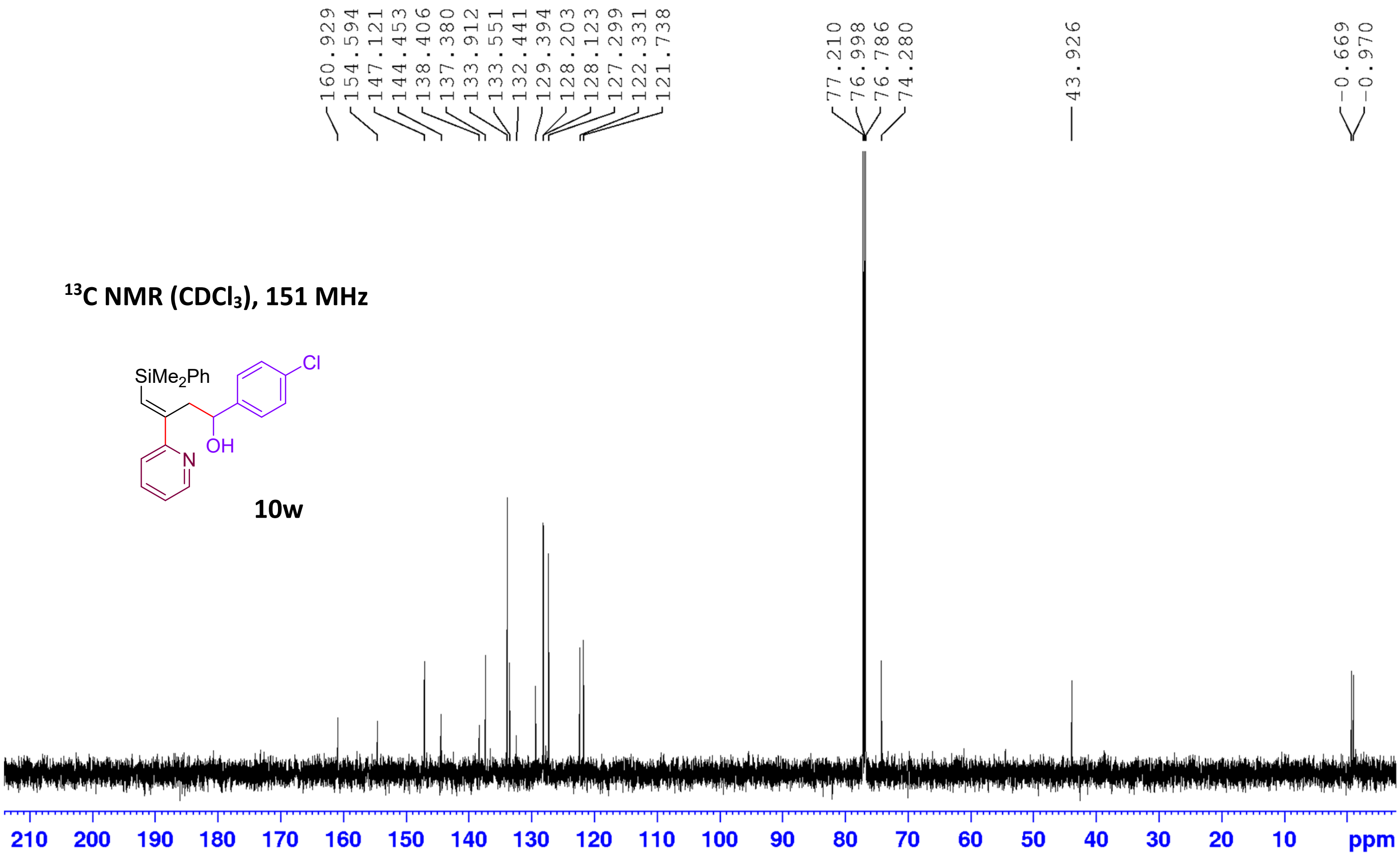
160.929
154.594
147.121
144.453
138.406
137.380
133.912
133.551
132.441
129.394
128.203
128.123
127.299
122.331
121.738
77.210
76.998
76.786
74.280
43.926
-0.669
-0.970
13C NMR (CDCl3), 151 MHz
SiMe2Ph
Cl
N
OH
10w
210 200 190 180 170 160 150 140 130 120 110 100 90 80 70 60 50 40 30 20 10 ppm

^{1}H NMR ($CDCl_3$), 600 MHz

10x

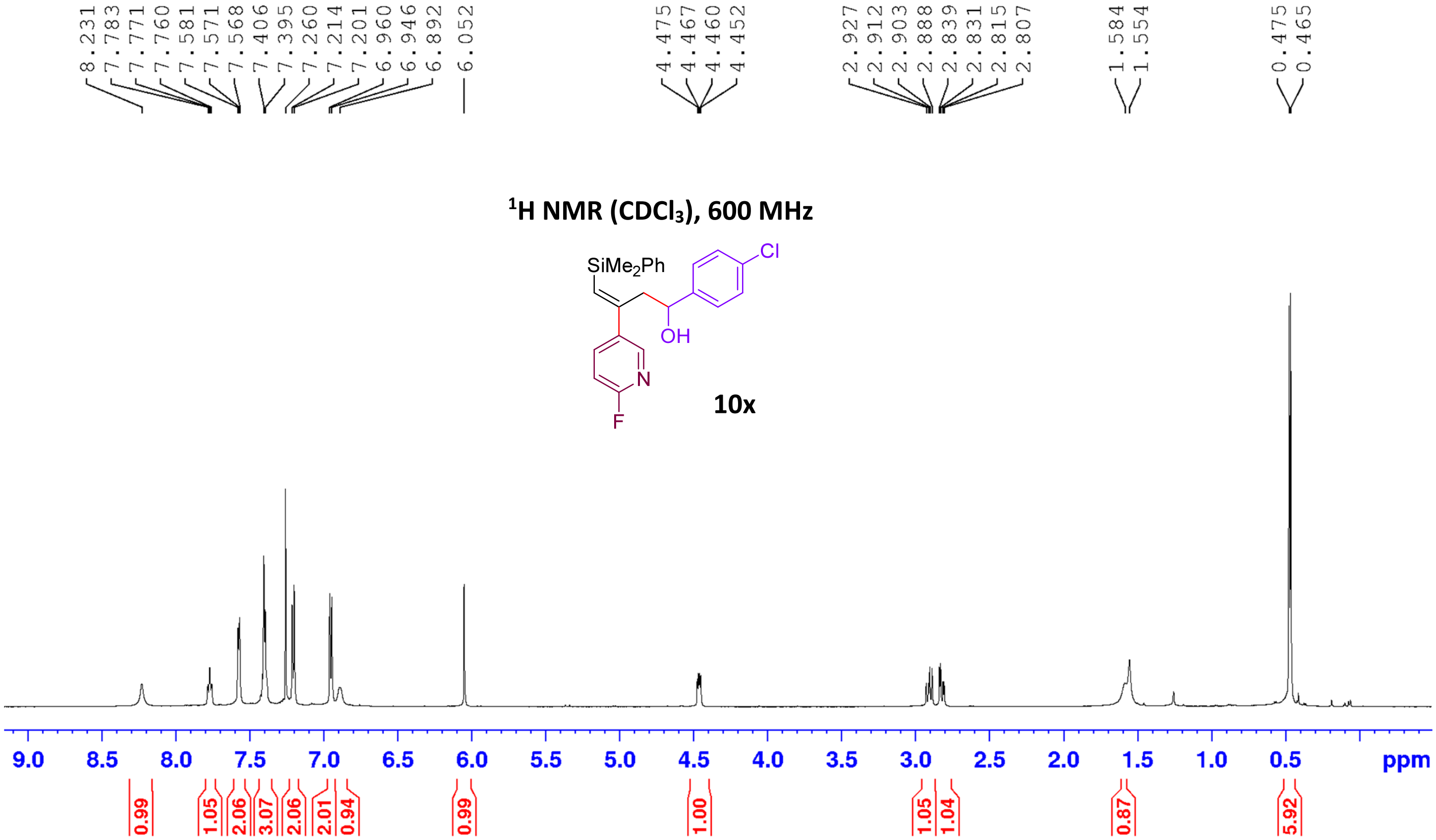

^{13}C NMR ($CDCl_3$), 151 MHz

10x

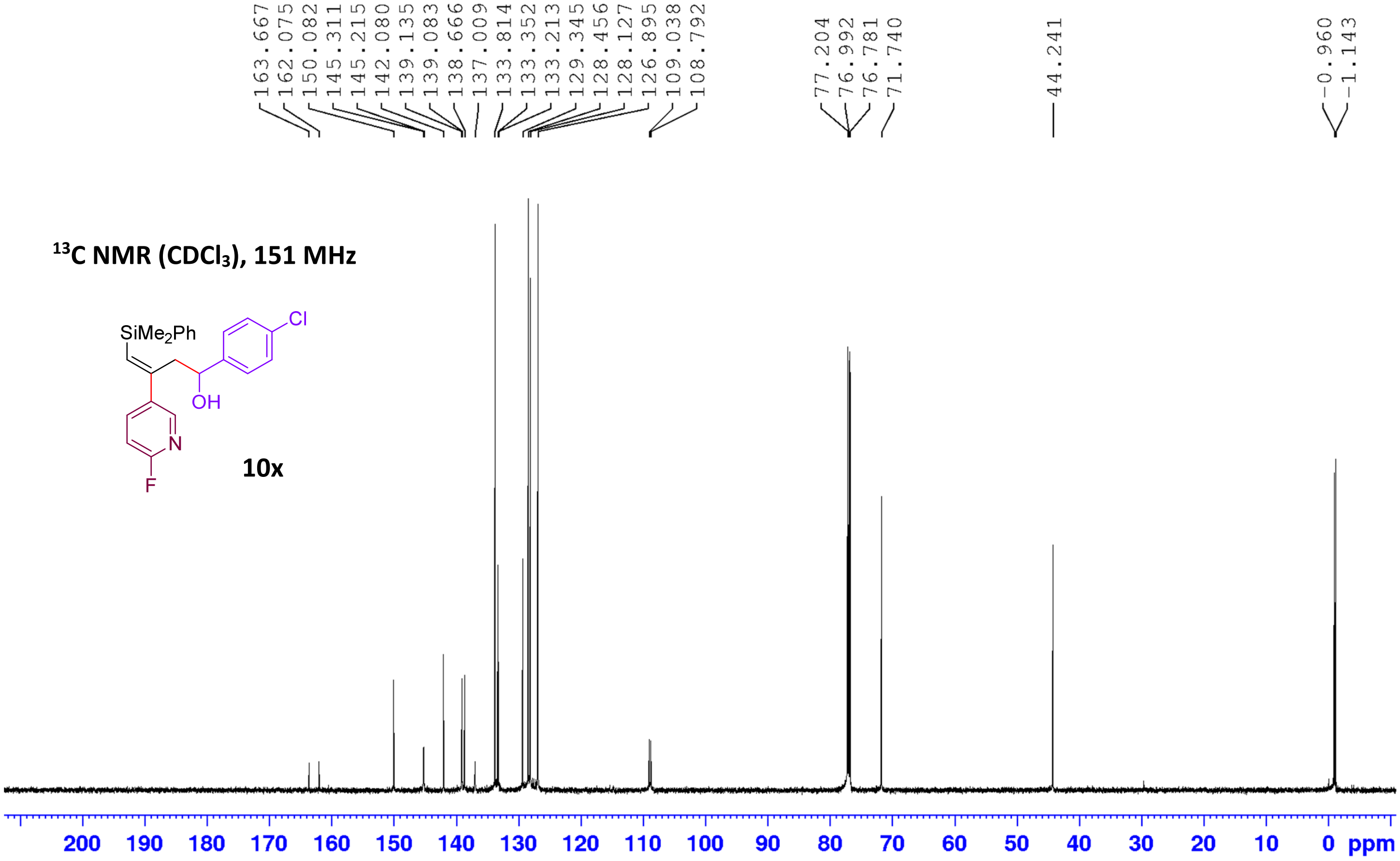

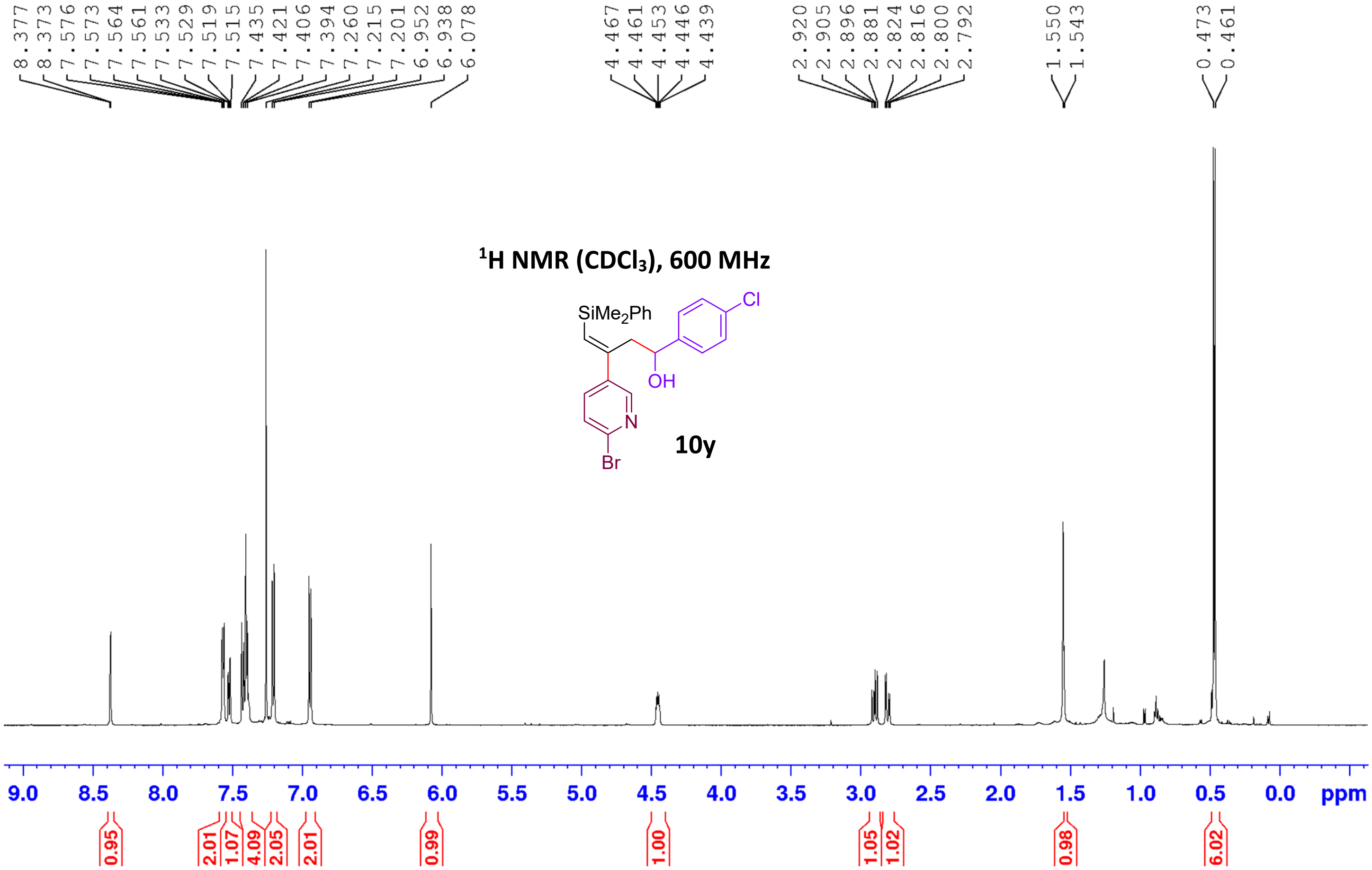

8.377
8.373
7.576
7.573
7.564
7.561
7.533
7.529
7.519
7.515
7.435
7.421
7.406
7.394
7.260
7.215
7.201
6.952
6.938
6.078
4.467
4.461
4.453
4.446
4.439
2.920
2.905
2.896
2.881
2.824
2.816
2.800
2.792
1.550
1.543
0.473
0.461
¹H NMR (CDCl₃), 600 MHz
SiMe₂Ph
Cl
OH
N
Br
10y
9.0 8.5 8.0 7.5 7.0 6.5 6.0 5.5 5.0 4.5 4.0 3.5 3.0 2.5 2.0 1.5 1.0 0.5 0.0 ppm
0.95
2.01
1.07
4.09
2.05
2.01
0.99
1.00
1.05
1.02
0.98
6.02

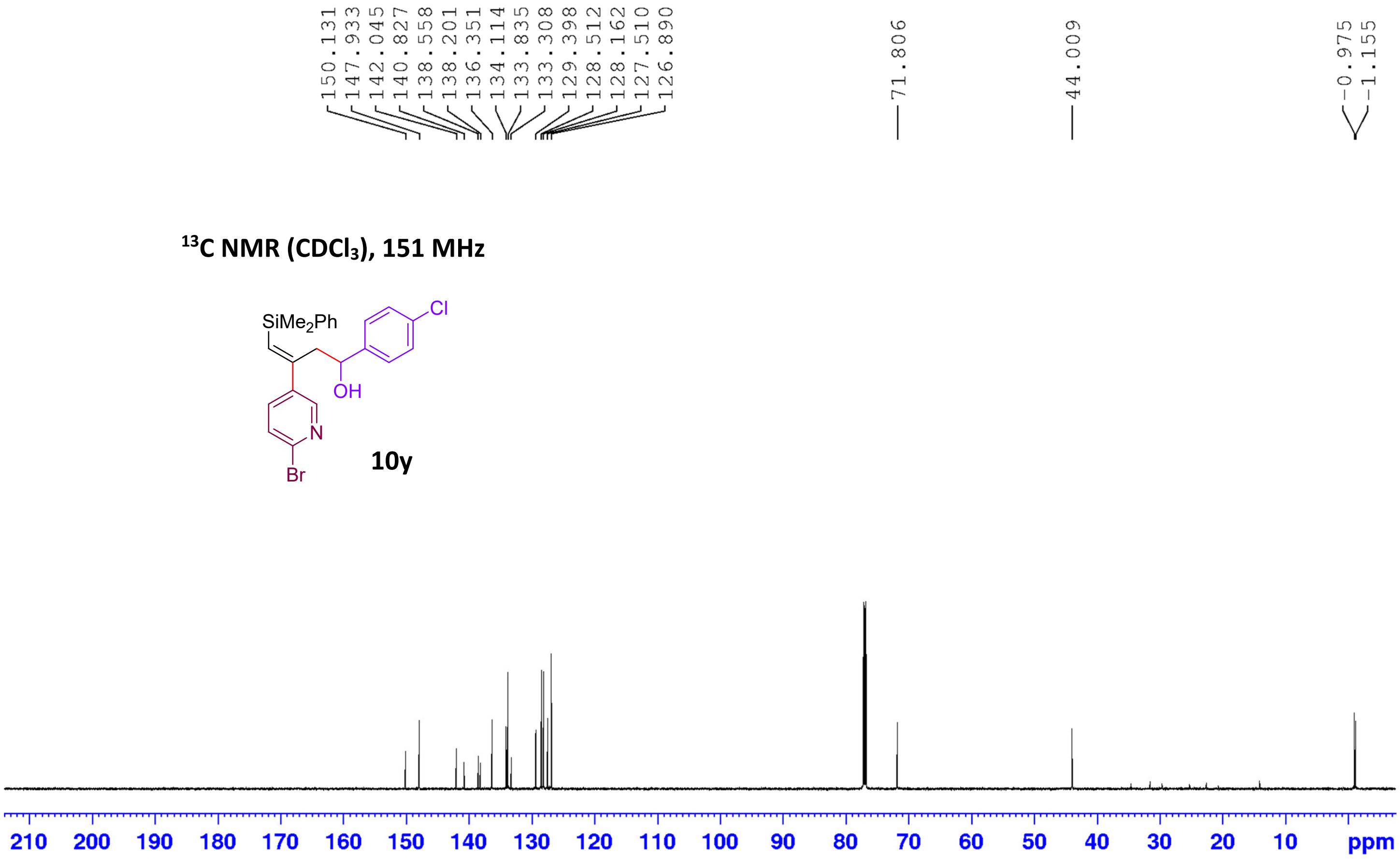

150.131
147.933
142.045
140.827
138.558
138.201
136.351
134.114
133.835
133.308
129.398
128.512
128.162
127.510
126.890
71.806
44.009
-0.975
-1.155
¹³C NMR (CDCl₃), 151 MHz
SiMe₂Ph
Cl
OH
N
Br
10y
210 200 190 180 170 160 150 140 130 120 110 100 90 80 70 60 50 40 30 20 10 ppm

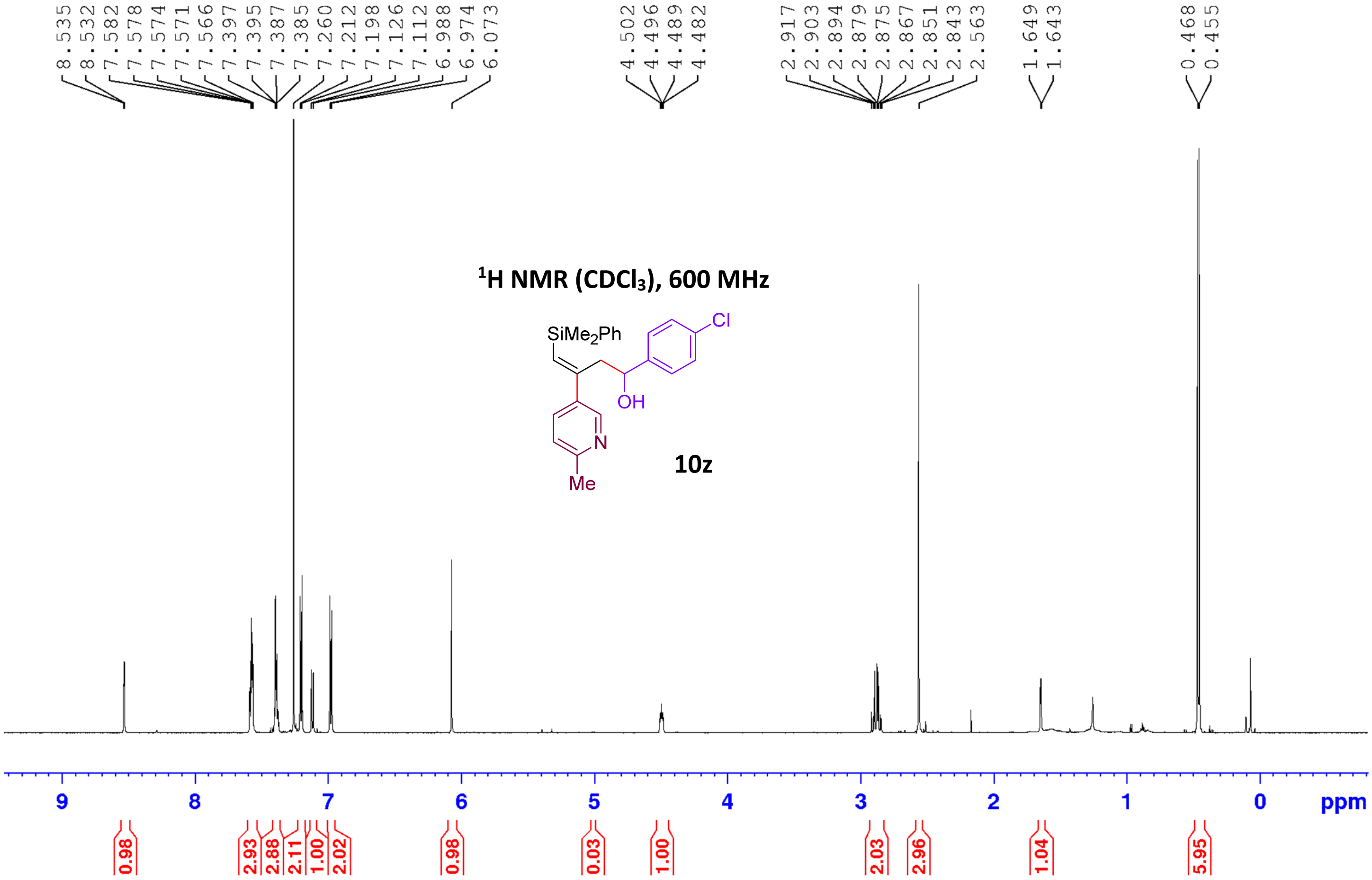

8.535 8.532 7.582 7.578 7.574 7.571 7.566 7.397 7.395 7.387 7.385 7.260 7.212 7.198 7.126 7.112 6.988 6.974 6.073
4.502 4.496 4.489 4.482
2.917 2.903 2.894 2.879 2.875 2.867 2.851 2.843 2.563
1.649 1.643
0.468 0.455
1H NMR (CDCl3), 600 MHz
SiMe2Ph
Cl
OH
N
Me
10z
9 8 7 6 5 4 3 2 1 0 ppm
0.98 2.93 2.88 2.11 1.00 2.02 0.98 0.03 1.00 2.03 2.96 1.04 5.95

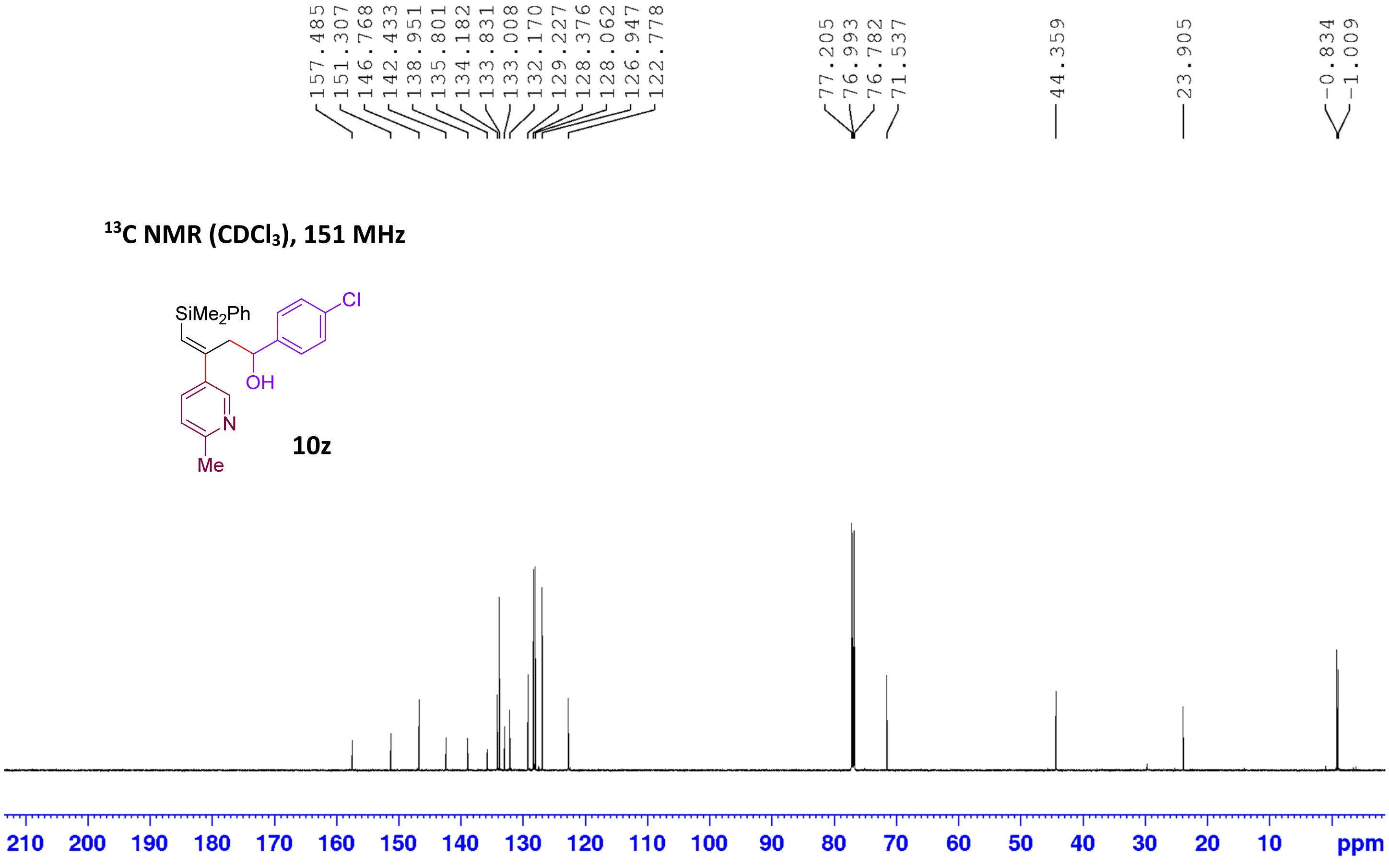

157.485 151.307 146.768 142.433 138.951 135.801 134.182 133.831 133.008 132.170 129.227 128.376 128.062 126.947 122.778
77.205 76.993 76.782 71.537
44.359
23.905
-0.834 -1.009
13C NMR (CDCl3), 151 MHz
SiMe2Ph
Cl
OH
N
Me
10z
210 200 190 180 170 160 150 140 130 120 110 100 90 80 70 60 50 40 30 20 10 ppm

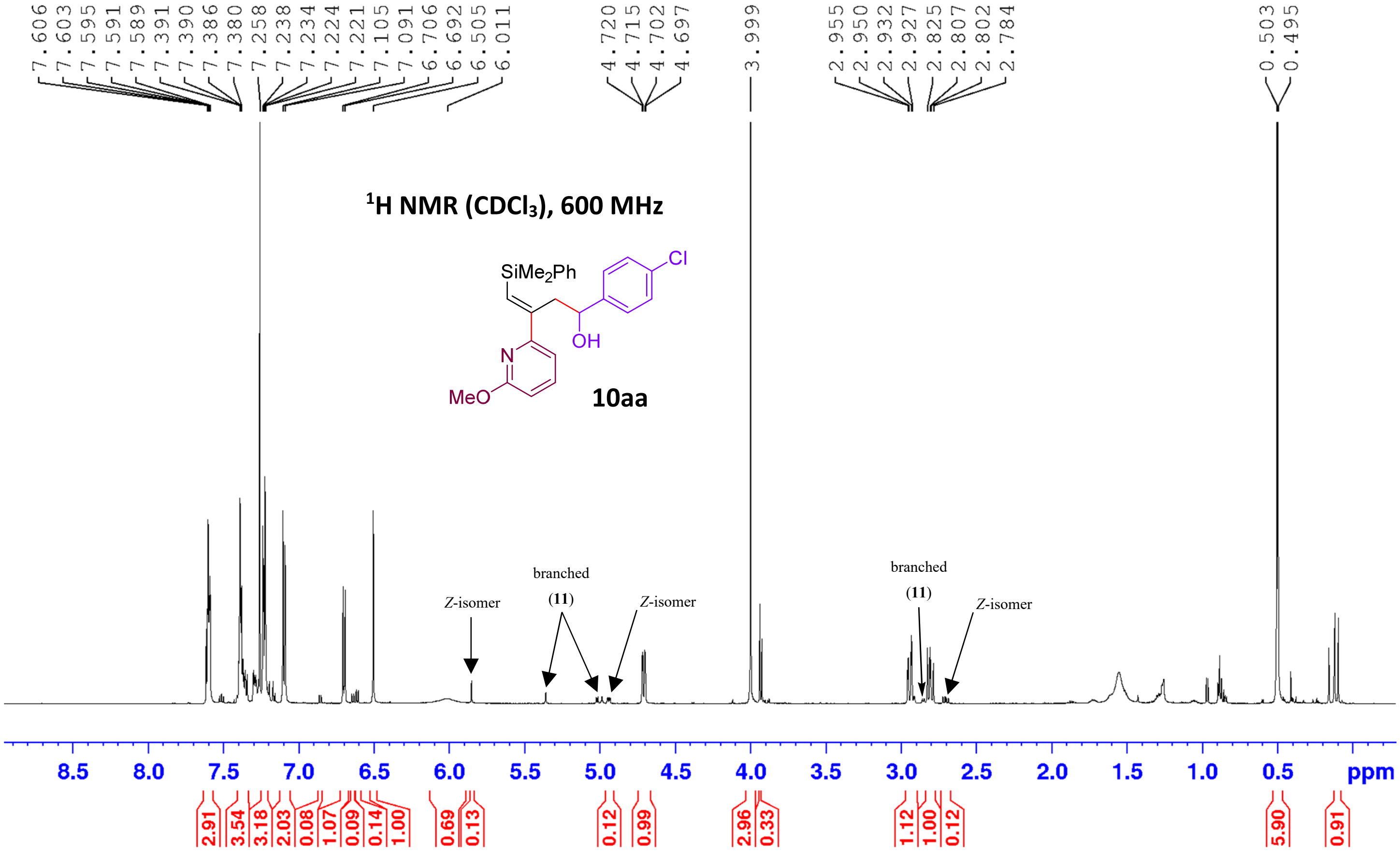
7.606 7.603 7.595 7.591 7.589 7.391 7.390 7.386 7.380 7.258 7.238 7.234 7.224 7.221 7.105 7.091 6.706 6.692 6.505 6.011
4.720 4.715 4.702 4.697
3.999
2.955 2.950 2.932 2.927 2.825 2.807 2.802 2.784
0.503 0.495
1H NMR (CDCl3), 600 MHz
SiMe2Ph
Cl
OH
N
MeO
10aa
Z-isomer
branched
(11)
Z-isomer
branched
(11)
Z-isomer
8.5 8.0 7.5 7.0 6.5 6.0 5.5 5.0 4.5 4.0 3.5 3.0 2.5 2.0 1.5 1.0 0.5 ppm
2.91 3.54 3.18 2.03 0.08 1.07 0.09 0.14 1.00 0.69 0.13 0.12 0.99 2.96 0.33 1.12 1.00 0.12 5.90 0.91

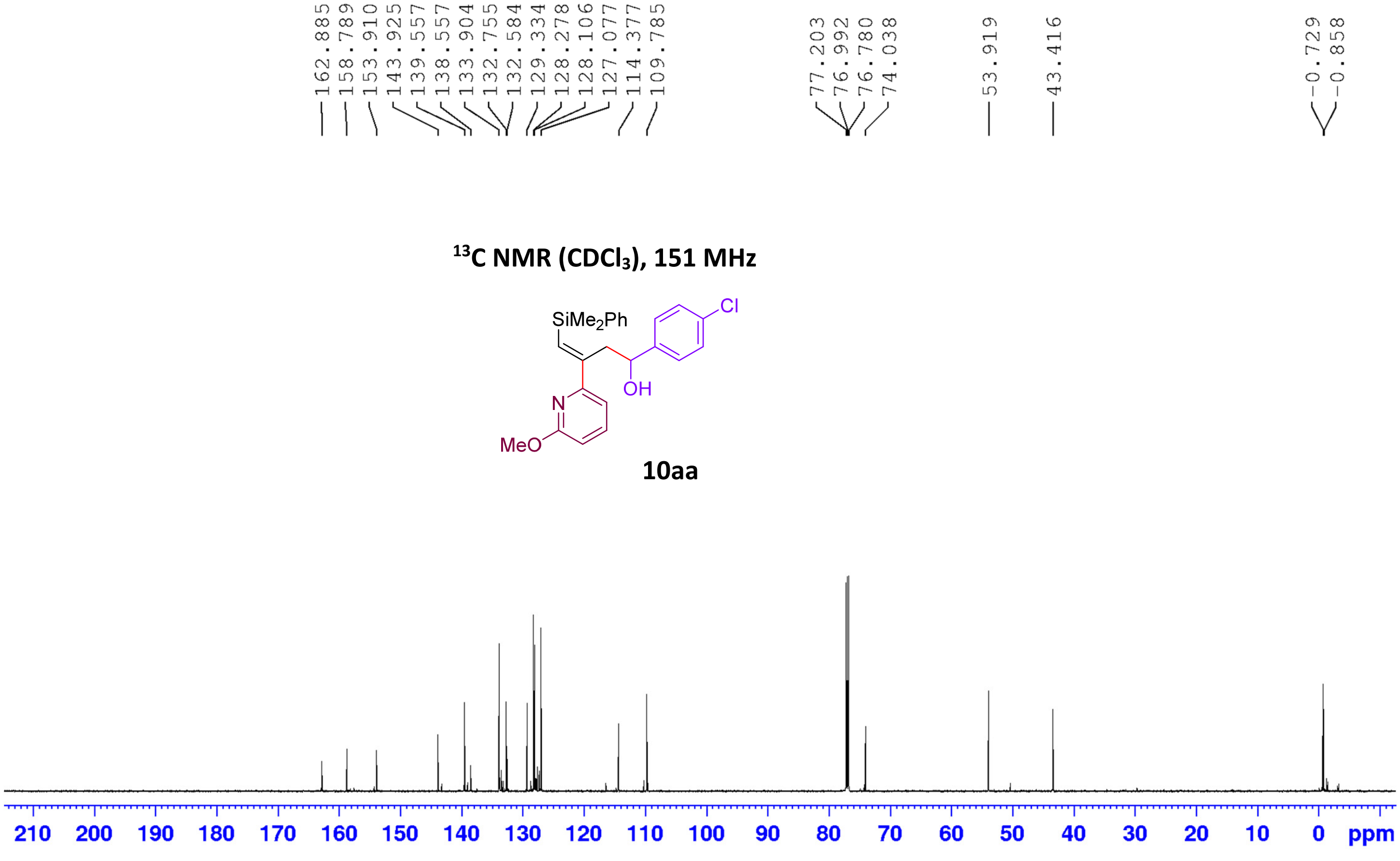
162.885 158.789 153.910 143.925 139.557 138.557 133.904 132.755 132.584 129.334 128.278 128.106 127.077 114.377 109.785
77.203 76.992 76.780 74.038
53.919
43.416
-0.729 -0.858
13C NMR (CDCl3), 151 MHz
SiMe2Ph
Cl
OH
N
MeO
10aa
210 200 190 180 170 160 150 140 130 120 110 100 90 80 70 60 50 40 30 20 10 0 ppm

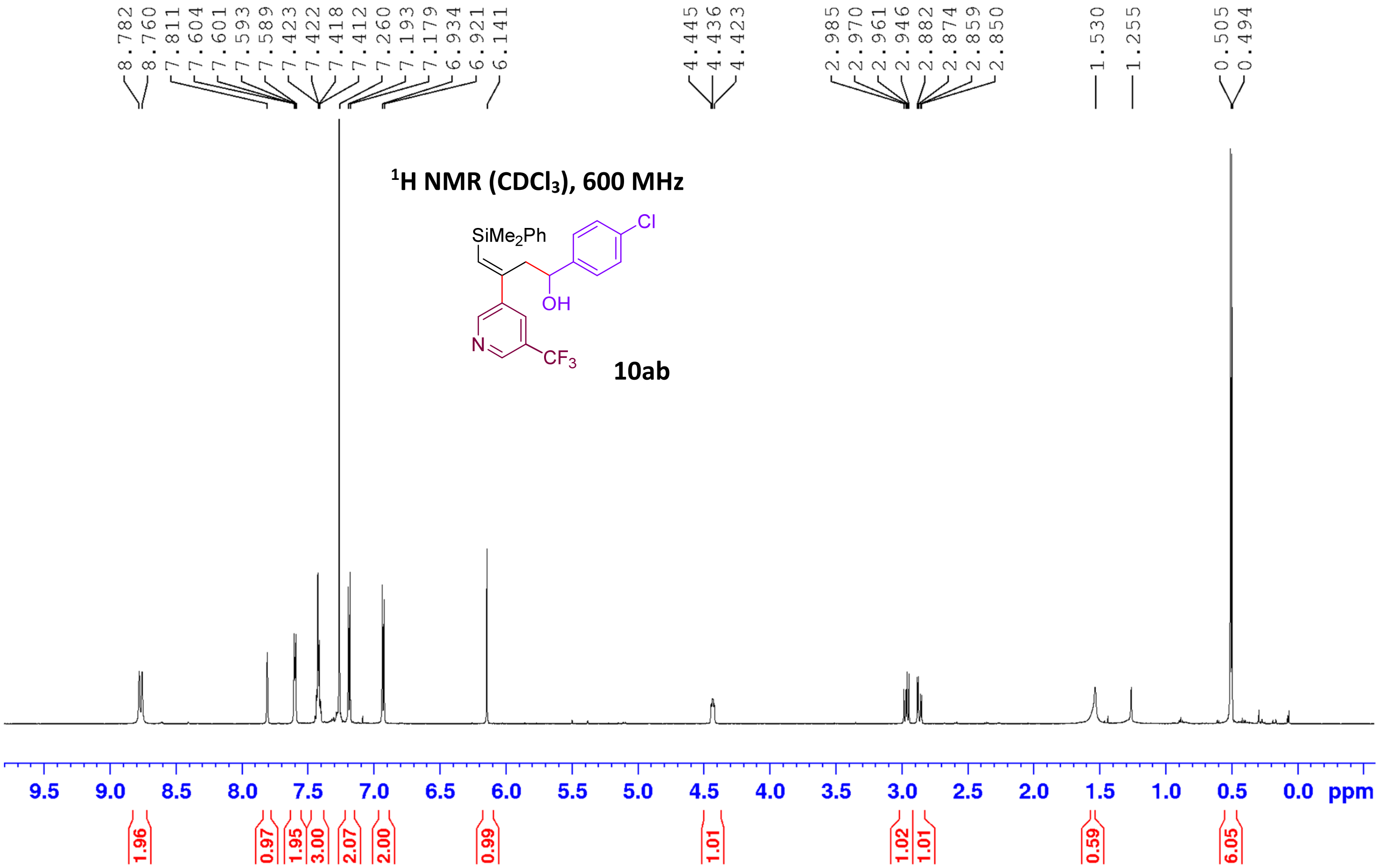
8.782
8.760
7.811
7.604
7.601
7.593
7.589
7.423
7.422
7.418
7.412
7.260
7.193
7.179
6.934
6.921
6.141
4.445
4.436
4.423
2.985
2.970
2.961
2.946
2.882
2.874
2.859
2.850
1.530
1.255
0.505
0.494
^{1}H NMR (CDCl$_3$), 600 MHz
SiMe$_2$Ph
Cl
OH
N
CF$_3$
10ab
9.5 9.0 8.5 8.0 7.5 7.0 6.5 6.0 5.5 5.0 4.5 4.0 3.5 3.0 2.5 2.0 1.5 1.0 0.5 0.0 ppm
1.96
0.97
1.95
3.00
2.07
2.00
0.99
1.01
1.02
1.01
0.59
6.05

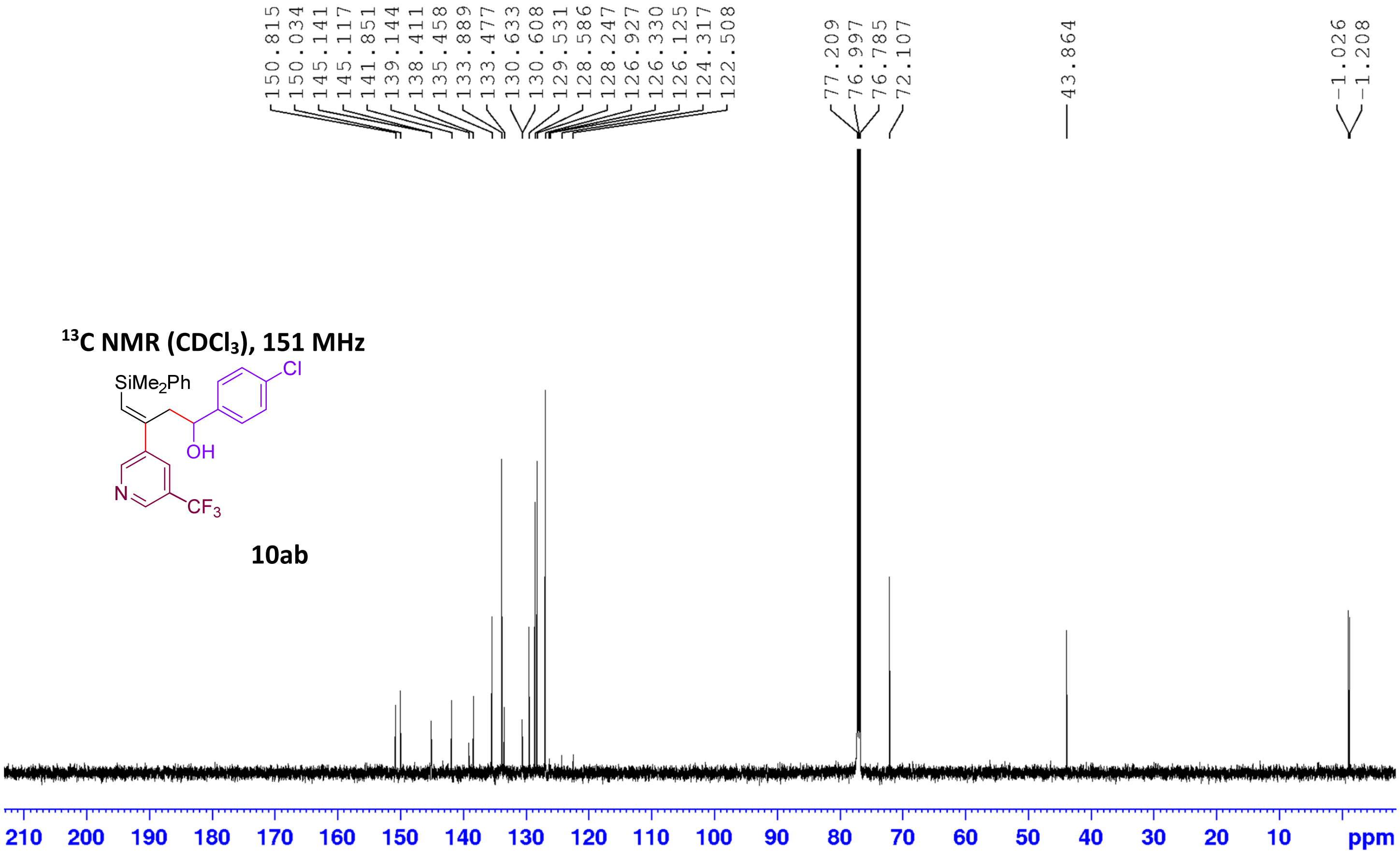
150.815
150.034
145.141
145.117
141.851
139.144
138.411
135.458
133.889
133.477
130.633
130.608
129.531
128.586
128.247
126.927
126.330
126.125
124.317
122.508
77.209
76.997
76.785
72.107
43.864
-1.026
-1.208
^{13}C NMR (CDCl$_3$), 151 MHz
SiMe$_2$Ph
Cl
OH
N
CF$_3$
10ab
210 200 190 180 170 160 150 140 130 120 110 100 90 80 70 60 50 40 30 20 10 ppm

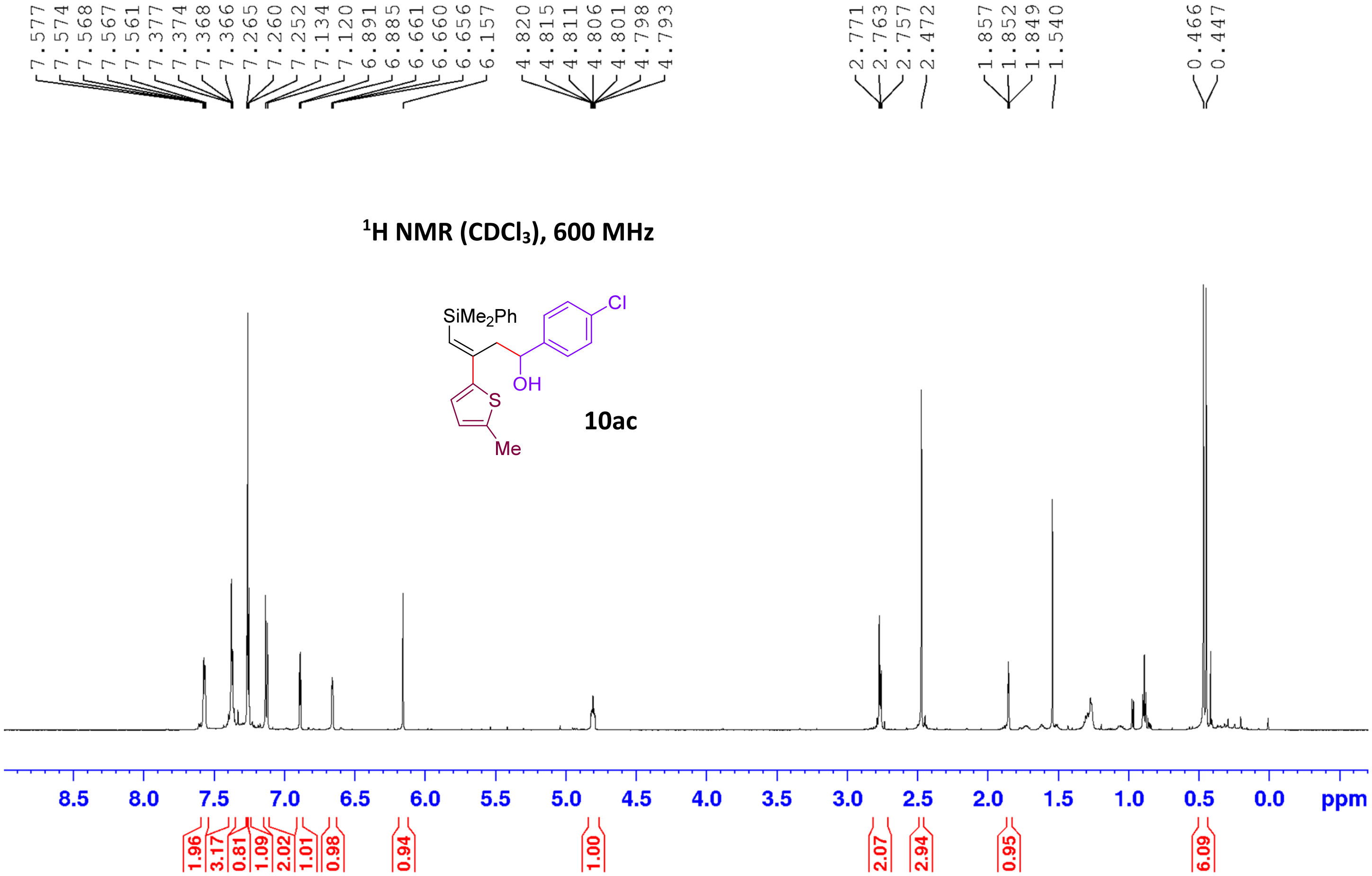
7.577 7.574 7.568 7.567 7.561 7.377 7.374 7.368 7.366 7.265 7.260 7.252 7.134 7.120 6.891 6.885 6.661 6.660 6.656 6.157 4.820 4.815 4.811 4.806 4.801 4.798 4.793 2.771 2.763 2.757 2.472 1.857 1.852 1.849 1.540 0.466 0.447
^{1}H NMR (CDCl$_3$), 600 MHz
SiMe$_2$Ph
Cl
OH
S
Me
10ac
8.5 8.0 7.5 7.0 6.5 6.0 5.5 5.0 4.5 4.0 3.5 3.0 2.5 2.0 1.5 1.0 0.5 0.0 ppm
1.96 3.17 0.81 1.09 2.02 1.01 0.98 0.94 1.00 2.07 2.94 0.95 6.09

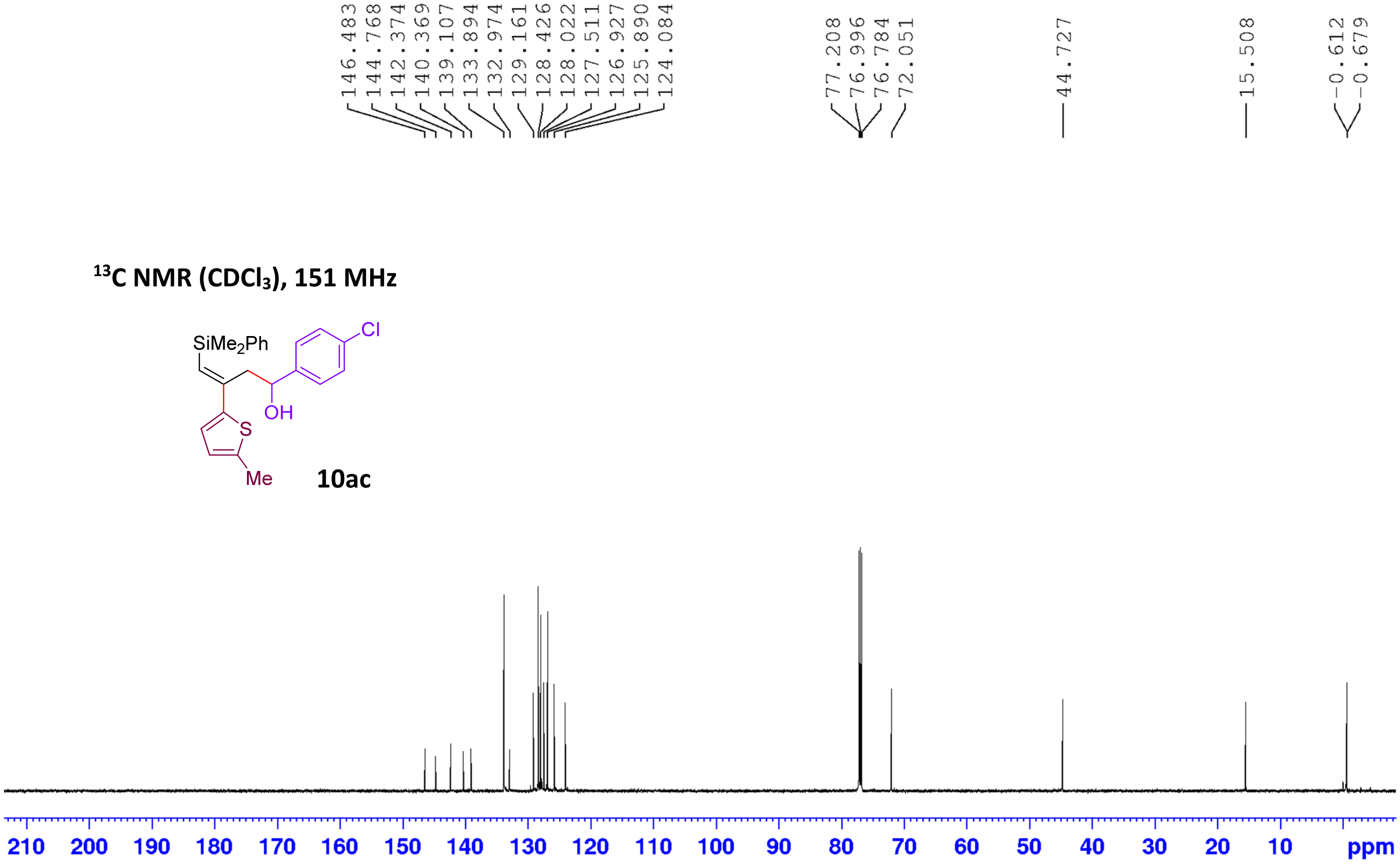
146.483 144.768 142.374 140.369 139.107 133.894 132.974 129.161 128.426 128.022 127.511 126.927 125.890 124.084 77.208 76.996 76.784 72.051 44.727 15.508 -0.612 -0.679
^{13}C NMR (CDCl$_3$), 151 MHz
SiMe$_2$Ph
Cl
OH
S
Me
10ac
210 200 190 180 170 160 150 140 130 120 110 100 90 80 70 60 50 40 30 20 10 ppm

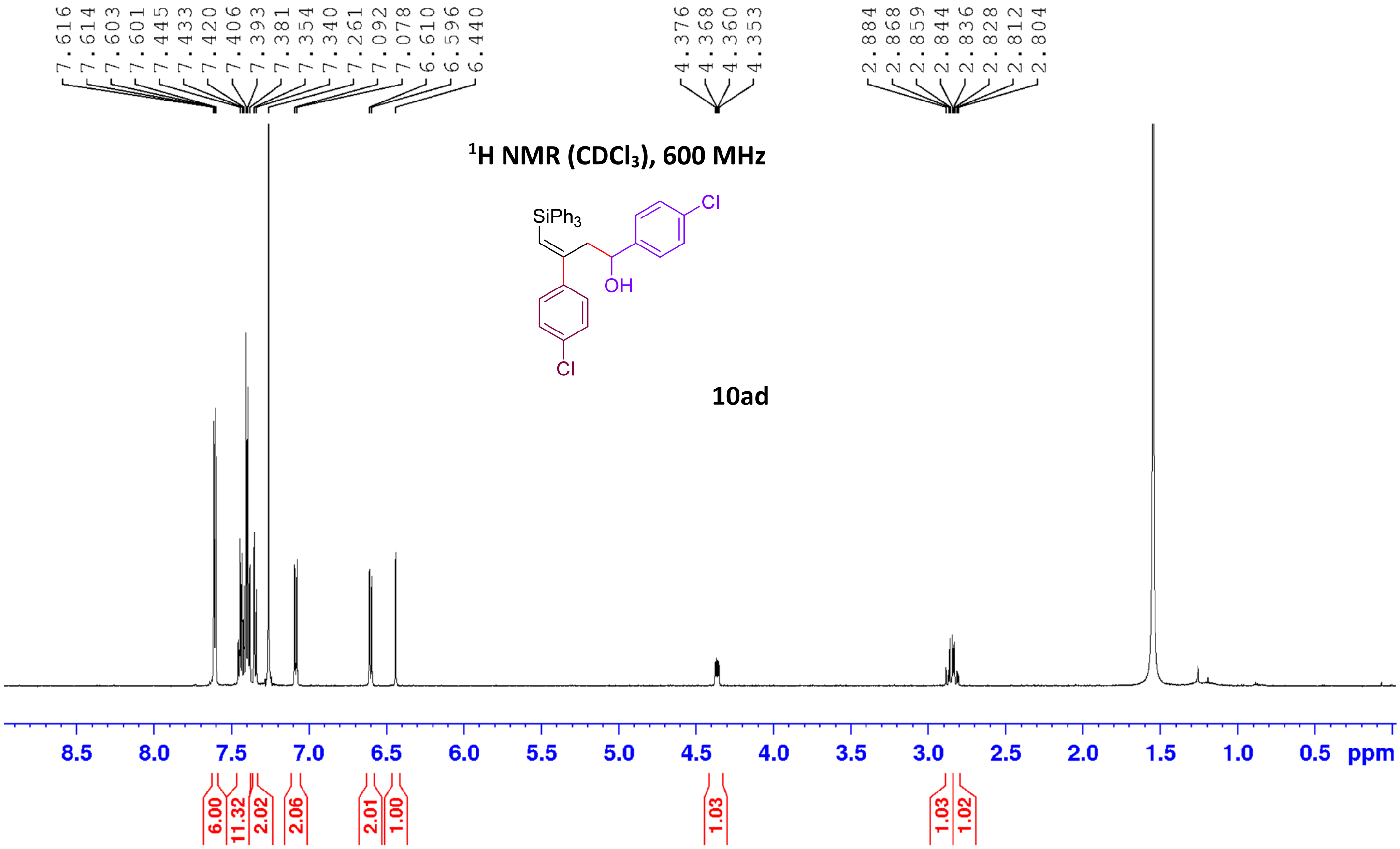

7.616
7.614
7.603
7.601
7.445
7.433
7.420
7.406
7.393
7.381
7.354
7.340
7.261
7.092
7.078
6.610
6.596
6.440
4.376
4.368
4.360
4.353
2.884
2.868
2.859
2.844
2.836
2.828
2.812
2.804
^{1}H NMR (CDCl$_3$), 600 MHz
SiPh$_3$
Cl
OH
Cl
10ad
8.5
8.0
7.5
7.0
6.5
6.0
5.5
5.0
4.5
4.0
3.5
3.0
2.5
2.0
1.5
1.0
0.5
ppm
6.00
11.32
2.02
2.06
2.01
1.00
1.03
1.03
1.02

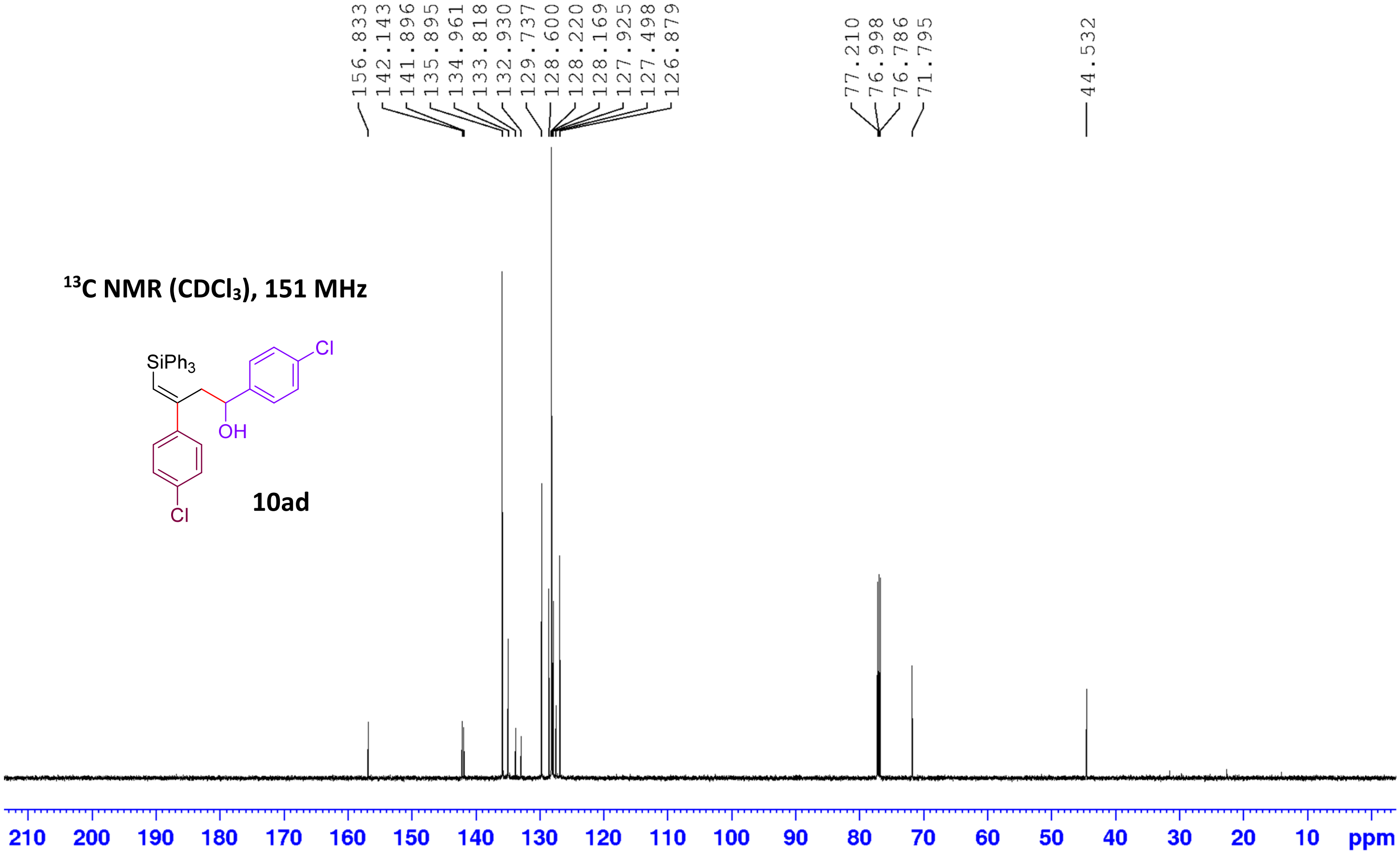

156.833
142.143
141.896
135.895
134.961
133.818
132.930
129.737
128.600
128.220
128.169
127.925
127.498
126.879
77.210
76.998
76.786
71.795
44.532
^{13}C NMR (CDCl$_3$), 151 MHz
SiPh$_3$
Cl
OH
Cl
10ad
210
200
190
180
170
160
150
140
130
120
110
100
90
80
70
60
50
40
30
20
10
ppm

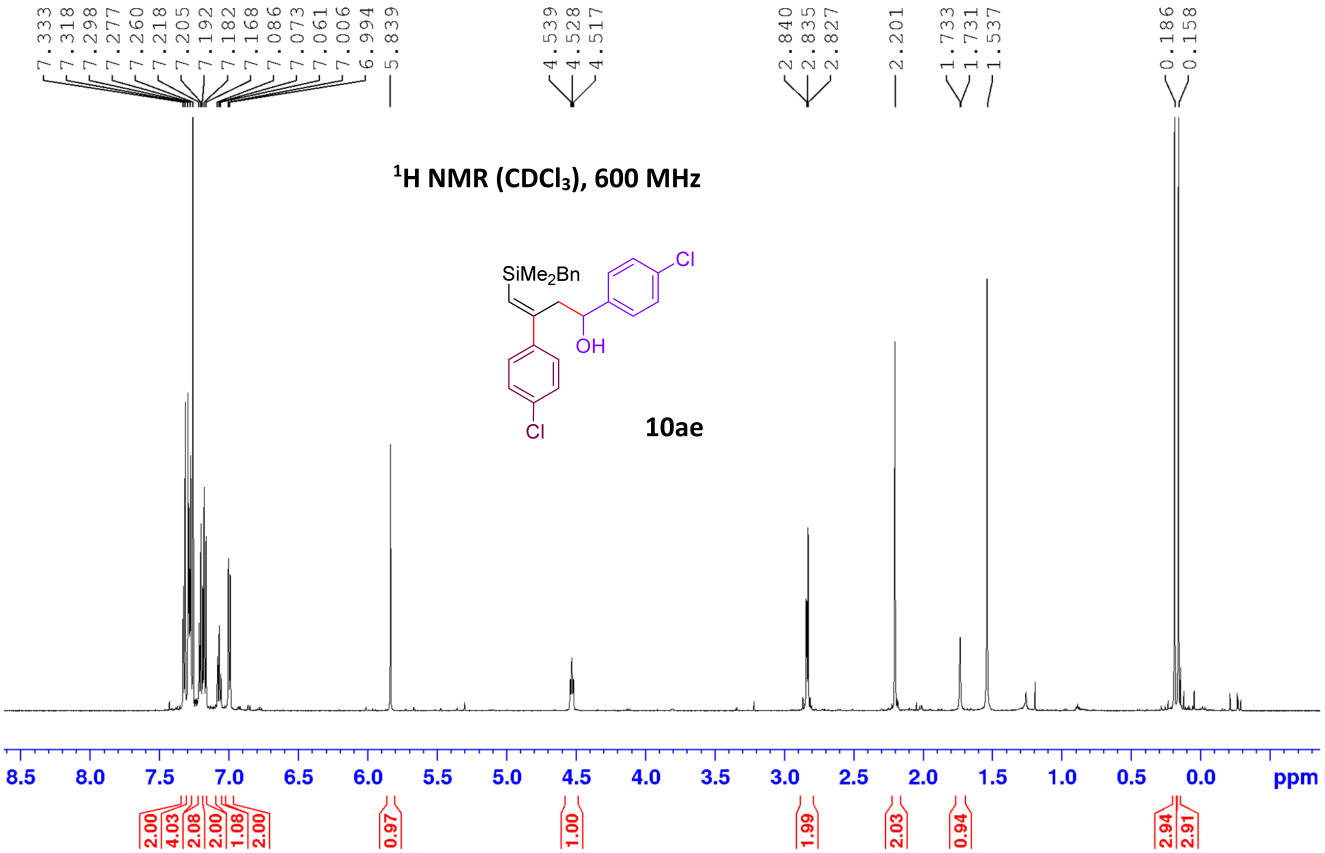

7.333
7.318
7.298
7.277
7.260
7.218
7.205
7.192
7.182
7.168
7.086
7.073
7.061
7.006
6.994
5.839
4.539
4.528
4.517
2.840
2.835
2.827
2.201
1.733
1.731
1.537
0.186
0.158
1H NMR (CDCl3), 600 MHz
SiMe2Bn
Cl
OH
Cl
10ae
8.5 8.0 7.5 7.0 6.5 6.0 5.5 5.0 4.5 4.0 3.5 3.0 2.5 2.0 1.5 1.0 0.5 0.0 ppm
2.00 4.03 2.08 2.00 1.08 2.00 0.97 1.00 1.99 2.03 0.94 2.94 2.91

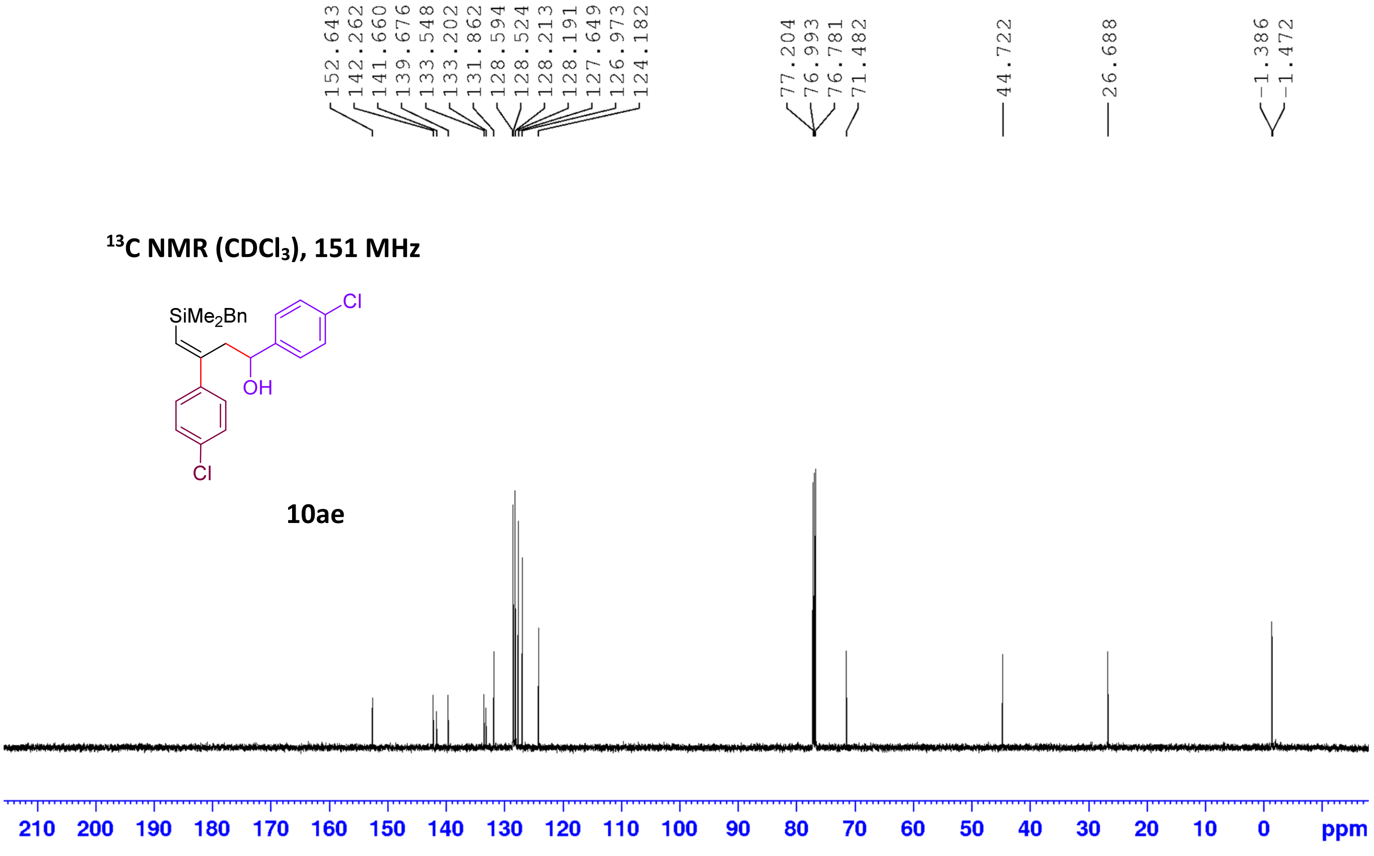

152.643
142.262
141.660
139.676
133.548
133.202
131.862
128.594
128.524
128.213
128.191
127.649
126.973
124.182
77.204
76.993
76.781
71.482
44.722
26.688
-1.386
-1.472
13C NMR (CDCl3), 151 MHz
SiMe2Bn
Cl
OH
Cl
10ae
210 200 190 180 170 160 150 140 130 120 110 100 90 80 70 60 50 40 30 20 10 0 ppm

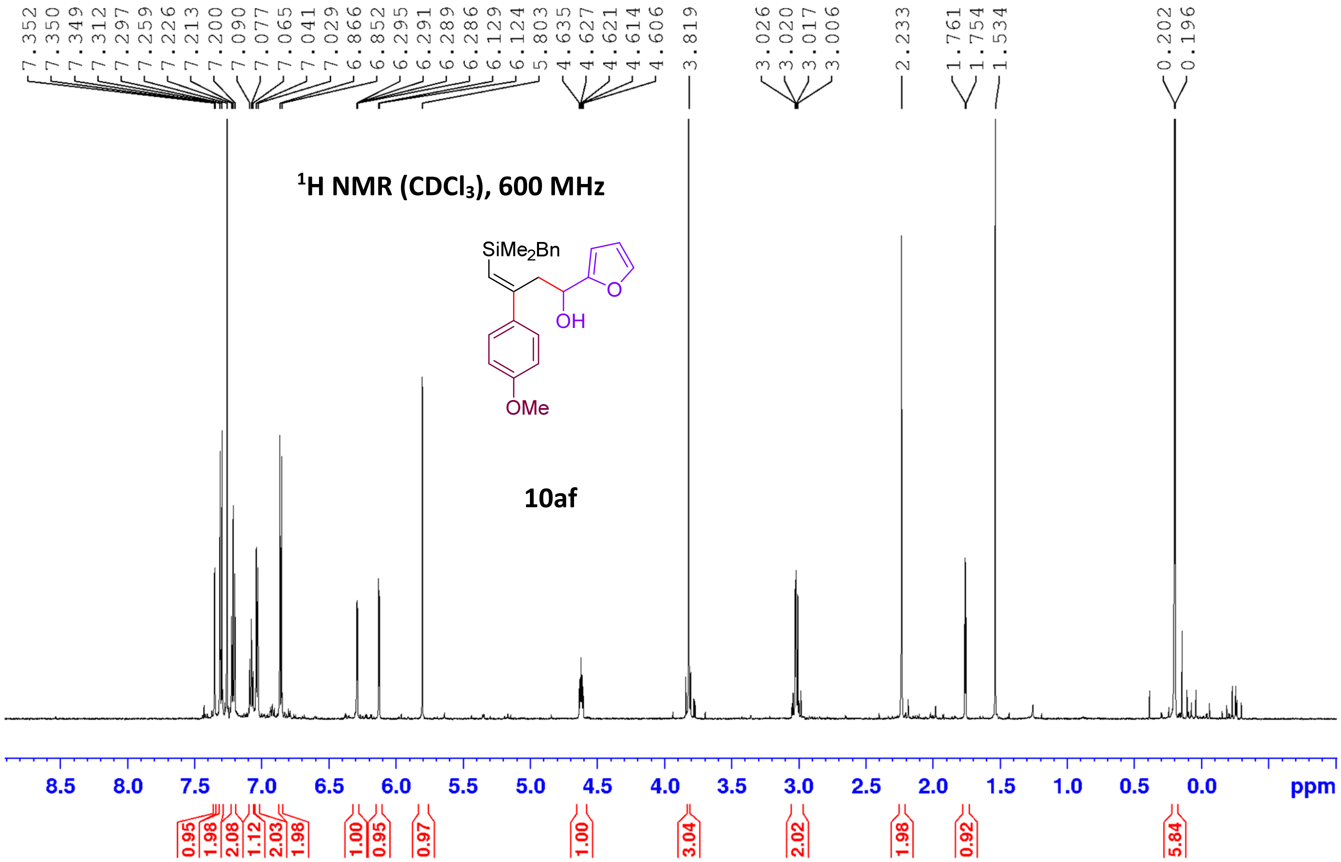

7.352 7.350 7.349 7.312 7.297 7.259 7.226 7.213 7.200 7.090 7.077 7.065 7.041 7.029 6.866 6.852 6.295 6.291 6.289 6.286 6.129 6.124 5.803 4.635 4.627 4.621 4.614 4.606 3.819 3.026 3.020 3.017 3.006 2.233 1.761 1.754 1.534 0.202 0.196
^{1}H NMR (CDCl$_3$), 600 MHz
SiMe$_2$Bn
O
OH
OMe
10af
8.5 8.0 7.5 7.0 6.5 6.0 5.5 5.0 4.5 4.0 3.5 3.0 2.5 2.0 1.5 1.0 0.5 0.0 ppm
0.95 1.98 2.08 1.12 2.03 1.98 1.00 0.95 0.97 1.00 3.04 2.02 1.98 0.92 5.84

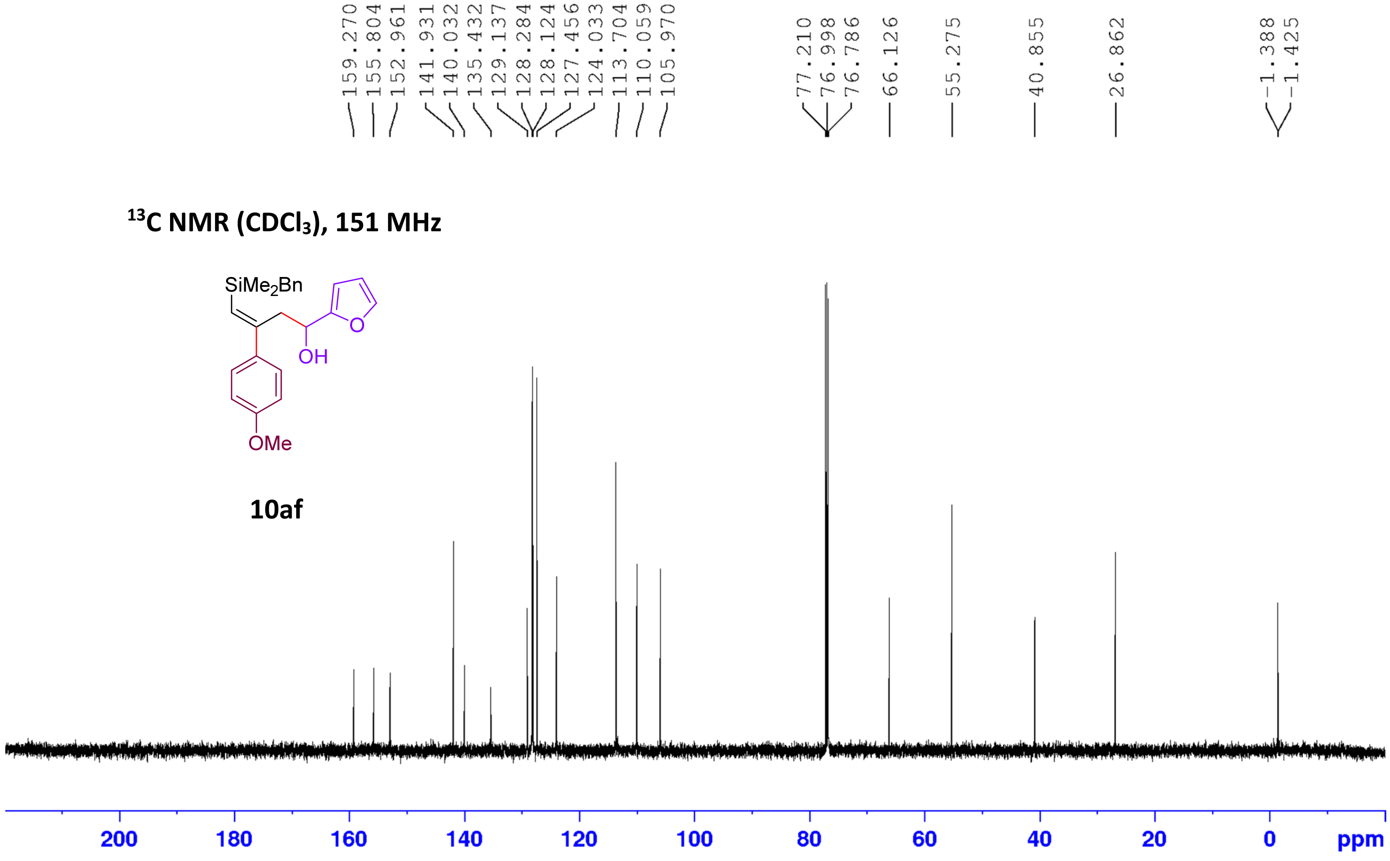

159.270 155.804 152.961 141.931 140.032 135.432 129.137 128.284 128.124 127.456 124.033 113.704 110.059 105.970 77.210 76.998 76.786 66.126 55.275 40.855 26.862 -1.388 -1.425
^{13}C NMR (CDCl$_3$), 151 MHz
SiMe$_2$Bn
O
OH
OMe
10af
200 180 160 140 120 100 80 60 40 20 0 ppm

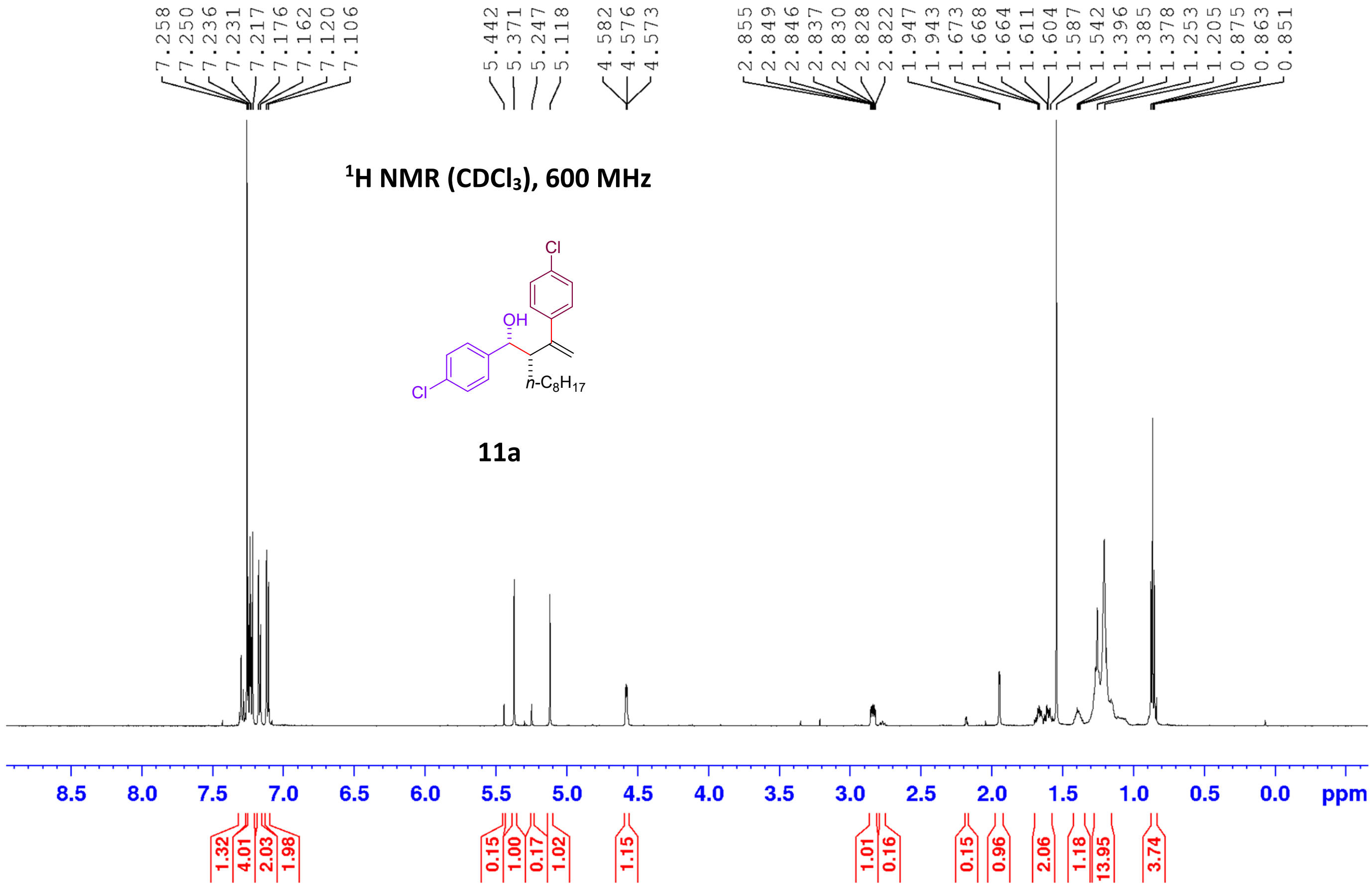
7.258 7.250 7.236 7.231 7.217 7.176 7.162 7.120 7.106
5.442 5.371 5.247 5.118
4.582 4.576 4.573
2.855 2.849 2.846 2.837 2.830 2.828 2.822 1.947 1.943 1.673 1.668 1.664 1.611 1.604 1.587 1.542 1.396 1.385 1.378 1.253 1.205 0.875 0.863 0.851
^{1}H NMR (CDCl$_3$), 600 MHz
OH
Cl
Cl
n-C_8H_{17}
11a
8.5 8.0 7.5 7.0 6.5 6.0 5.5 5.0 4.5 4.0 3.5 3.0 2.5 2.0 1.5 1.0 0.5 0.0 ppm
1.32 4.01 2.03 1.98 0.15 1.00 0.17 1.02 1.15 1.01 0.16 0.15 0.96 2.06 1.18 13.95 3.74

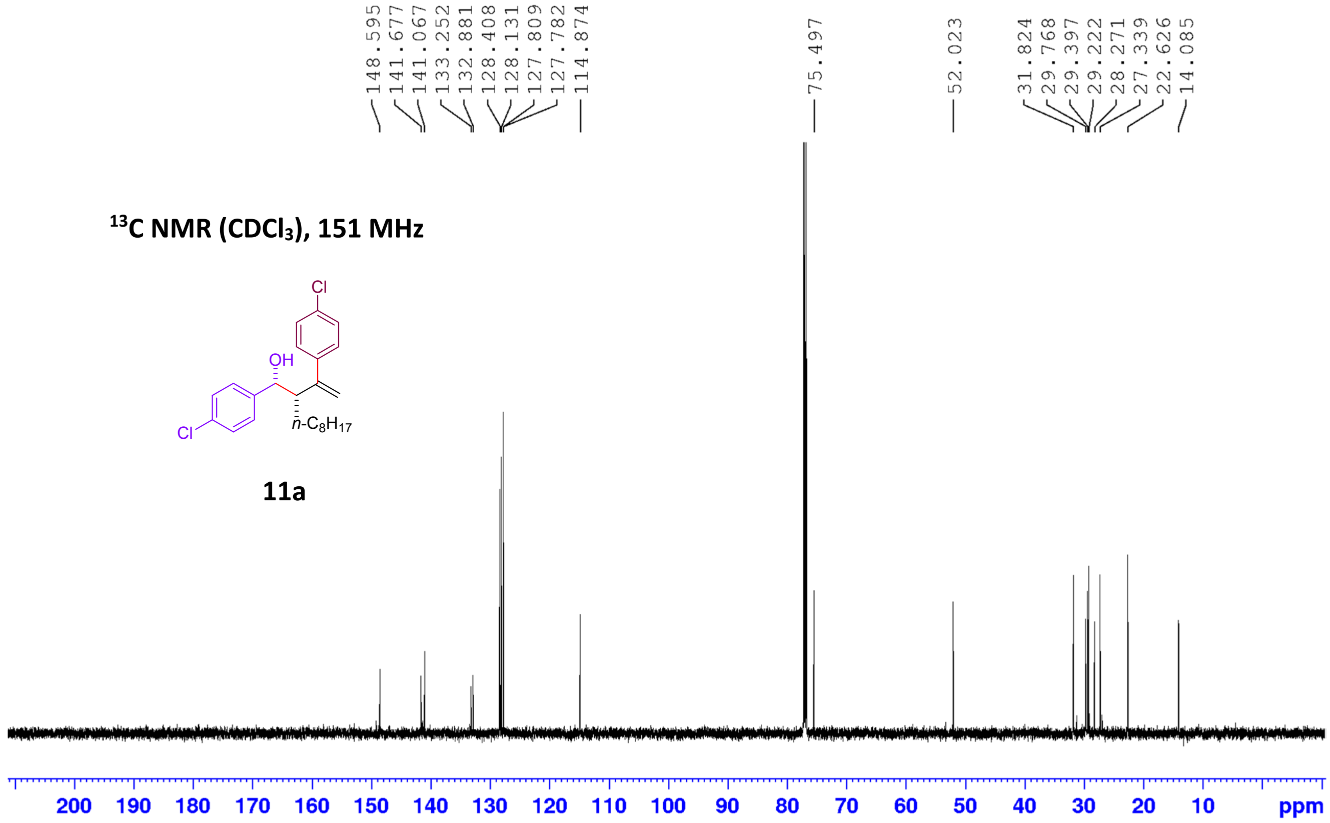
148.595 141.677 141.067 133.252 132.881 128.408 128.131 127.809 127.782 114.874
75.497
52.023
31.824 29.768 29.397 29.222 28.271 27.339 22.626 14.085
^{13}C NMR (CDCl$_3$), 151 MHz
OH
Cl
Cl
n-C_8H_{17}
11a
200 190 180 170 160 150 140 130 120 110 100 90 80 70 60 50 40 30 20 10 ppm

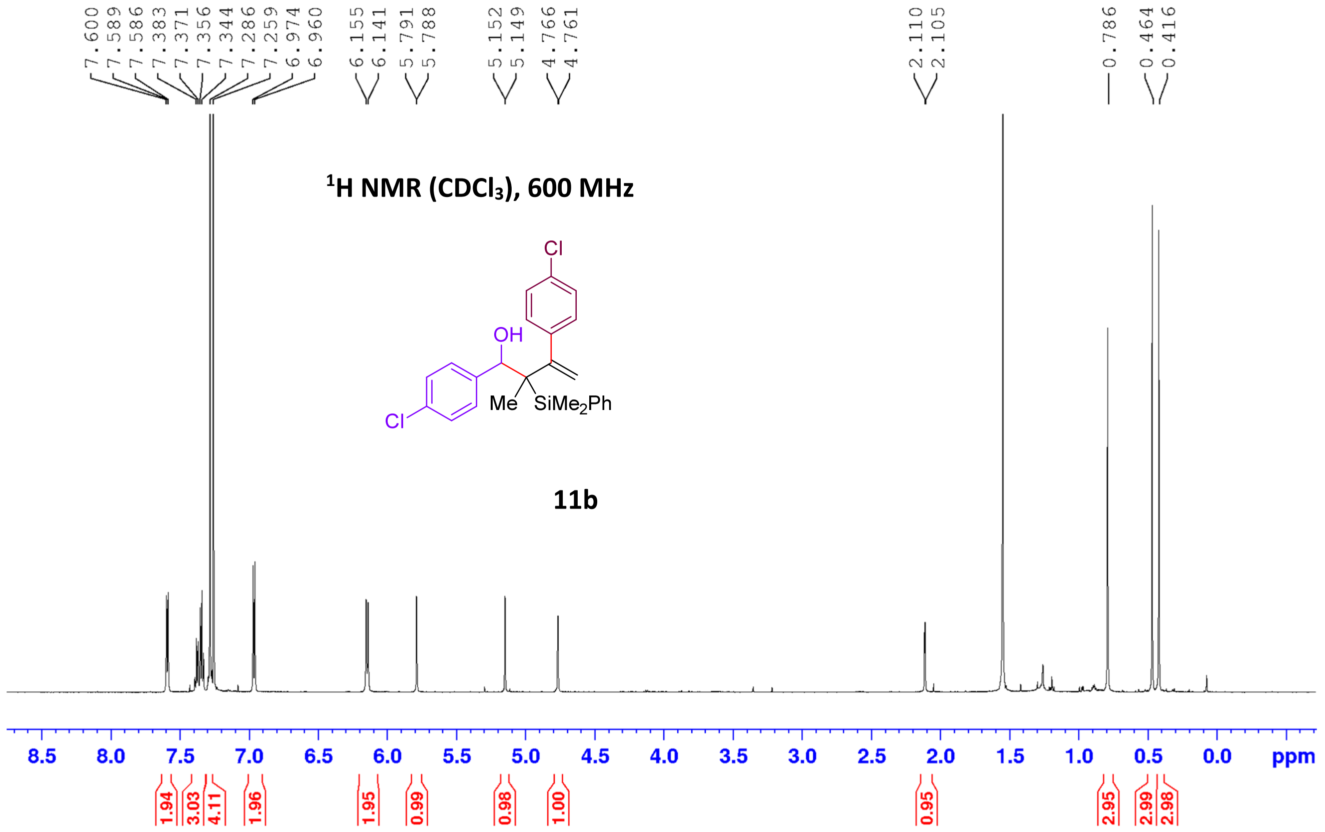

7.600
7.589
7.586
7.383
7.371
7.356
7.344
7.286
7.259
6.974
6.960
6.155
6.141
5.791
5.788
5.152
5.149
4.766
4.761
2.110
2.105
0.786
0.464
0.416
1H NMR (CDCl3), 600 MHz
Cl
OH
Me
SiMe2Ph
Cl
11b
8.5
8.0
7.5
7.0
6.5
6.0
5.5
5.0
4.5
4.0
3.5
3.0
2.5
2.0
1.5
1.0
0.5
0.0
ppm
1.94
3.03
4.11
1.96
1.95
0.99
0.98
1.00
0.95
2.95
2.99
2.98

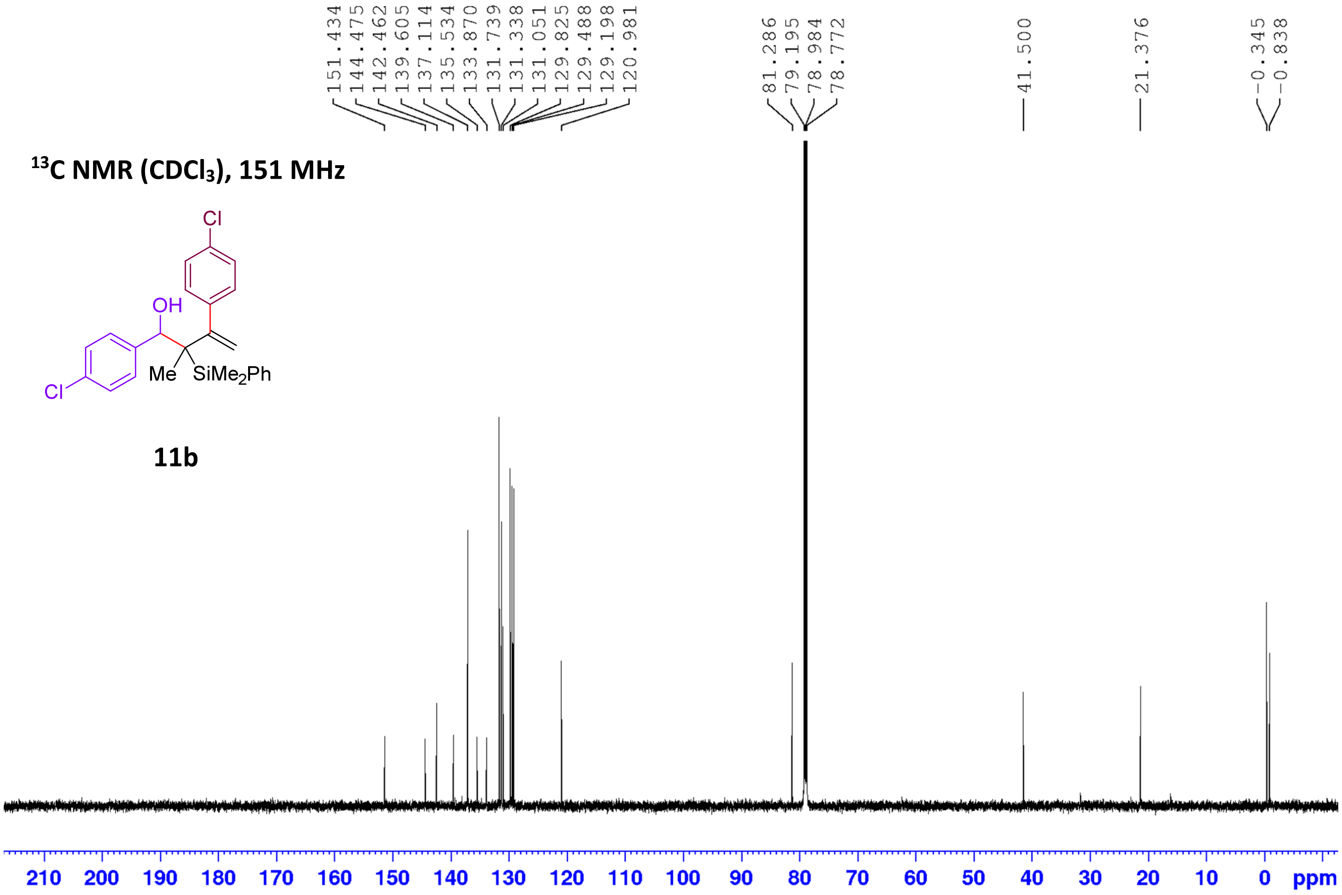

151.434
144.475
142.462
139.605
137.114
135.534
133.870
131.739
131.338
131.051
129.825
129.488
129.198
120.981
81.286
79.195
78.984
78.772
41.500
21.376
-0.345
-0.838
13C NMR (CDCl3), 151 MHz
Cl
OH
Me
SiMe2Ph
Cl
11b
210
200
190
180
170
160
150
140
130
120
110
100
90
80
70
60
50
40
30
20
10
0
ppm

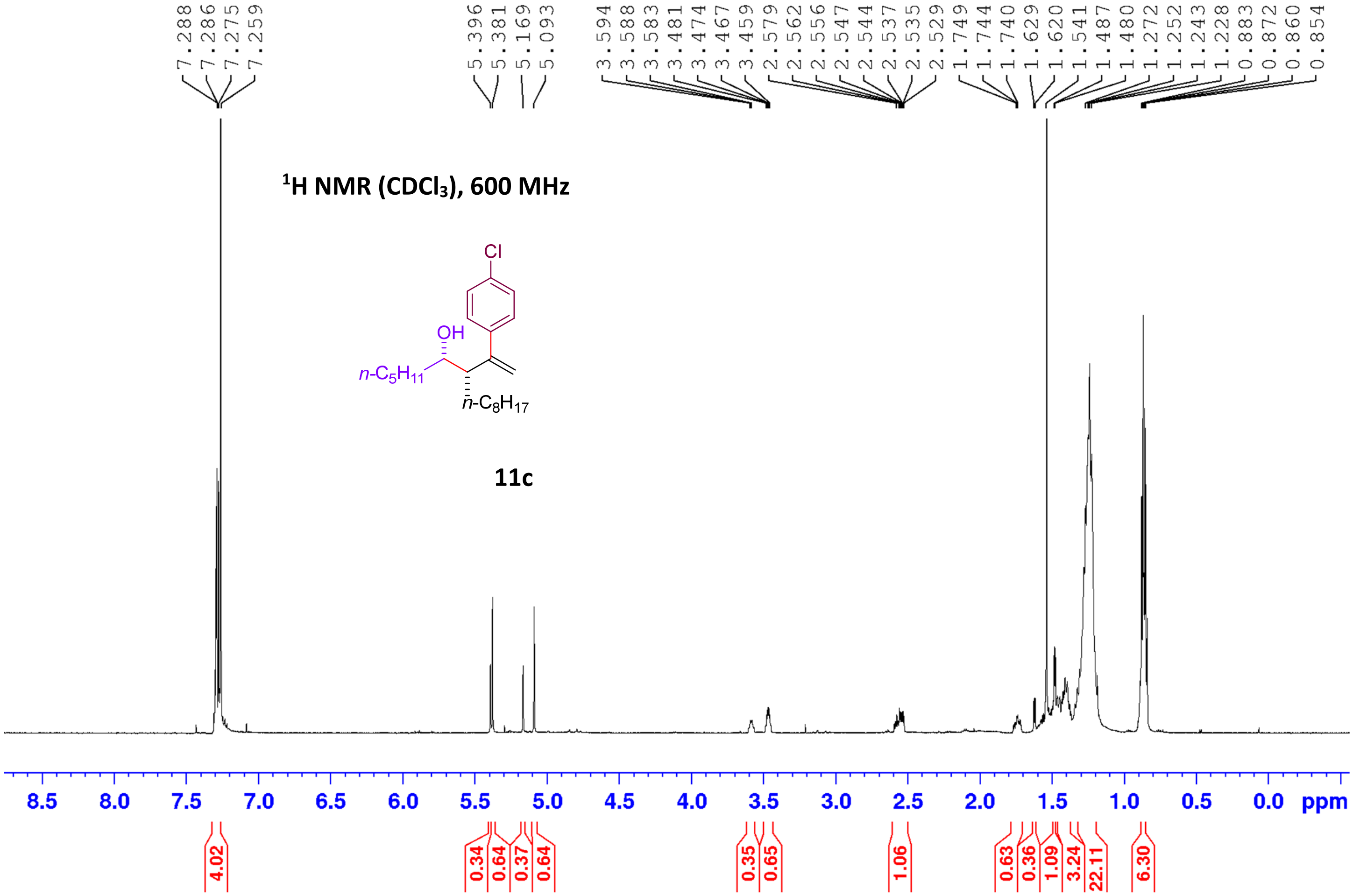

7.288
7.286
7.275
7.259
5.396
5.381
5.169
5.093
3.594
3.588
3.583
3.481
3.474
3.467
3.459
2.579
2.562
2.556
2.547
2.544
2.537
2.535
2.529
1.749
1.744
1.740
1.629
1.620
1.541
1.487
1.480
1.272
1.252
1.243
1.228
0.883
0.872
0.860
0.854
^{1}H NMR (CDCl$_3$), 600 MHz
Cl
OH
n-C5H11
n-C8H17
11c
4.02
0.34
0.64
0.37
0.64
0.35
0.65
1.06
0.63
0.36
1.09
3.24
22.11
6.30
ppm

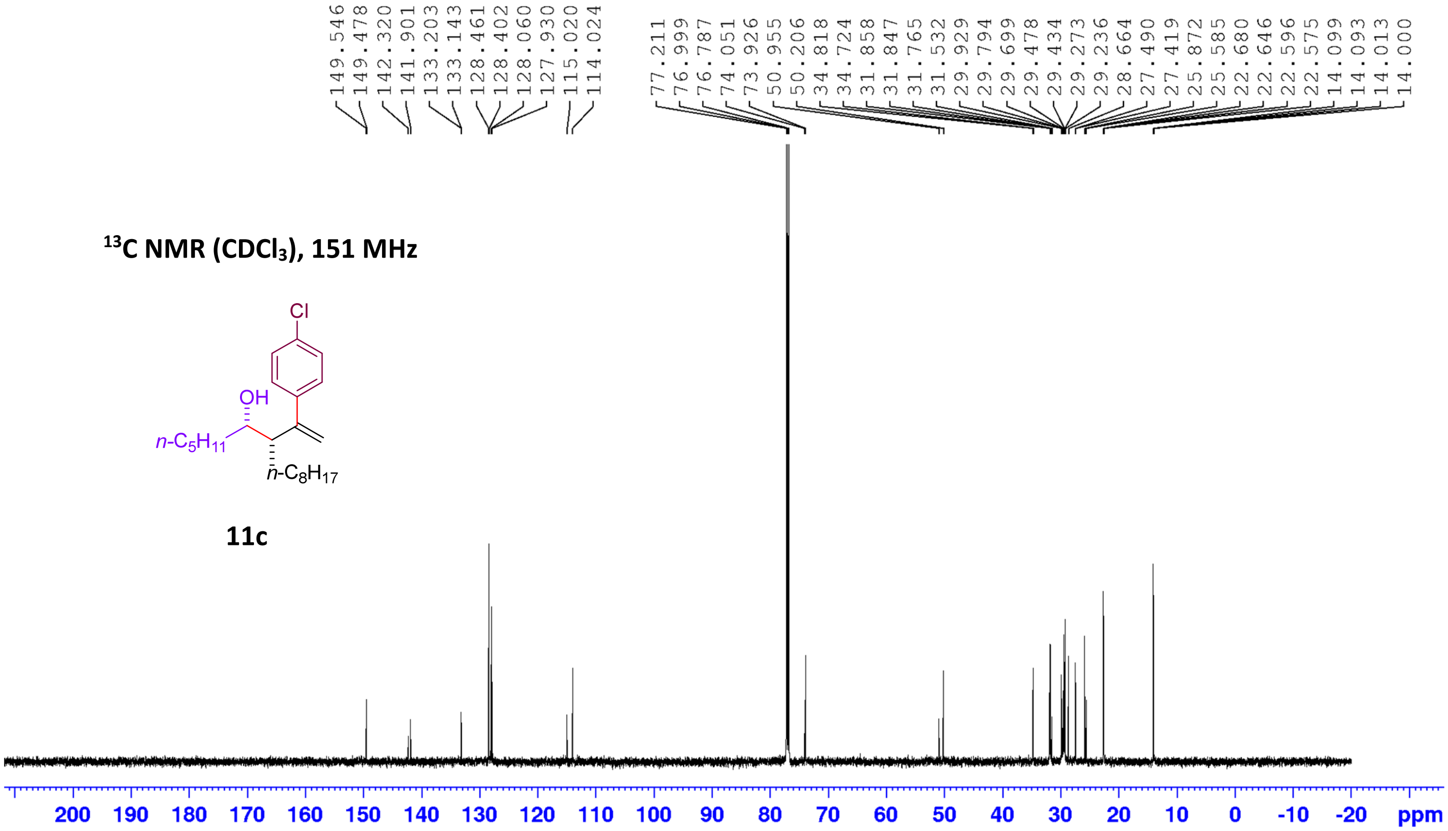

149.546
149.478
142.320
141.901
133.203
133.143
128.461
128.402
128.060
127.930
115.020
114.024
77.211
76.999
76.787
74.051
73.926
50.955
50.206
34.818
34.724
31.858
31.847
31.765
31.532
29.929
29.794
29.699
29.478
29.434
29.273
29.236
28.664
27.490
27.419
25.872
25.585
22.680
22.646
22.596
22.575
14.099
14.093
14.013
14.000
^{13}C NMR (CDCl$_3$), 151 MHz
Cl
OH
n-C5H11
n-C8H17
11c
ppm

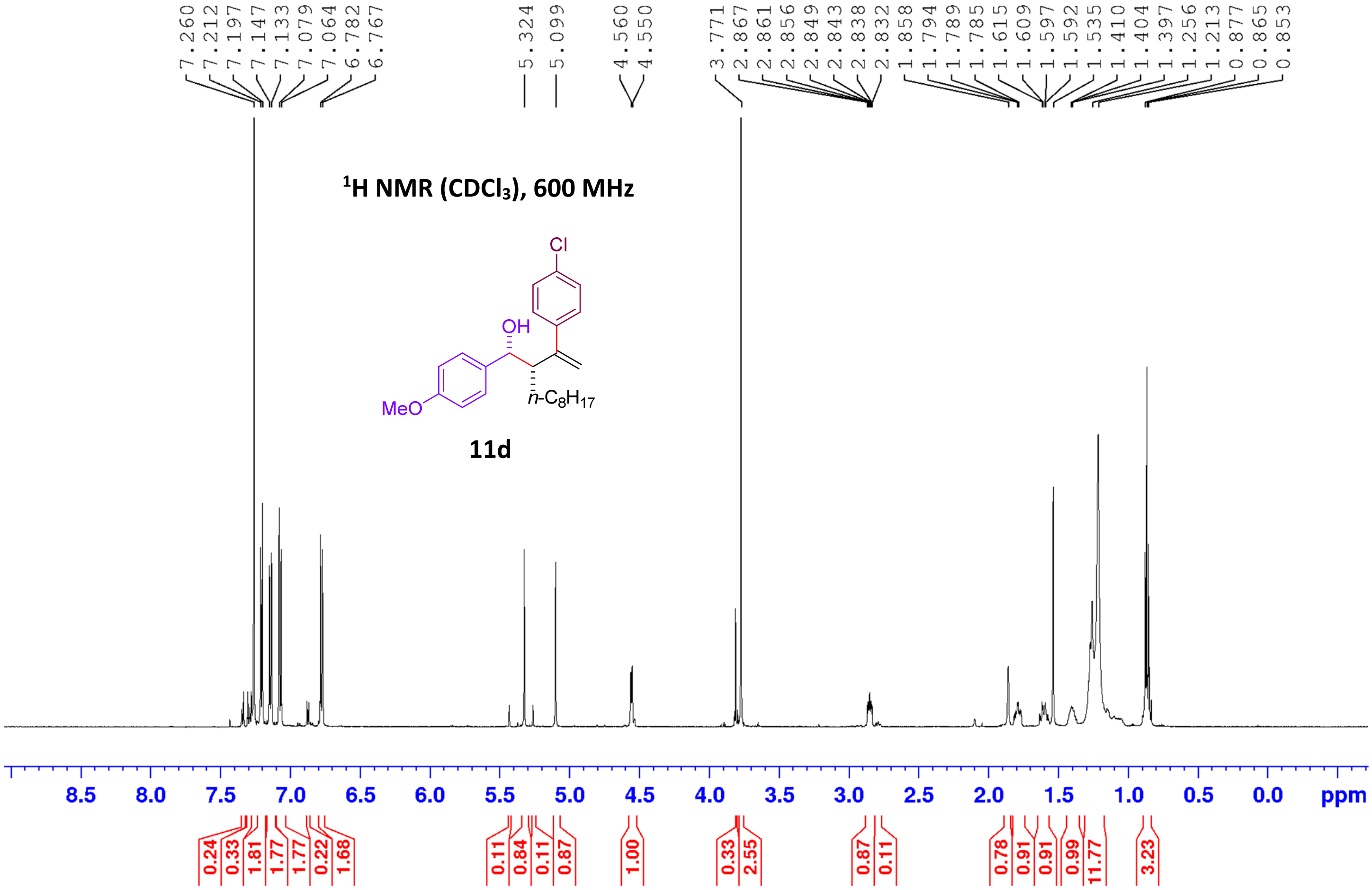

7.260
7.212
7.197
7.147
7.133
7.079
7.064
6.782
6.767
5.324
5.099
4.560
4.550
3.771
2.867
2.861
2.856
2.849
2.843
2.838
2.832
1.858
1.794
1.789
1.785
1.615
1.609
1.597
1.592
1.535
1.410
1.404
1.397
1.256
1.213
0.877
0.865
0.853
1H NMR (CDCl3), 600 MHz
Cl
OH
MeO
n-C8H17
11d
8.5
8.0
7.5
7.0
6.5
6.0
5.5
5.0
4.5
4.0
3.5
3.0
2.5
2.0
1.5
1.0
0.5
0.0
ppm
0.24
0.33
1.81
1.77
1.77
0.22
1.68
0.11
0.84
0.11
0.87
1.00
0.33
2.55
0.87
0.11
0.78
0.91
0.91
0.99
11.77
3.23

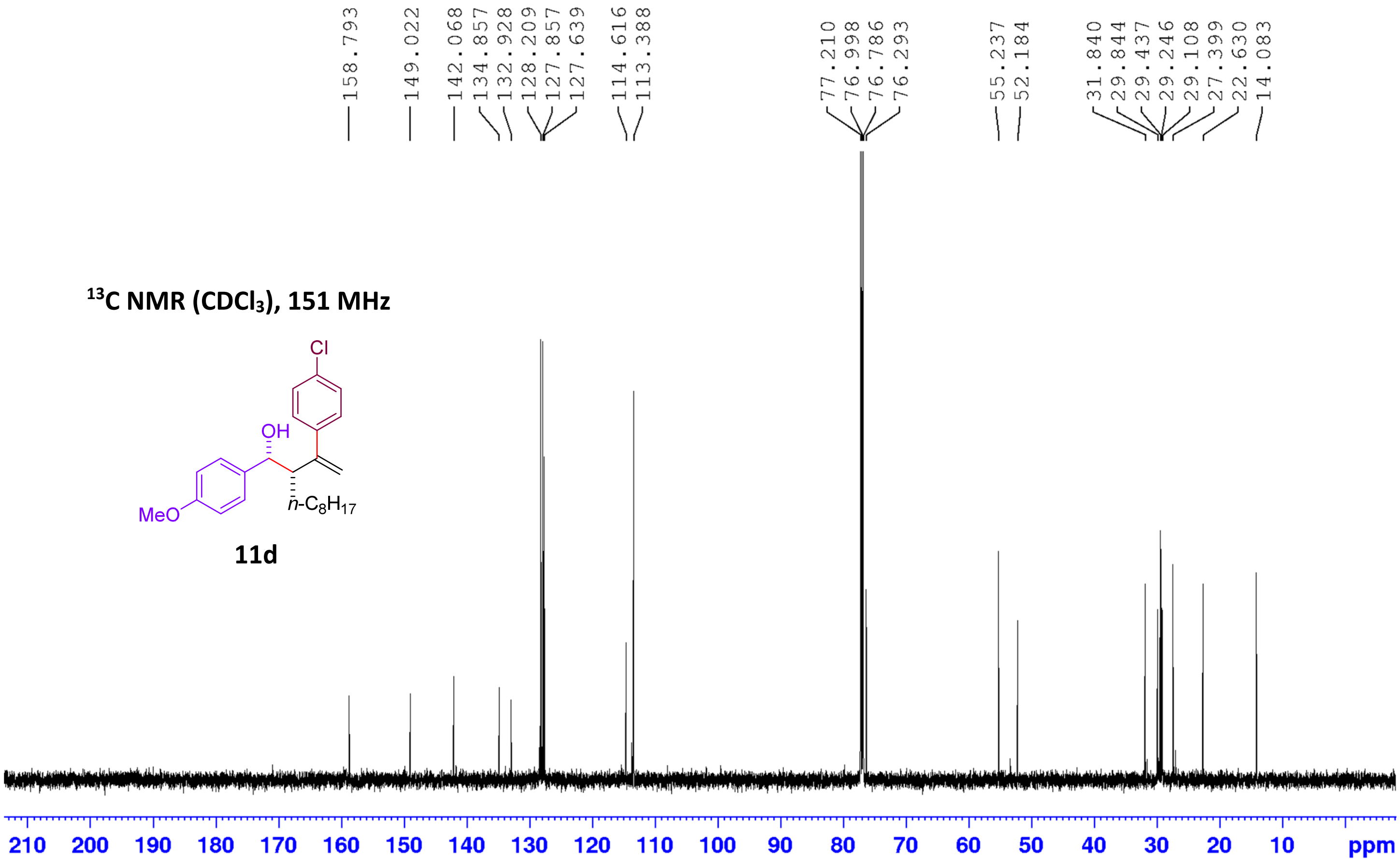

158.793
149.022
142.068
134.857
132.928
128.209
127.857
127.639
114.616
113.388
77.210
76.998
76.786
76.293
55.237
52.184
31.840
29.844
29.437
29.246
29.108
27.399
22.630
14.083
13C NMR (CDCl3), 151 MHz
Cl
OH
MeO
n-C8H17
11d
210
200
190
180
170
160
150
140
130
120
110
100
90
80
70
60
50
40
30
20
10
ppm

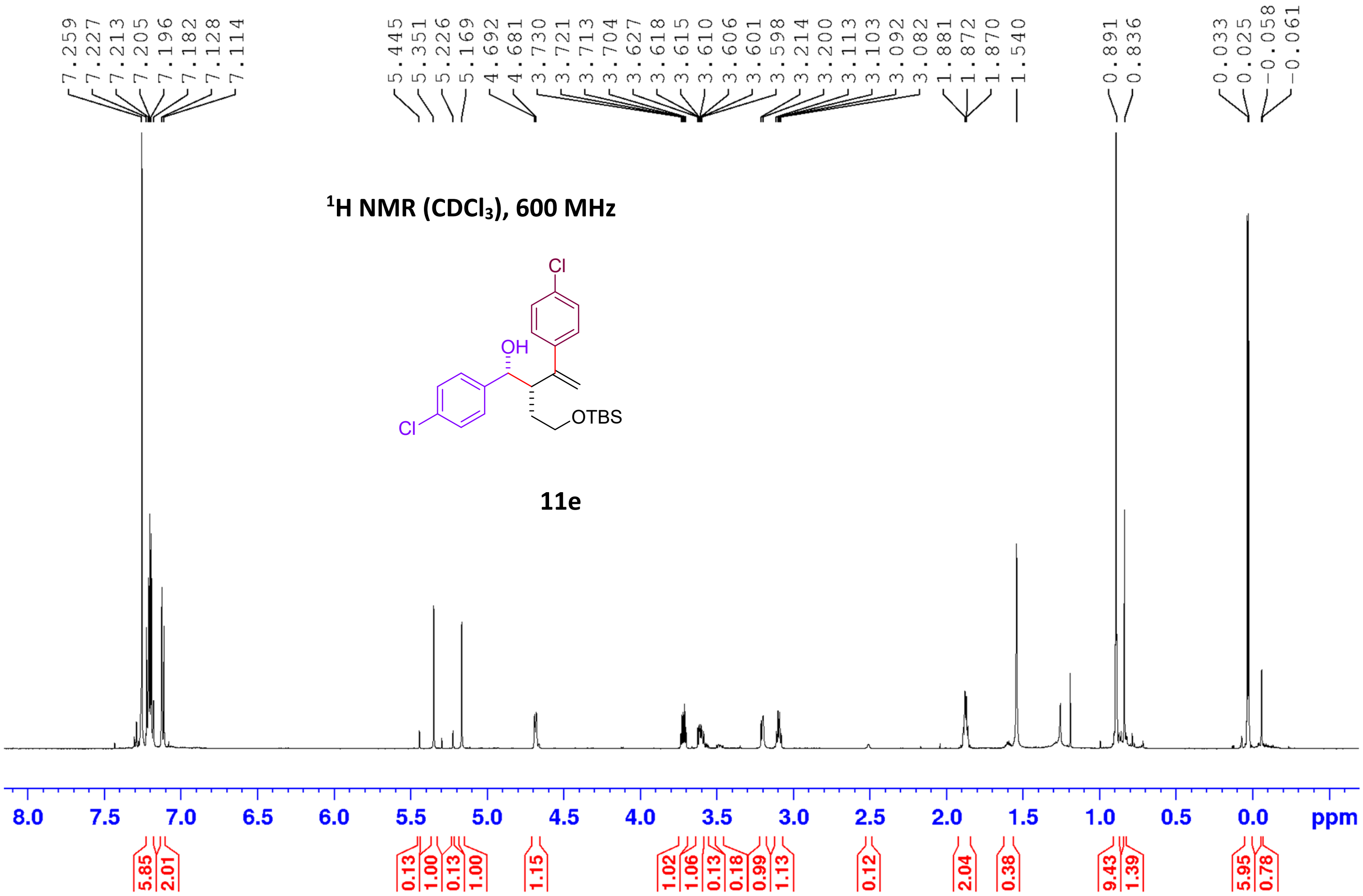
7.259 7.227 7.213 7.205 7.196 7.182 7.128 7.114
5.445 5.351 5.226 5.169 4.692 4.681 3.730 3.721 3.713 3.704 3.627 3.618 3.615 3.610 3.606 3.601 3.598 3.214 3.200 3.113 3.103 3.092 3.082 1.881 1.872 1.870 1.540
0.891 0.836
0.033 0.025 -0.058 -0.061
1H NMR (CDCl3), 600 MHz
Cl
OH
OTBS
Cl
11e
8.0 7.5 7.0 6.5 6.0 5.5 5.0 4.5 4.0 3.5 3.0 2.5 2.0 1.5 1.0 0.5 0.0 ppm
5.85 2.01 0.13 1.00 0.13 1.00 1.15 1.02 1.06 0.13 0.18 0.99 1.13 0.12 2.04 0.38 9.43 1.39 5.95 0.78

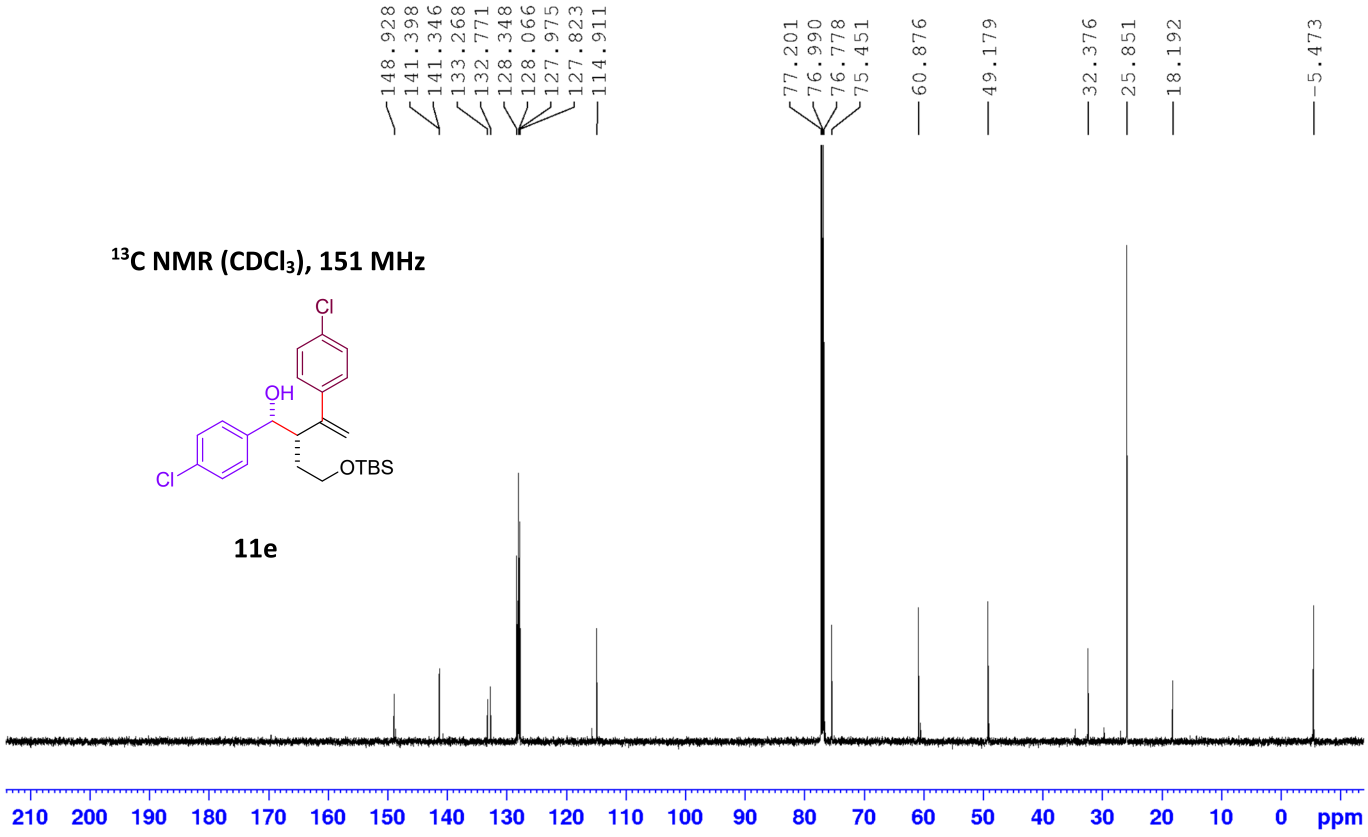
148.928 141.398 141.346 133.268 132.771 128.348 128.066 127.975 127.823 114.911
77.201 76.990 76.778 75.451 60.876 49.179 32.376 25.851 18.192 -5.473
13C NMR (CDCl3), 151 MHz
Cl
OH
OTBS
Cl
11e
210 200 190 180 170 160 150 140 130 120 110 100 90 80 70 60 50 40 30 20 10 0 ppm

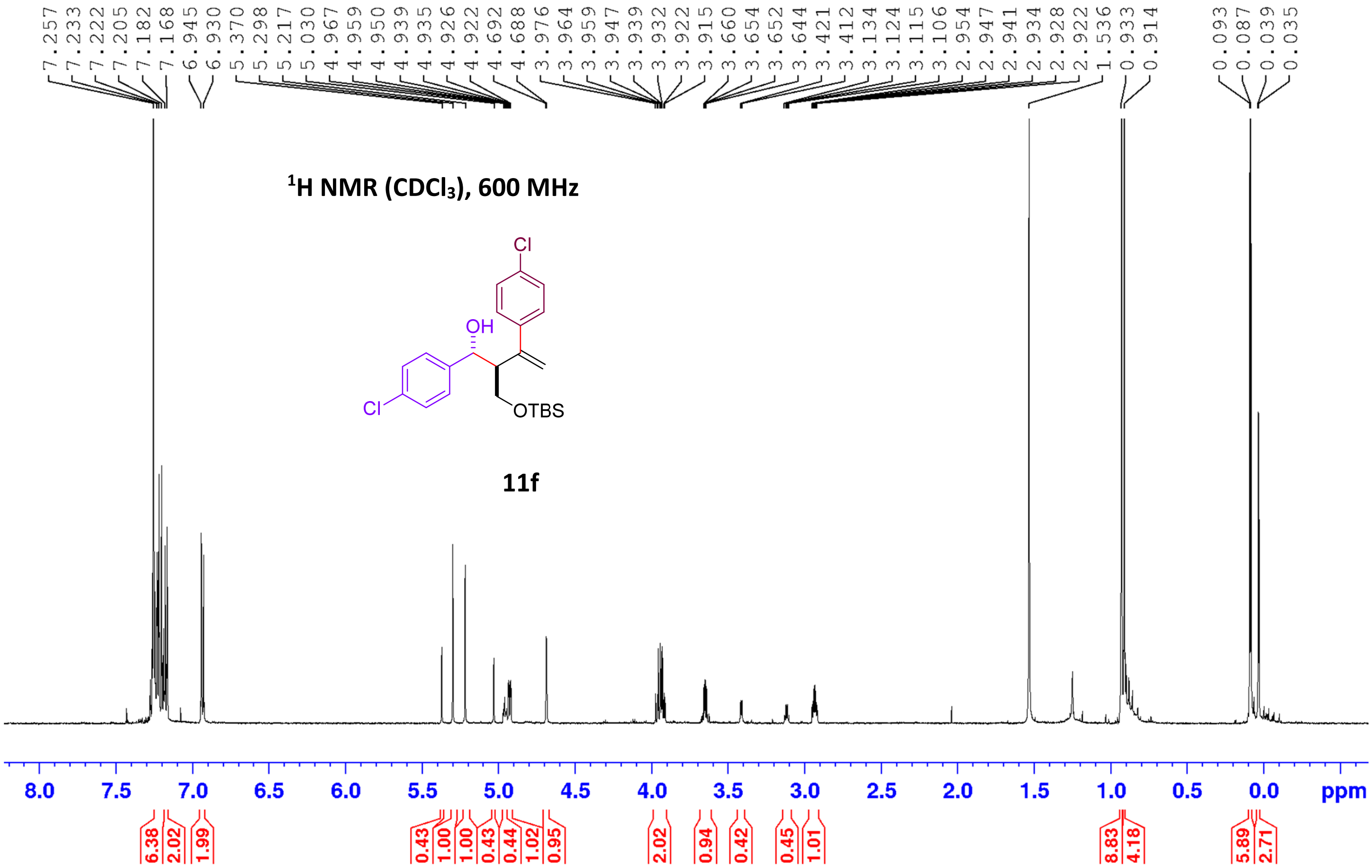
7.257
7.233
7.222
7.205
7.182
7.168
6.945
6.930
5.370
5.298
5.217
5.030
4.967
4.959
4.950
4.939
4.935
4.926
4.922
4.692
4.688
3.976
3.964
3.959
3.947
3.939
3.932
3.922
3.915
3.660
3.654
3.652
3.644
3.421
3.412
3.134
3.124
3.115
3.106
2.954
2.947
2.941
2.934
2.928
2.922
1.536
0.933
0.914
0.093
0.087
0.039
0.035
¹H NMR (CDCl₃), 600 MHz
Cl
OH
Cl
OTBS
11f
8.0
7.5
7.0
6.5
6.0
5.5
5.0
4.5
4.0
3.5
3.0
2.5
2.0
1.5
1.0
0.5
0.0
ppm
6.38
2.02
1.99
0.43
1.00
1.00
0.43
0.44
1.02
0.95
2.02
0.94
0.42
0.45
1.01
8.83
4.18
5.89
2.71

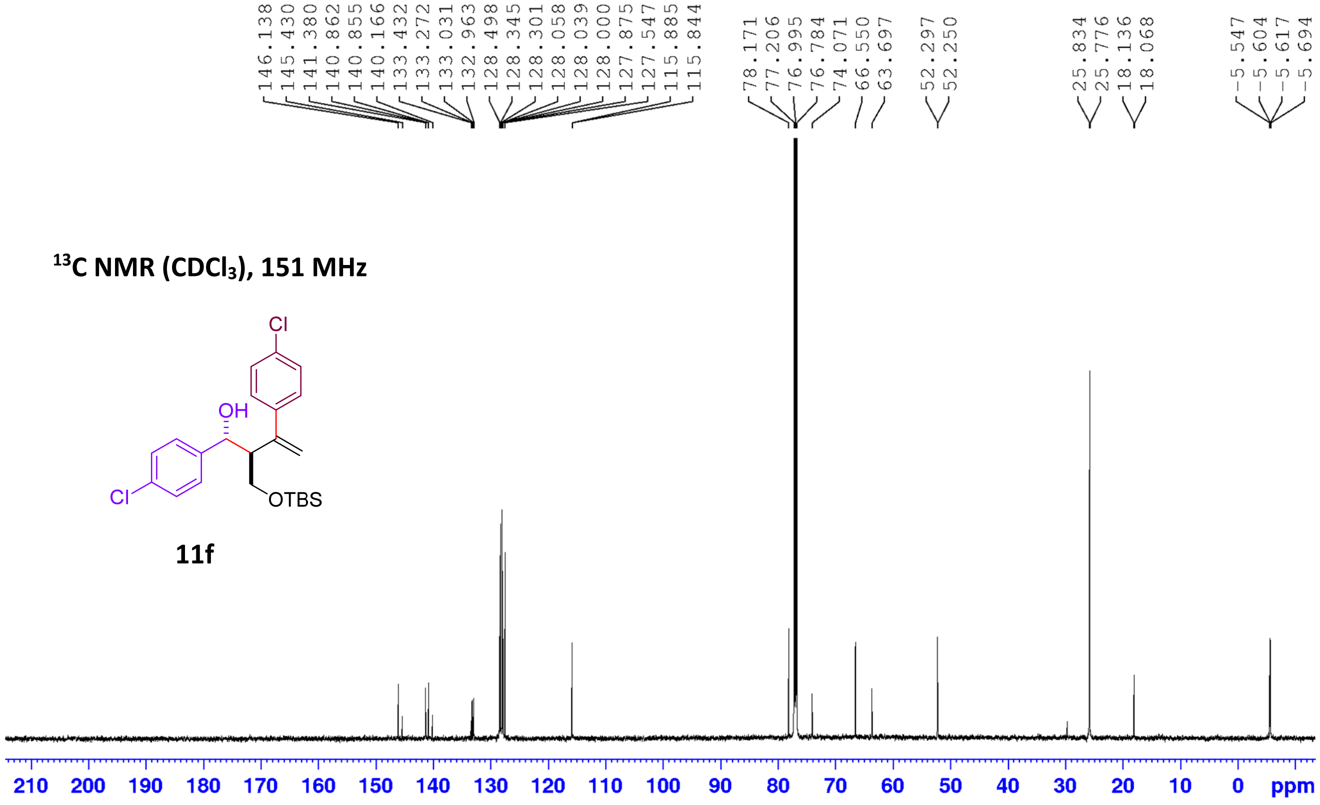
146.138
145.430
141.380
140.862
140.855
140.166
133.432
133.272
133.031
132.963
128.498
128.345
128.301
128.058
128.039
128.000
127.875
127.547
115.885
115.844
78.171
77.206
76.995
76.784
74.071
66.550
63.697
52.297
52.250
25.834
25.776
18.136
18.068
-5.547
-5.604
-5.617
-5.694
¹³C NMR (CDCl₃), 151 MHz
Cl
OH
Cl
OTBS
11f
210
200
190
180
170
160
150
140
130
120
110
100
90
80
70
60
50
40
30
20
10
0
ppm

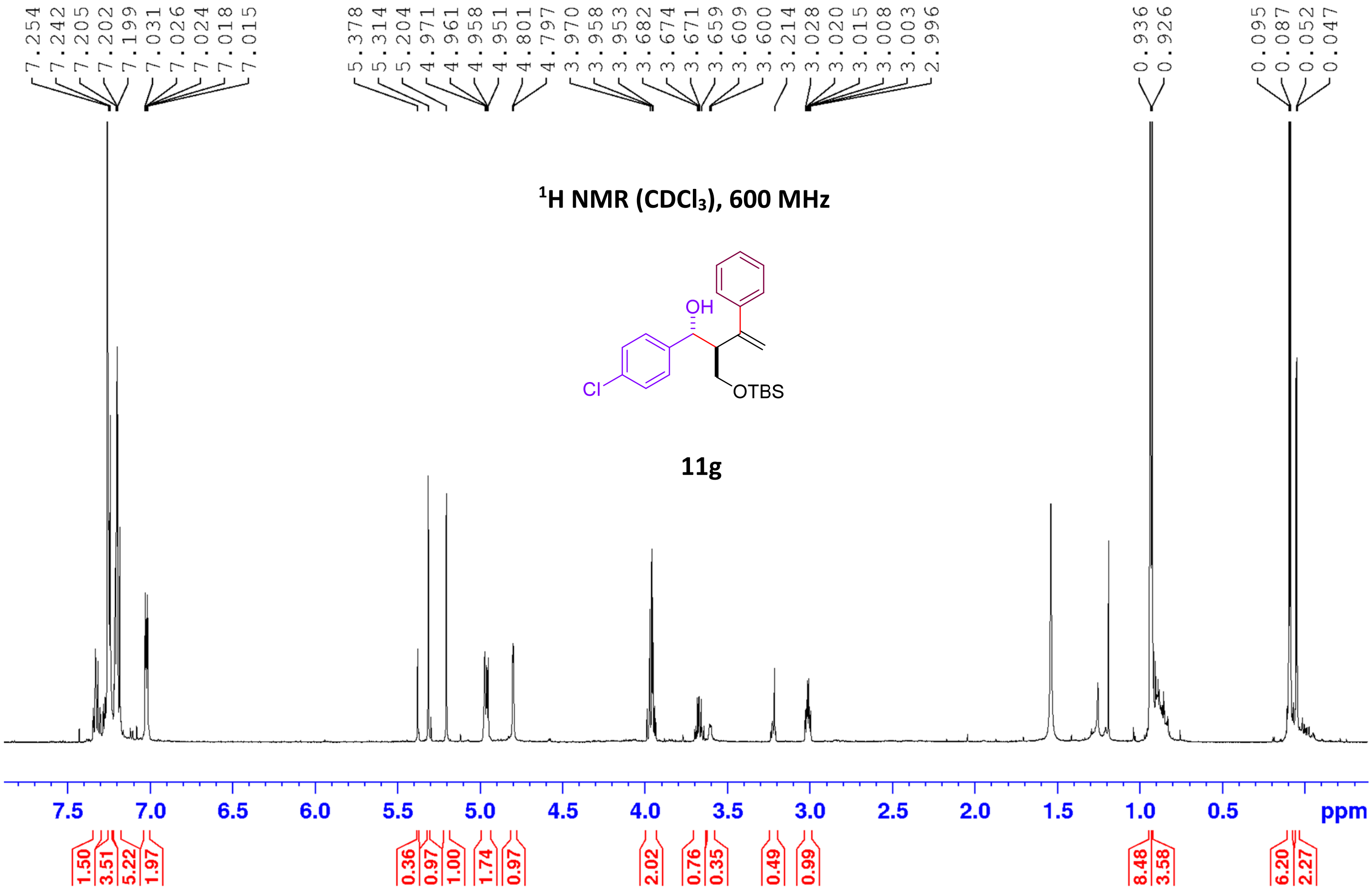

7.254 7.242 7.205 7.202 7.199 7.031 7.026 7.024 7.018 7.015
5.378 5.314 5.204 4.971 4.961 4.958 4.951 4.801 4.797 3.970 3.958 3.953 3.682 3.674 3.671 3.659 3.609 3.600 3.214 3.028 3.020 3.015 3.008 3.003 2.996
0.936 0.926
0.095 0.087 0.052 0.047
1H NMR (CDCl3), 600 MHz
OH
Cl
OTBS
11g
7.5 7.0 6.5 6.0 5.5 5.0 4.5 4.0 3.5 3.0 2.5 2.0 1.5 1.0 0.5 ppm
1.50 3.51 5.22 1.97 0.36 0.97 1.00 1.74 0.97 2.02 0.76 0.35 0.49 0.99 8.48 3.58 6.20 2.27

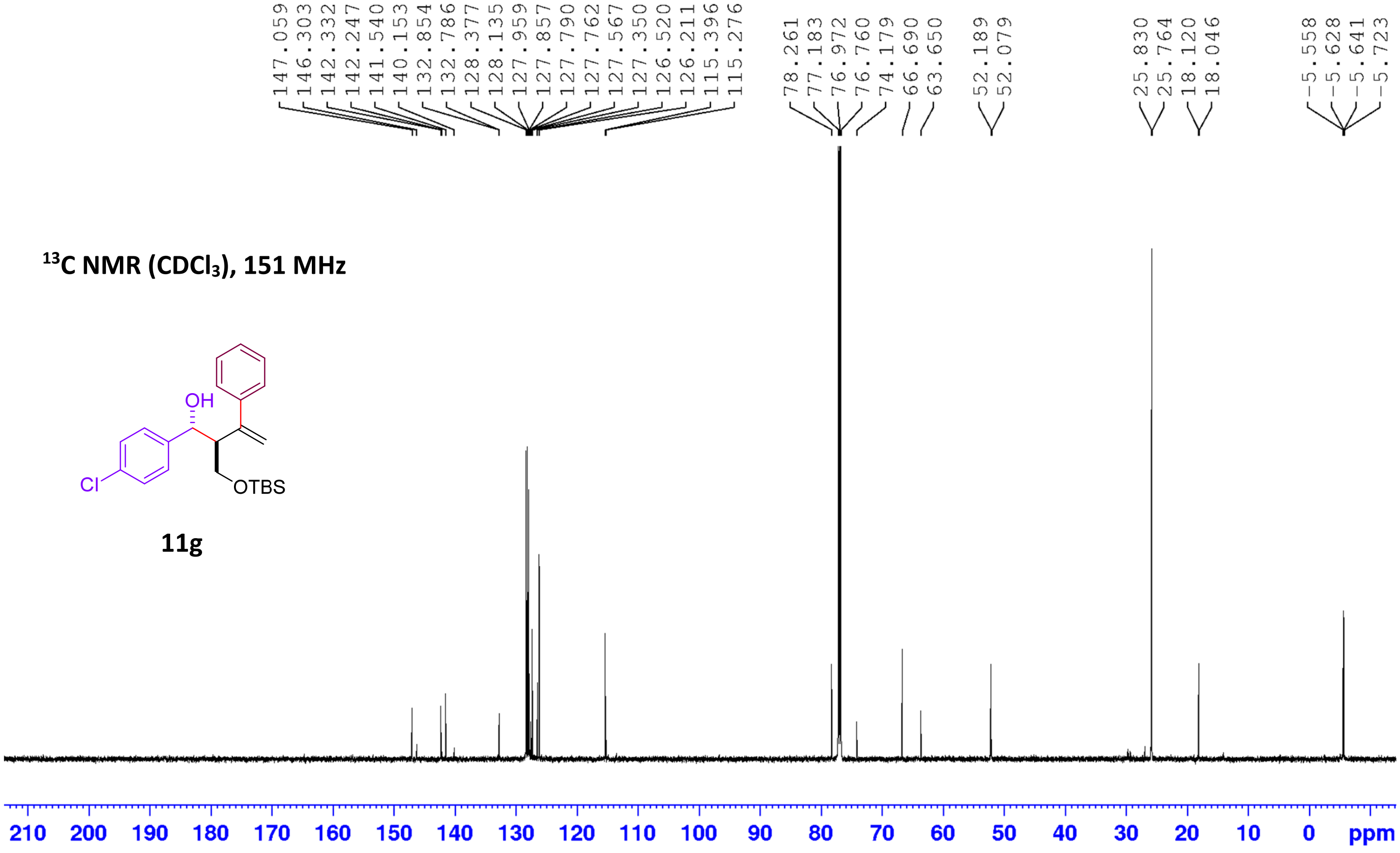

147.059 146.303 142.332 142.247 141.540 140.153 132.854 132.786 128.377 128.135 127.959 127.857 127.790 127.762 127.567 127.350 126.520 126.211 115.396 115.276
78.261 77.183 76.972 76.760 74.179 66.690 63.650
52.189 52.079
25.830 25.764 18.120 18.046
-5.558 -5.628 -5.641 -5.723
13C NMR (CDCl3), 151 MHz
OH
Cl
OTBS
11g
210 200 190 180 170 160 150 140 130 120 110 100 90 80 70 60 50 40 30 20 10 0 ppm

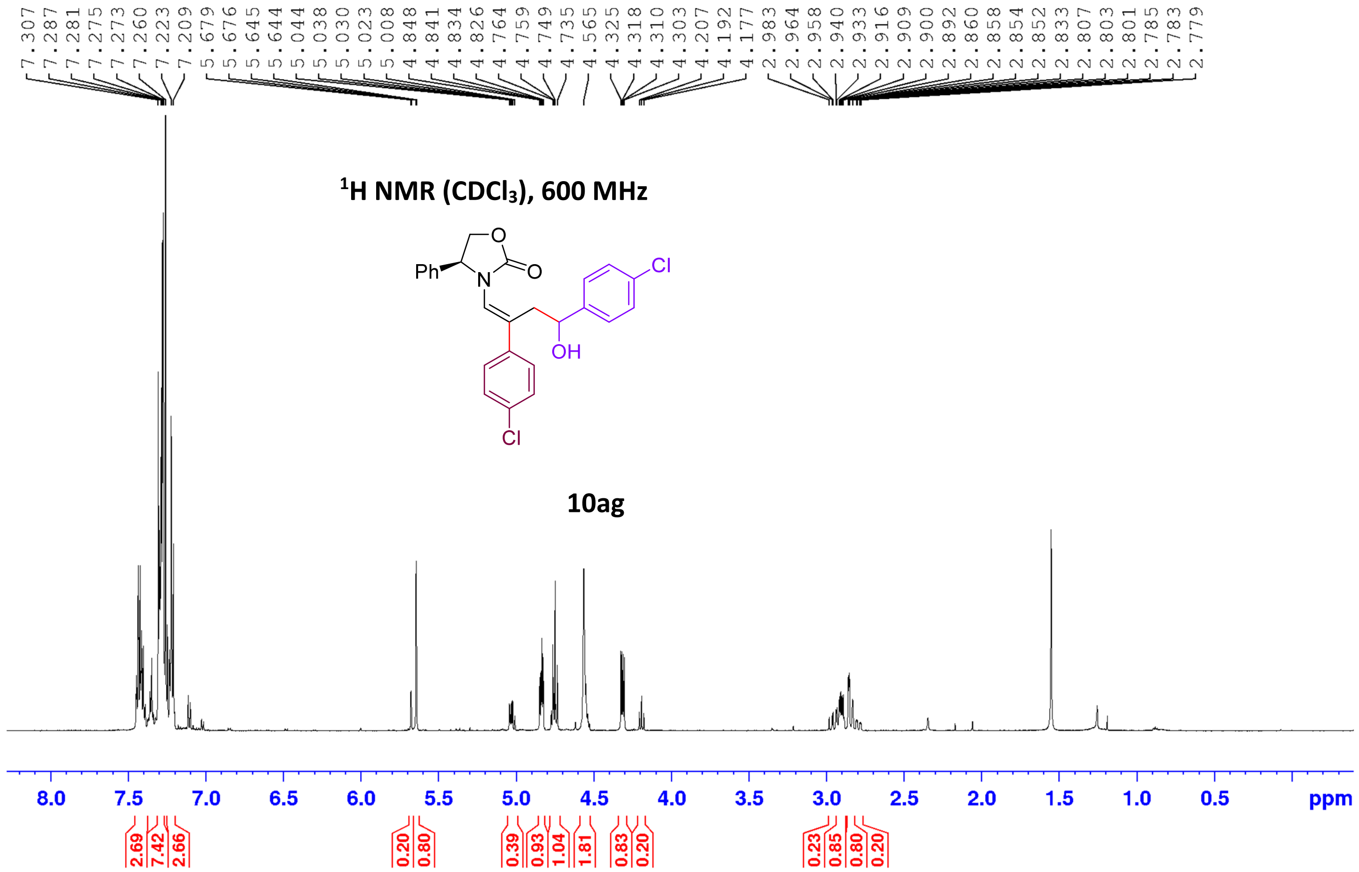
7.307
7.287
7.281
7.275
7.273
7.260
7.223
7.209
5.679
5.676
5.645
5.644
5.044
5.038
5.030
5.023
5.008
4.848
4.841
4.834
4.826
4.764
4.759
4.749
4.735
4.565
4.325
4.318
4.310
4.303
4.207
4.192
4.177
2.983
2.964
2.958
2.940
2.933
2.916
2.909
2.900
2.892
2.860
2.858
2.854
2.852
2.833
2.807
2.803
2.801
2.785
2.783
2.779
^{1}H NMR (CDCl$_3$), 600 MHz
Ph
N
O
O
OH
Cl
Cl
10ag
2.69
7.42
2.66
0.20
0.80
0.39
0.93
1.04
1.81
0.83
0.20
0.23
0.85
0.80
0.20
ppm

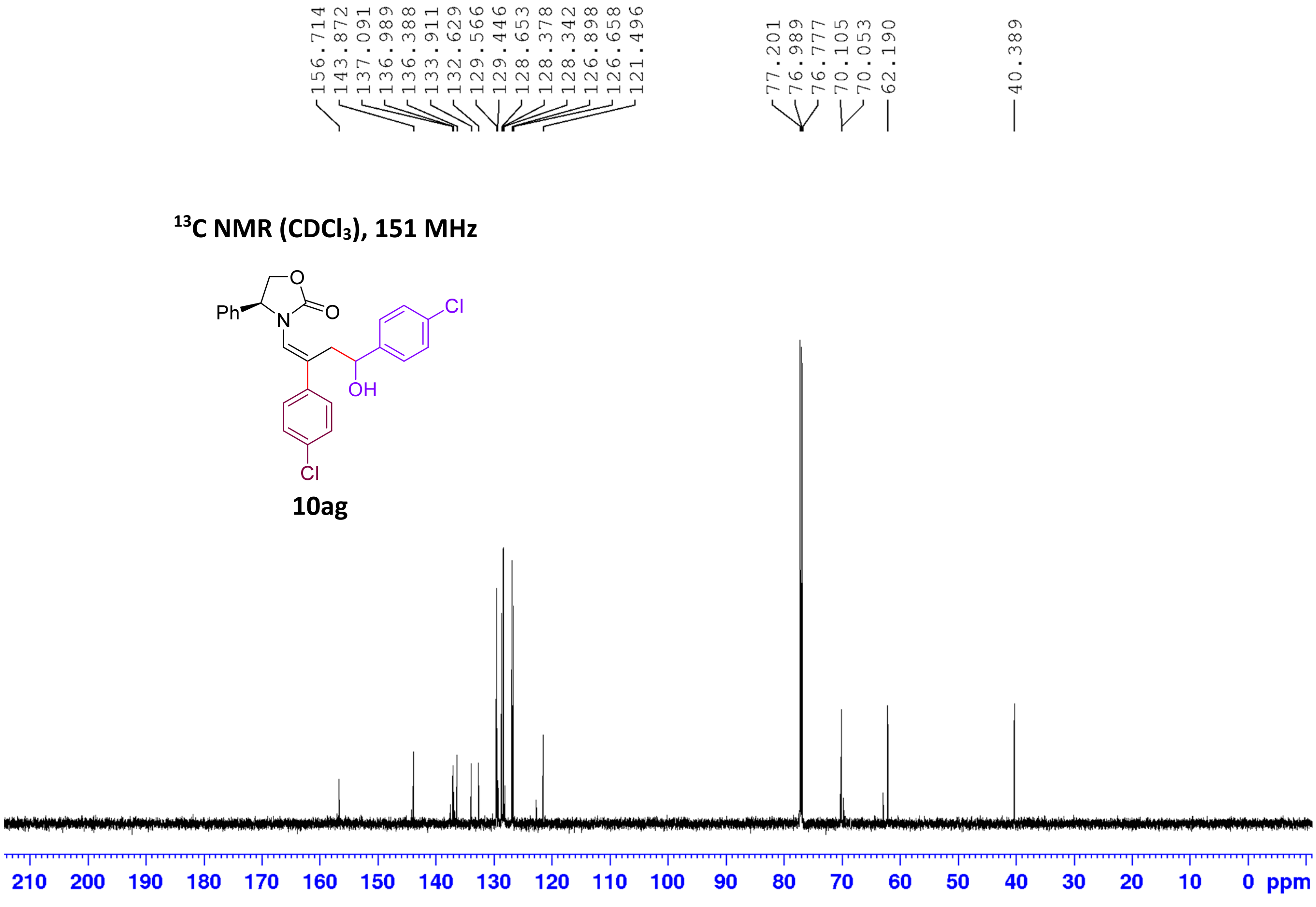
156.714
143.872
137.091
136.989
136.388
133.911
132.629
129.566
129.446
128.653
128.378
128.342
126.898
126.658
121.496
77.201
76.989
76.777
70.105
70.053
62.190
40.389
^{13}C NMR (CDCl$_3$), 151 MHz
Ph
N
O
O
OH
Cl
Cl
10ag
ppm

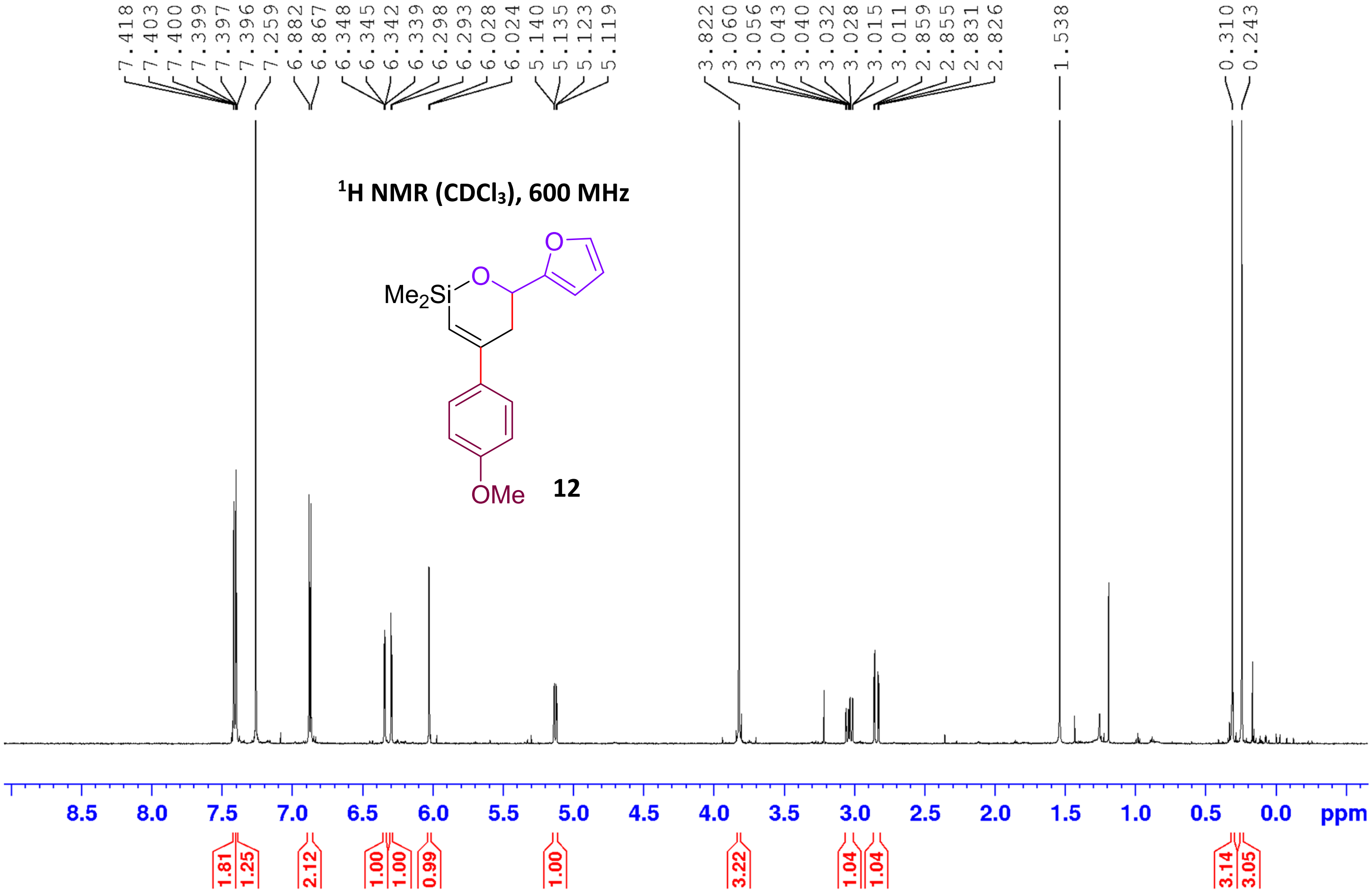
7.418 7.403 7.400 7.399 7.397 7.396 7.259 6.882 6.867 6.348 6.345 6.342 6.339 6.298 6.293 6.028 6.024 5.140 5.135 5.123 5.119
3.822 3.060 3.056 3.043 3.040 3.032 3.028 3.015 3.011 2.859 2.855 2.831 2.826
1.538
0.310 0.243
1H NMR (CDCl3), 600 MHz
Me2Si
O
O
OMe
12
8.5 8.0 7.5 7.0 6.5 6.0 5.5 5.0 4.5 4.0 3.5 3.0 2.5 2.0 1.5 1.0 0.5 0.0 ppm
1.81 1.25 2.12 1.00 1.00 0.99 1.00 3.22 1.04 1.04 3.14 3.05

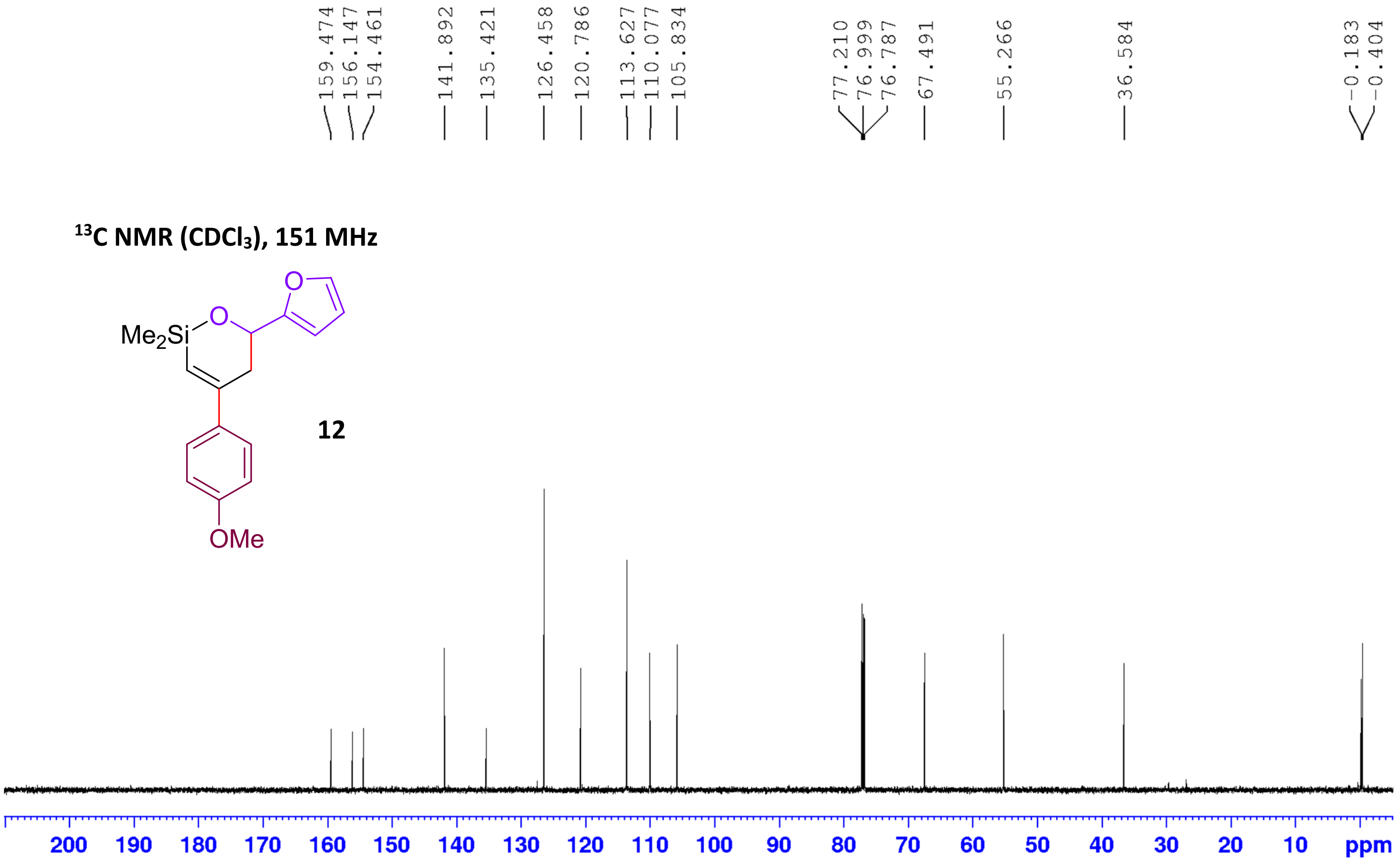
159.474 156.147 154.461 141.892 135.421 126.458 120.786 113.627 110.077 105.834
77.210 76.999 76.787 67.491 55.266 36.584
-0.183 -0.404
13C NMR (CDCl3), 151 MHz
Me2Si
O
O
OMe
12
200 190 180 170 160 150 140 130 120 110 100 90 80 70 60 50 40 30 20 10 ppm

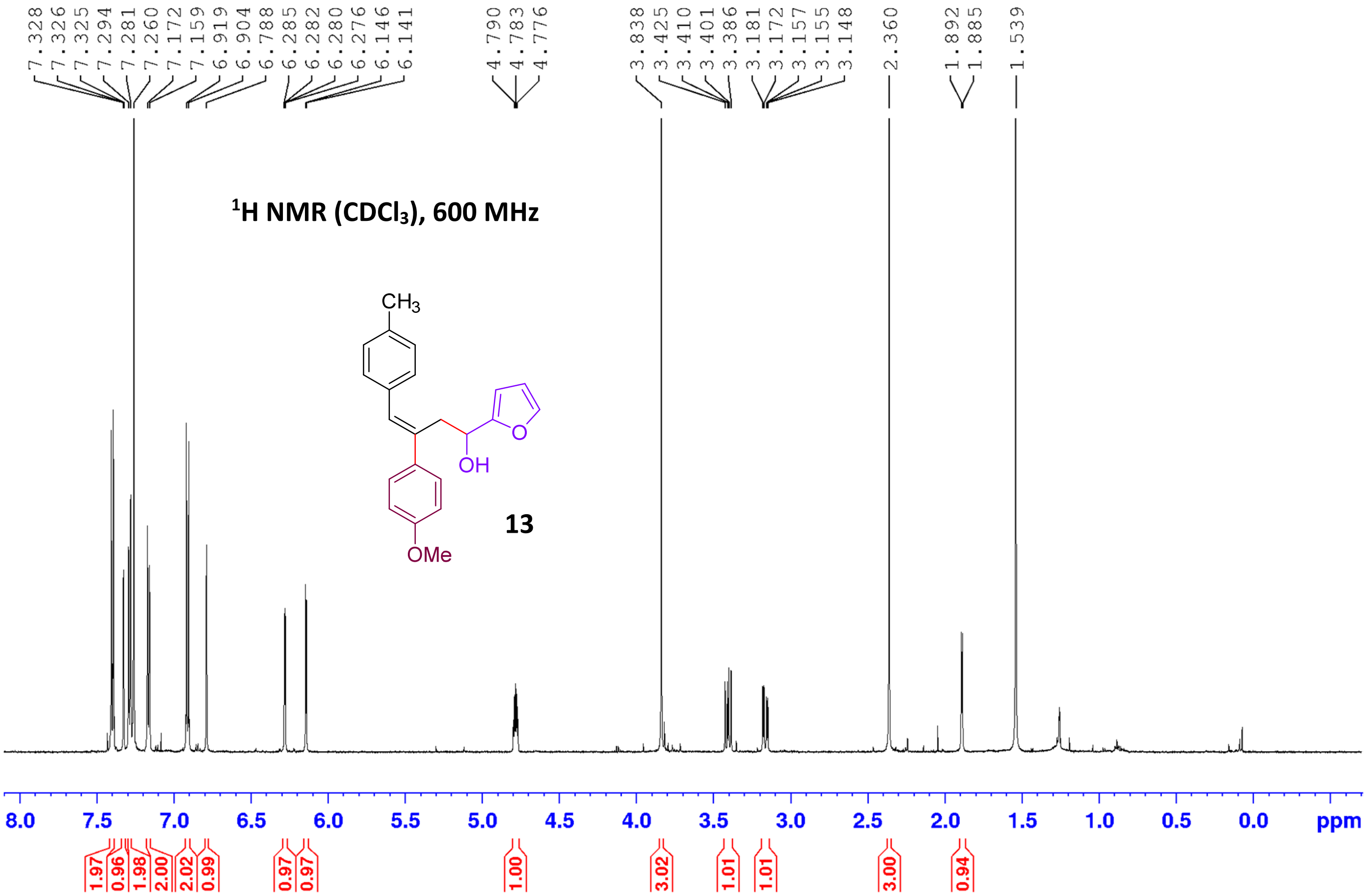

7.328
7.326
7.325
7.294
7.281
7.260
7.172
7.159
6.919
6.904
6.788
6.285
6.282
6.280
6.276
6.146
6.141
4.790
4.783
4.776
3.838
3.425
3.410
3.401
3.386
3.181
3.172
3.157
3.155
3.148
2.360
1.892
1.885
1.539
¹H NMR (CDCl₃), 600 MHz
CH3
OH
O
13
OMe
8.0 7.5 7.0 6.5 6.0 5.5 5.0 4.5 4.0 3.5 3.0 2.5 2.0 1.5 1.0 0.5 0.0 ppm
1.97 0.96 1.98 2.00 2.02 0.99 0.97 0.97 1.00 3.02 1.01 1.01 3.00 0.94

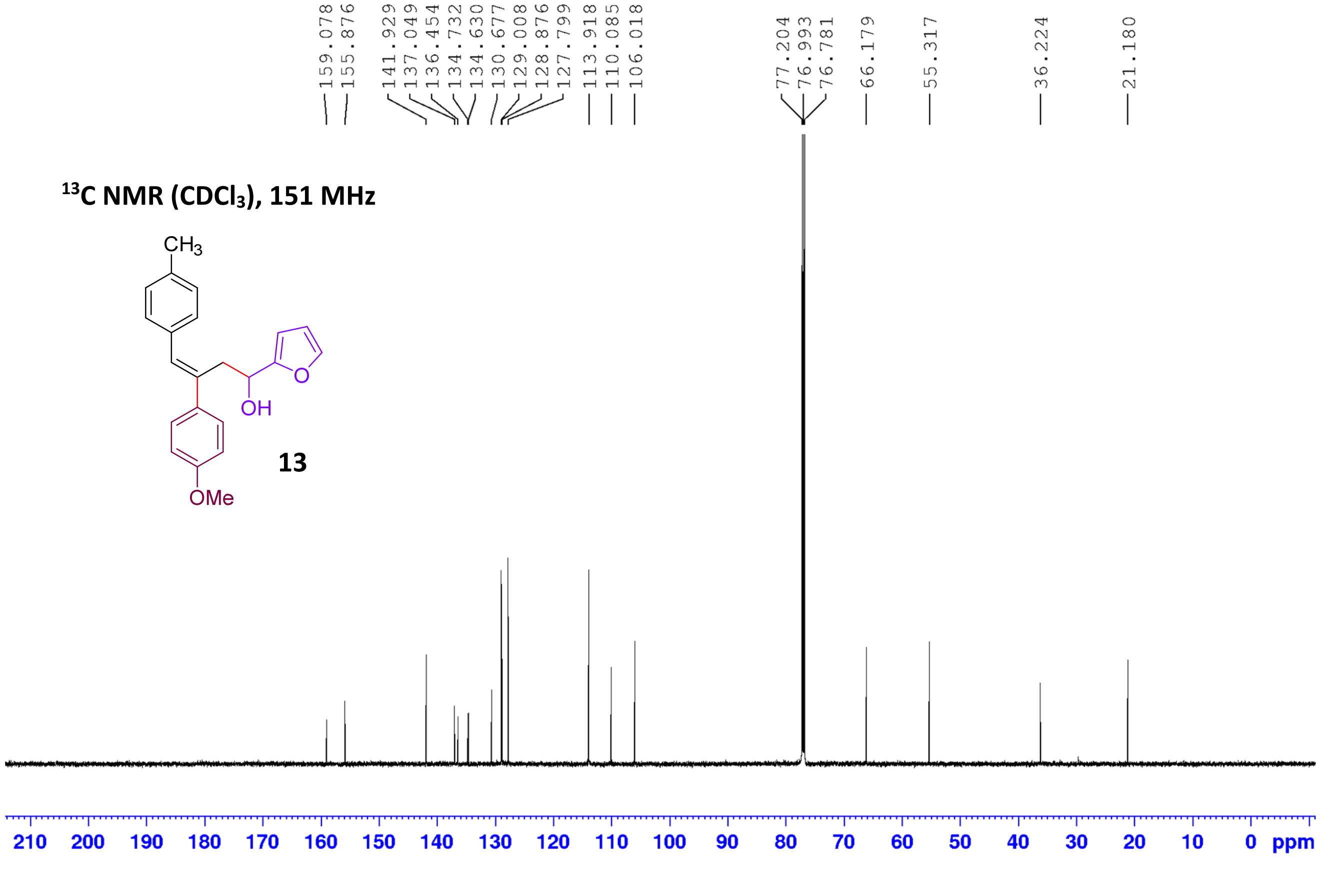

159.078
155.876
141.929
137.049
136.454
134.732
134.630
130.677
129.008
128.876
127.799
113.918
110.085
106.018
77.204
76.993
76.781
66.179
55.317
36.224
21.180
¹³C NMR (CDCl₃), 151 MHz
CH3
OH
O
13
OMe
210 200 190 180 170 160 150 140 130 120 110 100 90 80 70 60 50 40 30 20 10 0 ppm

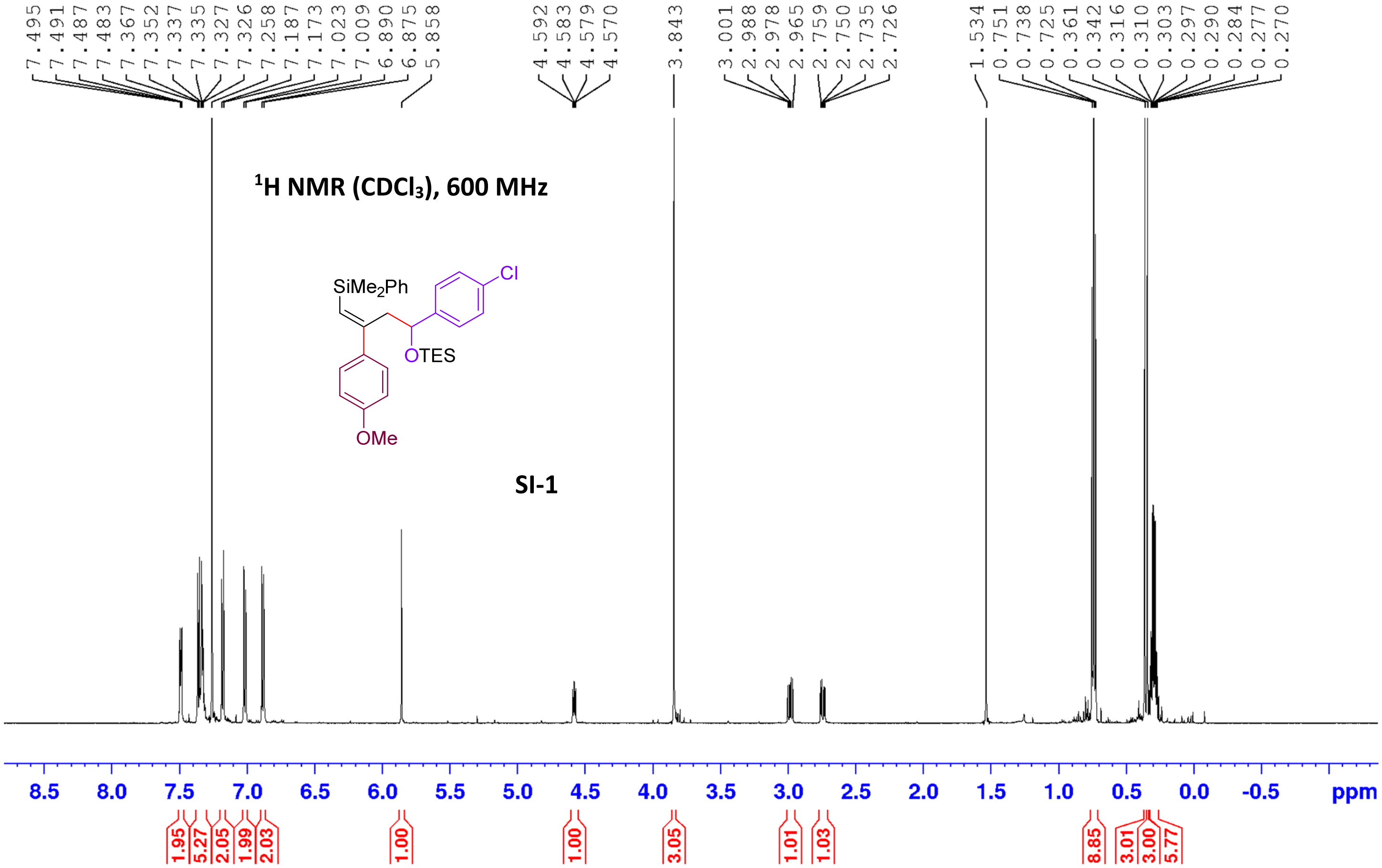
1H NMR (CDCl3), 600 MHz
SiMe2Ph
Cl
OTES
OMe
SI-1

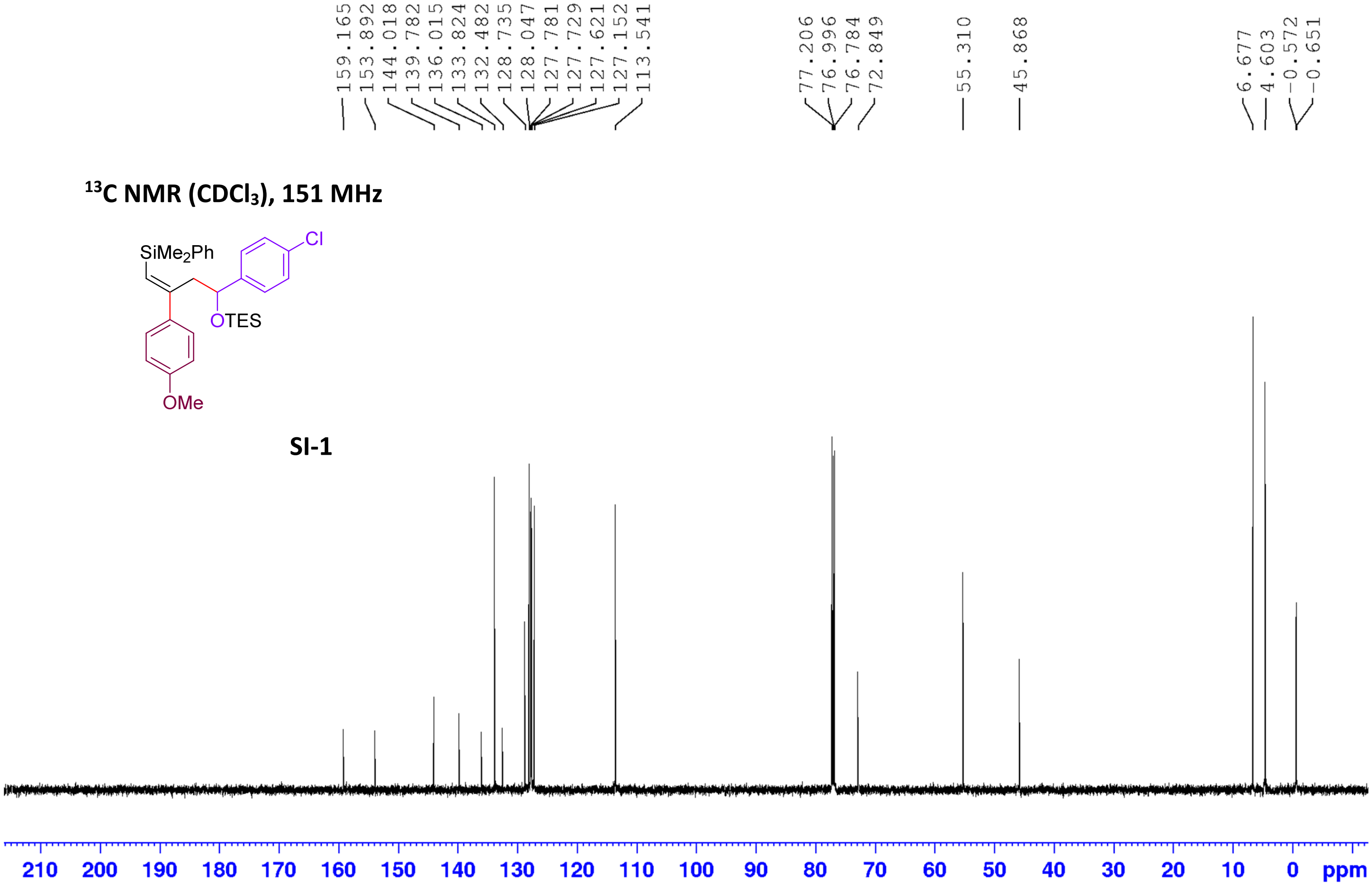
13C NMR (CDCl3), 151 MHz
SiMe2Ph
Cl
OTES
OMe
SI-1

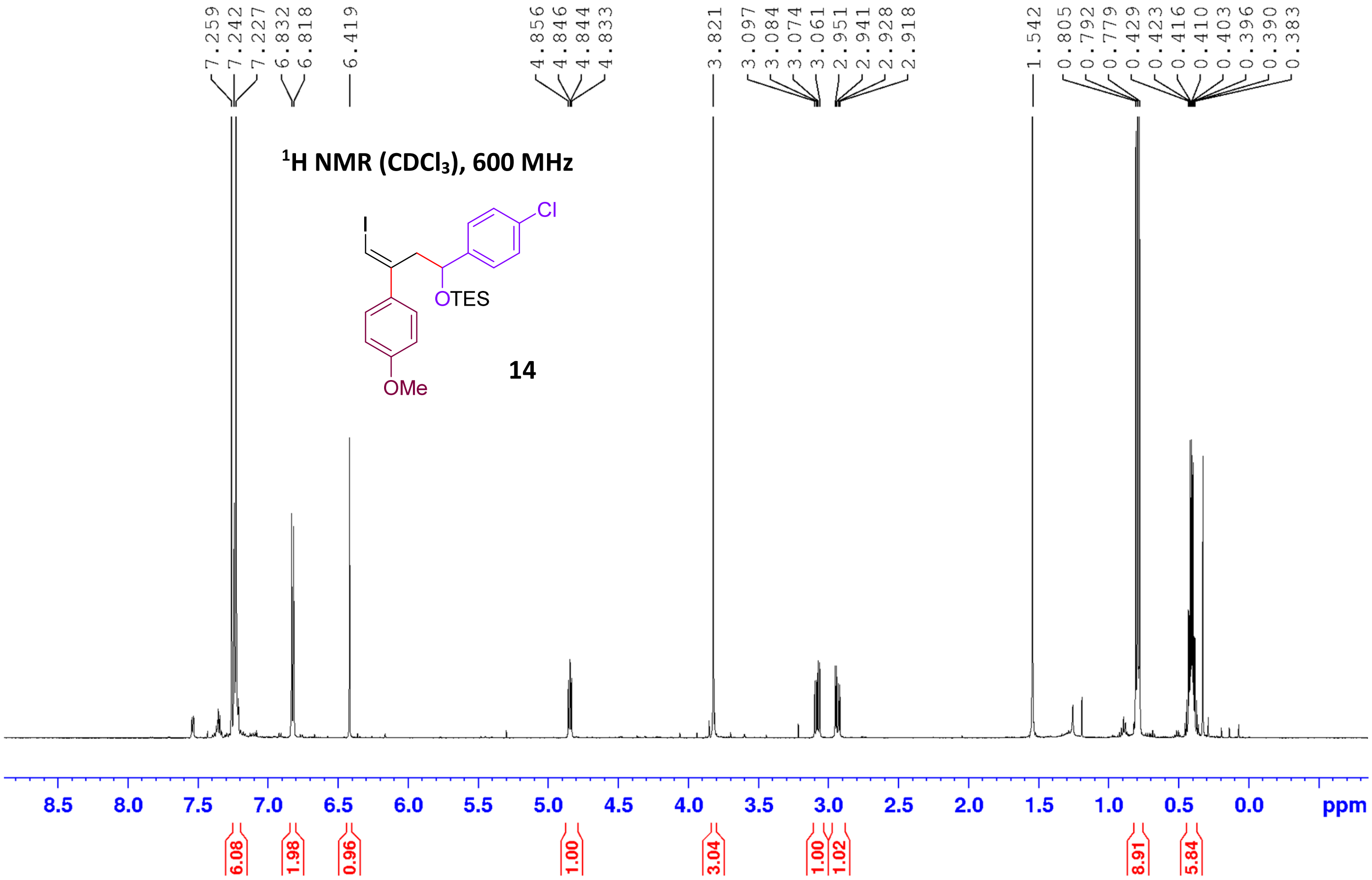
7.259
7.242
7.227
6.832
6.818
6.419
4.856
4.846
4.844
4.833
3.821
3.097
3.084
3.074
3.061
2.951
2.941
2.928
2.918
1.542
0.805
0.792
0.779
0.429
0.423
0.416
0.410
0.403
0.396
0.390
0.383
1H NMR (CDCl3), 600 MHz
I
Cl
OTES
OMe
14
8.5
8.0
7.5
7.0
6.5
6.0
5.5
5.0
4.5
4.0
3.5
3.0
2.5
2.0
1.5
1.0
0.5
0.0
ppm
6.08
1.98
0.96
1.00
3.04
1.00
1.02
8.91
5.84

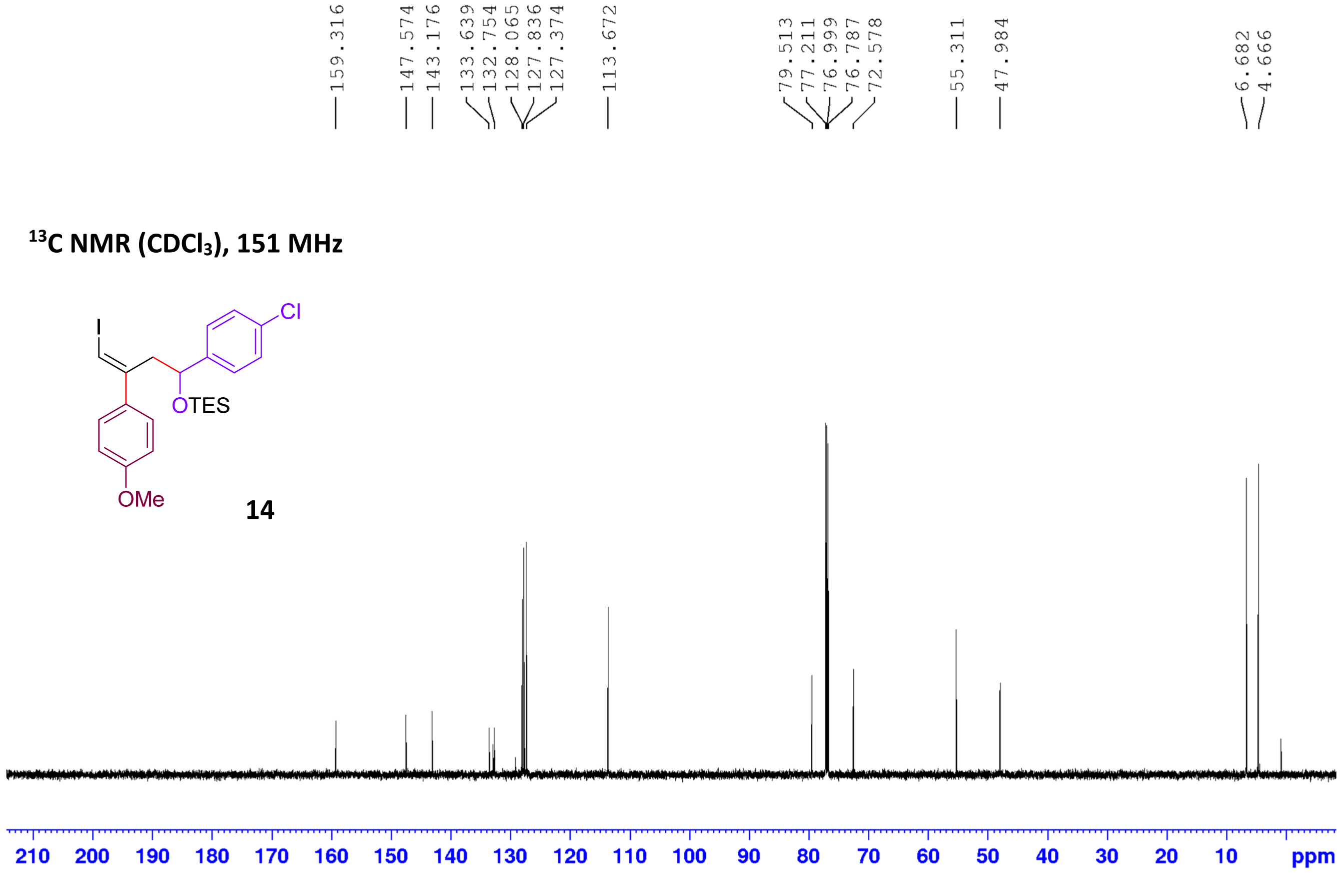
159.316
147.574
143.176
133.639
132.754
128.065
127.836
127.374
113.672
79.513
77.211
76.999
76.787
72.578
55.311
47.984
6.682
4.666
13C NMR (CDCl3), 151 MHz
I
Cl
OTES
OMe
14
210
200
190
180
170
160
150
140
130
120
110
100
90
80
70
60
50
40
30
20
10
ppm

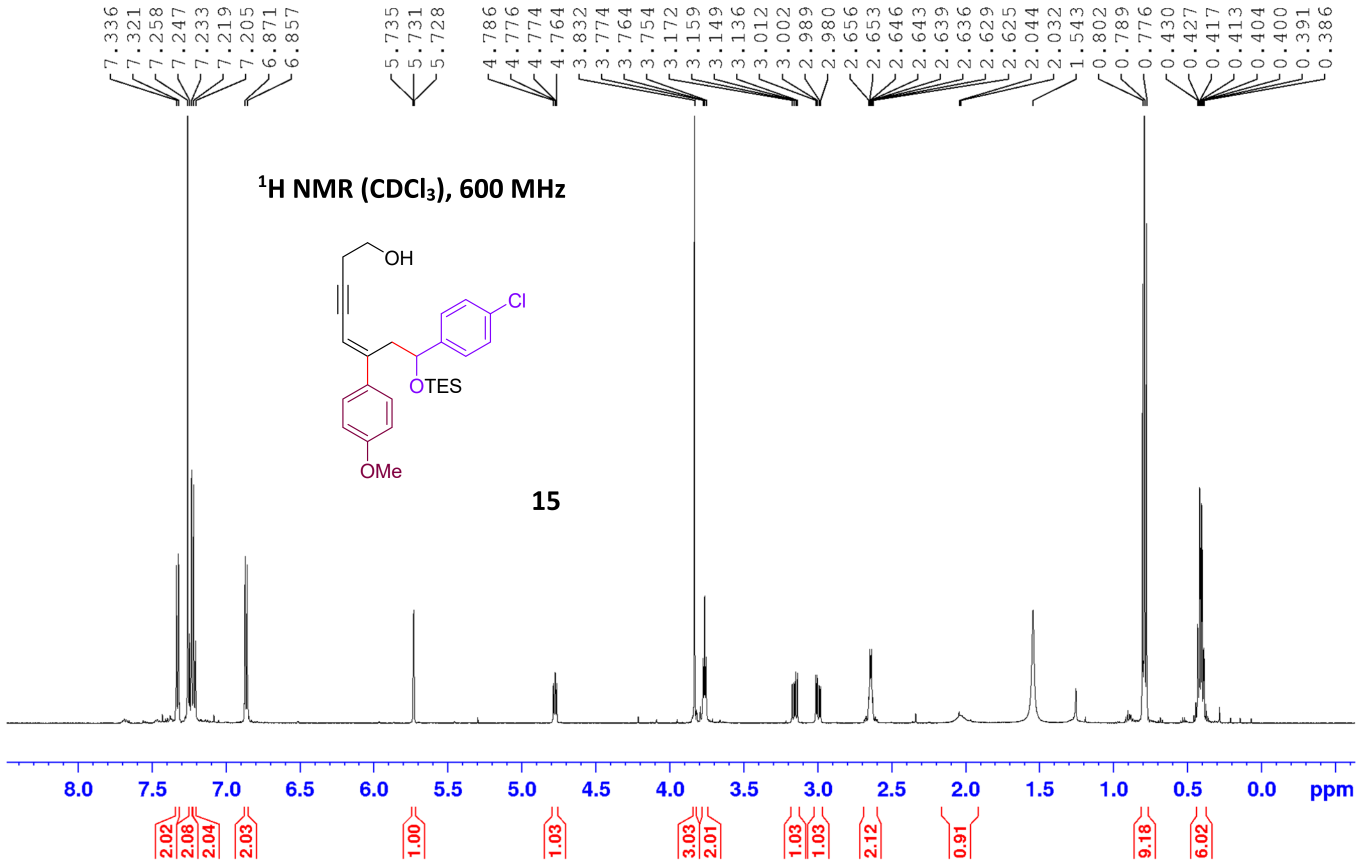

1H NMR (CDCl3), 600 MHz
OH
Cl
OTES
OMe
15
ppm

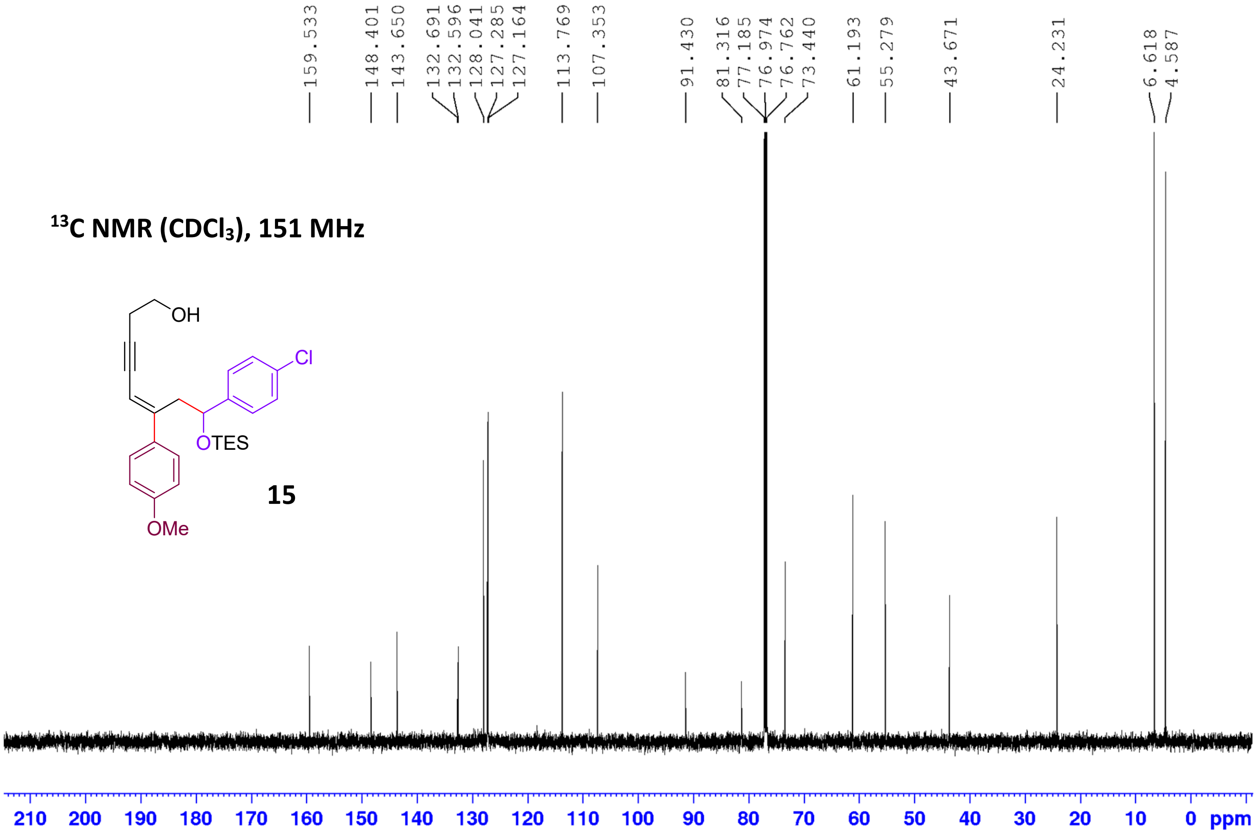

13C NMR (CDCl3), 151 MHz
OH
Cl
OTES
OMe
15
ppm

References.

1. Nogi, K.; Fujihara, T.; Terao, J.; Tsuji, Y. Cobalt-catalyzed carboxylation of propargyl acetates with carbon dioxide. *Chem. Commun.* **2014**, *50*, 13052 -13055.

2. (a) Li, Z.; Yang, C.; Zheng, H.; Qiu, H.; Lai, G. Selective mono- and di-allylation and allenylation of chlorsilanes using indium. *J. Organomet. Chem.* **2008**, *693*, 3771 – 3779. (b) Xu, M.; Lu, Q.; Gong, B.; Ti, W.; Lin, A.; Yao, H.; Gao, S. Copper-catalyzed enantioselective and regiodivergent allylation of ketones with allenylsilanes. *Angew. Chem. Int. Ed.* **2023**, *62*, e202311540.

3. Kuang, J.; Ma, S. An efficient synthesis of terminal allenes from terminal 1-alkynes. *J. Org. Chem.* **2008**, *74*, 1763 – 1765.

4. Boursfield, T. W.; Kimber, M. C. A simple one-pot preparation of *N*-allenyl amides, ureas, carbamates and sulfonamides using a DMSO/^{t}BuOk protocol. *Tetrahedron Lett.* **2015**, *56*, 350 – 352.

5. (a) Ghorai, S.; Chirke, S. S.; Xu, W. -B.; Chen, J. -F.; Li, C. Cobalt-catalyzed regio and enantioselective allylic amination. *J. Am. Chem. Soc.* **2019**, *141*, 11430 – 11434. (b) Wang, L.; Wang, L.; Li, M.; Chong, Q.; Meng, F. Cobalt-catalyzed diastereo- and enantioselective reductive allyl additions to aldehydes with allylic alcohol derivatives via allyl radical intermediates. *J. Am. Chem. Soc.* **2021**, *143*, 12755 – 12765.

6. (a) Zhu, D.; Korobkov, I.; Budzelaar, P. H. M. Radical mechanisms in the reaction of organic halides with diiminepyridine cobalt complexes. *Organometallics* **2012**, *31*, 3958 – 3971. (b) Zhu, D.; Budzelaar, P. H. M. Binuclear oxidative addition of aryl halides. *Organometallics* **2010**, *29*, 5759 – 5761.

7. Rummelt, S. M.; Zhong, H.; Leonard, N. G.; Semproni, S. P. Chirik, P. J. Oxidative addition of dihydrogen, boron compounds, and aryl halides to a cobalt(I) cation supported by a strong-field pincer ligand. *Organometallics* **2019**, *38*, 1081 – 1090.

8. (a) Chan, T. H.; Wang, D. Silylallyl anions in organic synthesis: a study in regio- and stereoselectivity. *Chem. Rev.* **1995**, *95*, 1279 – 1292. (b) Gao, S.; Chen, M. a-Silicon effect assisted Curtin-Hammett allylation using allylcopper reagents derived from 1,3-dienylsilanes. *Chem. Sci.* **2019**, *10*, 7554 – 7560.

9. Pelz, N. F.; Woodward, A. R.; Burks, H. E.; Sieber, J. D.; Morken, J. P. Palladium-catalyzed enantioselective diboration of prochiral allenes. *J. Am. Chem. Soc.* **2004**, *126*, 16328 – 16329.

10. Panayides, J. -L.; Riley, D. L.; Hasenmaile, F.; van Otterlo, W. A. L. The role of silicon in drug discovey: a review. *RSC Med. Chem.* **2024**, *15*, 3286 – 3344.

11. Komeyama, K.; Sakiyama, S.; Iwashita, K.; Osaka, I.; Takaki, K. Three-component coupling of aryl iodides, allenes, and aldehydes catalyzed by a Co/Cr-hybrid catalyst. *Beilstein J. Org. Chem.* **2018**, *14*, 1413 – 1420.
